# Synthesis of 2‑Naphthols via a Trifluoroacetic Acid-Mediated Epoxy Alcohol Rearrangement

**DOI:** 10.1021/acs.joc.6c00021

**Published:** 2026-03-31

**Authors:** Jack B. Story, Kalista E. Ringler, Chi L. Nguyen, Myles J. Drance, Evan M. Howard

**Affiliations:** Department of Chemistry, 6711 Vassar College, 124 Raymond Avenue, Poughkeepsie, New York 15405, United States

## Abstract

Epoxy alcohols derived from 2-benzylidene-1-indanones undergo rearrangement to 2-naphthols when exposed to trifluoroacetic acid. The reaction sequence tolerates a variety of functional groups from both the parent indanone and the benzaldehyde derivatives. No chromatographic purification is required until the final synthetic step, and the epoxy alcohol syntheses use only water and alcohols as solvents. The synthesis is operationally simple, and the 2-naphthol products can be easily derivatized into pharmaceutically relevant compounds.

## Graphical abstract

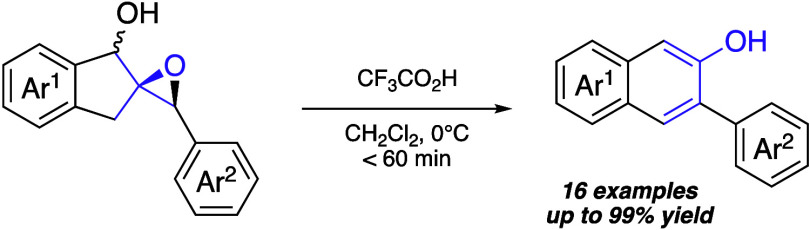

β-Naphthols or 2-naphthols are common structures found in a variety of different types of important compounds including natural products,^1–3^ non-natural medicinal agents,^4–6^ certain types of dyes,^7^ and chiral ligands^8^ (Figure 1).

**Figure 1:**
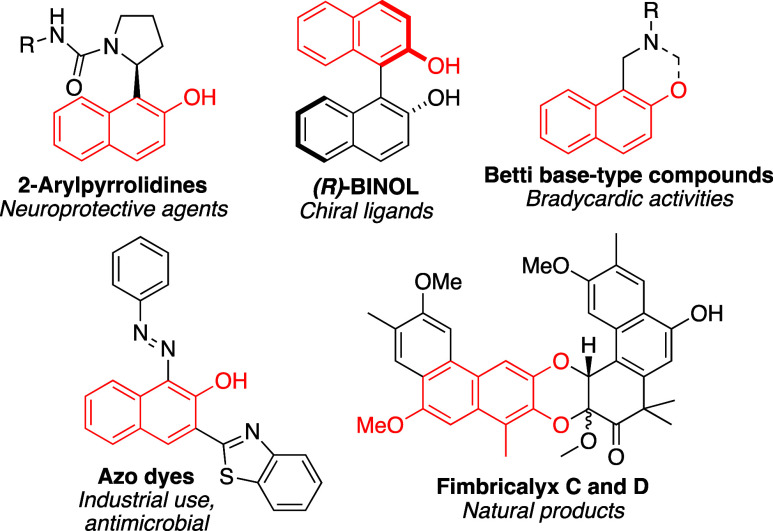
Examples of compounds which contain the 2-naphthol substructure, highlighted in red.

The 2-naphthol substructure is present in many 2-arylpyrrolidines, which are potential candidates to treat neurodegenerative conditions like Parkinson’s and Alzheimer’s diseases, as well as in Betti base-type compounds^9^ which are known to have bradycardic activities.^10^ This motif is also found in many azo dyes, which have numerous uses including a more recent discovery that some azo dyes have a wide range of biologically active properties.^11–13^

Owing to their importance and ubiquity, numerous methods have been developed to synthesize substituted 2-naphthols and each is accompanied by advantages and drawbacks. Functionalization of 2-naphthol itself by traditional electrophilic aromatic substitution is often straightforward, but regioselectivity is an issue and substitutions are typically limited to positions 1, 4, and 6.^14–20^ 2-Naphthols have more recently been synthesized by several different palladium-catalyzed annulation reactions^21–23^ and rhodium-catalyzed cycloaddition reactions.^24–26^ Friedel–Crafts-type reactions leading to 2-naphthols have also been developed, involving the intramolecular electrophilic cyclization of aromatic ynones.^27–30^ Other more recent examples of 2-naphthol synthesis include a Jones reagent-mediated cascade reaction reported by Fan in 2013,^31^ and a base-mediated rearrangement of coumarins reported by Snieckus in 2018 ^32^ (Scheme 1).

**Scheme 1:**
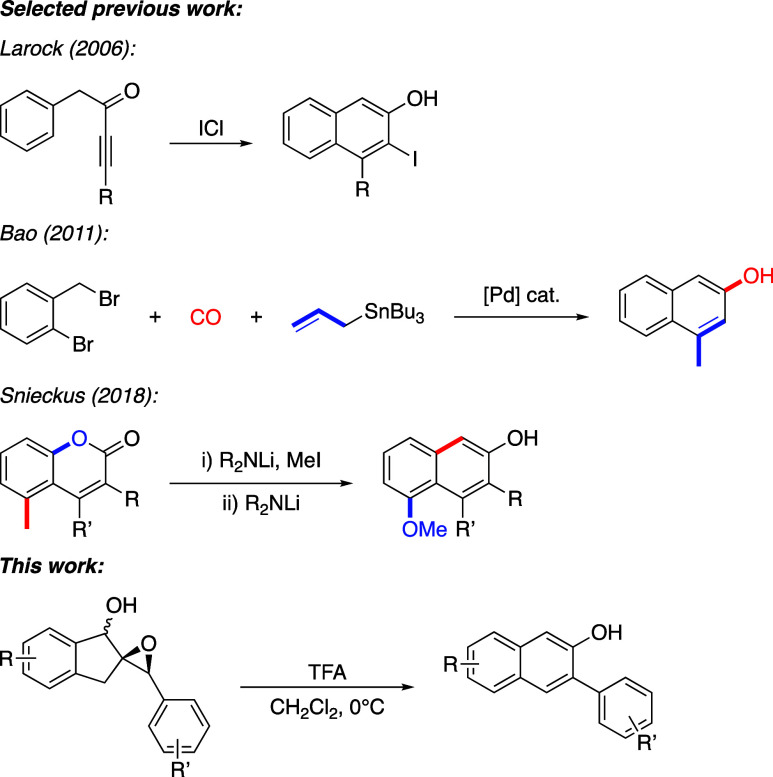
Selected Existing Methods for the Synthesis of 2-Naphthols and This Work

While a litany of methods exist to synthesize substituted 2-naphthols, most are somewhat limited in scope, require the use of objectionable reagents or precious metal catalysts, or are otherwise synthetically laborious. We recently discovered that epoxy alcohol **1a** readily and cleanly underwent rearrangement to 2-naphthol **2a** in 98% yield in under 15 min when exposed to trifluoroacetic acid (Scheme 2).

**Scheme 2:**
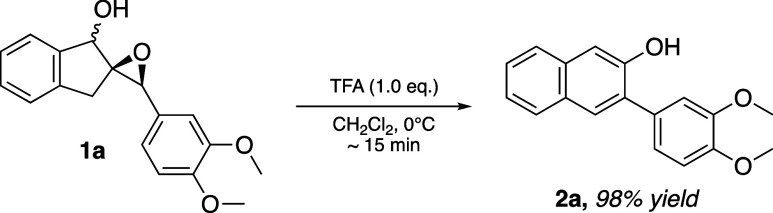
TFA-Mediated Rearrangement of Epoxy Alcohol **1a** to 2-Naphthol **2a**

Recrystallization by vapor diffusion of pentane into a saturated CH_2_Cl_2_ solution of **2a** enabled its unambiguous structure determination by single-crystal X-ray diffraction (Figure 2).

**Figure 2:**
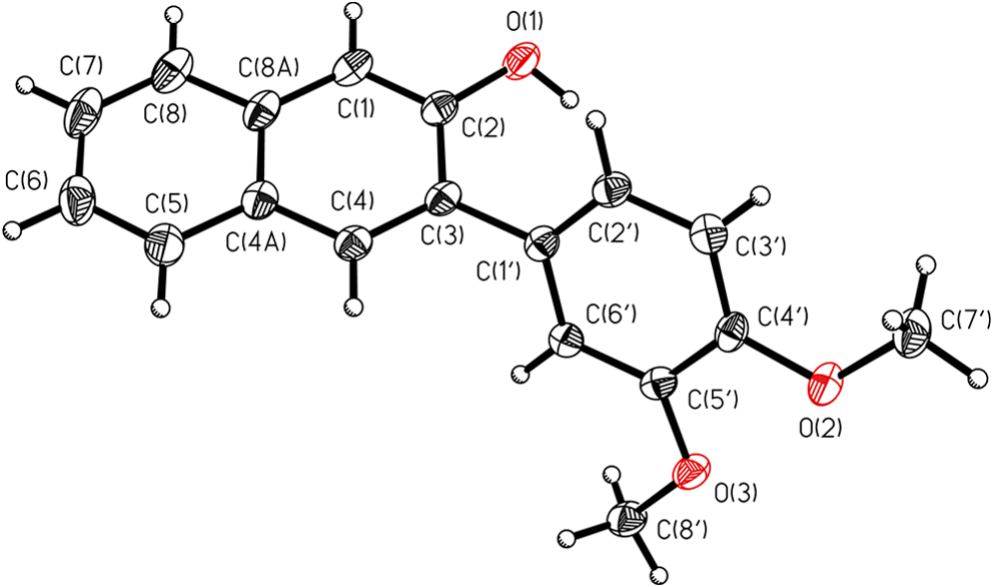
Oak Ridge Thermal Ellipsoid Plot showing the crystal structure of **2a**. 50% probability anisotropic displacement ellipsoids are shown.

Inspired by the ease with which we obtained **2a** and recognizing the potential to use this chemistry to synthesize a library of 2-naphthols with unique substitution patterns, we examined the scope of this reaction sequence. **1a** and its analogues thereof are readily synthesized on a gram scale in three operationally simple steps from 1-indanone derivatives and substituted benzaldehydes using known procedures (Scheme 3). Importantly, none of these synthetic steps require chromatographic purification, as the products precipitate cleanly out of the reaction mixture in both the aldol condensation and epoxidation steps. The epoxy alcohol is then easily isolated after borohydride reduction by simple aqueous extraction, normally as a mixture of inconsequential diastereomers.

**Scheme 3:**
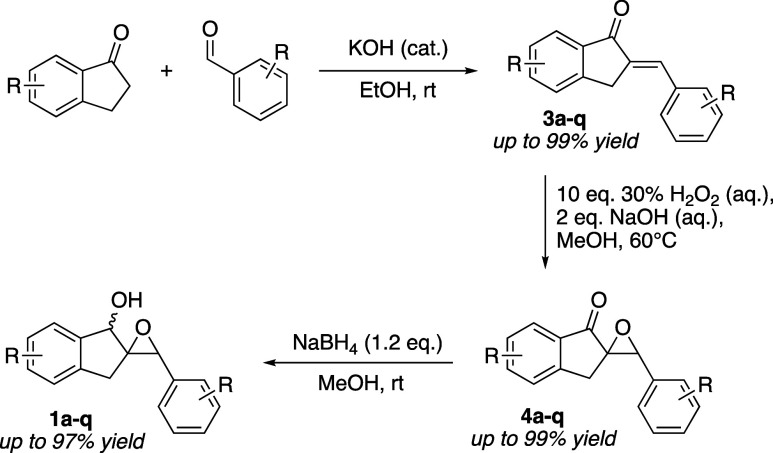
Synthesis of Epoxy Alcohols **1a**–**q**

This rearrangement/aromatization reaction is reasonably general, although somewhat sensitive to both the steric and electronic profiles of the aromatic rings Ar^1^, originating from the indanone, and Ar^2^, originating from the benzaldehyde. Varying the substituents on either aromatic ring revealed that the reaction is more efficient when either of the rings is electron-rich in some capacity, although this is a loose correlation.

As shown in Table 1, epoxy alcohols bearing an electron-rich Ar^1^ performed best, yielding naphthols **2a**–**c** in good to excellent yields. **1g** also performed moderately well. In the cases of **1d**–**f**, the electronics of the aromatic ring seem to be less of a factor; naphthols **2d** and **2f** were isolated in identical 25% yields, despite being electronically very different. In these cases, the substituents at C5 of the naphthol and the hydrogen atom at C4 would experience unavoidable 1,3-allylic strain, thereby affecting product stability and the rate of its formation. This is corroborated by the only marginally improved 46% yield of **2e**, which has a slightly smaller C5 substituent but still would experience an A^1,3^ strain. Although Ar^1^ of naphthol **2h** is electron-rich, it was isolated in only 20% yield. We attribute this to a competing acid-mediated Claisen rearrangement of the allyl group; at least two regioisomeric products of this side-reaction were observed in the crude reaction mixture. Importantly, **2a** can easily be synthesized on a gram scale, in 99% yield (4.40 g) from epoxy alcohol **1a** and 78% overall yield from 1-indanone.

**Table 1:**
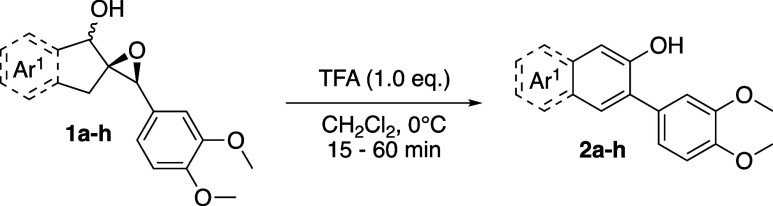

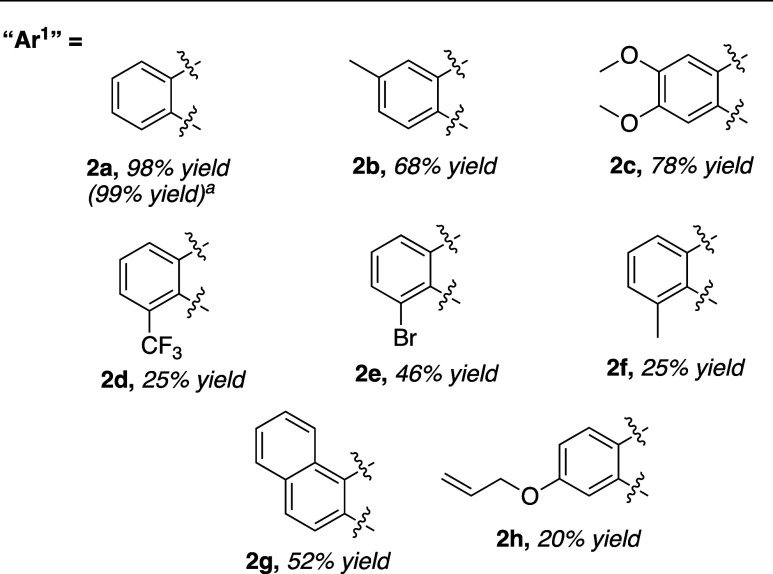
Substrate Scope for the Rearrangement of Epoxy Alcohols **1a**–**h**

a Yield on a multigram scale.

Varying Ar^2^ revealed trends similar to those of Ar^1^ (Table 2). Naphthols **2i**–**k** were isolated in good yields, notably **2j**, which was isolated in near-quantitative yield. As expected, due to the more electron-poor nature of their aromatic rings, **2m** and **2n** were isolated in diminished yields of 54 and 33%, respectively. Seemingly counterintuitive, this observation is actually consistent with the known Hammett parameters of *p*-F (σ = 0.06) and *p*-Br (σ = 0.23). The corresponding *p*-F and *p*-Br benzyl cations have parameters σ^+^ = −0.07 and +0.15, respectively.^33–36^ Because of the stronger electron-withdrawing nature of *p*-Br, and the weakly stabilizing effect of *p*-F versus the moderately destabilizing effect of *p*-Br, the cationic intermediate which would lead to **2m** is substantially easier to form than the one which leads to **2n**. Introducing substitution at the 2′-position of Ar^2^ had much the same effect on the efficiency of this reaction as did substitution at the 4-position of Ar^1^; **2o** and **2p** were isolated in nearly identical, diminished yields (33 and 34%, respectively). One might find this result intriguing given their electronic difference, but the steric inhibition of resonance^37^ experienced by either the 2′-alkoxy group^38^ or the 2′-chloride renders them roughly equal in terms of electron-withdrawing, destabilizing effects on a benzylic cation.^35^ For reasons we do not understand, the epoxy alcohol bearing two otherwise unsubstituted aromatic rings (Ar^1^ = Ar^2^ = phenyl) delivered only intractable mixtures upon exposure to TFA. In all cases, the theoretically possible 1-naphthol isomer was not observed.

**Table 2:**
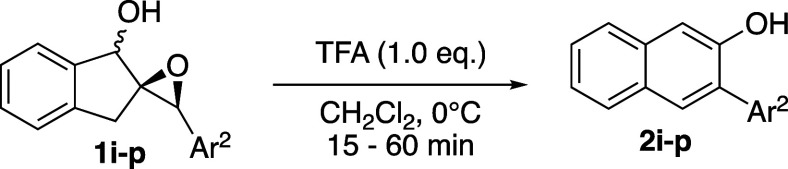

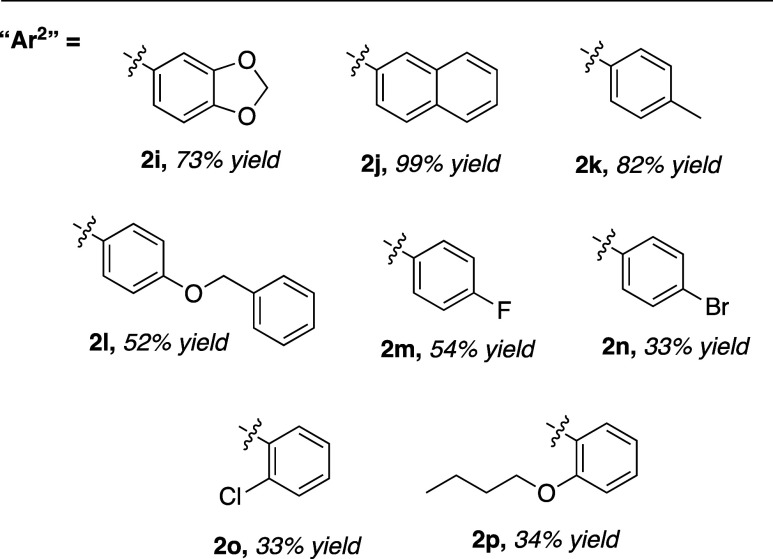
Substrate Scope for the Rearrangement of Epoxy Alcohols **1i**–**p**

We then attempted to replace the C3 aromatic group on the naphthol product with an alkyl group. To this end, we prepared epoxy alcohol **1q** and exposed it to TFA. While the synthesis of **1q** proceeded without incident, instead of isolating the expected naphthol **2q** upon reaction with TFA, the major product isolated was enone **3q** (68% yield), with no detectable amount of the expected 3-*tert-*butyl-2-naphthol **2q** (Scheme 4).

**Scheme 4:**
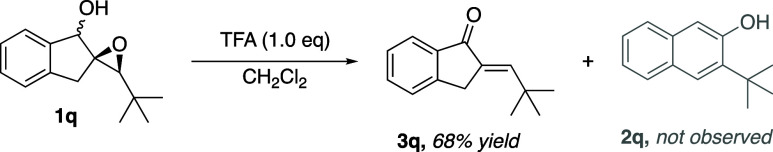
Attempted Rearrangement of Epoxy Alcohol **1q** Resulting in Enone **3q** Instead of a 2-Naphthol

These results, taken together, lead us to propose the general mechanism illustrated in Scheme 5. There are several likely driving forces behind this process. In the initial steps, substrates **1** experience significant relief of ring strain, both from the TFA-mediated epoxide opening to benzyl cations **6** and the 5-to-6 ring expansion forming the proposed intermediates **7**. A series of proton transfer events and a formal dehydration process then occur, surely motivated by the formation of the stable extended aromatic system. Formal mechanistic studies of this reaction are ongoing.

**Scheme 5:**
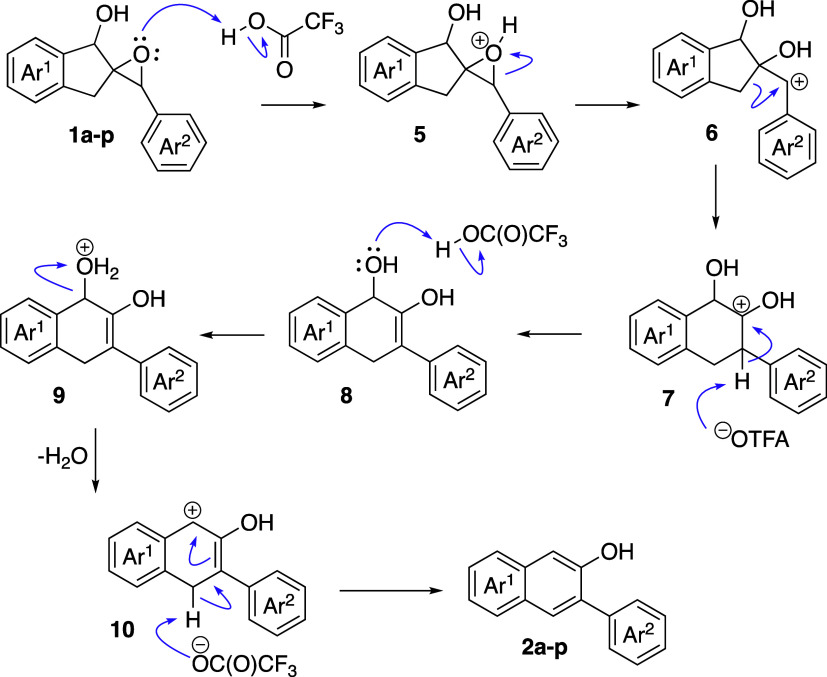
Proposed Mechanism of the TFA-Mediated Rearrangement of Spirocyclic Epoxy Alcohols

The failure of **1q** to convert to **2q** is consistent with this mechanism. In order to form **2q**, the pathway which would lead to productive naphthol formation must involve the protonated epoxide analogous to **5** opening to generate a secondary, nonstabilized carbocation. Epoxide opening in the other direction to generate a tertiary cation in this case is more likely, which through a subsequent series of proton transfer and elimination reactions would yield enone **3q**. Consistent with this mechanism as well, given that intermediates **10** must be deprotonated to generate **2a**–**q**, is the notion that this reaction could be rendered catalytic in acid. Attempts were made to this end (Table 3) and preliminary results suggest that Brønsted acids could behave catalytically in the reaction (entries 4 and 5). For the purposes of this study, though, the use of stoichiometric TFA was deemed the most prudent method.

**Table 3:**
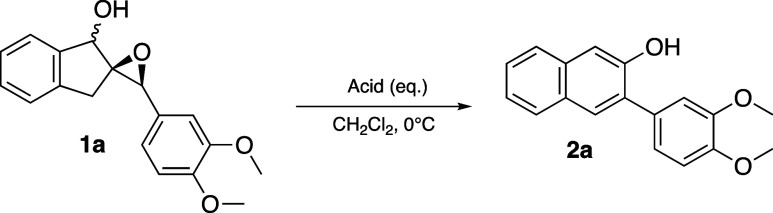
Survey of Alternative Acid Mediators/Catalysts for the Conversion of **1a** to **2a**

entry	acid	equiv	% yield **2a** ^a^
1	TFA	1.0	98%
2	BF_3_·Et_2_O	1.0	<5%
3	SnCl_4_	1.0	40%
4	TFA	0.2	46%
5	H_2_SO_4_	0.2	41%
6	HCl (conc.)	0.2	17%
7	AcOH	0.2	0%
8	AlCl_3_	0.2	16%

a Determined by ^1^H NMR of the crude reaction mixture using BHT (2,6-di-*tert*-butyl-4-methylphenol) as an internal standard.

In order to demonstrate the utility and applications of the 2-naphthol products synthesized in this study, we subjected **2a** to two different derivatization reactions (Scheme 6).

**Scheme 6:**
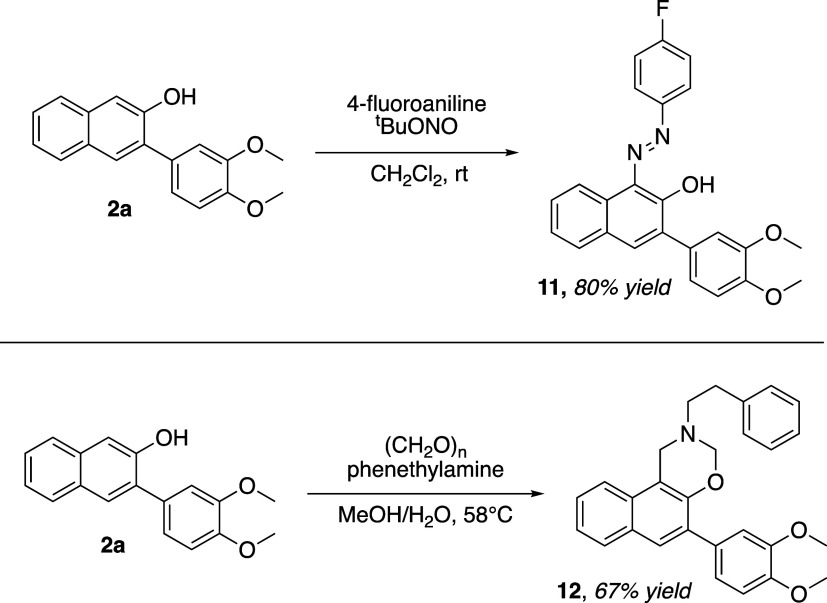
Derivatization Reactions of 2-Naphthol **2a**

Azo coupling of **2a** with 4-fluoroaniline in the presence of *tert*-butyl nitrite^39^ provided diazene **11** in 80% yield as a deeply colored reddish-purple solid with no other detectable regioisomers. When **2a** was subjected to Betti reaction conditions using excess aqueous formaldehyde and phenethylamine, hemiaminal **12** was isolated in 67% yield. The structures of **11** and **12** map nicely onto potential analogues of existing antimicrobial azo dyes and bradycardic Betti base-type compounds, respectively, and the ease with which they could be synthesized using the route presented here will enable the preparation of libraries of each of these types of compounds for biological evaluation.

In summary, we have developed a rapid and operationally simple route for the synthesis of 2-naphthols from 2-benzylidene-1-indanones. The preparation of epoxy alcohol starting materials **1a**–**q** does not require chromatographic purification and uses only water and alcohols as solvents. The TFA-mediated rearrangement/aromatization reaction is reasonably tolerant of different functionalities originating from both the parent indanone and benzaldehyde derivatives. We also demonstrated that the products could easily be derivatized into industrially relevant compounds. Further exploration into related scaffolds and compounds that could be accessed using this chemistry, as well as mechanistic studies, will be reported in due course.

## Supplementary Material



## Data Availability

The data underlying this study are available in the published article, in its Supporting Information, and openly available in Dryad at 10.5061/dryad.rbnzs7hsw.

## Notes

Experimental procedures and characterization data for new compounds; Crystallographic data for 2a (PDF)

## References

[1] Seephonkai P., Pyne S. G., Willis A. C., Lie W. (2013). Bioactive Compounds
from the Roots of Strophioblachia Fimbricalyx. J. Nat. Prod..

[2] Fang X., Hu X. (2018). Advances in the Synthesis
of Lignan Natural Products. Molecules.

[3] Ward R. S. (1995). Lignans,
Neolignans, and Related Compounds. Nat. Prod.
Rep..

[4] Baichwal R. S., Baichwal M. R., Khorana M. L. (1957). Antibacterial and Antifungal Properties
of β-Naphthol Derivatives IV. J. Am. Pharm.
Assoc..

[5] Baichwal R. S., Baichwal M. R., Khorana M. L. (1958). Antibacterial and Antifungal Properties
of β-Naphthol Derivatives V. J. Am. Pharm.
Assoc..

[6] Vargas J. A. M., Day D. P., Burtoloso A. C. B. (2021). Substituted Naphthols: Preparations,
Applications, and Reactions. Eur. J. Org. Chem..

[7] Benkhaya S., M’rabet S., Harfi A. E. (2020). Classifications, Properties, Recent
Synthesis and Applications of Azo Dyes. Heliyon.

[8] Brunel J. M. (2005). BINOL:
A Versatile Chiral Reagent. Chem. Rev..

[9] Olyaei A., Sadeghpour M. (2019). Recent Advances
in the Synthesis and Synthetic Applications
of Betti Base (Aminoalkylnaphthol) and Bis-Betti Base Derivatives. RSC Adv..

[10] Shen A. Y., Tsai C. T., Chen C. L. (1999). Synthesis
and Cardiovascular Evaluation
of N-Substituted 1-Aminomethyl-2-Naphthols. Eur. J. Med. Chem..

[11] Bansal R., Narang G., Zimmer C., Hartmann R. W. (2011). Synthesis of Some
Imidazolyl-Substituted 2-Benzylidene Indanone Derivatives as Potent
Aromatase Inhibitors for Breast Cancer Therapy. Med. Chem. Res..

[12] Mezgebe K., Mulugeta E. (2022). Synthesis and Pharmacological Activities
of Azo Dye
Derivatives Incorporating Heterocyclic Scaffolds: A Review. RSC Adv..

[13] Rizk H. F., Ibrahim S. A., El-Borai M. A. (2015). Synthesis,
Fastness Properties, Color
Assessment and Antimicrobial Activity of Some Azo Reactive Dyes Having
Pyrazole Moiety. Dyes Pigments.

[14] Fieser L. F. (1931). 1-amino-2-naphthol-4-sulfonic
acid. Org. Synth..

[15] Arsenijevic L., Arsenijevic V., Horeau A., Jacques J. (1973). 2-acetyl-6-methoxynaphthalene. Org. Synth..

[16] Russel A., Lockhart L. B. (1942). 2-hydroxy-1-naphthaldehyde. Org.
Synth..

[17] Koelsch C. F. (1940). 6-bromo-2-naphthol. Org. Synth..

[18] Lima D. B., Santos P. H. V., Fiori P., Badshah G., Luz E. Q., Seckler D., Rampon D. S. (2019). Base-Promoted Direct
Chalcogenylation
of 2-Naphthols. ChemistrySelect.

[19] de
Koning C. B., Rousseau A. L., van Otterlo W. A. L. (2003). Modern
Methods for the Synthesis of Substituted Naphthalenes. Tetrahedron.

[20] Smith W. B. (1985). Some Observations
on the Iodination of 2-Naphthol and Its Methyl Ether. J. Org. Chem..

[21] Cai J., Wang Z.-K., Zhang Y.-H., Yao F., Hu X.-D., Liu W.-B. (2020). Synthesis of Polysubstituted 2-Naphthols
by Palladium-Catalyzed
Intramolecular Arylation/Aromatization Cascade. Adv. Synth. Catal..

[22] Dai Y., Feng X., Liu H., Jiang H., Bao M. (2011). Synthesis
of 2-Naphthols via Carbonylative Stille Coupling Reaction of 2-Bromobenzyl
Bromides with Tributylallylstannane Followed by the Heck Reaction. J. Org. Chem..

[23] Chen Z., Duan H.-Q., Jiang X., Zhu Y.-M., Ji S.-J., Yang S.-L. (2015). Palladium-Catalyzed
Domino Synthesis of 4-Amino-3-Acyl-2-
Naphthols via Isocyanide Chemoselective Insertion. J. Org. Chem..

[24] Wang L., Yu Y., Yang M., Kuai C., Cai D., Yu J., Cui X. (2017). Rhodium-Catalyzed
Synthesis of Multiaryl-Substituted Naphthols via
a Removable Directing Group. Adv. Synth. Catal..

[25] Hanchate V., Kumar A., Prabhu K. R. (2019). Synthesis of Naphthols by Rh­(III)-Catalyzed
Domino C–H Activation, Annulation, and Lactonization Using
Sulfoxonium Ylide as a Traceless Directing Group. Org. Lett..

[26] Han S. H., Pandey A. K., Lee H., Kim S., Kang D., Jung Y. H., Kim H. S., Hong S., Kim I. S. (2018). One-Pot
Synthesis of 2-Naphthols from Nitrones and MBH Adducts via Decarboxylative
N–O Bond Cleavage. Org. Chem. Front..

[27] Kim H. Y., Oh K. (2014). A Facile Access to
4-Substituted-2-Naphthols via a Tandem Friedel–Crafts
Reaction: A β-Chlorovinyl Ketone Pathway. Org. Lett..

[28] Shan C., Li R., Wang X. (2024). Efficient Construction of a β-Naphthol Library
under Continuous Flow Conditions. RSC Adv..

[29] Zhang X., Sarkar S., Larock R. C. (2006). Synthesis
of Naphthalenes and 2-Naphthols
by the Electrophilic Cyclization of Alkynes. J. Org. Chem..

[30] Juteau H., Gareau Y., Lachance H. (2005). Synthesis of Polysubstituted-2-Naphthols. Tetrahedron Lett..

[31] He Y., Zhang X., Shen N., Fan X. (2013). Tunable Synthesis of
3-Acyl-2-Naphthols and 3-Substituted Isocoumarins via Jones Reagent
Promoted Cascade Reactions of 2-(4-Hydroxy-but-1-Ynyl)­Benzaldehydes. J. Org. Chem..

[32] Patel J. J., Laars M., Gan W., Board J., Kitching M. O., Snieckus V. (2018). Directed Remote Lateral
Metalation: Highly Substituted
2-Naphthols and BINOLs by In Situ Generation of a Directing Group. Angew. Chem..

[33] Kim C. K., Han I. S., Ryu W. S., Lee H. W., Lee B.-S., Kim C. K. (2006). Unusual π-Donating
Effects of π-Accepting
Substituents on the Stabilities of Benzylic Cations: A Theoretical
Study. J. Phys. Chem. A.

[34] Hansch, C. Substituent Constants for Correlation Analysis in Chemistry and Biology; Wiley: New York, 1979. 10.1021/jm00212a024 836503

[35] Hansch C., Leo A., Taft R. W. (1991). A Survey
of Hammett Substituent Constants and Resonance
and Field Parameters. Chem. Rev..

[36] Fernández I., Frenking G. (2006). Correlation between
Hammett Substituent Constants and
Directly Calculated π-Conjugation Strength. J. Org. Chem..

[37] Arnold R. T., Peirce G., Barnes R. A. (1940). The steric
inhibition of resonance. J. Am. Chem. Soc..

[38] Fujio M., Keeffe J. R., More
O’Ferrall R. A., O’Donoghue A.
C. (2004). Unexpectedly
Small Ortho-Oxygen Substituent Effects on Stabilities of Benzylic
Carbocations. J. Am. Chem. Soc..

[39] Liu T.-T., Yan J.-Z., Cheng X.-W., Duan P., Zeng Y.-F. (2021). One-Pot
Synthesis of Azo Compounds in the Absence of Acidic or Alkaline Additives. J. Chem. Res..

