# Supplementary material for: Synthesis of 2‑Naphthols via a Trifluoroacetic Acid-Mediated Epoxy Alcohol Rearrangement
Source: J Org Chem. 2026 Mar 31;91(14):5249–54. doi: 10.1021/acs.joc.6c00021 (PMC13077693; doi:10.1021/acs.joc.6c00021)
Supplement: Supplementary file 1 [file jo6c00021_si_001.pdf]

Supporting Information

Synthesis of 2-Naphthols via a Trifluoroacetic Acid Mediated Epoxy Alcohol Rearrangement

*Jack B. Story, Kalista E. Ringler, Chi Nguyen, Myles J. Drance, and Evan M. Howard**

Department of Chemistry, Vassar College
124 Raymond Avenue, Poughkeepsie, NY 12604, United States
ehoward@vassar.edu

Table of Contents

General Experimental Details & Procedures…S3

Characterization Data for New Compounds…S4

X-Ray Crystal Data for 2a…S17

References…S18

^{1}H, ^{13}C, ^{19}F NMR Spectra…S19

Enones…S19
Epoxy ketones…S38
Epoxy Alcohols…S65
β-Naphthols….S101

GENERAL EXPERIMENTAL DETAILS

All reactions were performed in flame-dried glassware under an atmosphere of nitrogen unless otherwise specified. All commercially available reagents were used as received, unless otherwise specified. Known compounds which are not commercially available were prepared according to existing literature procedures. 200-proof ethanol was kept dry by storing over activated 4Å molecular sieves or was prepared by drying 190-proof ethanol over activated 4Å molecular sieves for a minimum of 12 hours prior to use. Dichloromethane (CH_2Cl_2) was degassed by sparging with active nitrogen for at least 30 minutes and dried over activated 4Å molecular sieves for at least 4 hours prior to use. TLC analysis was performed on aluminum-backed silica gel plates. Plates were visualized using UV light or iodine vapor staining. Reaction mixtures were concentrated using a Büchi R-210 rotary evaporator equipped with a digital self-cleaning vacuum pump, cold-water condenser, and digitally controlled warm water bath. Flash chromatography was performed on a Teledyne ISCO CombiFlash® R_f 200 automated chromatography system using pre-packaged silica gel columns.

^{1}H and ^{13}C NMR spectra were recorded at room temperature on a Bruker Avance III NMR spectrometer at 400 MHz and 100 MHz, respectively. Chemical shifts are reported in ppm (δ units) downfield from tetramethylsilane. ^{1}H NMR spectra are referenced to the TMS signal at 0.00 ppm or the $CDCl_3$ solvent residual peak at 7.26 ppm. ^{13}C NMR spectra are referenced to the $CDCl_3$ solvent residual peak at 77.16 ppm. ^{19}F spectra are uncorrected and decoupled from ^{1}H and ^{13}C. IR data were collected neat on a Thermo Nicolet iS50 FTIR spectrometer and values are reported in wavenumbers (cm^{-1}). Exact mass analysis was performed using an Agilent 6520 QToF LCMS operated in positive or negative ESI mode. Single-crystal X-ray diffraction was performed using a Bruker APEX-II CCD X-Ray diffractometer.

EXPERIMENTAL PROCEDURES

General Procedure A: Preparation of 2-benzylidene-1-indanones

If aldehyde is solid: A flame-dried roundbottom flask equipped with a magnetic stir bar, open to air, was charged with the requisite indanone (1.0 eq.) and aldehyde (1.0 eq.). 200-proof ethanol (2 mL/mmol) was added to the flask, and the suspension was stirred rapidly (>450 rpm) until a homogenous or slightly turbid solution was achieved. A catalytic amount of solid KOH (1-3 pellets) was then added to the reaction mixture. Near-immediate formation of a precipitate was observed and stirring was continued until TLC analysis of the supernatant indicated that the starting aldehyde was consumed. The reaction flask was then placed on ice for a minimum of 1 hour, at which point the precipitated solids were collected by vacuum filtration to afford the desired 2-benzylidene-1-indanones. Further purification was typically not required.

If aldehyde is liquid: A flame-dried roundbottom flask equipped with a magnetic stir bar, open to air, was charged with the requisite indanone (1.0 eq.). 200-proof ethanol (1.5 mL/mmol) was added to the flask followed by 2M aqueous NaOH (1.5 mL/mmol). The mixture was stirred until a homogeneous solution was achieved, at which point the requisite aldehyde (1.0 eq.) was added in one portion. Near-immediate formation of a precipitate was observed and stirring was continued until TLC analysis indicated that the starting aldehyde was consumed. The reaction flask was then placed on ice for a minimum of 1 hour, at which point the precipitated solids were collected by vacuum filtration to afford the desired 2-benzylidene-1-indanones. Further purification was typically not required.

General Procedure B: Epoxidation of 2-benzylidene-1-indanones

A flame-dried roundbottom flask equipped with a magnetic stir bar, open to air, was charged with the requisite 2-benzylidene-1-indanone (1.0 eq.). Methanol (4 mL/mmol) and 2M aqueous NaOH (2.0 eq.) were then added each in one portion, followed by 30% aqueous hydrogen peroxide (9 - 10 eq.) in a slow stream. The reaction flask was then placed in a pre-heated oil bath at 60°C and affixed with a rubber septum which was pierced with a vent needle. The reaction was monitored by aliquot NMR until the 2-benzylidene-1-indanone was consumed, at which point the flask was cooled in the refrigerator overnight. The resulting solids were then collected by vacuum filtration to afford the desired epoxides. Further purification was typically not required.

General Procedure C: Reduction of epoxy ketones

A flame-dried roundbottom flask equipped with a magnetic stir bar was charged with the requisite epoxy ketone (1.0 eq.). The solids were then suspended in methanol (4 mL/mmol) and the reaction flask cooled to 0°C on an ice-water bath. $NaBH_4$ (1.2 eq.) was then added in portions so as to control the evolution of gas. The reaction was monitored by TLC analysis until complete consumption of the starting material was observed, at which point the reaction mixture was concentrated *in vacuo* to remove most of the MeOH. The resulting suspension was then partitioned between CH_2Cl_2 and water. The organic phase was then separated and the aqueous phase extracted 3 times with CH_2Cl_2. The combined organic extracts were dried over $MgSO_4$ and concentrated *in vacuo* to afford the desired epoxy alcohols as inconsequential mixtures of diastereomers. These epoxy alcohols were typically of reasonable purity and used without further purification.
NOTE: It is important to stop the reduction reactions as soon as they are deemed complete. Unnecessarily prolonged reaction times have proven deleterious to mass recovery.

General Procedure D: **TFA-mediated epoxy alcohol rearrangement**

A flame-dried roundbottom flask equipped with a magnetic stir bar, under nitrogen, was charged with the requisite epoxy alcohol (1.0 eq.). The material was then dissolved in CH_2Cl_2 (5 mL/mmol) and cooled to 0°C on an ice-water bath. Trifluoroacetic acid (1.0 eq.) was then added dropwise *via* syringe. The reaction was monitored by TLC analysis. Once deemed complete, the reaction mixture was directly concentrated *in vacuo* to remove all volatiles. The residue was then purified by flash chromatography on SiO_2 to give the 2-naphthol products.

CHARACTERIZATION DATA FOR NEW COMPOUNDS

---2-BENZYLIDENE-1-INDANONES---

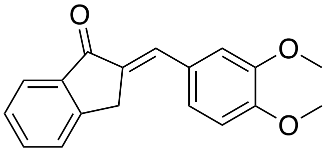

2-(3,4-dimethoxybenzylidene)-1-indanone (3a): Prepared according to General Procedure A from 1-indanone (5.00 g, 37.8 mmol, 1.0 eq.) and 3,4-dimethoxybenzaldehyde (6.29 g, 37.8 mmol, 1.0 eq.). Collection of the precipitated solids by vacuum filtration followed by air drying for 1 hour afforded the analytically pure title compound as a yellow solid (10.44 g, 99% yield). Spectral data matched those previously reported.[1]

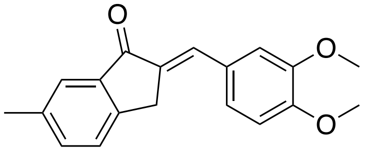

2-(3,4-dimethoxybenzylidene)-6-methyl-1-indanone (3b): Prepared according to General Procedure A from 6-methyl-1-indanone (670 mg, 4.58 mmol, 1.0 eq.) and 3,4-dimethoxybenzaldehyde (761 mg, 4.58 mmol, 1.0 eq.). Collection of the precipitated solids by vacuum filtration followed by air drying for 1 hour afforded the analytically pure title compound as a yellow solid (1.26 g, 89% yield). HRMS (ESI) m/z: $[M+H]^+$ calculated for $C_{19}H_{19}O_3$ 295.1329; found 295.1334. ^1H NMR (400 MHz, $CDCl_3$) δ 7.70 (dt, *J* = 1.7, 0.8 Hz, 1H), 7.60 (t, *J* = 2.1 Hz, 1H), 7.46 – 7.39 (m, 2H), 7.29 (dd, *J* = 8.4, 2.1 Hz, 1H), 7.18 (d, *J* = 2.0 Hz, 1H), 6.95 (d, *J* = 8.4 Hz, 1H), 3.96 (s, 5H), 3.94 (s, 3H), 2.43 (s, 3H). $^{13}C\{1H\}$ NMR (101 MHz, $CDCl_3$) δ 194.5, 150.7, 149.2, 146.9, 138.5, 137.7, 135.7, 133.9, 133.3, 128.7, 125.9, 124.7, 124.5, 113.6, 111.4, 56.1, 32.2, 21.3.

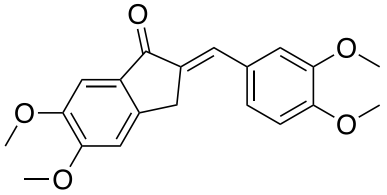

5,6-dimethoxy-2-(3,4-dimethoxybenzylidene)-1-indanone (3c): Prepared according to General Procedure A from 5,6-dimethoxy-1-indanone (1.50 g, 7.80 mmol, 1.0 eq.) and 3,4-dimethoxybenzaldehyde (1.30 g, 7.80 mmol, 1.0 eq.). Collection of the precipitated solids by vacuum filtration followed by air drying for 1 hour afforded the analytically pure title compound as a light yellow solid (2.65 g, >99% yield). Spectral data matched those previously reported.[2]

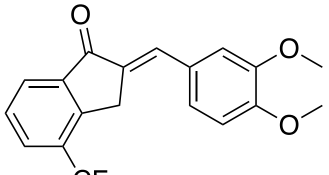

2-(3,4-dimethoxybenzylidene)-4-(trifluoromethyl)-1-indanone (3d): Prepared according to General Procedure A from 4-(trifluoromethyl)-1-indanone (504 mg, 2.52 mmol, 1.0 eq.) and 3,4-dimethoxybenzaldehyde (419 mg, 2.52 mmol, 1.0 eq.). Collection of the precipitated solids by vacuum filtration followed by air drying for 1 hour afforded the analytically pure title compound as a yellow solid (702 mg, 80% yield). HRMS (ESI) m/z: $[M+H]^+$ calculated for $C_{19}H_{16}F_3O_3$ 349.1046, found 349.1051. ^1H NMR (400 MHz, $CDCl_3$) δ 8.10 (d, *J* = 7.6 Hz, 1H), 7.90 – 7.86 (m, 1H), 7.70 (t, *J* = 2.1 Hz, 1H), 7.58 (td, *J* = 7.7, 1.0 Hz, 1H), 7.35 (dd, *J* = 8.4, 2.1 Hz, 1H), 7.20 (d, *J* = 2.1 Hz, 1H), 7.01 (d, *J* = 8.4 Hz, 1H), 4.20 (s, 2H), 3.97 (s, 3H), 3.96 (s, 3H). $^{13}C\{1H\}$ NMR (101 MHz, $CDCl_3$) δ 192.8, 151.2, 149.4, 146.7, 139.9, 135.6, 131.2, 131.0, 131.0, 128.2, 128.1, 127.9, 124.9, 113.9, 111.6, 77.4, 56.2, 56.1, 31.5. ^{19}F NMR (376 MHz, $CDCl_3$) δ -62.1.

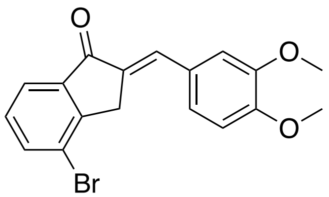

4-bromo-2-(3,4-dimethoxybenzylidene)-1-indanone (3e): Prepared according to General Procedure A from 4-bromo-1-indanone (366 mg, 1.73 mmol, 1.0 eq.) and 3,4-dimethoxybenzaldehyde (288 mg, 1.73 mmol, 1.0 eq.). Collection of the precipitated solids by vacuum filtration followed by air drying for 1 hour afforded the analytically pure title compound as a yellow solid (526 mg, 85% yield). HRMS (ESI) m/z: $[M+H]^+$ calculated for $C_{18}H_{16}BrO_3$ 359.0278, found 359.0281. ^1H NMR (400 MHz, $CDCl_3$) δ 7.86 (dd, *J* = 7.5, 1.0 Hz, 1H), 7.77 (dd, *J* = 7.8, 1.0 Hz, 1H), 7.67 (t, *J* = 2.1 Hz, 1H), 7.38 – 7.31 (m, 2H), 7.20 (d, *J* = 2.1 Hz, 1H), 6.99 (d, *J* = 8.4 Hz, 1H), 3.97 (s, 3H), 3.96 (s, 3H), 3.94 (d, *J* = 2.1 Hz, 2H). $^{13}C\{1H\}$ NMR (101 MHz, $CDCl_3$) δ 193.6, 151.1, 149.4, 149.3, 140.5, 137.1, 135.3, 131.8, 129.6, 128.2, 124.8, 123.3, 121.8, 114.0, 111.6, 56.2, 56.1, 33.7.

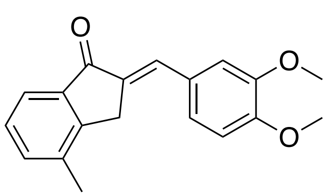

2-(3,4-dimethoxybenzylidene)-4-methyl-1-indanone (3f): Prepared according to General Procedure A from 4-methyl-1-indanone (530 mg, 3.63 mmol, 1.0 eq.) and 3,4-dimethoxybenzaldehyde (602 mg, 3.63 mmol, 1.0 eq.). Collection of the precipitated solids by vacuum filtration followed by air drying for 1 hour afforded the analytically pure title compound as a yellow solid (1.02 g, 95% yield). HRMS (ESI) m/z: $[M+H]^+$ calculated for $C_{19}H_{19}O_3$ 295.1329, found 295.1351. ^{1}H NMR (400 MHz, $CDCl_3$) δ 7.76 (d, *J* = 7.5 Hz, 1H), 7.65 (d, *J* = 2.2 Hz, 1H), 7.43 (d, *J* = 7.3 Hz, 1H), 7.38 – 7.33 (m, 2H), 7.21 (d, *J* = 2.0 Hz, 1H), 6.99 (d, *J* = 8.4 Hz, 1H), 3.97 (s, 3H), 3.96 (s, 3H), 3.90 (d, *J* = 2.0 Hz, 2H), 2.45 (s, 3H). ^{13}C{1H} NMR (101 MHz, $CDCl_3$) δ 194.7, 150.7, 149.3, 148.5, 138.2, 135.3, 135.1, 134.1, 133.0, 128.7, 128.0, 124.4, 121.9, 114.1, 111.5, 56.2, 31.3, 18.1.

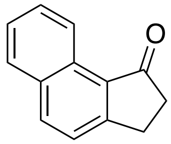

2,3-Dihydro-1*H*-cyclopenta[*a*]naphthlaen-1-one (SI-1): $AlCl_3$ (6.5 g, 47.0 mmol, 1.2 eq.) was charged to a flame-dried 250 mL roundbottom flask equipped with a magnetic stir bar and placed under N_2. The solids were suspended in CH_2Cl_2 (65 mL) with rapid stirring and the flask was cooled to 0°C on an ice-water bath. The septum was pierced with an 18 ga vent needle, and 3-chloropropionyl chloride (3.8 mL, 39.0 mmol, 1.0 eq.) was added to the suspension dropwise *via* syringe. After stirring for 5 minutes, naphthalene (5.0 g, 39.0 mmol, 1.0 eq.) was added as a solid in small portions against a positive pressure of N_2. The reaction mixture was stirred overnight, allowing the ice bath to expire. The CH_2Cl_2 was then removed *in vacuo*, and 30 mL concentrated H_2SO_4 was added to the resulting residue. The mixture was then heated to 90°C for 90 minutes. The resulting thick slurry was then removed from heat and allowed to cool to room temperature. The mixture was then partitioned between EtOAc (125 mL) and H_2O (125 mL) and the organic layer separated. The aqueous layer was then extracted twice more with EtOAc (125 mL), and the combined organic layers were dried over $MgSO_4$ and concentrated *in vacuo*. The residue was them purified by flash chromatography on SiO_2 (gradient elution, 0 to 10% EtOAc in hexane) to give the title compound as a light brown solid (4.04 g, 57% yield). Spectral data matched those previously reported.[3]

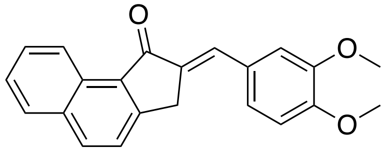

2-(3,4-dimethoxybenzylidene)-2,3-dihydro-1*H*-cyclopenta[*a*]naphthlaen-1-one (3g): Prepared according to General Procedure A from **SI-1** (558 mg, 2.96 mmol, 1.0 eq.) and 3,4-dimethoxybenzaldehyde (500 mg, 2.96 mmol, 1.0 eq.). Collection of the precipitated solids by vacuum filtration followed by air drying for 1 hour afforded the analytically pure title compound as a yellow solid (644 mg, 66% yield). HRMS (ESI) m/z: $[M+H]^+$ calculated for $C_{22}H_{19}O_3$ 331.1329, found 331.1348. ^{1}H NMR (400 MHz, $CDCl_3$) δ 9.35 (dp, *J* = 8.3, 0.7 Hz, 1H), 8.07 (d, *J* = 8.3 Hz, 1H), 7.91 (dd, *J* = 8.3, 1.2 Hz, 1H), 7.71 (ddd, *J* = 8.3, 6.9, 1.3 Hz, 1H), 7.65 – 7.55 (m, 3H), 7.34 (dd, *J* = 8.4, 2.0 Hz, 1H), 7.23 (d, *J* = 2.0 Hz, 1H), 6.97 (d, *J* = 8.4 Hz, 1H), 4.11 (d, *J* = 2.0 Hz, 2H), 3.99 (s, 3H), 3.95 (s, 3H). ^{13}C{1H} NMR (101 MHz, $CDCl_3$) δ 194.9, 152.1, 150.6, 149.3, 135.7, 133.4, 133.1, 133.0, 132.7, 130.0, 129.0, 128.8, 128.4, 126.9, 124.7, 124.6, 123.6, 113.6, 111.5, 56.2, 56.1, 32.9.

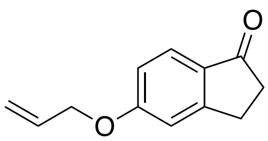

5-allyloxy-1-indanone (SI-2): In a roundbottom flask equipped with a magnetic stir bar, 5-hydroxy-1-indanone (778 mg, 5.25 mmol, 1.0 eq.) and K_2CO_3 (1.04 g, 7.5 mmol, 1.4 eq.) were suspended in acetone (15 mL). Allyl bromide (0.60 mL, 7.0 mmol, 1.3 eq.) was then added *via* syringe, and the reaction mixture was heated to reflux until TLC indicated consumption of the starting material. The reaction mixture was then allowed to cool to room temperature and the solids filtered off, rinsing the filter cake with acetone. The filtrate was concentrated *in vacuo* to give the analytically pure title compound as an off-white solid (950 mg, 96% yield). Spectral data matched those previously reported.[4]

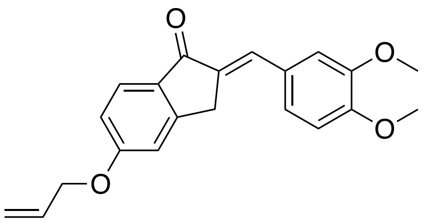

5-allyloxy-2-(3,4-dimethoxybenzylidene)-1-indanone (3h): Prepared according to General Procedure A from **SI-2** (574 mg, 3.05 mmol, 1.0 eq.) and 3,4-dimethoxybenzaldehyde (507 mg, 3.05 mmol, 1.0 eq.). Collection of the precipitated solids by vacuum filtration followed by air drying for 1 hour afforded the analytically pure title compound as a yellow solid (910 mg, 89% yield). HRMS (ESI) m/z: $[M+H]^+$ calculated for $C_{21}H_{21}O_4$ 337.1453, found 337.1447. ^{1}H NMR (400 MHz, $CDCl_3$) δ 7.85 (d, *J* = 8.4 Hz, 1H), 7.57 (t, *J* = 2.1 Hz, 1H), 7.32 – 7.27 (m, 2H), 7.18 (d, *J* = 2.1 Hz, 1H), 7.03 – 6.97 (m, 2H), 6.95 (d, *J* = 8.3 Hz, 1H), 6.08 (ddt, *J* = 17.2, 10.5, 5.3 Hz, 1H), 5.45 (ddt, *J* = 17.2, 5.3, 1.6 Hz, 1H), 5.35 (ddt, *J* = 10.5, 5.3, 1.6 Hz, 1H), 4.66 (dt, *J* = 5.3, 1.6 Hz, 2H), 3.98 (d, *J* = 2.0 Hz, 2H), 3.96 (s, 3H), 3.95 (s, 3H). ^{13}C{1H} NMR (101 MHz, $CDCl_3$) δ 192.9, 164.2, 152.3, 150.5, 149.2, 133.3, 133.0, 132.6, 131.9, 128.8, 126.2, 124.5, 118.4, 115.9, 113.6, 111.5, 110.7, 69.3, 56.1, 32.6.

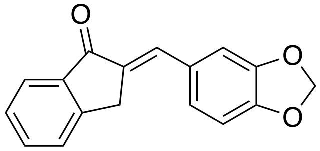

2-(3,4-methylenedioxybenzylidene)-1-indanone (3i): Prepared according to General Procedure A from piperonal (1.00 g, 7.50 mmol, 1.0 eq.) and 3,4-dimethoxybenzaldehyde (1.13 g, 7.50 mmol, 1.0 eq.). Collection of the precipitated solids by vacuum filtration followed by air drying for 1 hour afforded the analytically pure title compound as a yellow solid (1.90 g, 96% yield). Spectral data matched those previously reported.[1]

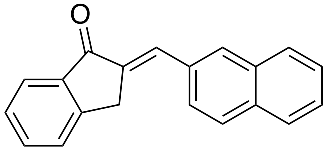

2-(2-naphthylidene)-1-indanone (3j): Prepared according to General Procedure A from 2-naphthaldehyde (1.56 g, 10.0 mmol, 1.0 eq.) and 1-indanone (1.32 g, 10.0 mmol, 1.0 eq.). Collection of the precipitated solids by vacuum filtration followed by air drying for 1 hour afforded the analytically pure title compound as a white solid (2.62 g, 93% yield). HRMS (ESI) m/z: $[M+H]^+$ calculated for $C_{20}H_{15}O$ 271.1118, found 271.1129. ^{1}H NMR (400 MHz, $CDCl_3$) δ 8.17 – 8.12 (m, 1H), 7.98 – 7.87 (m, 3H), 7.89 – 7.83 (m, 2H), 7.80 (dd, *J* = 8.6, 1.8 Hz, 1H), 7.68 – 7.56 (m, 2H), 7.58 – 7.51 (m, 2H), 7.49 – 7.40 (m, 1H), 4.17 (d, *J* = 2.2 Hz, 2H). ^{13}C{1H} NMR (101 MHz, $CDCl_3$) δ 149.8, 138.3, 135.1, 134.8, 134.3, 133.8, 133.5, 133.1, 131.8, 128.8, 128.7, 127.9, 127.5, 127.0, 126.9, 126.3, 124.6, 32.7.

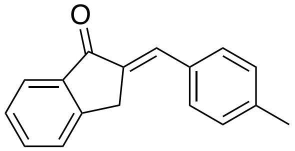

2-(4-methylbenzylidene)-1-indanone (3k): Prepared according to General Procedure A from *p*-tolualdehyde (0.36 mL, 3.06 mmol, 1.0 eq.) and 1-indanone (404 mg, 3.06 mmol, 1.0 eq.). Collection of the precipitated solids by vacuum filtration followed by air drying for 1 hour afforded the analytically pure title compound as a white solid (888 mg, 81% yield). Spectral data matched those previously reported.[5]

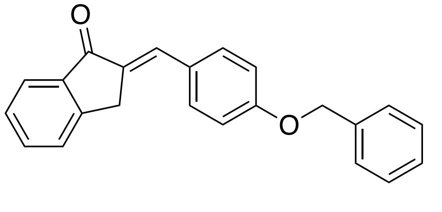

2-(4-benzyloxybenzylidene)-1-indanone (3l): Prepared according to General Procedure A from 4-benzyloxybenzaldehyde (2.12 g, 10.0 mmol, 1.0 eq.) and 1-indanone (1.32 g, 10.0 mmol, 1.0 eq.). Collection of the precipitated solids by vacuum filtration followed by air drying for 1 hour afforded the analytically pure title compound as a beige solid (3.18 g, 97% yield). HRMS (ESI) m/z: $[M+H]^+$ calculated for $C_{23}H_{19}O_2$ 327.1380, found 327.1395. ^{1}H NMR (400 MHz, $CDCl_3$) δ 7.91 (d, *J* = 7.6 Hz, 1H), 7.67 – 7.62 (m, 3H), 7.60 (dd, *J* = 7.2, 1.3 Hz, 1H), 7.55 (d, *J* = 7.5 Hz, 1H), 7.47 – 7.38 (m, 5H), 7.37 – 7.32 (m, 1H), 7.08 – 7.04 (m, 2H), 5.14 (s, 2H), 4.04 – 4.00 (m, 2H). ^{13}C{1H} NMR (101 MHz, $CDCl_3$) δ 194.5, 160.2, 149.6, 138.4, 136.6, 134.5, 133.9, 132.7, 128.8, 128.6, 128.3, 127.7, 127.6, 126.2, 124.5, 115.5, 70.3, 32.6.

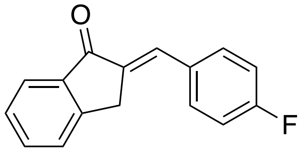

2-(4-fluorobenzylidene)-1-indanone (3m): Prepared according to General Procedure A from 4-fluorobenzaldehyde (0.8 mL, 7.50 mmol, 1.0 eq.) and 1-indanone (1.00 g, 7.50 mmol, 1.0 eq.). Collection of the precipitated solids by vacuum filtration followed by air drying for 1 hour afforded the analytically pure title compound as a beige solid (1.49 g, 84% yield). Spectral data matched those previously reported.[5]

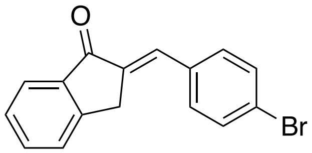

2-(4-bromobenzylidene)-1-indanone (3n): Prepared according to General Procedure A from 4-bromobenzaldehyde (501 mg, 2.71 mmol, 1.0 eq.) and 1-indanone (501 mg, 2.71 mmol, 1.0 eq.). Collection of the precipitated solids by vacuum filtration followed by air drying for 1 hour afforded the analytically pure title compound as a beige solid (693 mg, 86% yield). Spectral data matched those previously reported.[6]

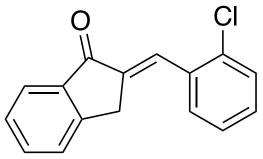

2-(2-chlorobenzylidene)-1-indanone (3o): Prepared according to General Procedure A from *freshly distilled* 2-chlorobenzaldehyde (1.0 mL, 8.97 mmol, 1.0 eq.) and 1-indanone (1.18 g, 8.97 mmol, 1.0 eq.). Collection of the precipitated solids by vacuum filtration followed by air drying for 1 hour afforded the analytically pure title compound as a white solid (2.06 g, 90% yield). Spectral data matched those previously reported.[7]

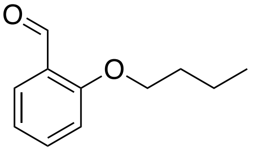

2-butyloxybenzaldehyde (SI-3): K_2CO_3 (3.98 g, 28.8 mmol, 1.5 eq.) was charged to a flame-dried 200 mL roundbottom flask equipped with a magnetic stir bar. The solids were suspended in acetone (60 mL) and placed under N_2. Salicylaldehyde (2.0 mL, 19.2 mmol, 1.0 eq.) was then added in a slow stream *via* syringe, followed by 1-bromobutane (6.2 mL, 57.6 mmol, 3.0 eq.) in a slow stream. The mixture was heated to reflux overnight, after which time it was allowed to cool to room temperature. The solids were filtered off, rinsing the filter cake with acetone, and the filtrate concentrated *in vacuo*. The residue was purified by flash chromatography on SiO_2 (gradient elution, 0 to 10% EtOAc in hexane) to give the title compound as a pale yellow oil (1.66 g, 49% yield). Spectral data matched those previously reported.[8]

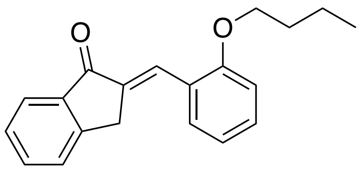

2-(2-butyloxybenzylidene)-1-indanone (3p): Prepared according to General Procedure A from **SI-3** (556 mg, 3.12 mmol, 1.0 eq.) and 1-indanone (412 mg, 3.12 mmol, 1.0 eq.). Collection of the precipitated solids by vacuum filtration followed by air drying for 1 hour afforded the analytically pure title compound as a white solid (655 mg, 72% yield). HRMS (ESI) m/z: $[M+H]^+$calculated for $C_{20}H_{21}O_2$ 293.1563, found 293.1551. ^1H NMR (400 MHz, $CDCl_3$) δ 8.17 (t, *J* = 2.2 Hz, 1H), 7.91 (dt, *J* = 7.7, 1.0 Hz, 1H), 7.68 (dd, *J* = 7.7, 1.7 Hz, 1H), 7.60 (td, *J* = 7.3, 1.2 Hz, 1H), 7.53 (dp, *J* = 7.7, 1.0 Hz, 1H), 7.45 – 7.39 (m, 1H), 7.35 (ddd, *J* = 8.3, 7.4, 1.7 Hz, 1H), 7.02 (td, *J* = 7.6, 1.1 Hz, 1H), 6.95 (dd, *J* = 8.3, 1.1 Hz, 1H), 4.05 (t, *J* = 6.5 Hz, 2H), 4.01 (d, *J* = 2.2 Hz, 2H), 1.85 (ddt, *J* = 8.9, 7.8, 6.4 Hz, 2H), 1.57 – 1.49 (m, 2H), 1.00 (t, *J* = 7.4 Hz, 3H). $^{13}C\{1H\}$ NMR (101 MHz, $CDCl_3$) δ 194.4, 158.9, 149.9, 138.5, 134.6, 134.5, 131.2, 129.8, 128.9, 127.7, 126.2, 124.8, 124.5, 120.4, 112.3, 68.5, 32.6, 31.4, 19.5, 14.0.

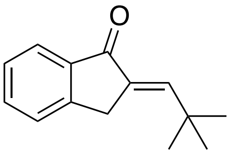

(E)-2-(2,2-dimethylpropenylidene)-1-indanone (3q): Prepared according to General Procedure A from pivaldehyde (2.52 mL, 23.2 mmol, 1.0 eq.) and 1-indanone (3.07 g, 23.2 mmol, 1.0 eq.). Collection of the precipitated solids by vacuum filtration followed by air drying for 1 hour afforded the analytically pure title compound as a white solid (3.03 g, 65% yield). Spectral data matched those previously reported.[9]

---EPOXY KETONES---

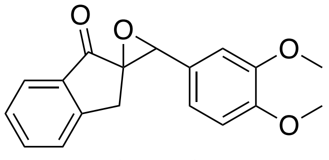

3'-(3,4-dimethoxyphenyl)spiro[2H-indene-2,2'oxiran]-1(3H)-one (4a): Prepared according to General Procedure B from enone **3a** (5.29 g, 18.87 mmol). Collection of the precipitated solids by vacuum filtration after cooling in the refrigerator overnight afforded the analytically pure title compound as a white solid (5.61 g, quantitative yield). HRMS (ESI) m/z: [M+H]+ calculated for $C_{18}H_{17}O_4$ 297.1122, found 297.1141 ^1H NMR (400 MHz, $CDCl_3$) δ 7.86 – 7.83 (m, 1H), 7.63 (td, *J* = 7.5, 1.3 Hz, 1H), 7.45 – 7.40 (m, 2H), 6.90 (d, *J* = 1.5 Hz, 2H), 6.82 (t, *J* = 1.1 Hz, 1H), 4.44 (s, 1H), 3.90 (s, 3H), 3.90 (s, 3H), 3.25 (d, *J* = 18.2 Hz, 1H), 2.97 (d, *J* = 18.1 Hz, 1H). $^{13}C\{1H\}$ NMR (101 MHz, $CDCl_3$) δ 199.7, 151.3, 149.5, 149.3, 136.0, 135.7, 128.1, 127.2, 126.8, 124.2, 119.3, 111.3, 109.3, 67.4, 63.3, 56.1, 29.2.

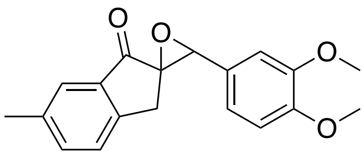

3'-(3,4-dimethoxyphenyl)spiro[6-methyl-2H-indene-2,2'oxiran]-1(3H)-one (4b): Prepared according to General Procedure B from enone **3b** (429 mg, 1.46 mmol). Collection of the precipitated solids by vacuum filtration after cooling in the refrigerator overnight afforded the analytically pure title compound as a white solid (374 mg, 83% yield). HRMS (ESI) m/z: $[M+H]^+$ calculated for $C_{19}H_{19}O_4$ 311.1278, found 311.1302. ^1H NMR (400 MHz, $CDCl_3$) δ 7.65 (dt, *J* = 1.6, 0.8 Hz, 1H), 7.44 (ddd, *J* = 7.8, 1.8, 0.7 Hz, 1H), 7.29 (dd, *J* = 7.8, 0.9 Hz, 1H), 6.89 (d, *J* = 1.4 Hz, 2H), 6.82 (t, *J* = 1.1 Hz, 1H), 4.42 (s, 1H), 3.90 (s, 3H), 3.89 (s, 3H), 3.19 (d, *J* = 18.0 Hz, 1H), 2.92 (d, *J* = 18.0 Hz, 1H), 2.42 (s, 3H). $^{13}C\{1H\}$ NMR (101 MHz, $CDCl_3$) δ 199.8, 149.5, 149.3, 148.6, 138.2, 136.9, 136.1, 127.3, 126.5, 124.2, 119.3, 111.3, 109.3, 77.4, 67.7, 63.2, 56.1, 28.9, 21.3.

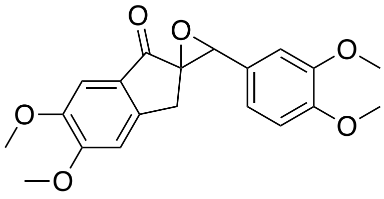

3'-(3,4-dimethoxyphenyl)spiro[5,6-dimethoxy-2H-indene-2,2'oxiran]-1(3H)-one (4c): Prepared according to General Procedure B from enone **3c** (301 mg, 0.88 mmol). Collection of the precipitated solids by vacuum filtration after cooling in the refrigerator overnight afforded the analytically pure title compound as a white solid (264 mg, 84% yield). HRMS (ESI) m/z: $[M+Na]^+$ calculated for $C_{20}H_{20}O_6Na$ 379.1175, found 379.1152. ^1H NMR (400 MHz, $CDCl_3$) δ 7.27 (s, 1H), 6.91 – 6.89 (m, 2H), 6.84 – 6.81 (m, 2H), 4.43 (s, 1H), 3.94 (s, 3H), 3.93 (s, 3H), 3.90 (s, 3H), 3.90 (s, 3H), 3.15 (d, *J* = 17.9 Hz, 1H), 2.88 (d, *J* = 17.9 Hz, 1H). $^{13}C\{1H\}$ NMR (101 MHz, $CDCl_3$) δ 197.9, 156.4, 150.0, 149.4, 149.3, 146.9, 128.9, 127.5, 119.2, 111.3, 109.2, 107.8, 104.8, 67.7, 62.8, 56.5, 56.4, 56.1, 56.1, 29.2.

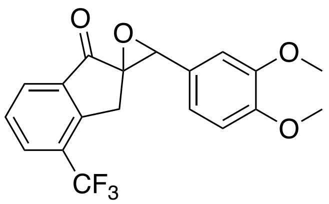

3'-(3,4-dimethoxyphenyl)spiro[4-(trifluoromethyl)-2H-indene-2,2'oxiran]-1(3H)-one (4d): Prepared according to General Procedure B from enone **3d** (680 mg, 1.95 mmol). Collection of the precipitated solids by vacuum filtration after cooling in the refrigerator overnight afforded the analytically pure title compound as a white solid (618 mg, 87% yield). HRMS (ESI) m/z: $[M-H]^-$ calculated for $C_{19}H_{14}F_3O_4$ 363.0849, found 363.0848. 1H NMR (400 MHz, $CDCl_3$) δ 8.03 (d, *J* = 7.7 Hz, 1H), 7.90 (d, *J* = 7.7 Hz, 1H), 7.59 (dt, *J* = 7.7, 1.1 Hz, 1H), 6.91 (d, *J* = 1.1 Hz, 2H), 6.82 (s, 1H), 4.48 (s, 1H), 3.92 (s, 3H), 3.90 (s, 3H), 3.38 (d, *J* = 19.0 Hz, 1H), 3.15 (d, *J* = 19.0 Hz, 1H). $^{13}C\{1H\}$ NMR (101 MHz, $CDCl_3$) δ 198.7, 149.7, 149.4, 148.5, 137.3, 132.3, 132.2, 128.9, 128.6, 127.6, 126.4, 122.3, 119.3, 111.4, 109.3, 66.6, 63.7, 56.1, 28.2. ^{19}F NMR (376 MHz, $CDCl_3$) δ -62.4.

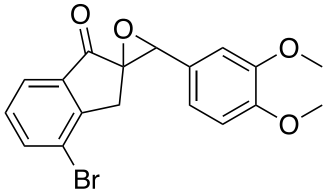

3'-(3,4-dimethoxyphenyl)spiro[4-methyl-2H-indene-2,2'oxiran]-1(3H)-one (4e): Prepared according to General Procedure B from enone **3h** (505 mg, 1.40 mmol). Collection of the precipitated solids by vacuum filtration after cooling in the refrigerator overnight afforded the analytically pure title compound as a white solid (498 mg, 95% yield). HRMS (ESI) m/z: $[M+K]^+$ calculated for $C_{18}H_{15}BrO_4K$ 412.9786, found 412.9809. ^1H NMR (400 MHz, $CDCl_3$) δ 7.83 – 7.77 (m, 2H), 7.38 – 7.32 (m, 1H), 6.92 (d, *J* = 1.1 Hz, 2H), 6.83 (d, *J* = 1.1 Hz, 1H), 4.45 (s, 1H), 3.92 (s, 3H), 3.91 (s, 3H), 3.15 (d, *J* = 18.5 Hz, 1H), 2.91 (d, *J* = 18.7 Hz, 1H). $^{13}C\{1H\}$ NMR (101 MHz, $CDCl_3$) δ 199.4, 151.2, 149.7, 149.4, 138.4, 138.0, 129.8, 126.7, 123.0, 122.3, 119.3, 111.4, 109.3, 67.2, 63.6, 56.2, 56.1, 30.4.

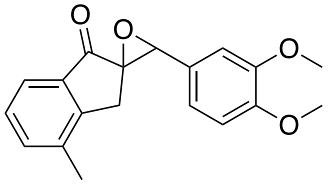

3'-(3,4-dimethoxyphenyl)spiro[4-methyl-2H-indene-2,2'oxiran]-1(3H)-one (4f): Prepared according to General Procedure B from enone **3f** (242 mg, 0.82 mmol). Collection of the precipitated solids by vacuum filtration after cooling in the refrigerator overnight afforded the analytically pure title compound as a white solid (254 mg, quantitative yield). HRMS (ESI) m/z: $[M+Na]^+$ calculated for $C_{19}H_{18}O_4Na$ 333.1097, found 333.1092. ^1H NMR (400 MHz, $CDCl_3$) δ 7.69 (d, *J* = 7.6 Hz, 1H), 7.44 (d, *J* = 7.4 Hz, 1H), 7.35 (t, *J* = 7.5 Hz, 1H), 6.92 – 6.90 (m, 2H), 6.84 (s, 1H), 4.45 (s, 1H), 3.91 (d, *J* = 2.3 Hz, 6H), 3.08 (d, *J* = 18.2 Hz, 1H), 2.83 (d, *J* = 18.1 Hz, 1H), 2.27 (s, 3H). $^{13}C\{1H\}$ NMR (101 MHz, $CDCl_3$) δ 194.8, 150.7, 149.2, 148.5, 138.1, 135.3, 135.1, 134.1, 133.0, 128.7, 128.0, 124.4, 121.9, 114.1, 111.5, 56.1, 31.3, 18.1.

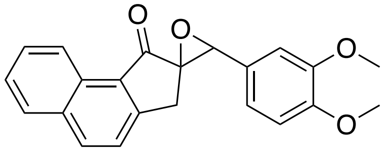

Epoxide 4g: Prepared according to General Procedure B from enone **3g** (641 mg, 1.94 mmol). Collection of the precipitated solids by vacuum filtration after cooling in the refrigerator overnight afforded the analytically pure title compound as a white solid (619 mg, 92% yield). HRMS (ESI) m/z: $[M+H]^+$ calculated for $C_{22}H_{19}O_4$ 347.1278, found 347.1300. ^1H NMR (400 MHz, $CDCl_3$) δ 9.17 – 9.12 (m, 1H), 8.09 (d, *J* = 8.4 Hz, 1H), 7.90 (d, *J* = 8.2 Hz, 1H), 7.72 (ddd, *J* = 8.3, 7.0, 1.3 Hz, 1H), 7.60 (ddd, *J* = 8.2, 7.0, 1.3 Hz, 1H), 7.46 (d, *J* = 8.4 Hz, 1H), 6.94 (dd, *J* = 8.4, 1.8 Hz, 1H), 6.90 (d, *J* = 8.3 Hz, 1H), 6.86 (d, *J* = 1.8 Hz, 1H), 4.50 (s, 1H), 3.91 (d, *J* = 0.7 Hz, 6H), 3.36 (d, *J* = 18.4 Hz, 1H), 3.06 (d, *J* = 18.5 Hz, 1H). $^{13}C\{1H\}$ NMR (101 MHz, $CDCl_3$) δ 194.6, 160.5, 154.7, 149.3, 136.9, 132.9, 129.7, 128.4, 127.3, 124.5, 123.8, 119.2, 111.3, 109.3, 67.6, 63.0, 56.2, 29.9.

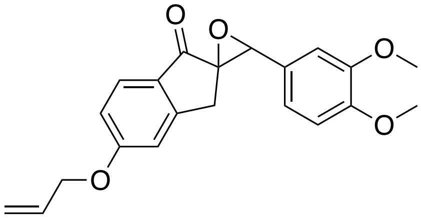

3'-(3,4-dimethoxyphenyl)spiro[5-allyloxy-2H-indene-2,2'oxiran]-1(3H)-one (4h): Prepared according to General Procedure B from enone **3h** (557 mg, 1.66 mmol). Collection of the precipitated solids by vacuum filtration after cooling in the refrigerator overnight afforded the analytically pure title compound as a white solid (448 mg, 77% yield). HRMS (ESI) m/z: $[M+H]^+$ calculated for $C_{21}H_{21}O_5$ 353.1384, found 353.1397. ^1H NMR (400 MHz, $CDCl_3$) δ 7.78 (d, *J* = 8.6 Hz, 1H), 6.96 (dt, *J* = 8.5, 2.5 Hz, 1H), 6.89 (d, *J* = 1.4 Hz, 2H), 6.84 (d, *J* = 2.2 Hz, 1H), 6.02 (ddt, *J* = 17.2, 10.5, 5.2 Hz, 1H), 5.41 (dt, *J* = 17.2, 1.5 Hz, 1H), 5.32 (dt, *J* = 10.5, 1.4 Hz, 1H), 4.60 (dt, *J* = 4.5, 1.5 Hz, 2H), 4.42 (s, 1H), 3.90 (s, 3H), 3.90 (s, 3H), 3.18 (d, *J* = 18.2 Hz, 1H), 2.91 (d, *J* = 18.2 Hz, 1H). $^{13}C\{1H\}$ NMR (101 MHz, $CDCl_3$) δ 197.6, 165.1, 154.0, 149.4, 149.3, 132.2, 129.4, 127.4, 126.2, 119.2, 118.6, 116.5, 111.3, 111.1, 109.3, 69.3, 67.6, 62.9, 56.1, 29.5.

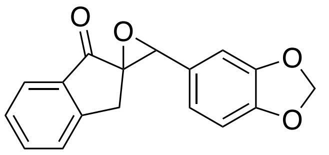

3'-(3,4-methylenedioxyphenyl)spiro[2H-indene-2,2'oxiran]-1(3H)-one (4i): Prepared according to General Procedure B from enone **3i** (1.87 g, 7.08 mmol). Collection of the precipitated solids by vacuum filtration after cooling in the refrigerator overnight afforded the analytically pure title compound as a white solid (1.96 g, 99% yield). HRMS (ESI) m/z: $[M+H]^+$ calculated for $C_{17}H_{13}O_4$ 281.0809, found 281.0818. ^1H NMR (400 MHz, $CDCl_3$) δ 7.87 – 7.82 (m, 1H), 7.63 (tt, *J* = 7.5, 2.0 Hz, 1H), 7.46 – 7.40 (m, 2H), 6.83 (t, *J* = 0.9 Hz, 2H), 6.80 – 6.77 (m, 1H), 6.00 (p, *J* = 1.7 Hz, 2H), 4.40 (s, 1H), 3.25 (d, *J* = 18.2 Hz, 1H), 2.99 (d, *J* = 18.2 Hz, 1H). $^{13}C\{1H\}$ NMR δ 199.7, 151.3, 148.1, 136.0, 135.8, 128.6, 128.1, 126.8, 124.3, 120.7, 108.6, 106.7, 101.5, 67.3, 63.3, 29.2.

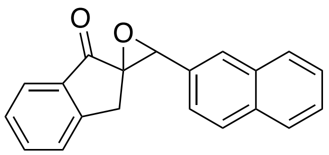

3'-(2-naphthyl)spiro[2H-indene-2,2'oxiran]-1(3H)-one (4j): Prepared according to General Procedure B from enone **3j** (2.17 g, 8.05 mmol). Collection of the precipitated solids by vacuum filtration after cooling in the refrigerator overnight afforded the analytically pure title compound as a white solid (1.91g, 83% yield). HRMS (ESI) m/z: [M+H]+ calculated for $[C_{20}H_{14}O_2+H]^+$ 287.1067, found 287.1082. ^1H NMR (400 MHz, $CDCl_3$) δ 7.90 – 7.81 (m, 6H), 7.61 (dt, *J* = 7.5, 1.3 Hz, 1H), 7.54 – 7.50 (m, 2H), 7.46 – 7.40 (m, 2H), 7.36 (dt, *J* = 7.7, 0.9 Hz, 1H), 4.65 (s, 1H), 3.31 (d, *J* = 18.2 Hz, 1H), 2.95 (d, *J* = 18.2 Hz, 1H).

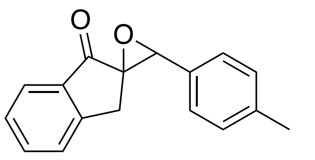

3’-(4-methylphenyl)spiro[2H-indene-2,2’oxiran]-1(3H)-one (4k): Prepared according to General Procedure B from enone **3k** (419 mg, 1.43 mmol). Collection of the precipitated solids by vacuum filtration after cooling in the refrigerator overnight afforded the analytically pure title compound as a white solid (279 mg, 63% yield). Spectral data matched those previously reported.[10]

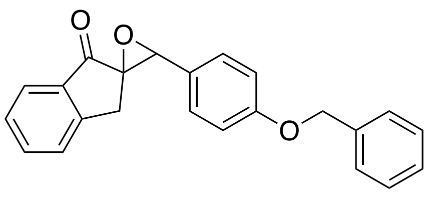

3’-(4-benzyloxyphenyl)spiro[2H-indene-2,2’oxiran]-1(3H)-one (4l): Prepared according to General Procedure B from enone **3l** (2.49 g, 7.63 mmol). Collection of the precipitated solids by vacuum filtration after cooling in the refrigerator overnight afforded the analytically pure title compound as a white solid (2.19 g, 84% yield). HRMS (ESI) m/z: $[M+Na]^+$ calculated for $C_{23}H_{18}O_3Na$ 365.1148, found 365.1169. ^1H NMR (400 MHz, $CDCl_3$) δ 7.87 – 7.81 (m, 1H), 7.65 – 7.57 (m, 1H), 7.47 – 7.32 (m, 7H), 7.24 (d, *J* = 2.1 Hz, 1H), 7.03 – 6.98 (m, 2H), 5.09 (s, 2H), 4.43 (s, 1H), 3.25 (d, *J* = 18.2 Hz, 1H), 2.97 (dd, *J* = 18.1, 0.9 Hz, 1H). $^{13}C\{1H\}$ NMR (101 MHz, $CDCl_3$) δ 199.8, 159.3, 151.3, 136.9, 136.0, 135.7, 128.8, 128.2, 128.1, 128.0, 127.6, 127.0, 126.8, 124.2, 115.1, 70.3, 67.4, 63.2, 29.2.

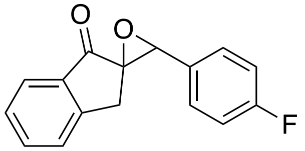

3’-(4-fluorophenyl)spiro[2H-indene-2,2’oxiran]-1(3H)-one (4m): Prepared according to General Procedure B from enone **3m** (1.64 g, 6.08 mmol). Collection of the precipitated solids by vacuum filtration after cooling in the refrigerator overnight afforded the analytically pure title compound as a white solid (1.49 g, 85% yield). Spectral data matched those previously reported.[6]

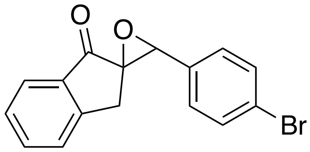

3’-(4-bromophenyl)spiro[2H-indene-2,2’oxiran]-1(3H)-one (4n): Prepared according to General Procedure B from enone **3n** (637 mg, 2.13 mmol). Collection of the precipitated solids by vacuum filtration after cooling in the refrigerator overnight afforded the analytically pure title compound as a white solid (605 mg, 90% yield). Spectral data matched those previously reported.[6]

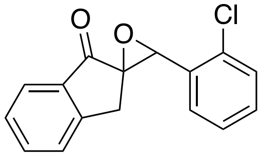

3’-(2-chlorophenyl)spiro[2H-indene-2,2’oxiran]-1(3H)-one (4o): Prepared according to General Procedure B from enone **3o** (309 mg, 1.21 mmol). Collection of the precipitated solids by vacuum filtration after cooling in the refrigerator overnight afforded the analytically pure title compound as a white solid (299 mg, 91% yield). Spectral data matched those previously reported.[6]

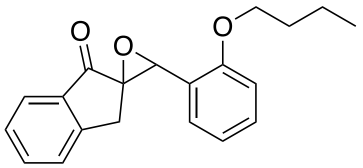

3’-(2-butyloxyphenyl)spiro[2H-indene-2,2’oxiran]-1(3H)-one (4p): Prepared according to General Procedure B from enone **3p** (419 mg, 1.43 mmol). Collection of the precipitated solids by vacuum filtration after cooling in the refrigerator overnight afforded the analytically pure title compound as a white solid (279 mg, 63% yield). HRMS (ESI) m/z: $[M+Na]^+$ calculated for $C_{20}H_{20}O_3Na$ 331.1304, found 331.1321.^1H NMR (400 MHz, $CDCl_3$) δ 7.86 (d, *J* = 7.7 Hz, 1H), 7.61 (td, *J* = 7.5, 1.3 Hz, 1H), 7.45 – 7.26 (m, 5H), 6.99 (td, *J* = 7.5, 1.0 Hz, 1H), 6.88 – 6.84 (m, 1H), 4.66 (s, 1H), 3.95 – 3.83 (m, 2H), 3.13 (d, *J* = 18.2 Hz, 1H), 2.82 (d, *J* = 18.0 Hz, 1H), 1.52 (ddd, *J* = 13.6, 7.5, 6.2 Hz, 2H), 1.17 (q, *J* = 7.4 Hz, 2H), 0.71 (t, *J* = 7.4 Hz, 3H). $^{13}C\{1H\}$ NMR (101 MHz, $CDCl_3$) δ 200.1, 157.7, 151.3, 136.1, 135.5, 129.5, 128.0, 126.8, 126.7, 124.2, 123.8, 120.5, 111.2, 67.9, 66.7, 60.3, 31.1, 29.4, 19.2, 13.7.

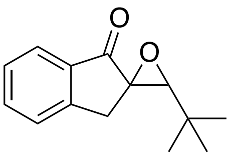

3’-*tert*-butylspiro[2H-indene-2,2’oxiran]-1(3H)-one (4q): Prepared according to General Procedure B from enone **3r** (3.02 g, 15.05 mmol). Collection of the precipitated solids by vacuum filtration after cooling in the refrigerator overnight afforded the analytically pure title compound as a white solid (2.39 g, 76% yield). HRMS (ESI) m/z: $[M+H]^+$ calculated for $[C_{14}H_{16}O_2+H]^+$ 217.1223, found 217.1218. ^1H NMR (400 MHz, $CDCl_3$) δ 7.83 (m, 1H), 7.67 (dt, *J* = 7.5, 1.3 Hz, 1H), 7.52 (m, 1H), 7.46 – 7.40 (m, 1H), 3.58 (d, *J* = 18.4 Hz , 1H), 3.33 (d, J = 1.84 Hz, 1H), 3.32 (s, 1H), 1.09 (s, 9H). $^{13}C\{1H\}$ NMR (101 MHz, $CDCl_3$) δ 201.9, 151.9, 135.7, 135.5, 128.1, 126.7, 124.2, 70.0, 63.4, 32.1, 30.7, 26.9.

---EPOXY ALCOHOLS---

NOTE: The epoxy alcohols were synthesized and used as inconsequential mixtures of diastereomers. Characterization data are provided for these mixtures as they were used in the subsequent reaction. In some cases, the epoxy alcohols were found to decompose upon purification or upon standing. Such cases are noted as appropriate.

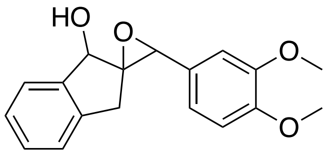

3'-(3,4-dimethoxyphenyl)-1,3-dihydrospiro[indene-2,2'-oxiran]-1-ol (1a): Prepared according to General Procedure C from epoxide **4a** (4.50 g, 15.2 mmol). The title compound was recovered in sufficiently pure form directly after aqueous extraction as a white solid (4.11 g, 91% yield, single diastereomer). HRMS (ESI) m/z: $[M+Cl]^-$ calculated for $C_{18}H_{18}O_4Cl$ 333.0899, found 333.0886. ^1H NMR (400 MHz, $CDCl_3$) δ 7.46 – 7.42 (m, 1H), 7.32 – 7.26 (m, 2H), 7.13 – 7.09 (m, 1H), 6.87 (d, *J* = 1.6 Hz, 2H), 6.81 (d, *J* = 1.5 Hz, 1H), 5.28 (s, 1H), 4.53 (s, 1H), 3.89 (s, 3H), 3.88 (s, 3H), 3.04 (d, *J* = 17.3 Hz, 1H), 2.83 (d, *J* = 17.3 Hz, 1H). $^{13}C\{1H\}$ NMR (101 MHz, $CDCl_3$) δ 149.1, 148.9, 141.7, 139.2, 129.0, 128.2, 127.5, 125.0, 124.4, 118.9, 111.2, 109.5, 77.7, 74.8, 59.9, 56.1, 56.1, 32.3.

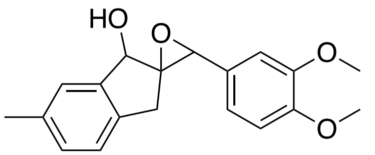

3'-(3,4-dimethoxyphenyl)-6-methyl-1,3-dihydrospiro[indene-2,2'-oxiran]-1-ol (1b): Prepared according to General Procedure C from epoxide **4b** (356 mg, 1.15 mmol). The title compound was recovered in sufficiently pure form directly after aqueous extraction as an orange solid (251 mg, 70% yield, 1.5:1 d.r.). HRMS (ESI) m/z: [M+H]+ calculated for $C_{19}H_{21}O_4$ 313.1435, found 313.1450. ^1H NMR (400 MHz, $CDCl_3$, mixture of diastereomers) δ 7.10 – 7.06 (m, 2H), 7.00 (d, *J* = 7.7 Hz, 1H), 6.92 – 6.89 (m, 1H), 6.87 – 6.85 (m, 2H), 6.80 (d, *J* = 1.6 Hz, 1H), 5.25 (d, *J* = 7.6 Hz, 1H), 5.11 (d, *J* = 7.3 Hz, 1H), 4.51 (s, 1H), 4.24 (s, 1H), 3.91 (s, 1H), 3.90 (s, 2H), 3.89 (s, 3H), 3.88 (s, 3H), 2.97 (d, *J* = 6.1 Hz, 1H), 2.78 (d, *J* = 17.3 Hz, 1H), 2.51 (d, *J* = 7.4 Hz, 1H), 2.37 (s, 3H), 2.36 (s, 3H), 2.10 (d, *J* = 7.7 Hz, 1H). $^{13}C\{1H\}$ NMR (101 MHz, $CDCl_3$) δ 149.0, 148.8, 141.7, 137.3, 136.1, 129.9, 129.8, 129.5, 128.3, 125.6, 125.2, 124.9, 124.8, 124.8, 120.0, 118.9, 118.8, 111.3, 111.1, 111.0, 109.5, 109.2, 84.1, 77.6, 77.5, 77.2, 76.8, 75.0, 74.0, 72.5, 67.2, 61.7, 59.9, 56.1, 56.1, 56.1, 40.0, 32.9, 31.9, 21.5, 21.2.

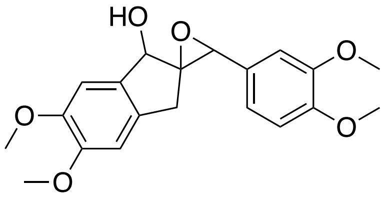

3'-(3,4-dimethoxyphenyl)-5,6-dimethoxy-1,3-dihydrospiro[indene-2,2'-oxiran]-1-ol (1c): Prepared according to General Procedure C from epoxide **4c** (246 mg, 0.69 mmol). The title compound was recovered in sufficiently pure form directly after aqueous extraction as a white solid (230 mg, 93% yield, appx. 1:1 d.r.). HRMS (ESI) m/z: [M+H]+ calculated for $C_{20}H_{23}O_6$ 359.1489, found 359.1504. ^1H NMR (400 MHz, $CDCl_3$, mixture of diastereomers) δ 7.08 – 7.00 (m, 1H), 6.96 (d, *J* = 0.8 Hz, 1H), 6.93 – 6.89 (m, 1H), 6.87 (d, *J* = 0.8 Hz, 2H), 6.81 (s, 1H), 6.63 (s, 1H), 5.21 (d, *J* = 8.0 Hz, 1H), 4.50 (s, 1H), 3.99 (d, *J* = 13.2 Hz, 1H), 3.94 (d, *J* = 8.5 Hz, 1H), 3.92 (d, *J* = 3.4 Hz, 2H), 3.90 (s, 3H), 3.89 (s, 3H), 3.89 (s, 3H), 3.87 – 3.85 (m, 3H), 3.82 (s, 3H), 3.31 (s, 1H), 2.96 (d, *J* = 17.2 Hz, 1H), 2.78 (d, *J* = 17.4 Hz, 1H), 2.07 (d, *J* = 8.1 Hz, 1H). $^{13}C\{1H\}$ NMR (101 MHz, $CDCl_3$, mixture of diastereomers) δ 150.3, 149.2, 149.1, 148.9, 140.9, 133.3, 131.4, 128.4, 118.9, 111.2, 110.6, 109.4, 109.2, 107.6, 107.1, 75.0, 60.0, 56.2, 56.1, 56.1, 56.1, 33.2, 32.4.

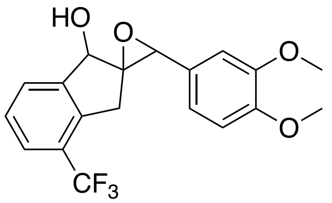

3'-(3,4-dimethoxyphenyl)-4-trifluoromethyl-1,3-dihydrospiro[indene-2,2'-oxiran]-1-ol (1d): Prepared according to General Procedure C from epoxide **4d** (549 mg, 1.51 mmol). The title compound was recovered in sufficiently pure form directly after aqueous extraction as a white solid (295 mg, 53% yield, appx. 5:1 d.r.). HRMS (ESI) m/z: [M+H]+ calculated for $C_{19}H_{17}F_3O_4$ 366.1079, found 366.1075. ^1H NMR (400 MHz, $CDCl_3$) δ 7.72 (d, *J* = 8.3 Hz, 0H), 7.66 – 7.47 (m, 4H), 7.45 – 7.29 (m, 2H), 7.13 – 7.00 (m, 1H), 7.00 – 6.77 (m, 6H), 5.26 (d, *J* = 7.6 Hz, 2H), 5.21 – 5.07 (m, 1H), 4.53 (s, 1H), 4.04 (d, *J* = 10.9 Hz, 1H), 4.00 – 3.92 (m, 1H), 3.90 (s, 4H), 3.88 (s, 5H), 3.83 (s, 1H), 3.37 – 3.28 (m, 1H), 3.25 – 2.92 (m, 5H), . $^{13}C\{1H\}$ NMR (101 MHz, $CDCl_3$) δ 205.0, 149.1, 149.0, 144.1, 143.5, 137.3, 131.3, 128.2, 127.9, 127.5, 127.4, 127.4, 126.1, 122.3, 119.8, 118.9, 113.1, 111.5, 111.2, 111.1, 109.4, 78.7, 78.3, 77.2, 74.6, 74.0, 60.1, 59.4, 56.1, 56.0, 31.4. ^{19}F NMR (376 MHz, $CDCl_3$) δ -62.31 (minor), -62.43 (major).

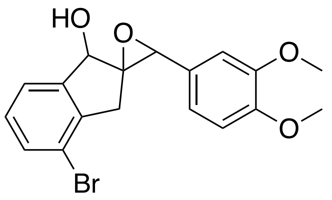

4-bromo-3'-(3,4-dimethoxyphenyl) -1,3-dihydrospiro[indene-2,2'-oxiran]-1-ol (1e): Prepared according to General Procedure C from epoxide **4e** (567 mg, 1.50 mmol). The title compound was recovered in sufficiently pure form directly after aqueous extraction as a white solid (430 mg, 75% yield, single diastereomer). **1e was found to be moderately unstable under ambient conditions. Analytical data and spectra reflect 1e as it was used in the subsequent reaction, which proceeded as normal.** HRMS (ESI) m/z: [M+Na]+ calculated for $C_{18}H_{17}BrO_4Na$ 399.0202, found 399.0196. ^1H NMR (400 MHz, $CDCl_3$) δ 7.63 (t, *J* = 4.1 Hz, 2H), 7.56 – 7.53 (m, 1H), 7.29 (s, 1H), 7.10 (dd, *J* = 8.2, 2.0 Hz, 1H), 7.05 (d, *J* = 2.0 Hz, 1H), 7.01 (d, *J* = 8.2 Hz, 1H), 5.28 (s, 1H), 3.95 (s, 3H), 3.93 (s, 3H), 2.48 (d, *J* = 1.0 Hz, 3H). $^{13}C\{1H\}$ NMR (101 MHz, $CDCl_3$) δ 149.1, 148.9, 143.5, 139.9, 132.1, 129.3, 128.7, 127.6, 126.8, 123.4, 122.3, 120.2, 119.0, 113.2, 111.2, 109.4, 78.6, 75.3, 73.6, 60.6, 60.0, 56.1, 56.1, 56.0, 44.3, 33.8, 21.2.

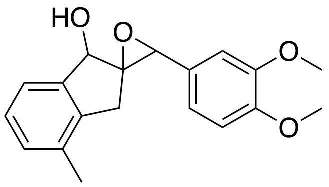

3'-(3,4-dimethoxyphenyl)-4-methyl-1,3-dihydrospiro[indene-2,2'-oxiran]-1-ol (1f): Prepared according to General Procedure C from epoxide **4f** (254 mg, 0.82 mmol). The title compound was recovered in sufficiently pure form directly after aqueous extraction as a white solid (136 mg, 53% yield, mixture of diastereomers). **1f was found to be moderately unstable under ambient conditions. Analytical data reflect 1f as it was used in the subsequent reaction, which proceeded as normal.** HRMS (ESI) m/z: [M+Cl]- calculated for $C_{19}H_{20}O_4Cl$ 347.1056, found 347.1053. ^{1}H NMR (400 MHz, $CDCl_3$) δ 7.28 (d, *J* = 7.8 Hz, 1H), 7.25 – 7.19 (m, 2H), 7.10 (d, *J* = 7.3 Hz, 1H), 6.88 (d, *J* = 1.4 Hz, 2H), 6.83 (s, 1H), 5.30 (s, 1H), 5.25 (d, *J* = 7.9 Hz, 1H), 4.53 (s, 1H), 3.92 – 3.91 (m, 1H), 3.90 (s, 3H), 3.89 (s, 3H), 2.89 (d, *J* = 17.7 Hz, 1H), 2.75 (d, *J* = 17.7 Hz, 1H), 2.14 (s, 3H), 2.14 (s, 3H), 2.05 (d, *J* = 7.9 Hz, 1H). ^{13}C NMR (101 MHz, $CDCl_3$) δ 150.7, 149.2, 148.8, 148.5, 143.1, 142.9, 140.1, 138.1, 135.3, 135.1, 134.2, 134.1, 133.0, 130.5, 129.6, 128.6, 128.0, 127.6, 126.3, 124.4, 122.3, 121.9, 121.3, 114.0, 112.3, 111.4, 111.2, 78.9, 56.1, 56.0, 34.5, 31.3, 18.9, 18.1.

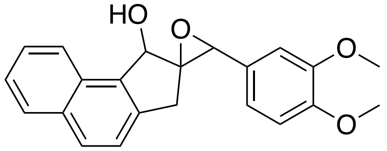

3'-(3,4-dimethoxyphenyl)-1,3-dihydrospiro[cyclopenta[*a*]naphthalene-2,2'-oxiran]-1-ol (1g): Prepared according to General Procedure C from epoxide **4g** (607 mg, 1.75 mmol). The title compound was recovered in sufficiently pure form directly after aqueous extraction as an orange-brown solid (220 mg, 36% yield, 1:1 d.r.). HRMS (ESI) m/z: [M+NH4]+ calculated for $C_{22}H_{20}O_4NH_4$ 366.1700, found 366.1700. ^{1}H NMR (400 MHz, $CDCl_3$, mixture of diastereomers) δ 9.12 (dd, *J* = 8.3, 1.2 Hz, 1H), 8.24 (ddd, *J* = 27.1, 8.3, 1.2 Hz, 1H), 8.06 (d, *J* = 8.4 Hz, 1H), 7.89 – 7.81 (m, 3H), 7.78 – 7.75 (m, 1H), 7.70 (ddd, *J* = 8.4, 7.0, 1.3 Hz, 1H), 7.60 – 7.57 (m, 1H), 7.54 (ddd, *J* = 8.4, 3.1, 1.4 Hz, 1H), 7.52 – 7.44 (m, 2H), 7.43 (d, *J* = 8.4 Hz, 1H), 7.29 – 7.25 (m, 1H), 6.95 – 6.79 (m, 7H), 5.61 – 5.55 (m, 1H), 5.28 (s, 1H), 4.59 (s, 1H), 3.90 (s, 2H), 3.90 (s, 2H), 3.89 (s, 3H), 3.89 (s, 5H), 3.34 – 3.23 (m, 2H), 3.08 – 3.01 (m, 2H). $^{13}C\{^{1}H\}$ NMR (101 MHz, $CDCl_3$) δ 154.6, 149.5, 149.3, 136.9, 132.8, 130.2, 129.9, 129.7, 128.6, 128.4, 127.5, 127.3, 127.0, 125.8, 124.5, 124.5, 124.2, 123.8, 123.0, 119.2, 118.8, 111.3, 109.4, 109.3, 109.2, 79.3, 74.8, 74.2, 67.5, 63.0, 56.1, 34.2, 34.1, 29.9.

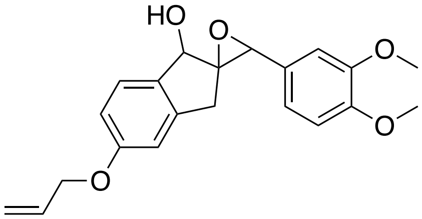

5-allyloxy-3'-(3,4-dimethoxyphenyl)-1,3-dihydrospiro[indene-2,2'-oxiran]-1-ol (1h): Prepared according to General Procedure C from epoxide **4h** (440 mg, 1.25 mmol). The title compound was recovered in sufficiently pure form directly after aqueous extraction as a bright yellow solid (310 mg, 70% yield, 1:1 d.r.). HRMS (ESI) m/z: [M+H]+ calculated for $C_{21}H_{23}O_5$ 355.1540, found 355.1536. ^{1}H NMR (400 MHz, $CDCl_3$, mixture of diastereomers) δ 7.37 (dd, *J* = 25.5, 8.4 Hz, 2H), 6.93 – 6.80 (m, 8H), 6.74 – 6.64 (m, 2H), 6.02 (ddq, *J* = 17.3, 10.4, 5.2 Hz, 2H), 5.42 – 5.34 (m, 2H), 5.27 (ddd, *J* = 10.6, 2.3, 1.4 Hz, 2H), 5.13 (dd, *J* = 44.1, 7.5 Hz, 2H), 4.53 – 4.48 (m, 5H), 3.96 (d, *J* = 7.0 Hz, 1H), 3.91 (s, 2H), 3.90 (s, 3H), 3.89 (s, 3H), 3.89 (s, 3H), 3.06 – 2.78 (m, 4H), 2.45 (d, *J* = 7.1 Hz, 1H), 1.98 (d, *J* = 7.9 Hz, 1H). $^{13}C\{^{1}H\}$ NMR (101 MHz, $CDCl_3$) δ 149.0, 133.4, 125.6, 120.1, 114.1, 111.5, 111.0, 82.5, 78.9, 69.1, 56.1.

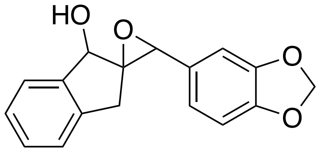

3'-(3,4-methylenedioxyphenyl)-1,3-dihydrospiro[indene-2,2'-oxiran]-1-ol (1i): Prepared according to General Procedure C from epoxide **4i** (503 mg, 1.79 mmol). The title compound was recovered in sufficiently pure form directly after aqueous extraction as a bright yellow solid (469 mg, 93% yield, 2:1 d.r.). HRMS (ESI) m/z: [M-H]- calculated for $C_{17}H_{13}O_4$ 281.0807, found 281.0807. ^{1}H NMR (400 MHz, $CDCl_3$, mixture of diastereomers) δ 7.52 – 7.48 (m, 0H), 7.45 – 7.41 (m, 1H), 7.40 (d, *J* = 5.4 Hz, 0H), 7.40 – 7.26 (m, 3H), 7.25 – 7.17 (m, 1H), 7.13 – 7.10 (m, 0H), 6.89 – 6.74 (m, 5H), 5.99 – 5.95 (m, 4H), 5.25 (d, *J* = 7.6 Hz, 1H), 5.14 (d, *J* = 7.4 Hz, 0H), 4.48 (s, 1H), 4.21 (s, 0H), 3.03 (ddd, *J* = 17.5, 4.2, 1.0 Hz, 2H), 2.99 – 2.86 (m, 1H), 2.83 (dd, *J* = 17.6, 0.9 Hz, 1H), 2.56 (d, *J* = 7.7 Hz, 0H), 2.19 (d, *J* = 7.9 Hz, 1H). $^{13}C\{^{1}H\}$ NMR (101 MHz, $CDCl_3$) δ 147.8, 147.8, 147.6, 147.4, 142.1, 141.7, 139.2, 139.1, 129.6, 129.2, 129.0, 128.9, 128.6, 127.5, 127.2, 125.3, 125.0, 124.7, 124.4, 121.2, 120.8, 120.2, 108.3, 108.2, 108.1, 106.8, 101.3, 101.2, 101.2, 83.9, 79.3, 79.0, 77.6, 74.8, 60.0, 40.5, 40.3, 32.2.

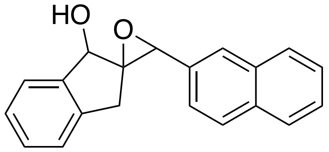

3'-(2-naphthyl)-1,3-dihydrospiro[indene-2,2'-oxiran]-1-ol (1j): Prepared according to General Procedure C from epoxide **4j** (1.05 g, 3.67 mmol). The title compound was recovered in sufficiently pure form directly after aqueous extraction as a brown solid (807 mg, 58% yield, 1:1 d.r.). **1j was found to be moderately unstable under ambient conditions. Analytical data reflect 1j as it was used in the subsequent reaction, which proceeded as normal.** HRMS (ESI) m/z: [M+H]+ calculated for $[C_{20}H_{16}O_2+Na]^+$ 311.1042, found 311.1071. ^{1}H NMR (400 MHz, $CDCl_3$) δ 7.90 – 7.82 (m, 8H), 7.80 – 7.78 (m, 1H), 7.55 – 7.45 (m, 7H), 7.41 (dd, *J* = 8.5, 1.7 Hz, 1H), 7.34 – 7.27 (m, 4H), 7.25 – 7.20 (m, 1H), 7.16 (d, *J* = 7.4 Hz, 1H), 7.07 (t, *J* = 8.1 Hz, 1H), 5.34 (d, *J* = 7.9 Hz, 1H), 5.23 (d, *J* = 7.8 Hz, 1H), 4.74 (s, 1H), 4.47 (s, 1H), 3.14 – 3.04 (m, 2H), 2.92 (d, *J* = 17.8 Hz, 1H), 2.80 (d, *J* = 17.6 Hz, 1H), 2.57 (d, *J* = 7.8 Hz, 1H). $^{13}C\{^{1}H\}$ NMR (101 MHz, $CDCl_3$) δ 141.7, 139.1, 133.3, 133.2, 133.2, 129.0, 128.3, 128.1, 128.0, 128.0,

127.9, 127.8, 127.5, 126.6, 126.5, 126.4, 126.2, 125.6, 125.3, 125.1, 125.0, 124.5, 124.3, 123.8, 77.7, 75.2, 74.1, 61.7, 60.2, 33.2, 32.3.

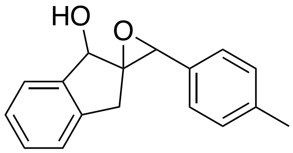

3'-(4-methylphenyl)-1,3-dihydrospiro[indene-2,2'-oxiran]-1-ol (1k): Prepared according to General Procedure C from epoxide **4k** (107 mg, 0.43 mmol). The title compound was recovered in sufficiently pure form directly after aqueous extraction as a white solid (66 mg, 36% yield, 1.2:1 d.r.). HRMS (ESI) m/z: [M+CHO_2]- calculated for $C_{17}H_{15}O$ 297.1132, found 297.1138. ^{1}H NMR (400 MHz, $CDCl_3$) δ 7.54 – 7.48 (m, 1H), 7.45 – 7.40 (m, 1H), 7.32 – 7.20 (m, 7H), 7.18 (s, 5H), 7.09 (dt, *J* = 6.8, 1.1 Hz, 1H), 5.26 (s, 1H), 5.15 (s, 1H), 4.53 (s, 1H), 4.25 (s, 1H), 3.03 (ddd, *J* = 17.5, 2.9, 0.9 Hz, 2H), 2.92 (dt, *J* = 17.8, 1.0 Hz, 1H), 2.80 (dd, *J* = 17.6, 0.9 Hz, 1H), 2.37 (s, 3H), 2.36 (s, 3H). ^{13}C{1H} NMR (101 MHz, $CDCl_3$) δ 142.6, 141.8, 139.3, 139.1, 138.0, 137.8, 132.7, 132.4, 129.2, 129.1, 129.0, 128.8, 127.5, 126.5, 126.2, 125.1, 125.0, 125.0, 124.5, 77.8, 74.8, 74.0, 72.2, 61.6, 60.0, 33.2, 32.3.

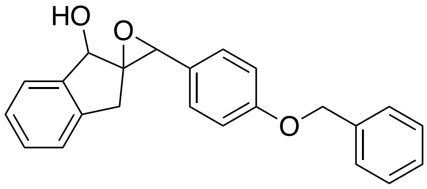

3'-(4-benzyloxyphenyl)-1,3-dihydrospiro[indene-2,2'-oxiran]-1-ol (1l): Prepared according to General Procedure C from epoxide **4l** (713 mg, 2.54 mmol). The title compound was recovered in sufficiently pure form directly after aqueous extraction as a white solid (540 mg, 75% yield 1.5:1 d.r.). **1l was found to be moderately unstable under ambient conditions. Analytical data reflect 1l as it was used in the subsequent reaction, which proceeded as normal.** HRMS (ESI) m/z: [M+H]+ calculated for $[C_{23}H_{20}O_3+Na]^+$ 367.1304, found 367.1326. ^{1}H NMR (400 MHz, $CDCl_3$) δ 7.47 – 7.35 (m, 8H), 7.34 – 7.26 (m, 2H), 7.24 – 7.18 (m, 3H), 7.03 – 6.97 (m, 3H), 5.31 – 5.24 (m, 2H), 5.08 (d, *J* = 5.6 Hz, 4H), 4.52 (s, 1H), 3.09 – 2.99 (m, 1H), 2.82 (d, *J* = 17.6 Hz, 1H). ^{13}C{1H} NMR (101 MHz, $CDCl_3$) δ 158.8, 141.7, 137.0, 129.6, 129.0, 128.8, 128.2, 127.8, 127.6, 127.5, 125.0, 124.4, 114.8, 87.7, 77.9, 77.5, 77.2, 76.8, 74.7, 70.2, 59.8, 32.3.

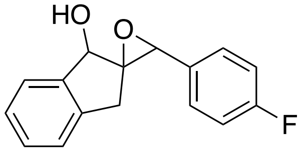

3'-(34-fluorophenyl)-1,3-dihydrospiro[indene-2,2'-oxiran]-1-ol (1m): Prepared according to General Procedure C from epoxide **4m** (253 mg, 0.99 mmol). The title compound was recovered in sufficiently pure form directly after aqueous extraction as a white solid (240 mg, 94% yield, 1:1 d.r.). HRMS (ESI) m/z: [M+H]+ calculated for $[C_{16}H_{13}FO_2+Na]^+$ 279.0792, found 279.0815. ^{1}H NMR (400 MHz, $CDCl_3$) δ 7.50 – 7.46 (m, 1H), 7.42 – 7.38 (m, 1H), 7.33 – 7.20 (m, 8H), 7.17 (d, *J* = 5.3 Hz, 1H), 7.10 – 6.99 (m, 4H), 5.23 (d, *J* = 6.2 Hz, 1H), 4.52 (s, 1H), 4.23 (s, 1H), 3.03 – 2.94 (m, 3H), 2.90 – 2.80 (m, 1H), 2.80 – 2.69 (m, 1H). ^{13}C{1H} NMR (101 MHz, $CDCl_3$) δ 163.9, 163.8, 161.4, 161.3, 142.4, 141.6, 138.9, 138.8, 131.5, 131.4, 131.2, 131.1, 128.9, 128.9, 128.2, 128.1, 127.9, 127.8, 127.5, 127.5, 125.0, 125.0, 124.9, 124.4, 115.6, 115.4, 115.4, 115.2, 77.4, 74.9, 73.9, 72.2, 60.9, 59.5, 33.0, 32.1. ^{19}F NMR (376 MHz, $CDCl_3$) δ -113.71, -114.08.

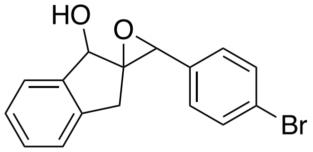

3'-(4-bromophenyl)-1,3-dihydrospiro[indene-2,2'-oxiran]-1-ol (1n): Prepared according to General Procedure C from epoxide **4n** (561 mg, 1.78 mmol). The title compound was recovered in sufficiently pure form directly after aqueous extraction as a white solid (331 mg, 59% yield, mixture of diastereomers) HRMS (ESI) m/z: [M+H]+ calculated for $[C_{16}H_{13}BrO_2+Na]^+$ 338.9991, found 339.0022 ^{1}H NMR (400 MHz, $CDCl_3$) δ 7.59 – 7.48 (m, 5H), 7.46 – 7.40 (m, 1H), 7.35 – 7.26 (m, 4H), 7.25 – 7.16 (m, 5H), 7.11 (dt, *J* = 6.6, 1.1 Hz, 1H), 5.32 – 5.24 (m, 1H), 5.17 (dd, *J* = 8.1, 0.8 Hz, 1H), 4.52 (s, 1H), 4.25 (s, 1H), 3.06 – 2.94 (m, 2H), 2.93 – 2.84 (m, 1H), 2.74 (dt, *J* = 17.6, 0.8 Hz, 1H), 2.47 (d, *J* = 8.1 Hz, 1H), 2.19 – 2.10 (m, 1H). ^{13}C{1H} NMR (101 MHz, $CDCl_3$) δ 142.4, 141.5, 138.9, 138.7, 134.9, 134.6, 131.7, 131.6, 129.1, 129.0, 128.2, 127.9, 127.6, 125.0, 125.0, 124.4, 122.2, 122.0, 77.6, 74.9, 74.0, 72.3, 60.9, 59.5, 33.1, 32.2.

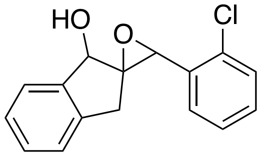

3'-(2-chlorophenyl)-1,3-dihydrospiro[indene-2,2'-oxiran]-1-ol (1o): Prepared according to General Procedure C from epoxide **4o** (182 mg, 0.71 mmol). The title compound was recovered in sufficiently pure form directly after aqueous extraction as a white solid (183 mg, 99% yield, 5:1 d.r.). HRMS (ESI) m/z: [M+Na]+ calculated for $C_{16}H_{13}ClO_2Na$ 295.0496, found 295.0498 ^{1}H NMR (400 MHz, $CDCl_3$) δ 7.56 – 7.45 (m, 1H), 7.45 – 7.21 (m, 6H), 7.18 – 7.05 (m, 1H), 5.33 – 5.23 (m, 1H), 4.73 (s, 1H), 4.48 (s, 0H), 2.99 – 2.85 (m, 1H), 2.75 (dd, *J* = 17.7, 0.8 Hz, 0H), 2.66 (dd, *J* = 17.8, 0.9 Hz, 1H). ^{13}C{1H} NMR (101 MHz, $CDCl_3$) δ 141.7, 139.3, 134.1, 133.1, 129.2, 129.2, 127.6, 127.6, 127.5, 126.9, 125.0, 124.8, 78.1, 74.6, 73.6, 59.2, 58.5, 33.5, 32.7.

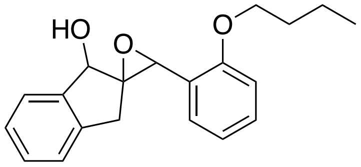

3'-(2-butyloxyphenyl)-1,3-dihydrospiro[indene-2,2'-oxiran]-1-ol (1p): Prepared according to General Procedure C from epoxide **4p** (202 mg, 0.69 mmol). The title compound was recovered in sufficiently pure form directly after aqueous extraction as a white solid (175 mg, 82% yield, 10:1 d.r.). HRMS (ESI) m/z: [M+H]+ calculated for $[C_{22}H_{22}O_3+Na]^+$ 333.1461, found 333.1492. ^{1}H NMR (400 MHz, $CDCl_3$) δ 7.56 – 7.44 (m, 1H), 7.34 – 7.26 (m, 1H), 7.30 – 7.18 (m, 3H), 7.18 – 7.03 (m, 1H), 6.98 (td, *J* = 7.5,

1.1 Hz, 1H), 6.92 – 6.80 (m, 1H), 5.33 – 5.17 (m, 1H), 4.70 (s, 1H), 4.48 (s, 0H), 4.04 – 3.88 (m, 1H), 3.90 – 3.78 (m, 1H), 2.96 – 2.84 (m, 1H), 2.71 – 2.56 (m, 1H), 1.91 (d, J = 9.2 Hz, 1H), 1.75 – 1.64 (m, 0H), 1.57 – 1.43 (m, 2H), 1.43 – 1.35 (m, 0H), 1.32 – 1.15 (m, 2H), 0.89 (t, J = 7.4 Hz, 0H), 0.79 (t, J = 7.4 Hz, 3H). $^{13}C\{^1H\}$ NMR (101 MHz, $CDCl_3$) δ 157.3, 142.2, 139.6, 129.0, 128.9, 128.8, 128.7, 127.4, 126.7, 126.5, 125.0, 125.0, 124.8, 124.7, 124.7, 120.4, 111.0, 111.0, 77.9, 77.5, 77.2, 76.8, 74.2, 73.8, 67.8, 58.5, 57.1, 33.5, 32.7, 31.4, 31.2, 19.4, 19.2, 13.9, 13.8.

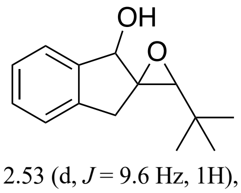

3'-*tert*-butyl-1,3-dihydrospiro[indene-2,2'oxiran]-1-ol (1q): Prepared according to General Procedure B from enone **4q** (2.37 g, 10.9 mmol). The title compound was recovered in sufficiently pure form directly after aqueous extraction as a white solid (1.75 g, 73% yield, 6:1 d.r.). HRMS (ESI) m/z: [M+Na]+ calculated for $C_{14}H_{18}O_2Na$ 241.1199, found 241.1202. ^1H NMR (400 MHz, $CDCl_3$) δ 7.48 (dd, J = 6.5, 2.2 Hz, 1H), 7.31 – 7.26 (m, 2H), 7.24 (dt, J = 5.7, 1.5 Hz, 1H), 5.05 (dd, J = 9.6, 1.0 Hz, 1H), 4.75 (d, J = 6.9 Hz, 0H), 3.58 – 3.35 (m, 1H), 3.19 (s, 0H), 3.17 – 3.03 (m, 1H), 2.97 (s, 1H), 2.53 (d, J = 9.6 Hz, 1H), 1.76 (d, J = 7.1 Hz, 0H), 1.09 (s, 9H), 1.07 (s, 1H). $^{13}C\{^1H\}$ NMR (101 MHz, $CDCl_3$) δ 142.5, 138.9, 128.5, 127.4, 124.8, 124.7, 74.3, 69.2, 67.6, 34.8, 31.8, 27.1.

β-NAPHTHOLS

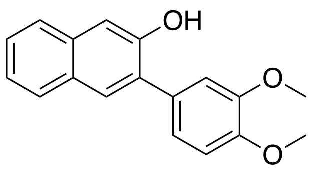

3-(3,4-dimethoxyphenyl)-2-naphthol (2a): Prepared according to General Procedure D from epoxy alcohol **1a** (220 mg, 0.74 mmol). The residue was purified by flash chromatography on SiO_2 (gradient elution, 0 to 30% EtOAc in hexane) to give the title compound as a reddish solid (203 mg, 98% yield). HRMS (ESI) m/z: [M+H]+ calculated for $[C_{18}H_{16}O_3+H]^+$ 281.1172, found 281.1182. ^1H NMR (400 MHz, $CDCl_3$) δ 7.77 (ddd, J = 8.0, 1.4, 0.6 Hz, 1H), 7.74 – 7.70 (m, 2H), 7.43 (ddd, J = 8.2, 6.8, 1.3 Hz, 1H), 7.36 – 7.30 (m, 2H), 7.11 (dd, J = 8.1, 2.0 Hz, 1H), 7.06 (d, J = 2.0 Hz, 1H), 7.01 (d, J = 8.1 Hz, 1H), 5.41 (s, 1H), 3.95 (s, 3H), 3.93 (s, 3H). $^{13}C\{^1H\}$ NMR (101 MHz, $CDCl_3$) δ 151.1, 149.7, 149.3, 134.4, 130.4, 129.4, 129.4, 129.0, 127.8, 126.5, 126.4, 124.0, 121.6, 112.7, 111.9, 110.2, 56.2.

Gram scale preparation of 2a: A flame-dried roundbottom flask equipped with a magnetic stir bar, under nitrogen, was charged with epoxy alcohol **1a** (4.54 g, 15.2 mmol, 1.0 eq.). CH_2Cl_2 (75 mL) was added *via* syringe and the resulting solution was cooled to 0°C on an ice-water bath. Trifluoroacetic acid (1.16 mL, 15.2 mL, 1.0 eq.) was then added dropwise *via* syringe. The reaction was monitored by TLC analysis. Once deemed complete, the reaction mixture was directly concentrated *in vacuo* to remove all volatiles. The dark purple residue was then purified by flash chromatography on SiO_2 (gradient elution, 0 to 40% EtOAc in hexane) to give **2a** as a reddish solid (4.40 g, 99% yield).

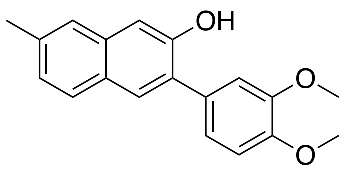

3-(3,4-dimethoxyphenyl)-6-methyl-2-naphthol (2b): Prepared according to General Procedure D from epoxy alcohol **1b** (219 mg, 0.70 mmol). The residue was purified by flash chromatography on SiO_2 (gradient elution, 0 to 20% EtOAc in hexane) to give the title compound as a tan solid (140 mg, 68% yield). HRMS (ESI) m/z: [M+H]+ calculated for $[C_{19}H_{18}O_3+H]^+$ 295.1329, found 295.1329. ^1H NMR (400 MHz, $CDCl_3$) δ 7.63 (t, J = 4.2 Hz, 2H), 7.55 – 7.53 (m, 1H), 7.10 (dd, J = 8.1, 2.0 Hz, 1H), 7.05 (d, J = 2.0 Hz, 1H), 7.01 (d, J = 8.2 Hz, 1H), 5.28 (s, 1H), 3.95 (s, 3H), 3.93 (s, 3H), 2.48 (d, J = 1.0 Hz, 3H). $^{13}C\{^1H\}$ NMR (101 MHz, $CDCl_3$) δ 150.4, 149.7, 149.2, 133.4, 132.5, 130.3, 129.6, 129.2, 128.9, 128.7, 126.7, 126.3, 121.6, 112.7, 111.9, 110.1, 56.2, 21.7.

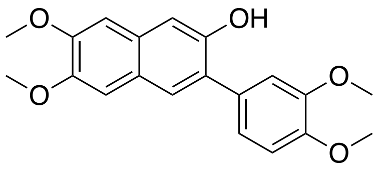

5,6-dimethoxy-3-(3,4-dimethoxyphenyl)-2-naphthol (2c): Prepared according to General Procedure D from epoxy alcohol **1c** (179 mg, 0.50 mmol). The residue was purified by flash chromatography on SiO_2 (gradient elution, 0 to 30% EtOAc in hexane) to give the title compound as a brown solid (132 mg, 78% yield). HRMS (ESI) m/z: [M+H]+ calculated for $[C_{20}H_{20}O_5+H]^+$ 341.1384, found 341.1390. ^1H NMR (400 MHz, $CDCl_3$) δ 7.56 (s, 1H), 7.20 (s, 1H), 7.11 – 7.06 (m, 2H), 7.04 (d, J = 2.0 Hz, 1H), 7.02 – 6.98 (m, 2H), 4.00 (s, 3H), 3.97 (s, 3H), 3.94 (s, 3H), 3.92 (s, 3H). $^{13}C\{^1H\}$ NMR (101 MHz, $CDCl_3$) δ 150.3, 149.9, 149.7, 149.0, 148.2, 130.2, 129.7, 128.1, 127.7, 124.3, 121.5, 112.6, 111.8, 109.5, 106.4, 105.1, 56.2, 56.1, 56.0, 56.0.

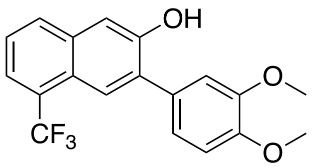

3-(3,4-dimethoxyphenyl)-4-(trifluoromethyl)-2-naphthol (2d): Prepared according to General Procedure D from epoxy alcohol **1d** (114 mg, 0.31 mmol). The residue was purified by flash chromatography on SiO_2 (gradient elution, 0 to 20% EtOAc in hexane) to give the title compound as a tan solid (26 mg, 25% yield). HRMS (ESI) m/z: [M-H]- calculated for $[C_{19}H_{15}F_3O_3-H]^-$ 347.0900, found 347.0909. ^1H NMR (400 MHz, $CDCl_3$) δ 7.95 (d, J = 8.2 Hz, 1H), 7.86 – 7.78 (m, 2H), 7.67 (d, J = 2.1 Hz, 1H), 7.37 (t, J = 7.7 Hz, 1H), 7.12 (dd, J = 8.2, 2.1 Hz, 1H), 7.08 – 7.00 (m, 2H), 5.54 (s, 1H), 3.96 (s, 3H), 3.94 (s, 3H). $^{13}C\{^1H\}$ NMR (101 MHz, $CDCl_3$) δ 152.5, 149.9, 149.6, 132.5, 131.2, 130.1, 130.0, 129.6, 128.6, 125.4, 125.3, 124.9, 124.6, 122.3, 121.6, 112.5, 112.0, 107.5, 107.5, 56.2. ^{19}F NMR (376 MHz, $CDCl_3$) δ -60.6.

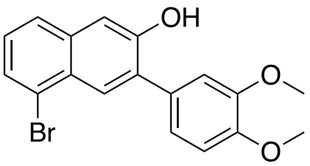

4-bromo-3-(3,4-dimethoxyphenyl)-2-naphthol (2e): Prepared according to General Procedure D from epoxy alcohol **1e** (139 mg, 0.37 mmol). The residue was purified by flash chromatography on SiO_2 (gradient elution, 0 to 30% EtOAc in hexane) to give the title compound as a brown solid (60 mg, 46% yield). HRMS (ESI) m/z: [M-H]- calculated for $[C_{18}H_{15}BrO_3\text{-}H]^-$ 357.0132, found 357.0136. ^{1}H NMR (400 MHz, $CDCl_3$) δ 7.75 (dd, *J* = 4.7, 1.0 Hz, 1H), 7.72 – 7.68 (m, 1H), 7.21 – 7.09 (m, 3H), 7.07 – 7.01 (m, 2H), 5.53 (s, 1H), 3.96 (s, 3H), 3.94 (s, 3H). ^{13}C{1H} NMR (101 MHz, $CDCl_3$) δ 152.4, 149.8, 149.5, 133.1, 131.3, 130.4, 130.0, 129.9, 128.7, 127.7, 124.3, 121.6, 121.0, 112.6, 112.0, 110.1, 56.2.

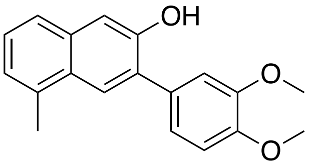

3-(3,4-dimethoxyphenyl)-4-methyl-2-naphthol (2f): Prepared according to General Procedure D from epoxy alcohol **1f** (96 mg, 0.31 mmol). The residue was purified by flash chromatography on SiO_2 (gradient elution, 0 to 20% EtOAc in hexane) to give the title compound as a tan solid (22 mg, 25% yield). HRMS (ESI) m/z: [M+H]+ calculated for $[C_{19}H_{18}O_3\text{+}H]^+$ 295.1329, found 295.1329. ^{1}H NMR (400 MHz, $CDCl_3$) δ 7.73 (s, 1H), 7.67 – 7.62 (m, 1H), 7.47 (d, *J* = 0.8 Hz, 1H), 7.28 (ddd, *J* = 6.9, 1.6, 0.8 Hz, 1H), 7.22 (s, 1H), 7.12 (dd, *J* = 8.1, 2.0 Hz, 1H), 7.07 – 7.01 (m, 2H), 5.42 (s, 1H), 3.96 (s, 3H), 3.94 (s, 3H), 2.65 (s, 3H). ^{13}C{1H} NMR (101 MHz, $CDCl_3$) δ 150.9, 149.8, 149.2, 133.6, 132.9, 129.9, 129.8, 129.3, 129.0, 127.1, 126.2, 123.8, 121.5, 112.6, 111.9, 107.2, 56.2, 19.6.

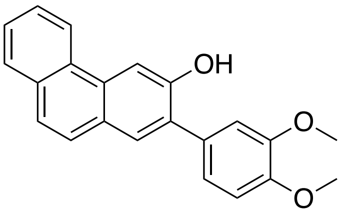

3-(3,4-dimethoxyphenyl)phenanthren-2-ol (2g): Prepared according to General Procedure D from epoxy alcohol **1g** (101 mg, 0.70 mmol). The residue was purified by flash chromatography on SiO_2 (gradient elution, 0 to 30% EtOAc in hexane) to give the title compound as a tan solid (50 mg, 52% yield). HRMS (ESI) m/z: [M+H]+ calculated for $[C_{22}H_{18}O_3\text{+}H]^+$ 331.1329, found 331.1341. ^{1}H NMR (400 MHz, $CDCl_3$) δ 8.57 (d, *J* = 8.2 Hz, 1H), 8.53 (s, 1H), 7.85 (dd, *J* = 7.8, 1.5 Hz, 1H), 7.73 – 7.50 (m, 5H), 7.41 (s, 1H), 7.16 (dd, *J* = 8.1, 2.0 Hz, 1H), 7.11 (d, *J* = 2.0 Hz, 1H), 7.05 (d, *J* = 8.1 Hz, 1H), 5.52 (br s, 1H), 3.97 (s, 3H), 3.95 (s, 3H). ^{13}C{1H} NMR δ 151.7, 149.9, 149.3, 133.3, 131.3, 130.6, 129.7, 129.3, 128.8, 127.8, 126.8, 126.1, 125.9, 124.8, 124.7, 124.2, 122.2, 121.7, 112.8, 112.4, 112.1, 56.25, 56.23.

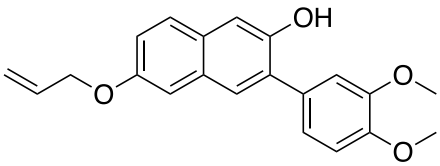

5-allyloxy-3-(3,4-dimethoxyphenyl)-2-naphthol (2h): Prepared according to General Procedure D from epoxy alcohol **1h** (160 mg, 0.45 mmol). The residue was purified by flash chromatography on SiO_2 (gradient elution, 0 to 30% EtOAc in hexane) to give the title compound as a brown solid (30 mg, 20% yield). HRMS (ESI) m/z: [M+H]+ calculated for $[C_{21}H_{20}O_4\text{+}Na]^+$ 359.1254, found 359.1287. ^{1}H NMR (400 MHz, $CDCl_3$) δ 7.68 – 7.65 (m, 1H), 7.63 (s, 1H), 7.22 (s, 1H), 7.08 (dd, *J* = 8.1, 2.0 Hz, 1H), 7.05 – 6.99 (m, 4H), 6.13 (ddt, *J* = 17.2, 10.5, 5.3 Hz, 1H), 5.48 (dq, *J* = 17.2, 1.6 Hz, 1H), 5.35 – 5.30 (m, 2H), 4.66 (dt, *J* = 5.4, 1.5 Hz, 2H), 3.95 (s, 3H), 3.93 (s, 3H). ^{13}C{1H} NMR (101 MHz, $CDCl_3$) δ 157.4, 151.6, 149.8, 149.2, 135.7, 133.3, 129.5, 129.4, 129.1, 128.0, 124.6, 121.5, 117.9, 117.1, 112.7, 112.0, 109.4, 105.8, 69.0, 56.2.

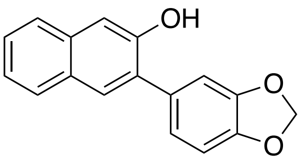

3-(3,4-methylenedioxyphenyl)-2-naphthol (2i): Prepared according to General Procedure D from epoxy alcohol **1i** (382 mg, 1.35 mmol). The residue was purified by flash chromatography on SiO_2 (gradient elution, 0 to 30% EtOAc in hexane) to give the title compound as a brown solid (259 mg, 73% yield). HRMS (ESI) m/z: [M-H]- calculated for $C_{17}H_{11}O_3$ 263.0713, found 263.0715. ^{1}H NMR (400 MHz, $CDCl_3$) δ 7.79 – 7.67 (m, 3H), 7.42 (ddd, *J* = 8.2, 6.8, 1.3 Hz, 1H), 7.39 – 7.31 (m, 1H), 7.31 (d, *J* = 1.0 Hz, 1H), 7.06 – 6.98 (m, 2H), 6.95 (dd, *J* = 7.8, 0.6 Hz, 1H), 6.04 (s, 2H), 5.50 – 5.11 (br s, 1H). ^{13}C{1H} NMR (101 MHz, $CDCl_3$) δ 151.0, 148.6, 147.8, 134.4, 130.7, 130.2, 129.5, 129.0, 127.8, 126.6, 126.4, 124.0, 122.8, 110.3, 110.1, 109.1, 101.5.

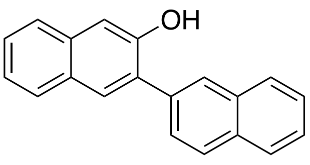

3-(2-naphthyl)-2-naphthol (2j): Prepared according to General Procedure D from epoxy alcohol **1j** (150 mg, 0.51 mmol). The residue was purified by flash chromatography on SiO_2 (gradient elution, 0 to 30% EtOAc in hexane) to give the title compound as a white solid (140 mg, quantitative yield). HRMS (ESI) m/z: [M-H]- calculated for $C_{20}H_{13}O$ 269.0972, found 269.0974. ^{1}H NMR (400 MHz, $CDCl_3$) δ 8.05 (d, *J* = 1.7 Hz, 1H), 8.01 (d, *J* = 8.5 Hz, 1H), 7.94 – 7.90 (m, 2H), 7.84 (s, 1H), 7.83 – 7.79 (m, 1H), 7.77 – 7.74 (m, 1H), 7.68 (dd, *J* = 8.4, 1.8 Hz, 1H), 7.58 – 7.54 (m, 2H), 7.46 (ddd, *J* = 8.2, 6.8, 1.3 Hz, 1H), 7.39 – 7.34 (m, 2H), 5.33 (s, 1H). ^{13}C{1H} NMR (101 MHz, $CDCl_3$) δ 151.1, 134.5, 134.5, 133.7, 133.0, 130.6, 130.0, 129.2, 129.2, 128.4, 128.3, 128.0, 128.0, 127.4, 126.9, 126.7, 126.4, 124.1, 110.5.

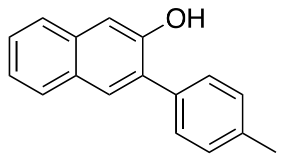

3-(4-methylphenyl)-2-naphthol (2k): Prepared according to General Procedure D from epoxy alcohol **1k** (65 mg, 0.26 mmol). The residue was purified by flash chromatography on SiO_2 (gradient elution, 0 to 30% EtOAc in hexane) to give the title compound as a white solid (49 mg, 82% yield). HRMS (ESI) m/z: [M+H]+ calculated for $C_{17}H_{15}O$ 235.1229, found 235.1240. ^{1}H NMR (400 MHz, $CDCl_3$) δ 7.80 – 7.75 (m, 1H), 7.72 (d, J = 7.6 Hz, 2H), 7.47 – 7.40 (m, 3H), 7.35 – 7.30 (m, 4H), 5.29 (s, 1H), 2.44 (s, 3H). ^{13}C{1H} NMR (101 MHz, $CDCl_3$) δ 151.1, 138.2, 134.4, 134.0, 130.6, 130.1, 129.5, 129.3, 129.1, 127.9, 126.5, 126.4, 124.0, 110.2, 21.4.

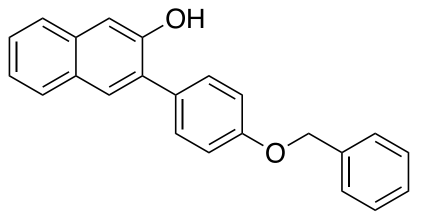

3-(4-benzyloxyphenyl)-2-naphthol (2l): Prepared according to General Procedure D from epoxy alcohol **1l** (146 mg, 0.42 mmol). The residue was purified by flash chromatography on SiO_2 (gradient elution, 0 to 40% EtOAc in hexane) to give the title compound as a white solid (71 mg, 52% yield). HRMS (ESI) m/z: [M+H]+ calculated for $[C_{23}H_{18}O_2+H]^+$ 327.1380, found 327.1410. ^{1}H NMR (400 MHz, $CDCl_3$) δ 7.78 – 7.74 (m, 1H), 7.74 – 7.69 (m, 2H), 7.51 – 7.45 (m, 4H), 7.45 – 7.39 (m, 3H), 7.38 – 7.31 (m, 3H), 7.15 – 7.11 (m, 2H), 5.27 (s, 1H), 5.15 (s, 2H). ^{13}C{1H} NMR (101 MHz, $CDCl_3$) δ 159.0, 151.1, 134.3, 130.7, 129.5, 129.4, 129.1, 128.8, 128.3, 127.8, 127.6, 126.5, 126.4, 124.0, 115.8, 110.2, 77.5, 77.2, 76.8, 70.3.

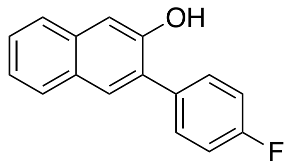

3-(4-fluorophenyl)-2-naphthol (2m): Prepared according to General Procedure D from epoxy alcohol **1m** (203 mg, 0.79 mmol). The residue was purified by flash chromatography on SiO_2 (gradient elution, 0 to 20% EtOAc in hexane) to give the title compound as a white solid (102 mg, 54% yield). HRMS (ESI) m/z: [M+Na]+ calculated for $C_{16}H_{11}FONa$ 261.0692, found 261.0696. ^{1}H NMR (400 MHz, $CDCl_3$) δ 7.79 – 7.75 (m, 1H), 7.74 – 7.70 (m, 2H), 7.58 – 7.52 (m, 2H), 7.44 (ddd, J = 8.3, 6.8, 1.2 Hz, 1H), 7.34 (ddd, J = 8.1, 6.9, 1.2 Hz, 1H), 7.30 (d, J = 0.8 Hz, 1H), 7.24 – 7.16 (m, 2H), 5.15 (s, 1H). ^{13}C{1H} NMR (101 MHz, $CDCl_3$) δ 164.0, 161.6, 150.9, 134.4, 133.1, 133.1, 131.3, 131.2, 129.9, 129.7, 129.1, 127.9, 126.7, 126.3, 124.2, 116.3, 116.1, 110.5. ^{19}F NMR (376 MHz, $CDCl_3$) δ -113.9.

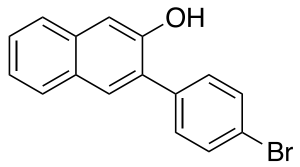

3-(4-bromophenyl)-2-naphthol (2n): Prepared according to General Procedure D from epoxy alcohol **1n** (97 mg, 0.31 mmol). The residue was purified by flash chromatography on SiO_2 (gradient elution, 0 to 20% EtOAc in hexane) to give the title compound as a white solid (33 mg, 33% yield). HRMS (ESI) m/z: [M-H]- calculated for $C_{16}H_{10}BrO$ 296.9920, found 296.9923. ^{1}H NMR (400 MHz, $CDCl_3$) δ 7.79 – 7.76 (m, 1H), 7.71 (d, J = 6.8 Hz, 2H), 7.66 – 7.62 (m, 2H), 7.49 – 7.42 (m, 3H), 7.35 (ddd, J = 8.1, 6.9, 1.3 Hz, 1H), 7.29 (s, 1H), 4.86 – 3.86 (br s, 1H).

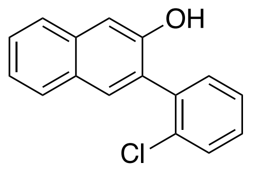

3-(2-chlorophenyl)-2-naphthol (2o): Prepared according to General Procedure D from epoxy alcohol **1o** (104 mg, 0.31 mmol). The residue was purified by flash chromatography on SiO_2 (gradient elution, 0 to 20% EtOAc in hexane) to give the title compound as a brown solid (32 mg, 33% yield). HRMS (ESI) m/z: [M-H]- calculated for $C_{16}H_{10}ClO$ 253.0425, found 253.0425. ^{1}H NMR (400 MHz, $CDCl_3$) δ 7.79 – 7.76 (m, 1H), 7.71 (d, J = 6.8 Hz, 2H), 7.66 – 7.62 (m, 2H), 7.49 – 7.42 (m, 3H), 7.35 (ddd, J = 8.1, 6.9, 1.3 Hz, 1H), 7.29 (s, 1H), 4.87 – 3.89 br s, 1H). ^{13}C{1H} NMR (101 MHz, $CDCl_3$) δ 151.0, 135.9, 134.8, 134.4, 132.4, 130.3, 130.2, 130.0, 128.8, 128.5, 128.0, 127.4, 126.9, 126.5, 124.1, 110.5.

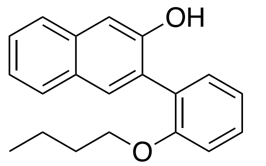

3-(2-butyloxyphenyl)-2-naphthol (2p): Prepared according to General Procedure D from epoxy alcohol **1q** (100 mg, 0.33 mmol). The residue was purified by flash chromatography on SiO_2 (gradient elution, 0 to 30% EtOAc in hexane) to give the title compound as a brown oil (32 mg, 34% yield). HRMS (ESI) m/z: [M+K]+ calculated for $C_{20}H_{20}O_2$+K 331.1095, found 331.1112. ^{1}H NMR (400 MHz, $CDCl_3$) δ 7.81 – 7.74 (m, 3H), 7.50 – 7.38 (m, 4H), 7.34 (ddd, J = 8.1, 6.8, 1.3 Hz, 1H), 7.15 (td, J = 7.5, 1.1 Hz, 1H), 7.08 (dd, J = 8.2, 1.1 Hz, 1H), 6.57 (s, 1H), 4.07 (t, J = 6.5 Hz, 2H), 1.70 (ddt, J = 9.0, 7.8, 6.4 Hz, 2H), 1.40 – 1.32 (m, 2H), 0.86 (t, J = 7.4 Hz, 3H). ^{13}C{1H} NMR (101 MHz, $CDCl_3$) δ 155.5, 152.4, 134.6, 133.1, 130.7, 129.7, 129.5, 129.2, 127.9, 127.8, 126.5, 126.4, 123.8, 122.5, 113.3, 112.3, 69.6, 31.2, 19.2, 13.8.

---DERIVATIVES---

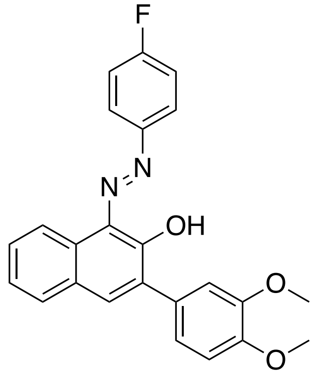

1-(3-(3,4-dimethoxyphenyl)-2-hydroxy-1-naphthyl)-2-(4-fluorophenyl)diazene (11): Naphthol **2a** (282 mg, 1.00 mmol, 1.0 eq.) was charged to a flame-dried, 25 mL roundbottom flask equipped with a magnetic stir bar and placed under N_2. The solid was taken up in CH_2Cl_2 (3 mL) at room temperature and 4-fluoroaniline (0.14 mL, 1.50 mmol, 1.5 eq.) was added *via* syringe. *tert*-Butyl nitrite (0.18 mL, 1.50 mmol, 1.5 eq.) was then added dropwise *via* syringe and the reaction mixture was allowed to stir overnight. Upon completion of the reaction as indicated by TLC analysis, the volatiles were removed *in vacuo* and the residue was directly purified by flash chromatography on SiO_2 (gradient elution, 0 to 40% EtOAc in hexane) to afford the title compound as a dark red solid (326 mg, 80% yield). HRMS (ESI) m/z: [M+K]+ calculated for $C_{24}H_{19}FN_2O_3K$ 441.1012, found 441.1025. ^1H NMR (400 MHz, $CDCl_3$) δ 8.58 – 8.52 (m, 1H), 7.79 (s, 1H), 7.76 – 7.70 (m, 2H), 7.67 – 7.63 (m, 1H), 7.55 (ddd, *J* = 8.3, 7.1, 1.4 Hz, 1H), 7.42 (ddd, *J* = 8.1, 7.1, 1.2 Hz, 1H), 7.23 – 7.13 (m, 5H), 6.99 (d, *J* = 8.8 Hz, 1H), 3.96 (s, 3H), 3.95 (s, 3H). $^{13}C\{1H\}$ NMR (101 MHz, $CDCl_3$) δ 169.7, 163.3, 160.8, 149.1, 148.8, 141.5, 141.5, 138.5, 135.7, 133.2, 130.2, 129.4, 128.8, 128.7, 128.2, 126.1, 121.9, 121.6, 120.4, 120.4, 116.9, 116.6, 112.9, 111.2, 56.2. ^{19}F NMR (376 MHz, $CDCl_3$) δ -113.5.

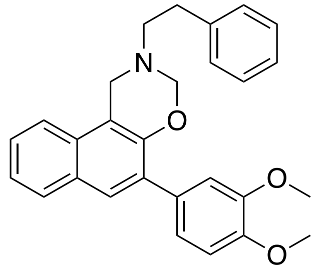

Hemiaminal 12: Naphthol **2a** (504 mg, 1.79 mmol, 1.0 eq.) was charged to a flame-dried, 25 mL roundbottom flask equipped with a magnetic stir bar and placed under N_2. The solid was taken up in MeOH (0.90 mL). To this solution was added phenethylamine (0.34 mL, 2.7 mmol, 1.5 eq.) *via* syringe, followed by 37% aqueous formaldehyde (0.94 mL, 11.5 mmol, 6.4 eq.) dropwise *via* syringe. The resulting mixture was heated to 58°C for 20 hours, at which point 5 mL of saturated aqueous $NaHCO_3$ were added. The mixture was partitioned between water (10 mL) and EtOAc (20 mL) and the layers separated. The aqueous layer was extracted 3x EtOAc (10 mL), and the combined organics were washed three times with water, once with brine, then dried over $MgSO_4$ and concentrated *in vacuo*. The residue was purified by flash chromatography on SiO_2 (gradient elution, 0 to 20% EtOAc in hexane) to give the title compound as a tacky, light brown solid (497 mg, 67% yield). HRMS (ESI) m/z: [M+H]+ calculated for $C_{28}H_{28}NO_3$ 426.2064, found 426.2051. ^1H NMR (400 MHz, $CDCl_3$) δ 7.77 – 7.72 (m, 1H), 7.64 (s, 1H), 7.58 (dd, *J* = 8.4, 1.1 Hz, 1H), 7.43 (ddd, *J* = 8.3, 6.8, 1.3 Hz, 1H), 7.33 (ddd, *J* = 8.0, 6.8, 1.1 Hz, 1H), 7.26 – 7.20 (m, 3H), 7.16 (ddt, *J* = 8.3, 3.7, 1.8 Hz, 6H), 4.92 (s, 2H), 4.38 (s, 2H), 3.89 (s, 3H), 3.87 (s, 3H), 3.12 – 3.06 (m, 2H), 2.91 (dd, *J* = 9.1, 6.3 Hz, 2H). $^{13}C\{1H\}$ NMR (101 MHz, $CDCl_3$) δ 149.7, 148.5, 148.5, 140.3, 139.8, 131.3, 131.1, 130.6, 128.7, 128.7, 128.6, 128.5, 128.4, 128.3, 128.1, 126.3, 126.1, 126.0, 123.8, 122.1, 120.7, 113.2, 112.2, 110.9, 82.4, 74.4, 55.9, 55.9, 54.4, 53.8, 48.3, 35.0, 34.5.

X-RAY CRYSTALLOGRAPHIC DATA FOR 2a

Sample Preparation and Measurement: 1.0 mL of a saturated solution of **2a** in CH_2Cl_2 was added to a flame-dried 1-dram vial. The vial was placed into a 20 mL scintillation vial containing 4-5 mL of *n*-pentane. The scintillation vial was tightly closed, and the vapors were allowed to diffuse over several days. A suitable crystal was selected and mounted for analysis. Data were collected using a Bruker APEX-II CCD X-Ray diffractometer.

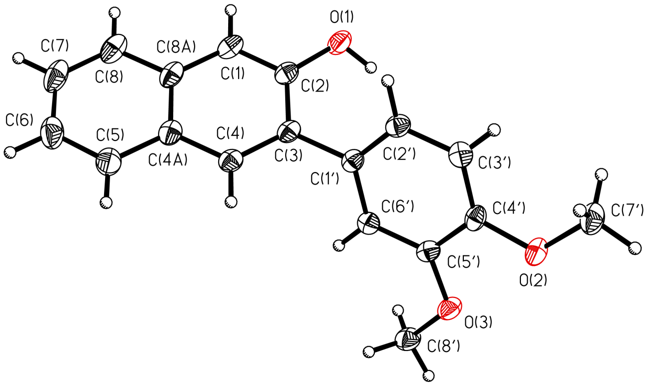


```
Bond precision:   C-C = 0.0019 A           Wavelength=0.71073

Cell:            a=10.8502(10)    b=9.7454(9)        c=13.8484(13)
                 alpha=90         beta=106.048(1)    gamma=90
Temperature:     125 K

                 Calculated                  Reported
Volume           1407.3(2)                   1407.3(2)
Space group      P 21/c                      P 1 21/c 1
Hall group       -P 2ybc                     -P 2ybc
Moiety formula   C18 H16 O3                  C18 H16 O3
Sum formula      C18 H16 O3                  C18 H16 O3
Mr               280.31                      280.31
Dx,g cm-3        1.323                       1.323
Z                4                           4
Mu (mm-1)        0.089                       0.089
F000             592.0                       592.0
F000'            592.30
h,k,lmax         13,11,16                    13,11,16
Nref             2602                        2603
Tmin,Tmax        0.987,0.992                 0.910,0.990
Tmin'            0.972

Correction method= # Reported T Limits: Tmin=0.910 Tmax=0.990
AbsCorr = MULTI-SCAN

Data completeness= 1.000           Theta(max)= 25.430

                                                 wR2(reflections)=
R(reflections)= 0.0335( 2133)                    0.0920( 2603)

S = 1.042                    Npar= 195
```

REFERENCES

(1) Bansal, R.; Narang, G.; Zimmer, C.; Hartmann, R. W. Synthesis of Some Imidazolyl-Substituted 2-Benzylidene Indanone Derivatives as Potent Aromatase Inhibitors for Breast Cancer Therapy. *Med Chem Res* **2011**, *20* (6), 661–669. https://doi.org/10.1007/s00044-010-9368-4.

(2) Huang, L.; Lu, C.; Sun, Y.; Mao, F.; Luo, Z.; Su, T.; Jiang, H.; Shan, W.; Li, X. Multitarget-Directed Benzylideneindanone Derivatives: Anti-β-Amyloid (Aβ) Aggregation, Antioxidant, Metal Chelation, and Monoamine Oxidase B (MAO-B) Inhibition Properties against Alzheimer's Disease. *J. Med. Chem.* **2012**, *55* (19), 8483–8492. https://doi.org/10.1021/jm300978h.

(3) Lubbe, A. S.; Ruangsupapichat, N.; Caroli, G.; Feringa, B. L. Control of Rotor Function in Light-Driven Molecular Motors. *J. Org. Chem.* **2011**, *76* (21), 8599–8610. https://doi.org/10.1021/jo201583z.

(4) Nagarapu, L.; Vulupala, H. R.; Bantu, R.; Sajja, Y.; Nanubolu, J. B. Efficient Synthesis and Resolution of Novel 2-(Hydroxymethyl)-7,8-Dihydro-1*H*-Indeno[5,4-*b*] Furan-6(2*H*)-One by Lipase *Pseudomonas Cepacia*. *Tetrahedron: Asymmetry* **2014**, *25* (6), 578–582. https://doi.org/10.1016/j.tetasy.2014.02.008.

(5) Kayal, S.; Mukherjee, S. Catalytic Enantioselective Cascade Michael/Cyclization Reaction of 3-Isothiocyanato Oxindoles with Exocyclic α,β-Unsaturated Ketones En Route to 3,2′-Pyrrolidinyl Bispirooxindoles. *Org. Biomol. Chem.* **2016**, *14* (43), 10175–10179. https://doi.org/10.1039/C6OB02187E.

(6) Adam, W.; Halász, J.; Jámbor, Z.; Lévai, A.; Nemes, C.; Patonay, T.; Tóth, G. Stereoselective Epoxidation of 2-Arylidene-1-Indanones and 2-Arylidene-1-Benzosuberones. *Monatsh Chem* **1996**, *127* (6), 683–690. https://doi.org/10.1007/BF00817259.

(7) Kadayat, T. M.; Banskota, S.; Gurung, P.; Bist, G.; Thapa Magar, T. B.; Shrestha, A.; Kim, J.-A.; Lee, E.-S. Discovery and Structure-Activity Relationship Studies of 2-Benzylidene-2,3-Dihydro-1*H*-Inden-1-One and Benzofuran-3(2*H*)-One Derivatives as a Novel Class of Potential Therapeutics for Inflammatory Bowel Disease. *European Journal of Medicinal Chemistry* **2017**, *137*, 575–597. https://doi.org/10.1016/j.ejmech.2017.06.018.

(8) Gadekar, S. C.; Dhayalan, V.; Nandi, A.; Zak, I. L.; Mizrachi, M. S.; Kozuch, S.; Milo, A. Rerouting the Organocatalytic Benzoin Reaction toward Aldehyde Deuteration. *ACS Catal.* **2021**, *11* (23), 14561–14569. https://doi.org/10.1021/acscatal.1c04583.

(9) Camps, P.; Domingo, L. R.; Formosa, X.; Galdeano, C.; González, D.; Muñoz-Torrero, D.; Segalés, S.; Font-Bardia, M.; Solans, X. Highly Diastereoselective One-Pot Synthesis of Spiro{cyclopenta[a]Indene-2,2‘-Indene}diones from 1-Indanones and Aromatic Aldehydes. *J. Org. Chem.* **2006**, *71* (9), 3464–3471. https://doi.org/10.1021/jo0600095.

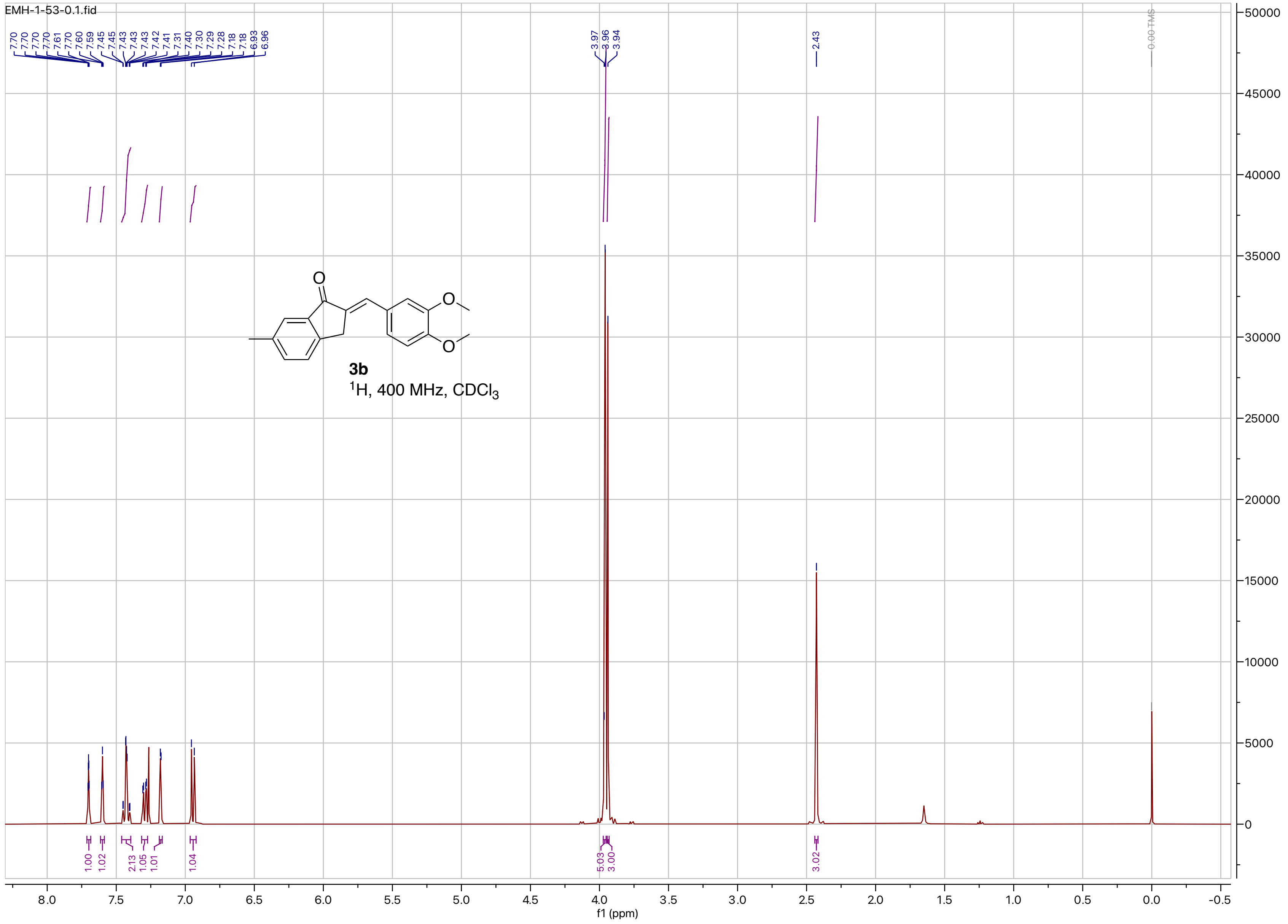
EMH-1-53-0.1.fid
7.70
7.70
7.70
7.70
7.61
7.70
7.60
7.59
7.45
7.43
7.43
7.43
7.42
7.41
7.31
7.40
7.30
7.29
7.28
7.18
7.18
6.93
6.96
3.97
3.96
3.94
2.43
0.00 TMS
O
O
O
3b
1H, 400 MHz, CDCl3
1.00
1.02
2.13
1.05
1.01
1.04
5.03
3.00
3.02
f1 (ppm)

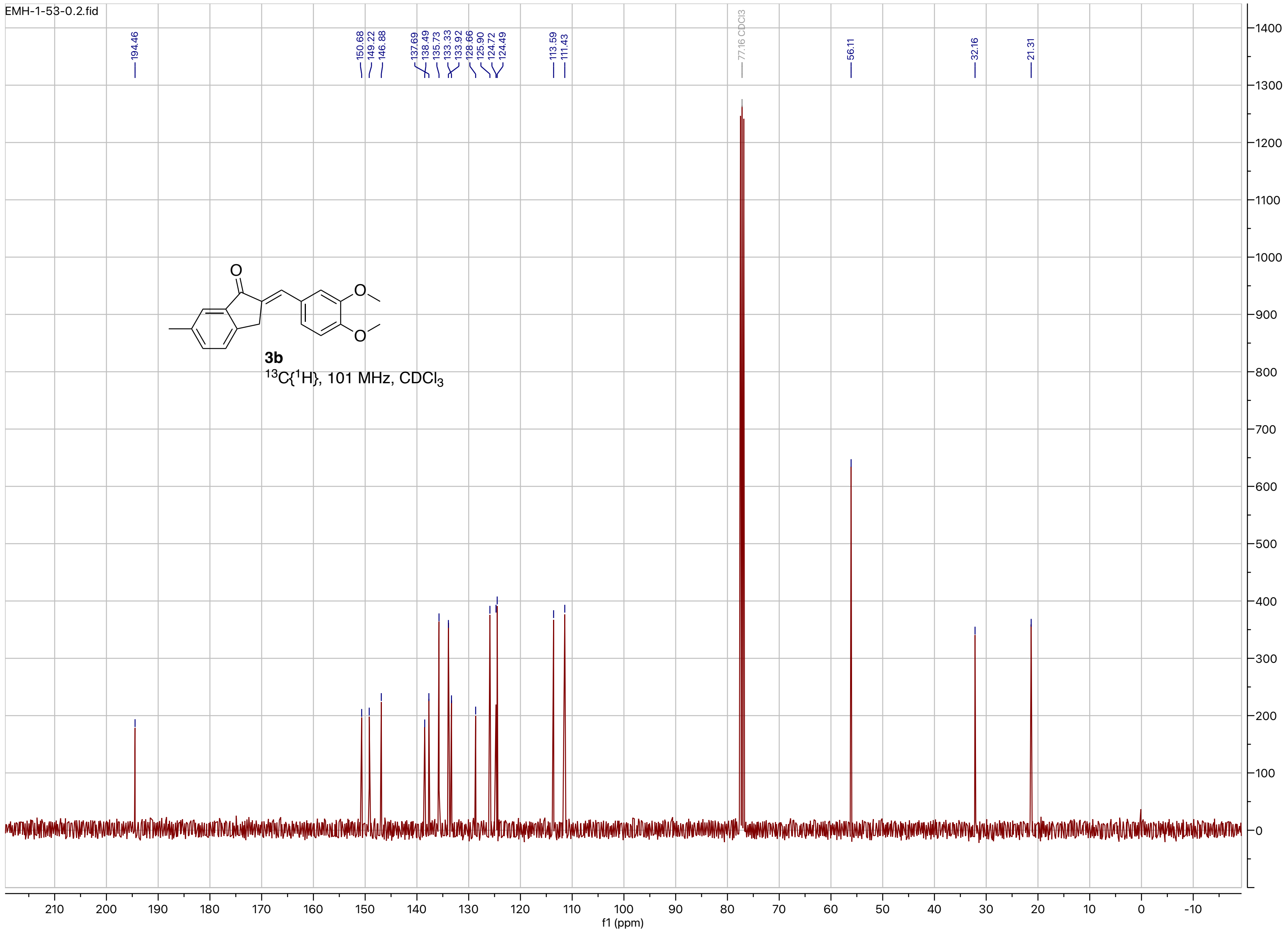
EMH-1-53-0.2.fid
194.46
150.68
149.22
146.88
137.69
138.49
135.73
133.33
133.92
128.66
125.90
124.72
124.49
113.59
111.43
77.16 CDCl3
56.11
32.16
21.31
3b
$^{13}C\{^1H\}$, 101 MHz, $CDCl_3$
f1 (ppm)

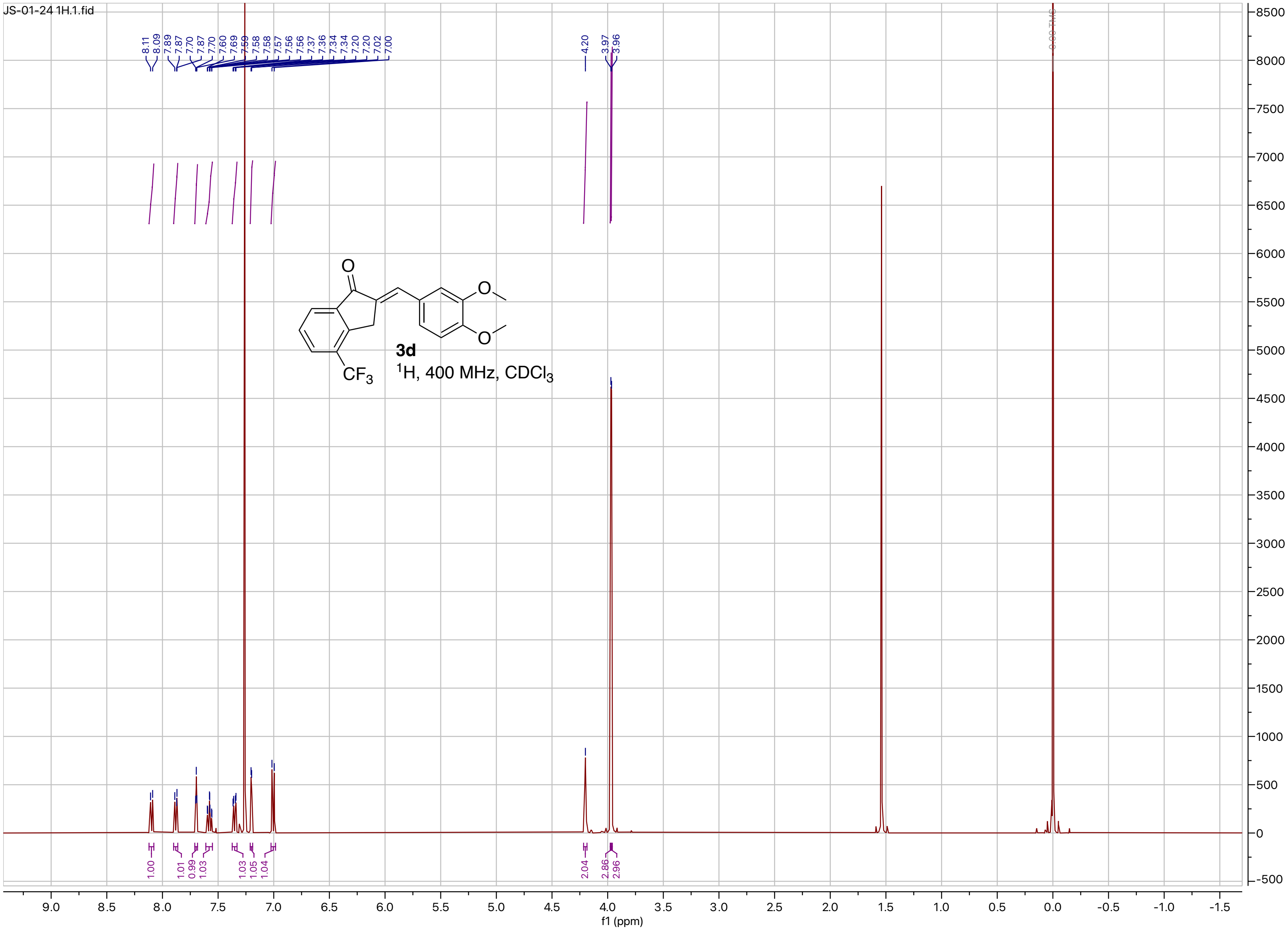
JS-01-24 1H.1.fid
3d
^{1}H, 400 MHz, CDCl$_3$
8.11
8.09
7.89
7.87
7.70
7.70
7.69
7.60
7.59
7.58
7.58
7.57
7.56
7.56
7.37
7.36
7.34
7.34
7.20
7.20
7.02
7.00
4.20
3.97
3.96
0.00 TMS
1.00
1.01
0.99
1.03
1.03
1.05
1.04
2.04
2.86
2.96
f1 (ppm)

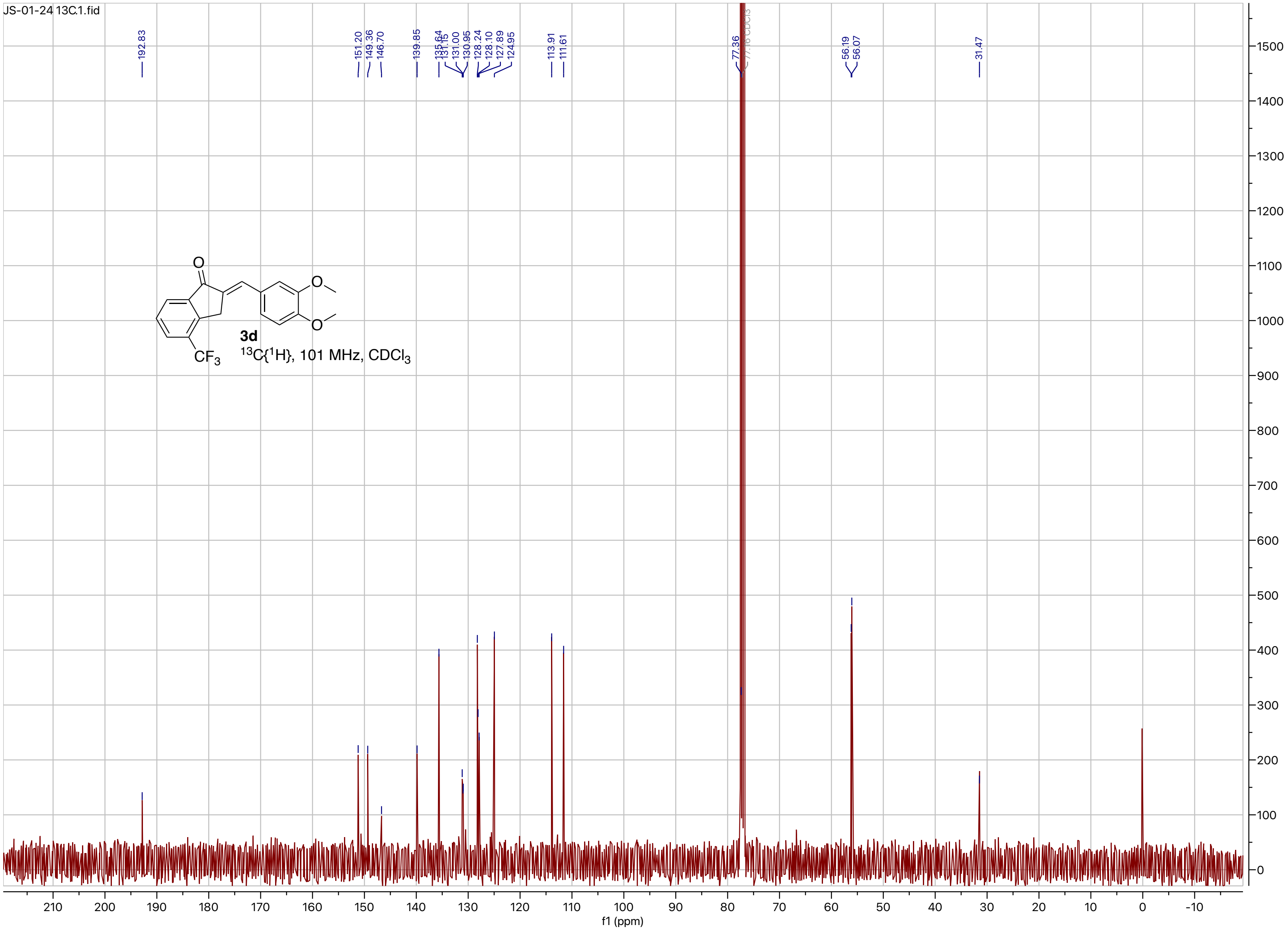
JS-01-24 13C.1.fid
192.83
151.20
149.36
146.70
139.85
135.64
131.15
131.00
130.95
128.24
128.10
127.89
124.95
113.91
111.61
77.36
77.16 CDCl3
56.19
56.07
31.47
3d
13C{1H}, 101 MHz, CDCl3
CF3
f1 (ppm)

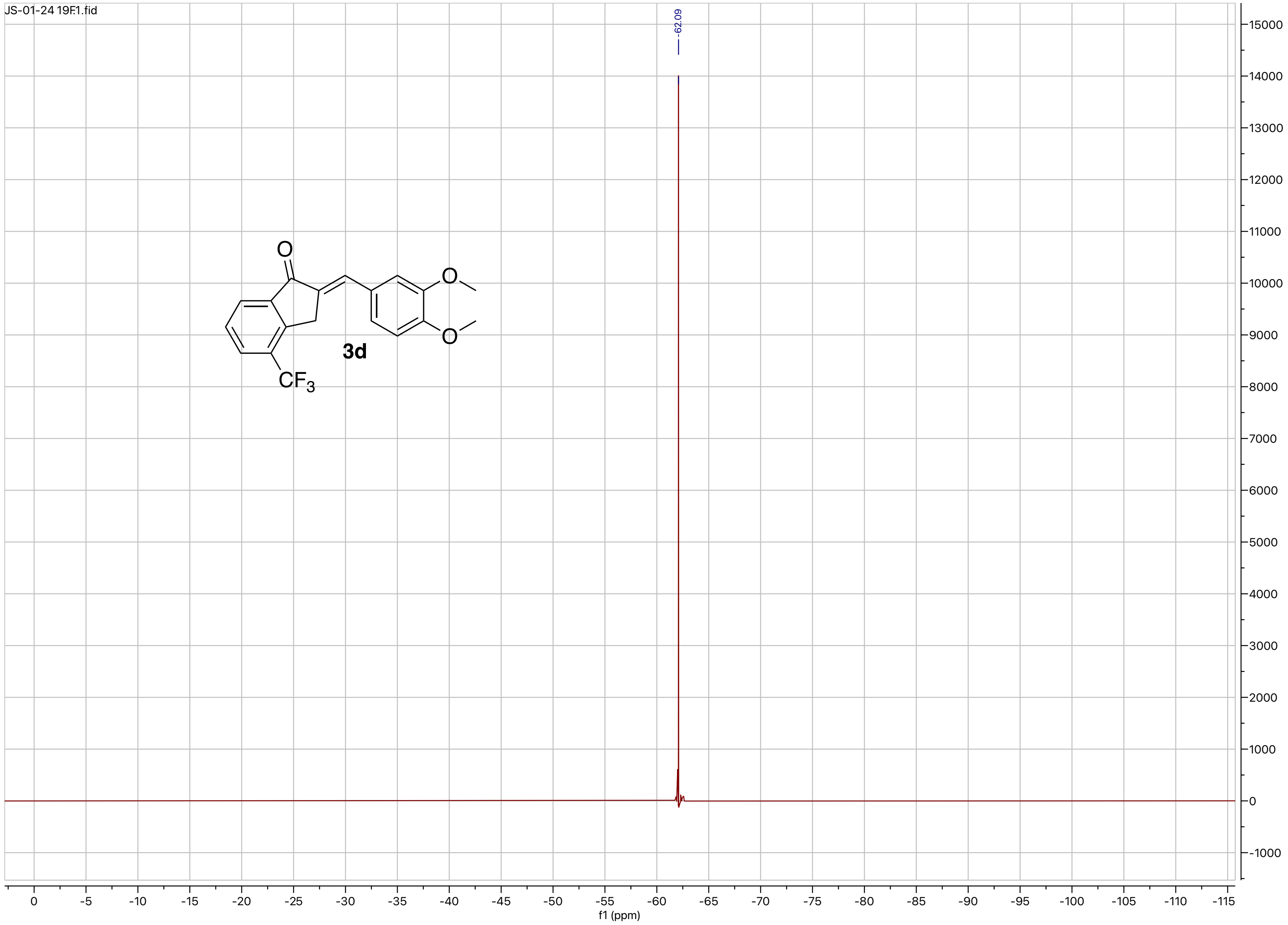

JS-01-24 19F.1.fid
-62.09
O
O
O
3d
CF3
15000
14000
13000
12000
11000
10000
9000
8000
7000
6000
5000
4000
3000
2000
1000
0
-1000
0
-5
-10
-15
-20
-25
-30
-35
-40
-45
-50
-55
-60
-65
-70
-75
-80
-85
-90
-95
-100
-105
-110
-115
f1 (ppm)

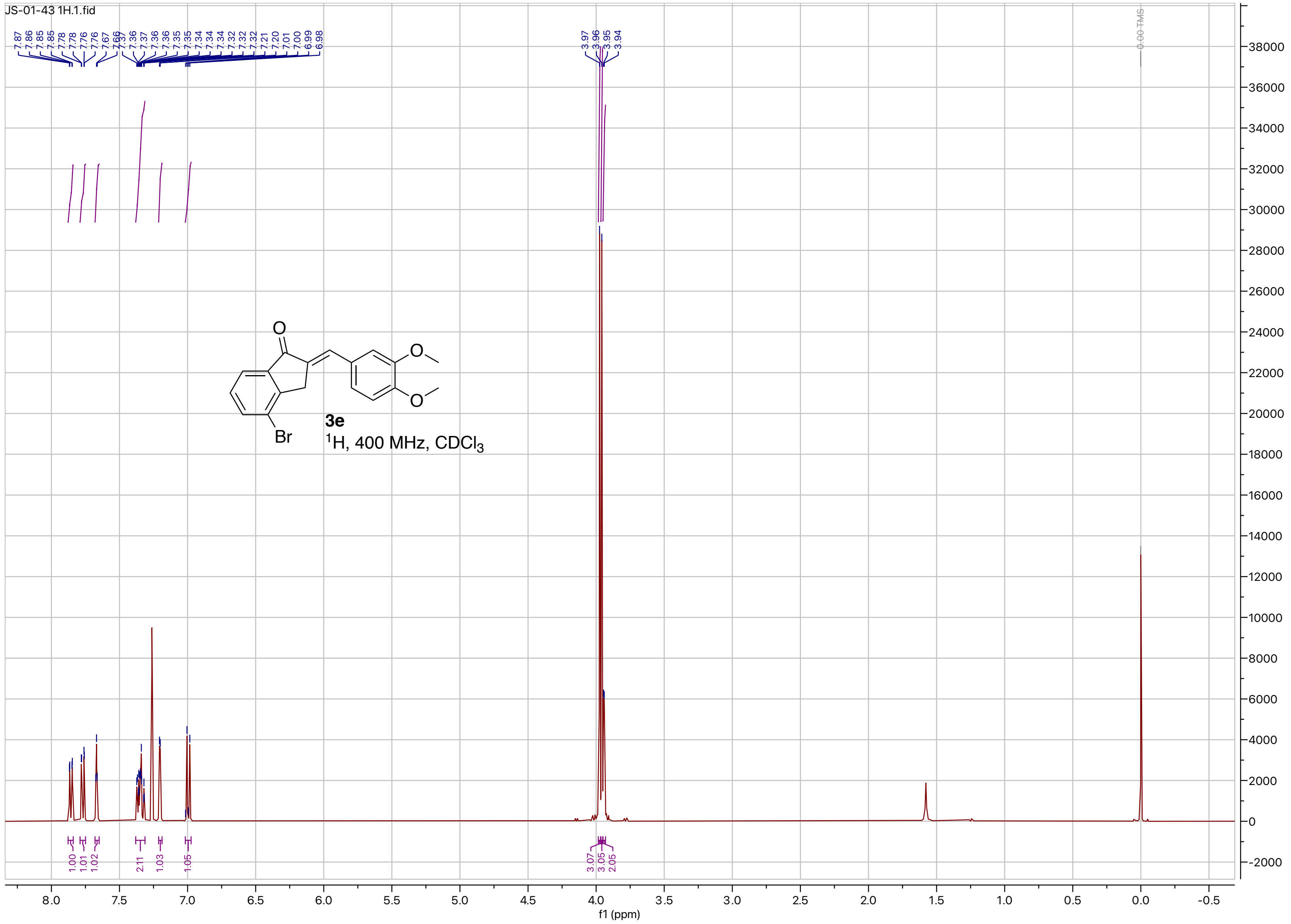
JS-01-43 1H.1.fid
3e
1H, 400 MHz, CDCl3
Br
O
f1 (ppm)

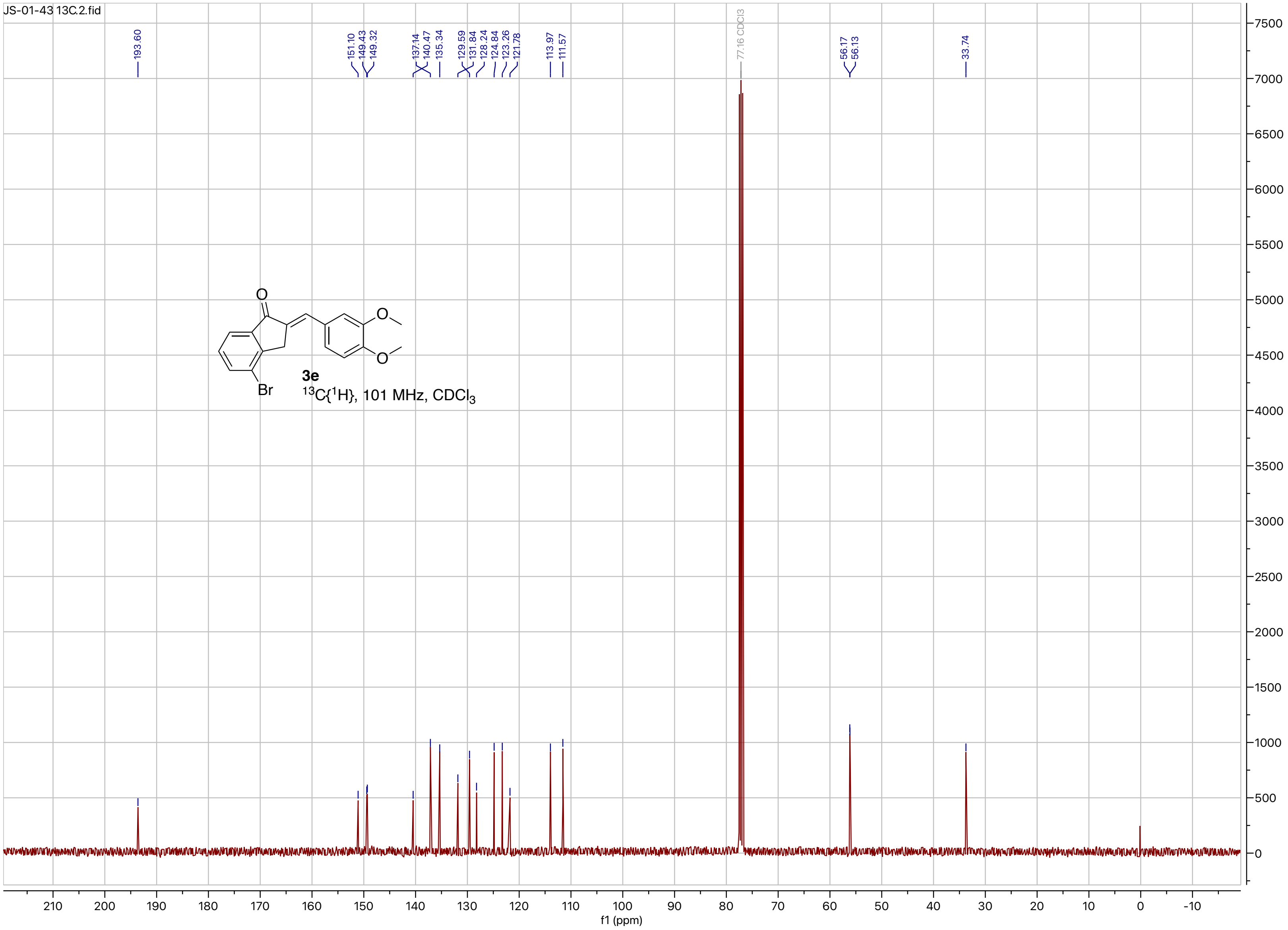
JS-01-43 13C.2.fid
193.60
151.10
149.43
149.32
137.14
140.47
135.34
129.59
131.84
128.24
124.84
123.26
121.78
113.97
111.57
77.16 CDCl3
56.17
56.13
33.74
3e
13C{1H}, 101 MHz, CDCl3
f1 (ppm)

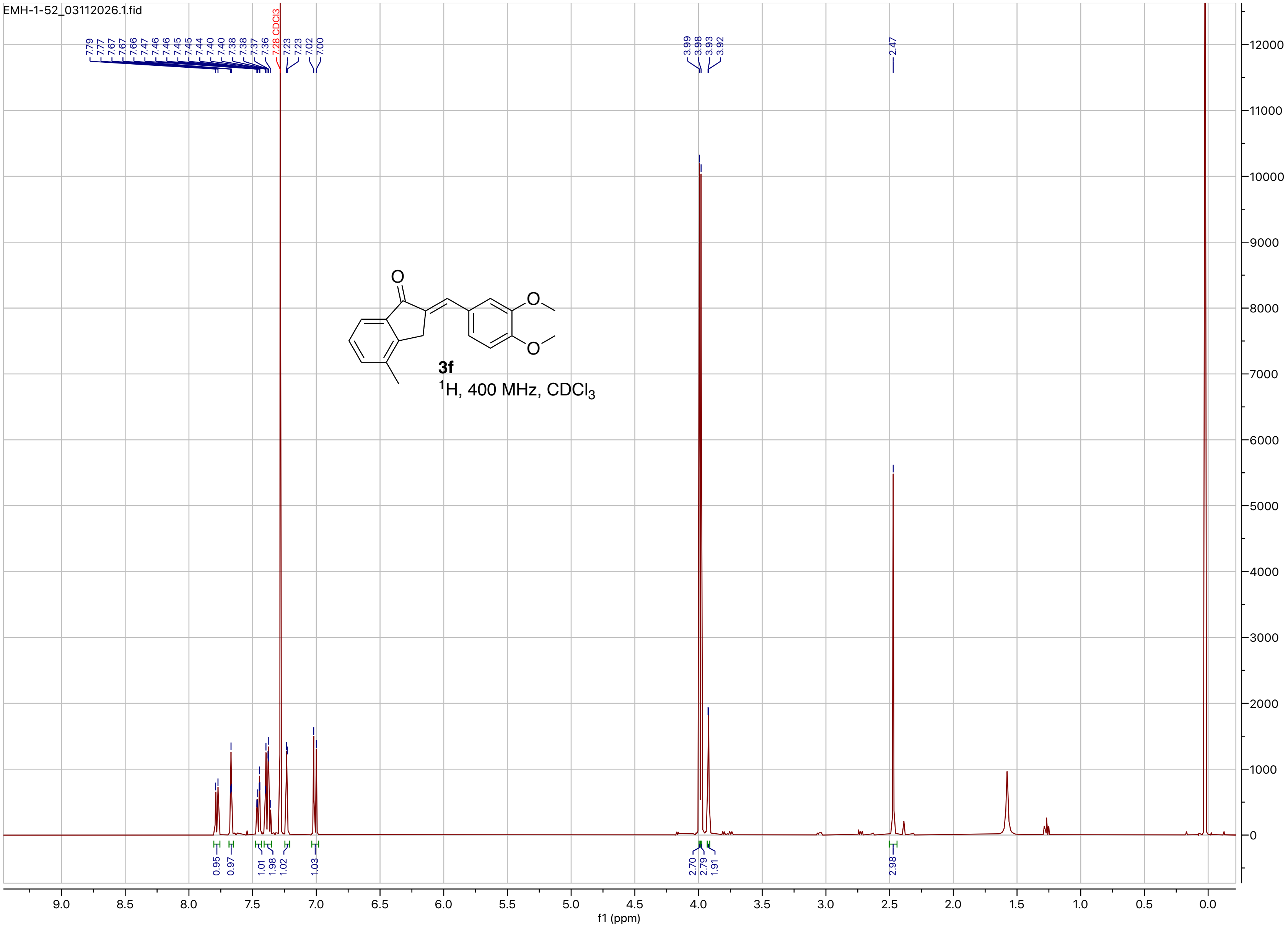
EMH-1-52_03112026.1.fid
7.79 7.77 7.67 7.67 7.66 7.47 7.46 7.45 7.45 7.44 7.40 7.40 7.38 7.38 7.37 7.36 7.28 CDCl3 7.23 7.23 7.02 7.00
3.99 3.98 3.93 3.92
2.47
3f
^{1}H, 400 MHz, CDCl$_3$
0.95 0.97 1.01 1.98 1.02 1.03 2.70 2.79 1.91 2.98
f1 (ppm)

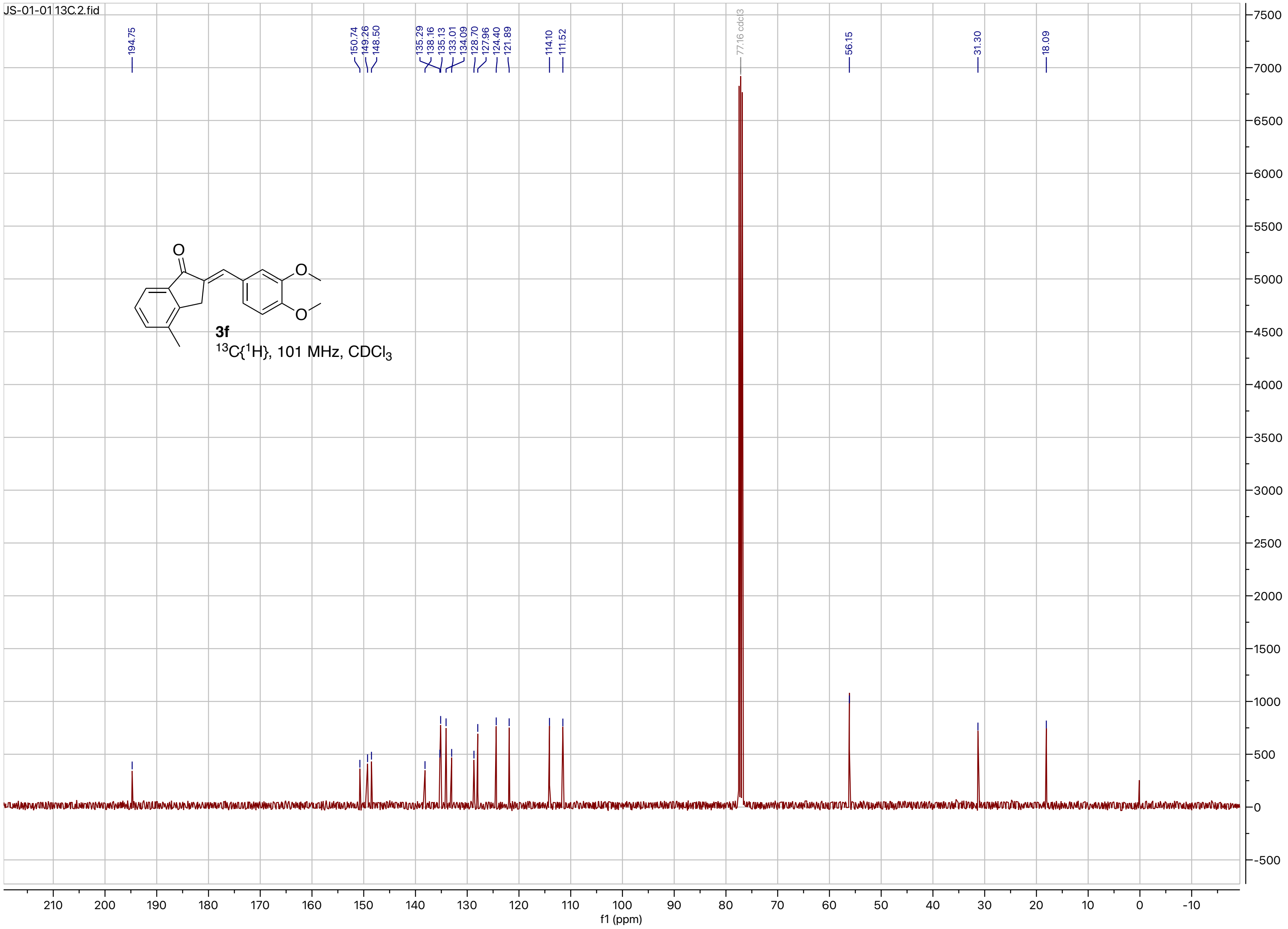

JS-01-01 13C.2.fid
194.75
150.74
149.26
148.50
135.29
138.16
135.13
133.01
134.09
128.70
127.96
124.40
121.89
114.10
111.52
77.16 cdcl3
56.15
31.30
18.09
3f
$^{13}C\{^1H\}$, 101 MHz, $CDCl_3$
f1 (ppm)

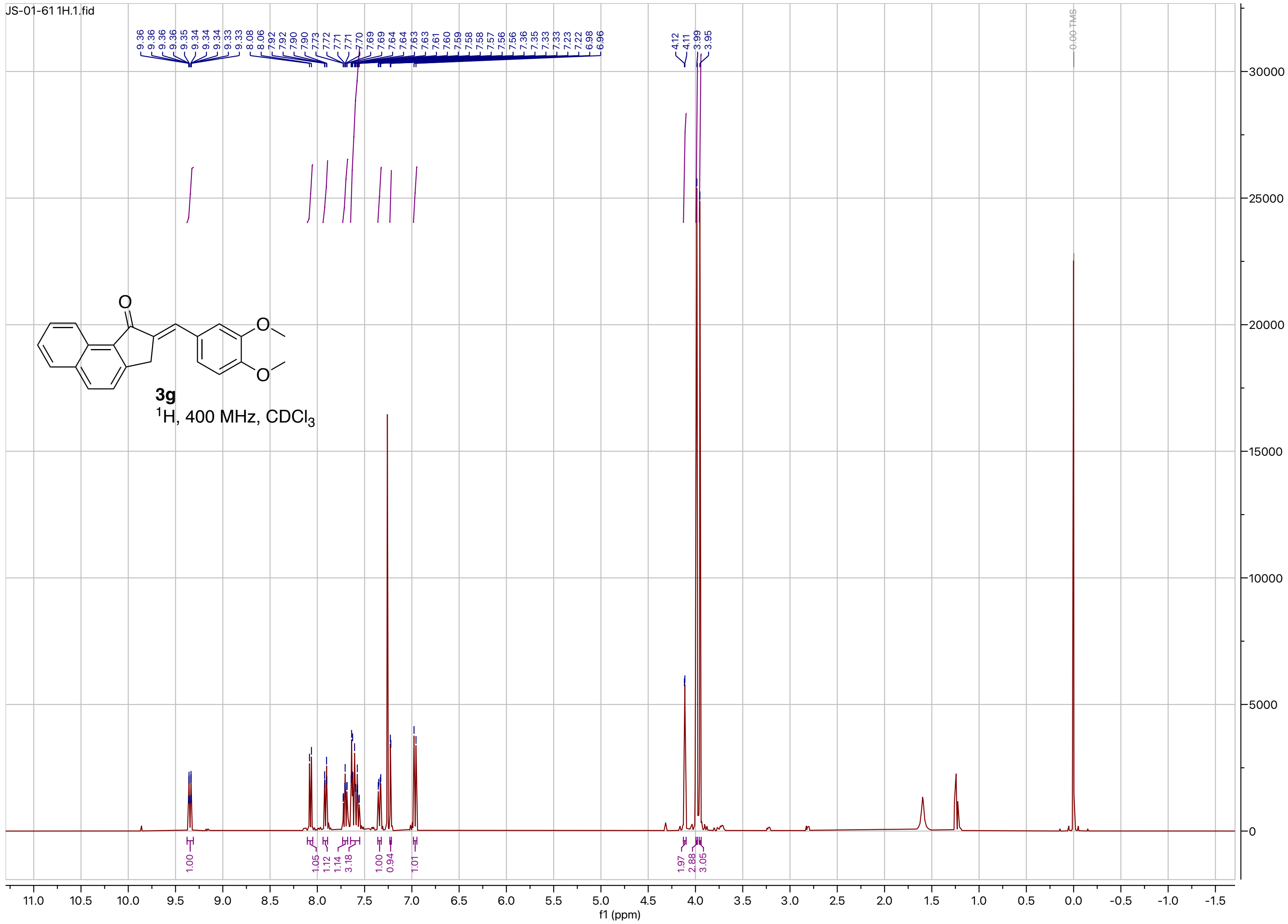

JS-01-61 1H.1.fid
3g
^{1}H, 400 MHz, $CDCl_3$
9.36 9.36 9.36 9.36 9.35 9.34 9.34 9.34 9.33 9.33 8.08 8.06 7.92 7.92 7.90 7.90 7.73 7.72 7.71 7.70 7.69 7.69 7.64 7.64 7.63 7.63 7.61 7.60 7.59 7.58 7.58 7.57 7.56 7.56 7.36 7.35 7.33 7.33 7.23 7.22 6.98 6.96
4.12 4.11 3.99 3.95
0.00 TMS
1.00 1.05 1.12 1.14 3.18 1.00 0.94 1.01 1.97 2.88 3.05
f1 (ppm)

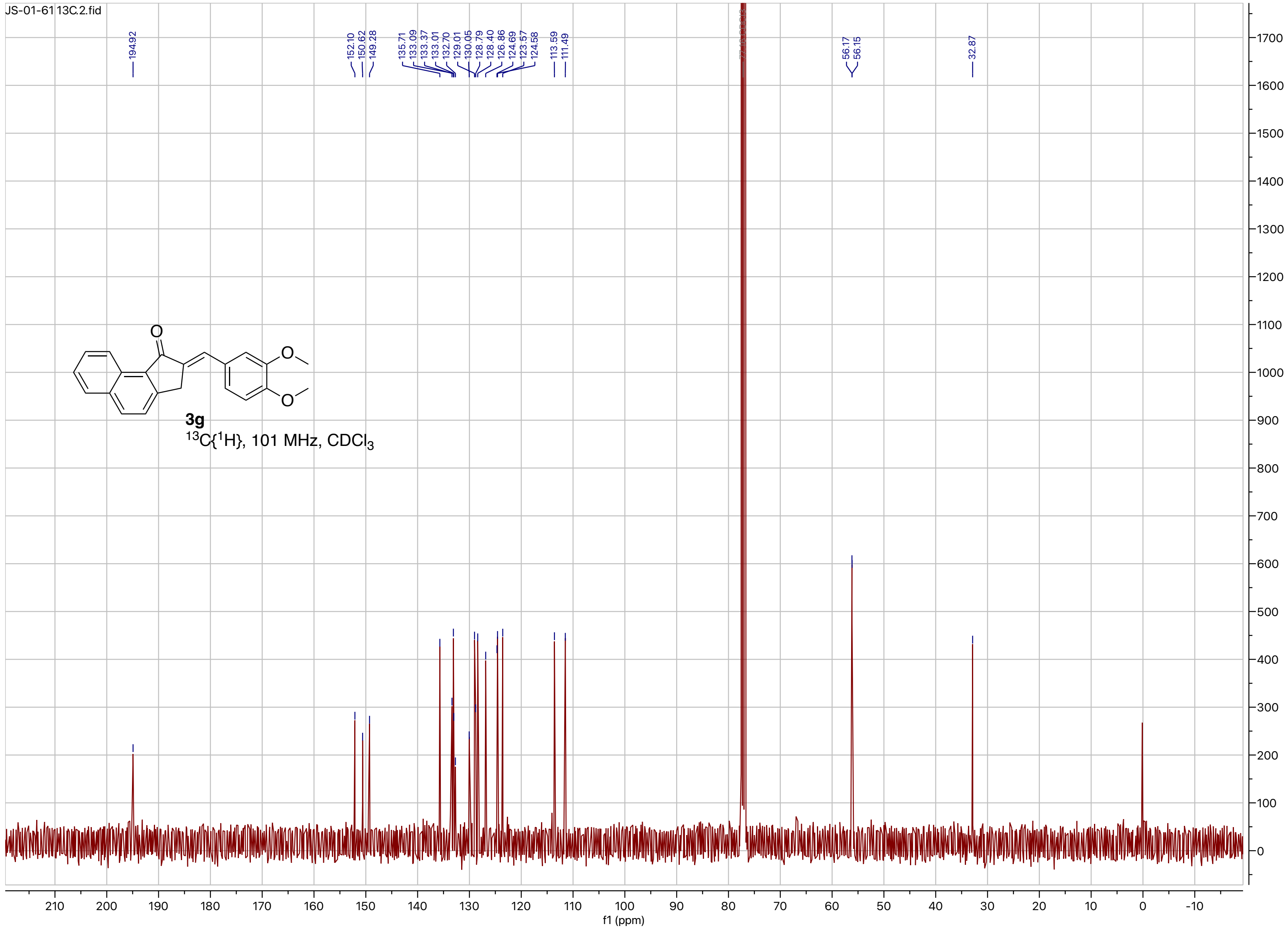

JS-01-61 13C.2.fid
194.92
152.10
150.62
149.28
135.71
133.09
133.37
133.01
132.70
129.01
130.05
128.79
128.40
126.86
124.69
123.57
124.58
113.59
111.49
56.17
56.15
32.87
3g
$^{13}C\{^{1}H\}$, 101 MHz, $CDCl_3$
f1 (ppm)

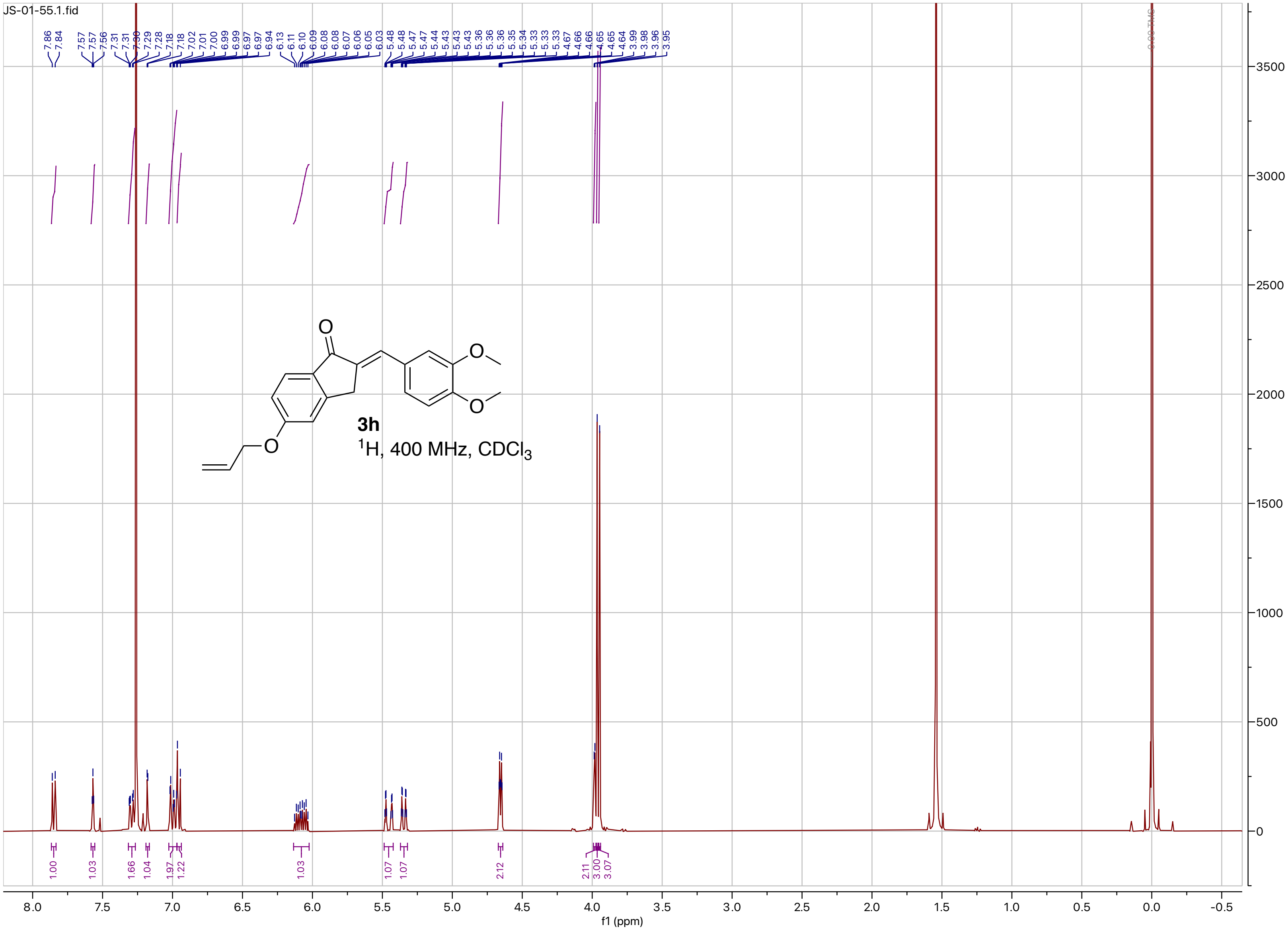

JS-01-55.1.fid
3h
1H, 400 MHz, CDCl3
f1 (ppm)

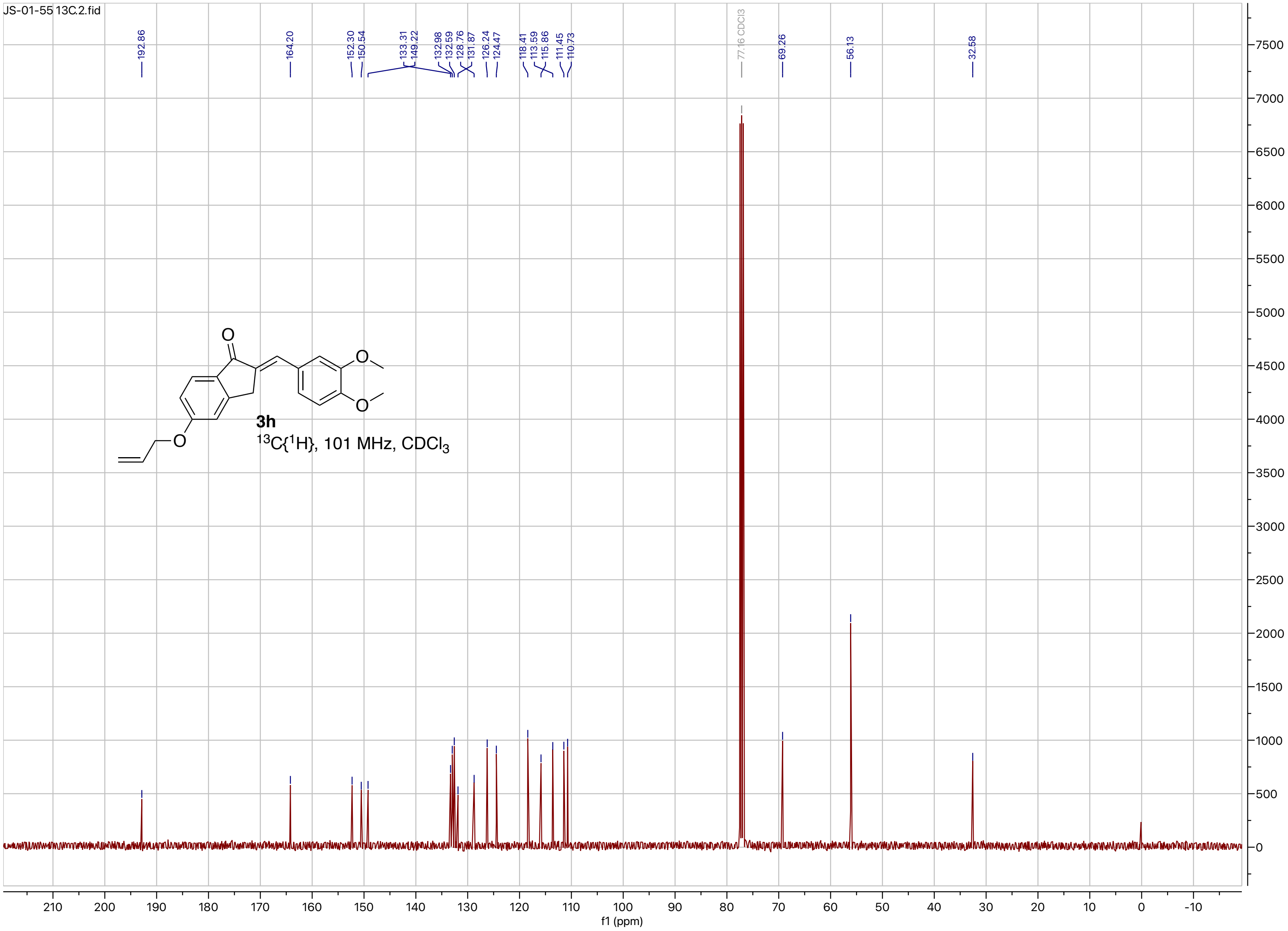
JS-01-55 13C.2.fid
192.86
164.20
152.30
150.54
133.31
149.22
132.98
132.59
128.76
131.87
126.24
124.47
118.41
113.59
115.86
111.45
110.73
77.16 CDCl3
69.26
56.13
32.58
O
O
O
O
3h
$^{13}C\{^{1}H\}$, 101 MHz, $CDCl_3$
f1 (ppm)

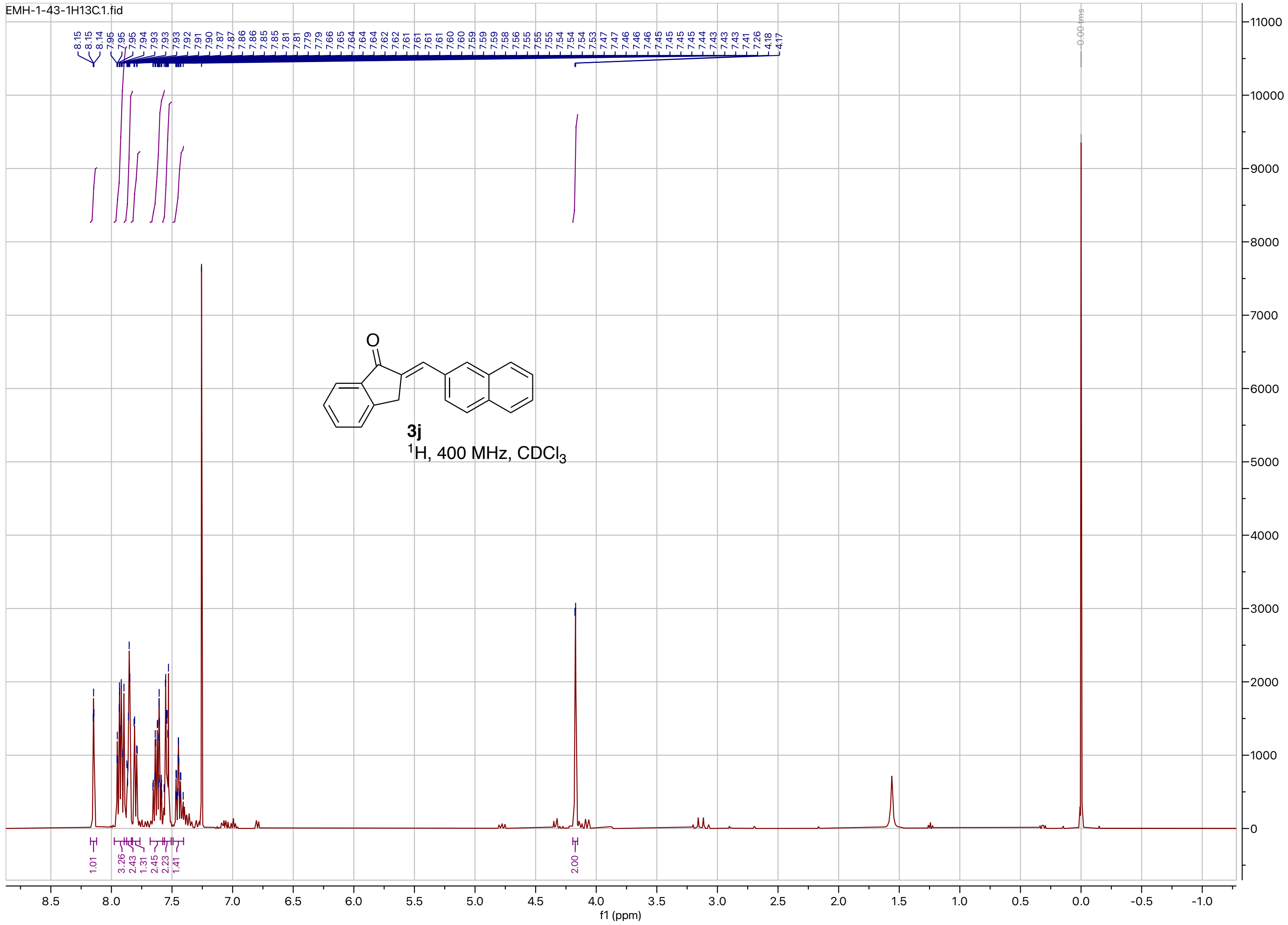
EMH-1-43-1H13C.1.fid
3j
1H, 400 MHz, CDCl3
f1 (ppm)

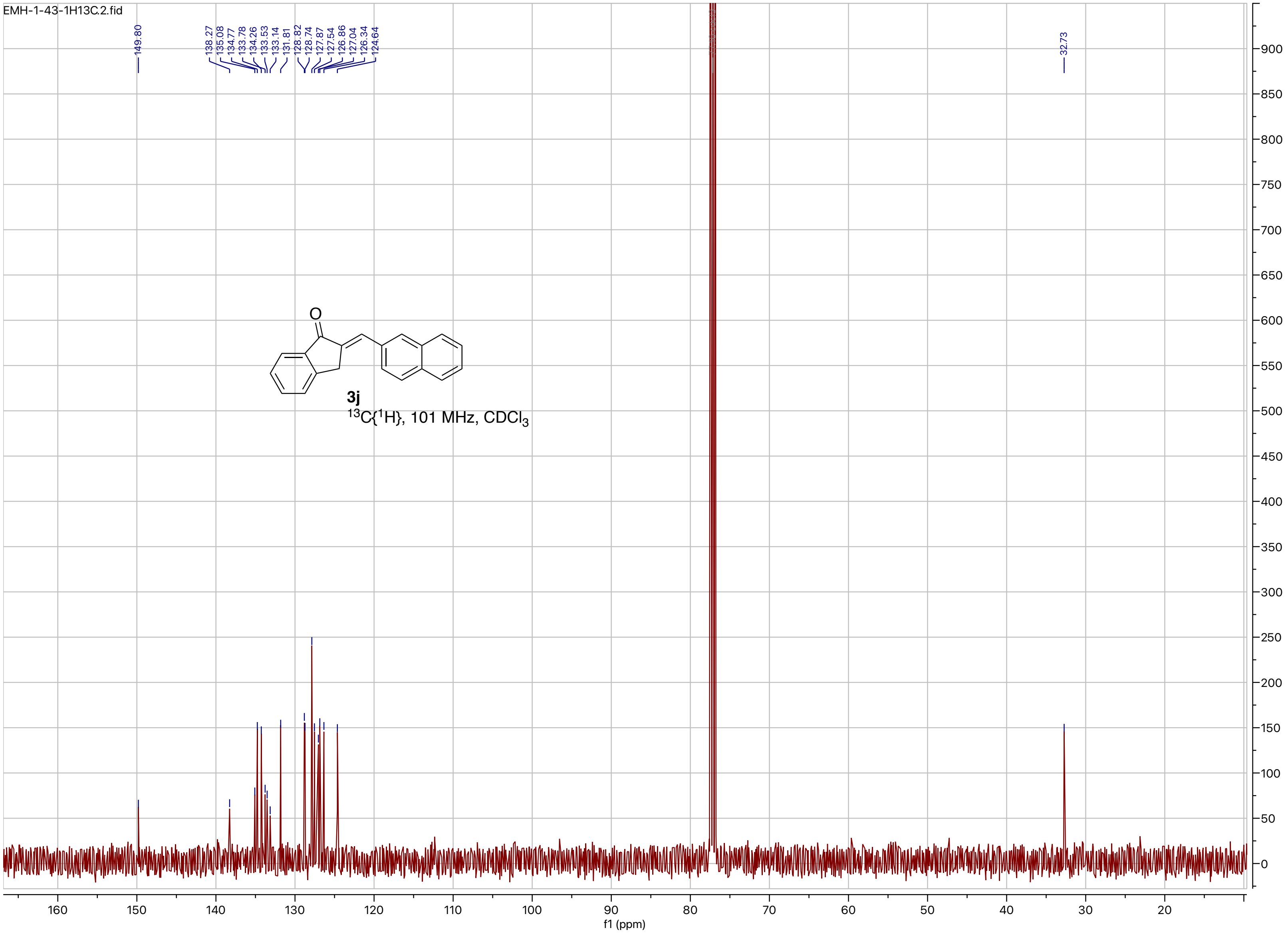

EMH-1-43-1H13C.2.fid
149.80
138.27
135.08
134.77
133.78
134.26
133.53
133.14
131.81
128.82
128.74
127.87
127.54
126.86
127.04
126.34
124.64
32.73
3j
$^{13}C\{^1H\}$, 101 MHz, $CDCl_3$
f1 (ppm)

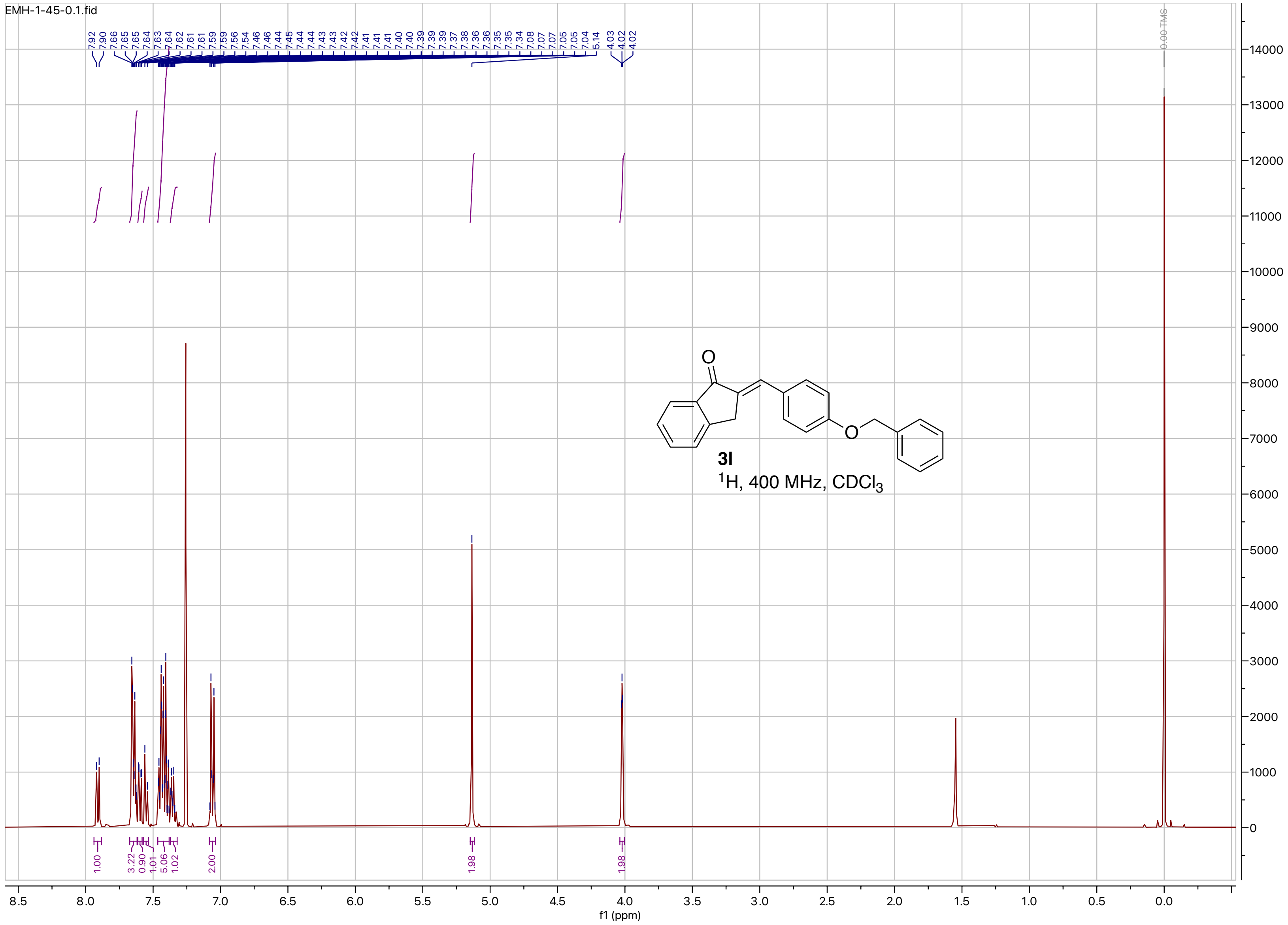
EMH-1-45-0.1.fid
3l
1H, 400 MHz, CDCl3
f1 (ppm)

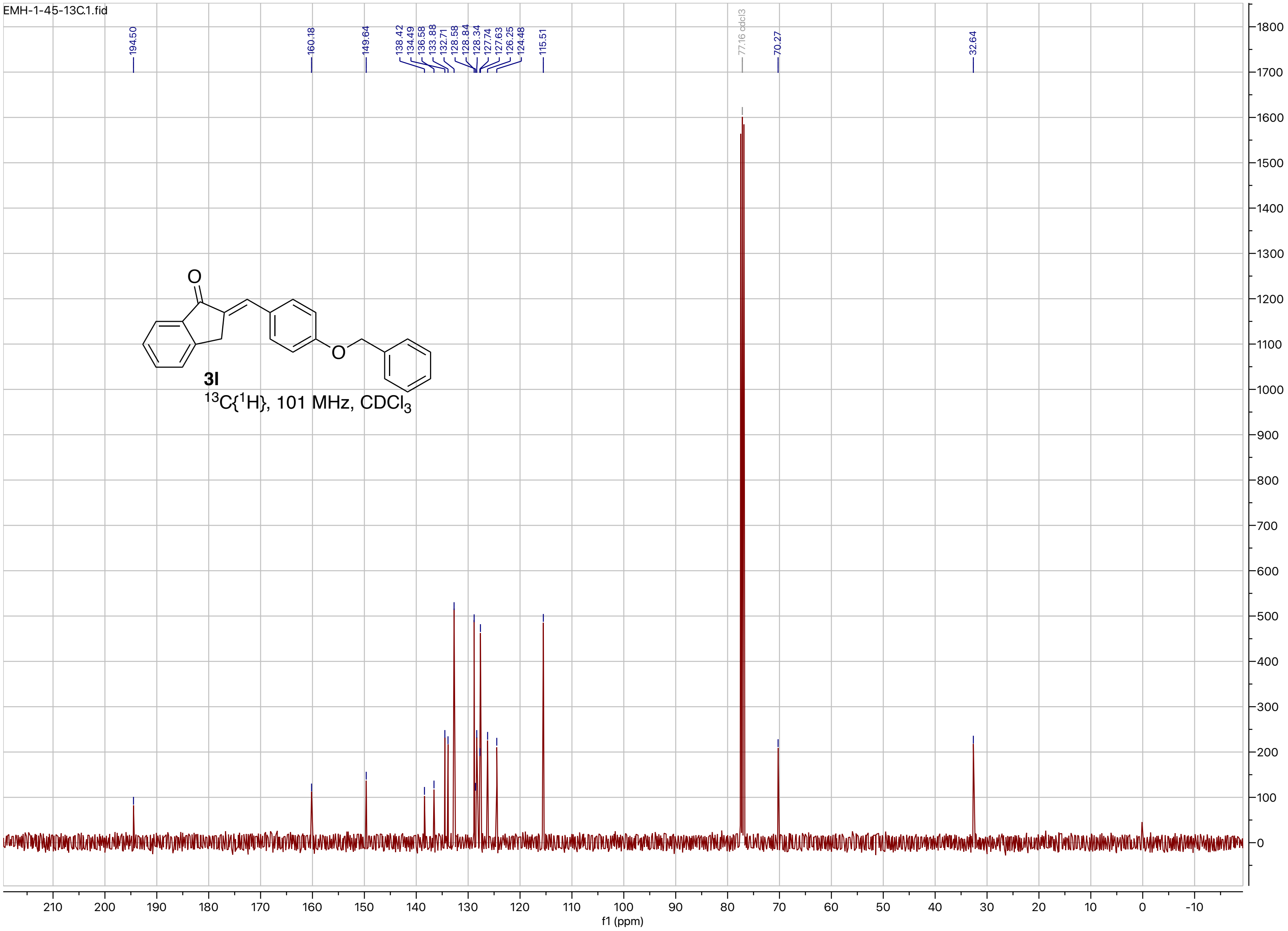
EMH-1-45-13C1.fid
194.50
160.18
149.64
138.42
134.49
136.58
133.88
132.71
128.58
128.84
128.34
127.74
127.63
126.25
124.48
115.51
77.16 cdcl3
70.27
32.64
O
O
3l
$^{13}C\{^1H\}$, 101 MHz, $CDCl_3$
f1 (ppm)

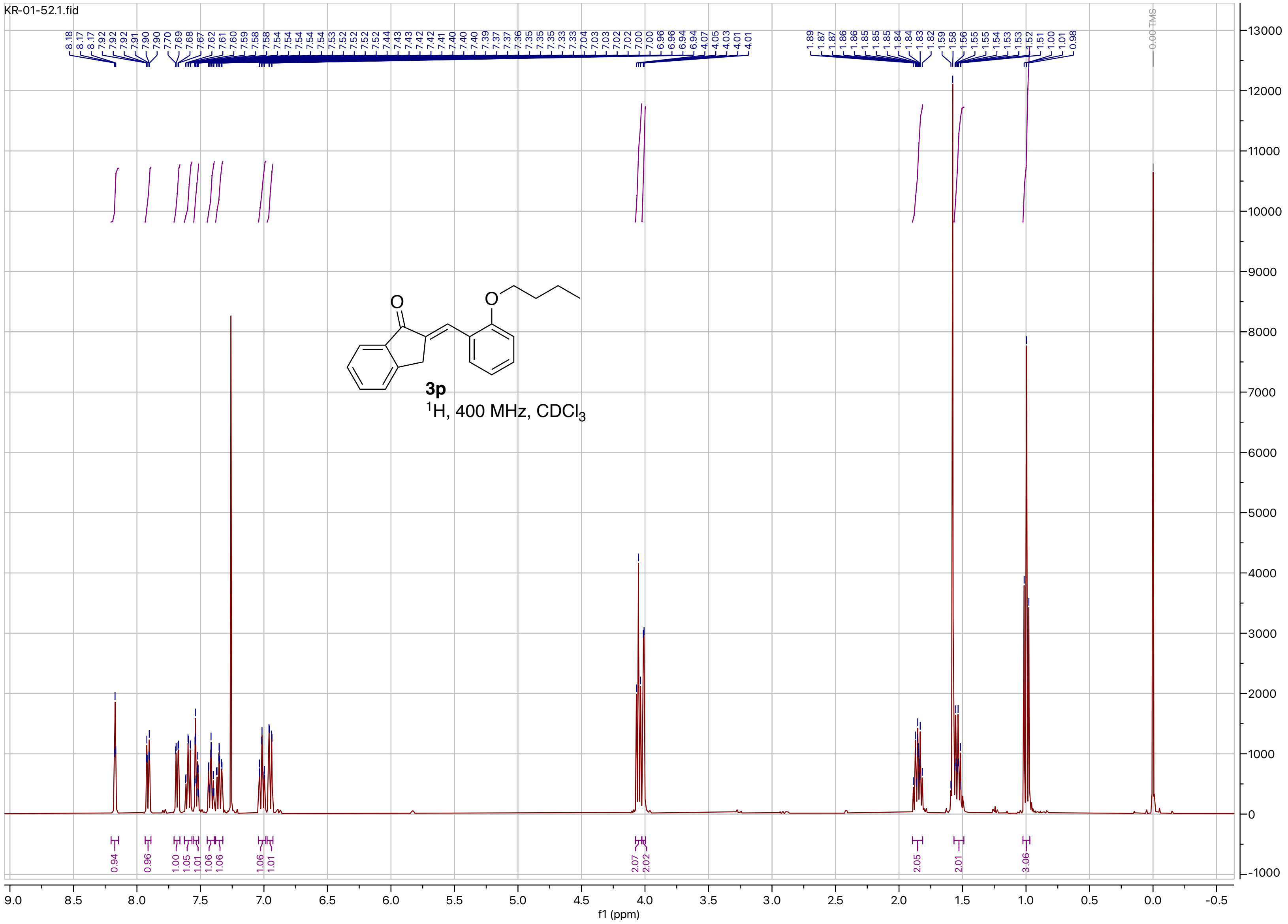
KR-01-52.1.fid
O
O
3p
^{1}H, 400 MHz, CDCl$_3$
0.00 TMS
f1 (ppm)

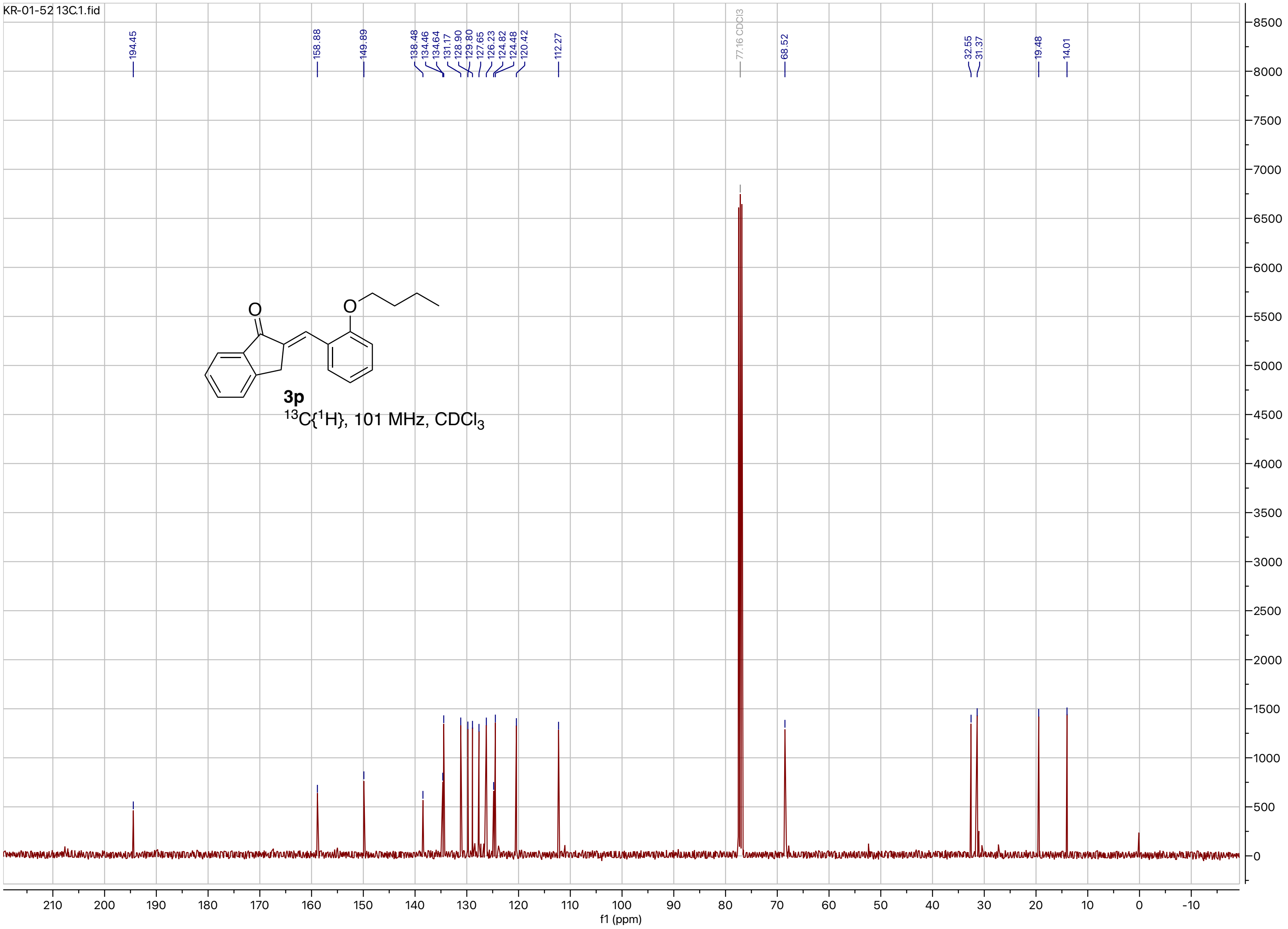
KR-01-52 13C1.fid
194.45
158.88
149.89
138.48
134.46
134.64
131.17
128.90
129.80
127.65
126.23
124.82
124.48
120.42
112.27
77.16 CDCl3
68.52
32.55
31.37
19.48
14.01
3p
13C{1H}, 101 MHz, CDCl3
f1 (ppm)

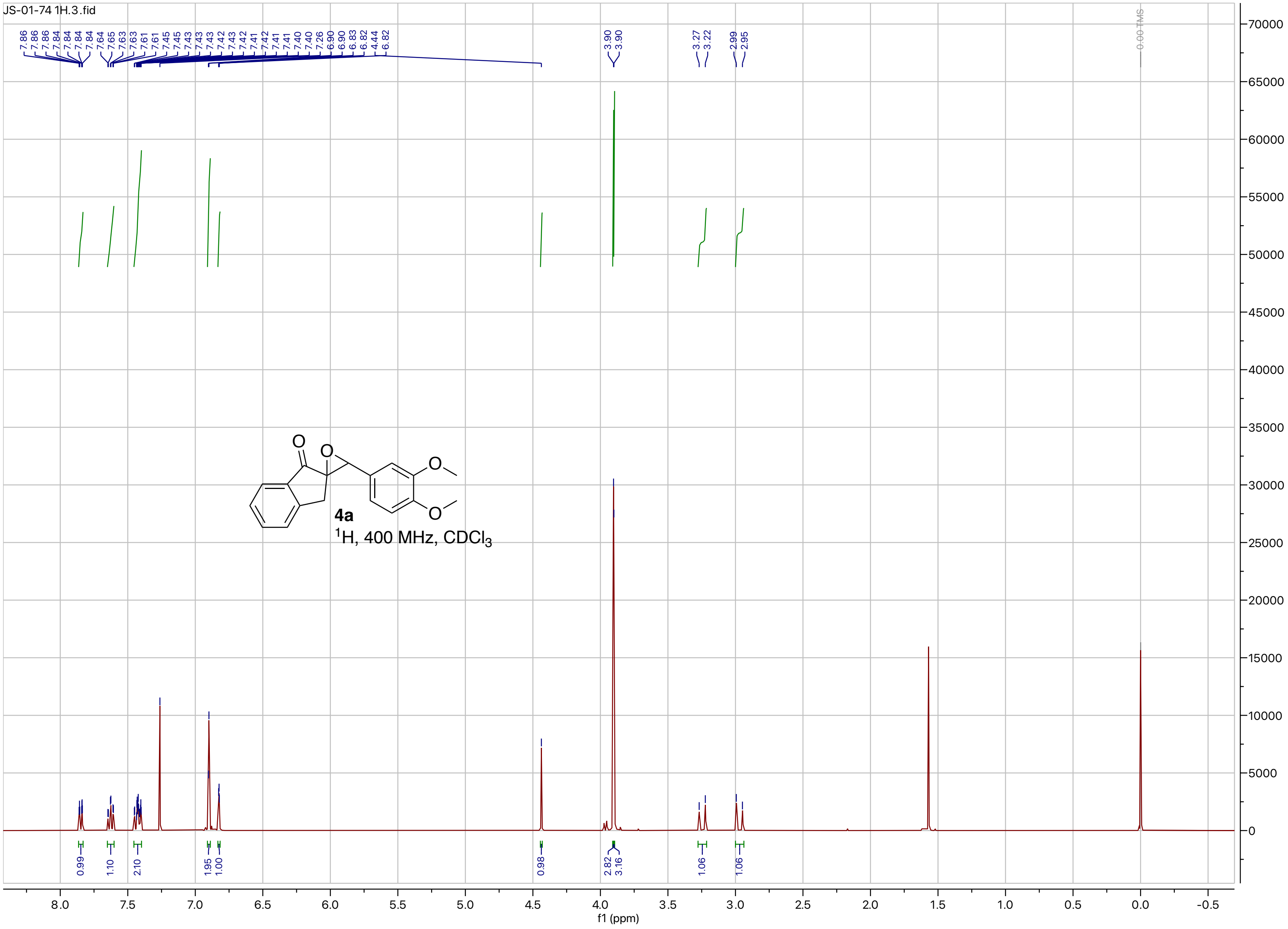
JS-01-74 1H.3.fid
7.86
7.86
7.86
7.84
7.84
7.84
7.84
7.64
7.65
7.63
7.63
7.61
7.61
7.45
7.45
7.43
7.43
7.43
7.42
7.43
7.42
7.42
7.41
7.41
7.41
7.40
7.40
7.26
6.90
6.90
6.83
6.82
4.44
6.82
3.90
3.90
3.27
3.22
2.99
2.95
0.00 TMS
O
O
O
O
4a
1H, 400 MHz, CDCl3
0.99
1.10
2.10
1.95
1.00
0.98
2.82
3.16
1.06
1.06
f1 (ppm)

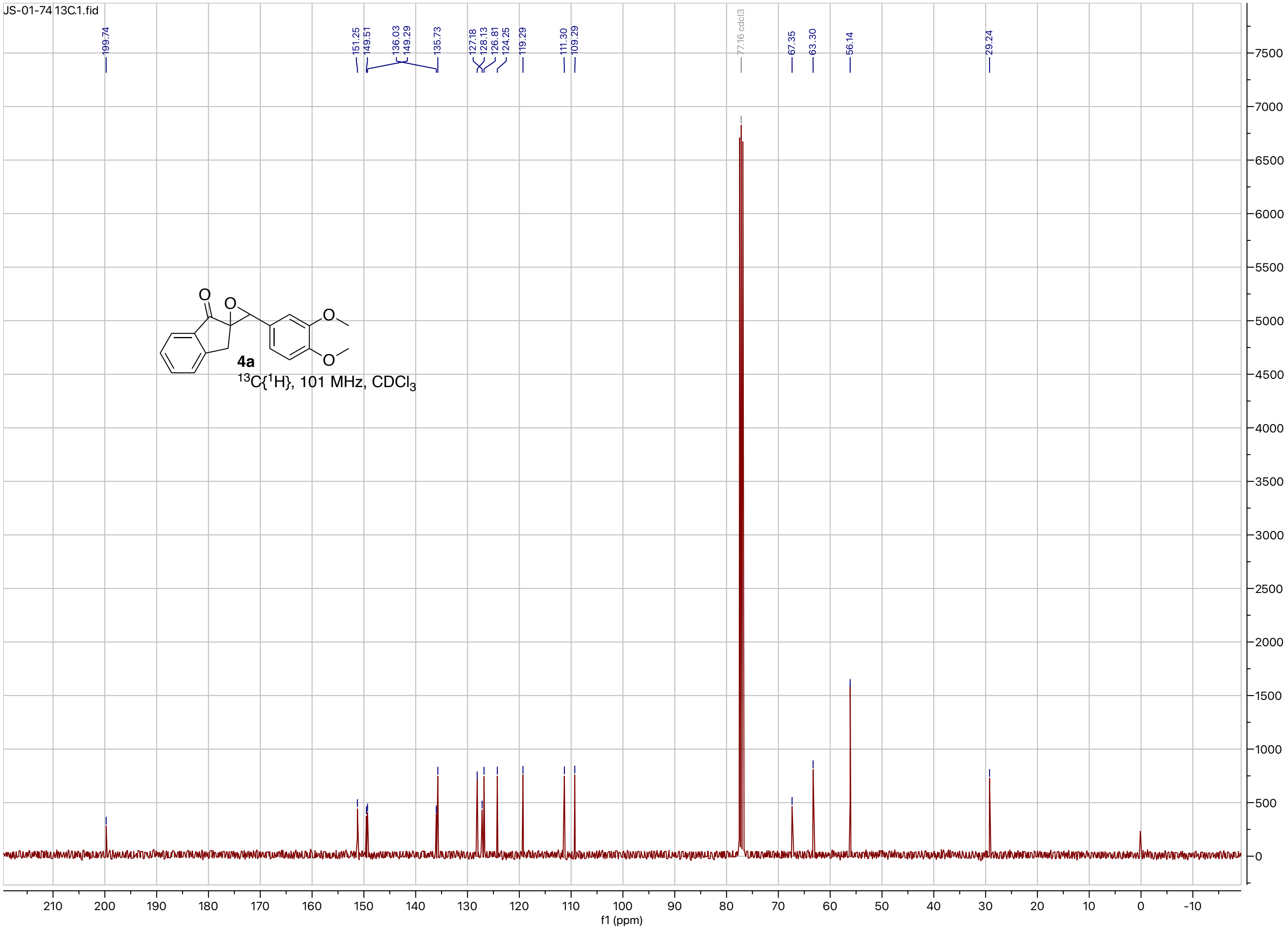
JS-01-74 13C.1.fid
199.74
151.25
149.51
136.03
149.29
135.73
127.18
128.13
126.81
124.25
119.29
111.30
109.29
77.16 cdcl3
67.35
63.30
56.14
29.24
4a
$^{13}C\{^1H\}$, 101 MHz, $CDCl_3$
f1 (ppm)

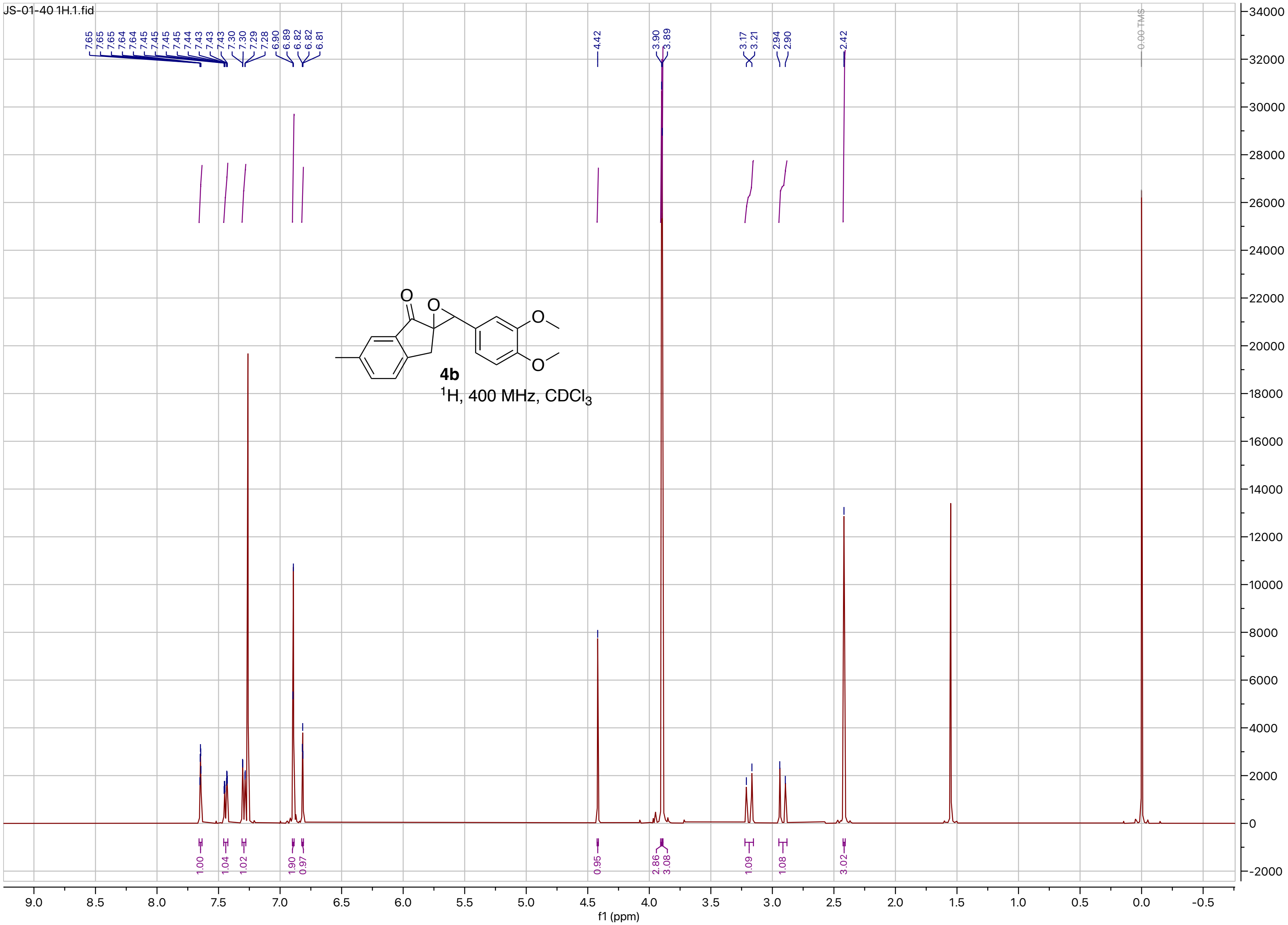
JS-01-40 1H.1.fid
4b
^{1}H, 400 MHz, CDCl$_3$
f1 (ppm)

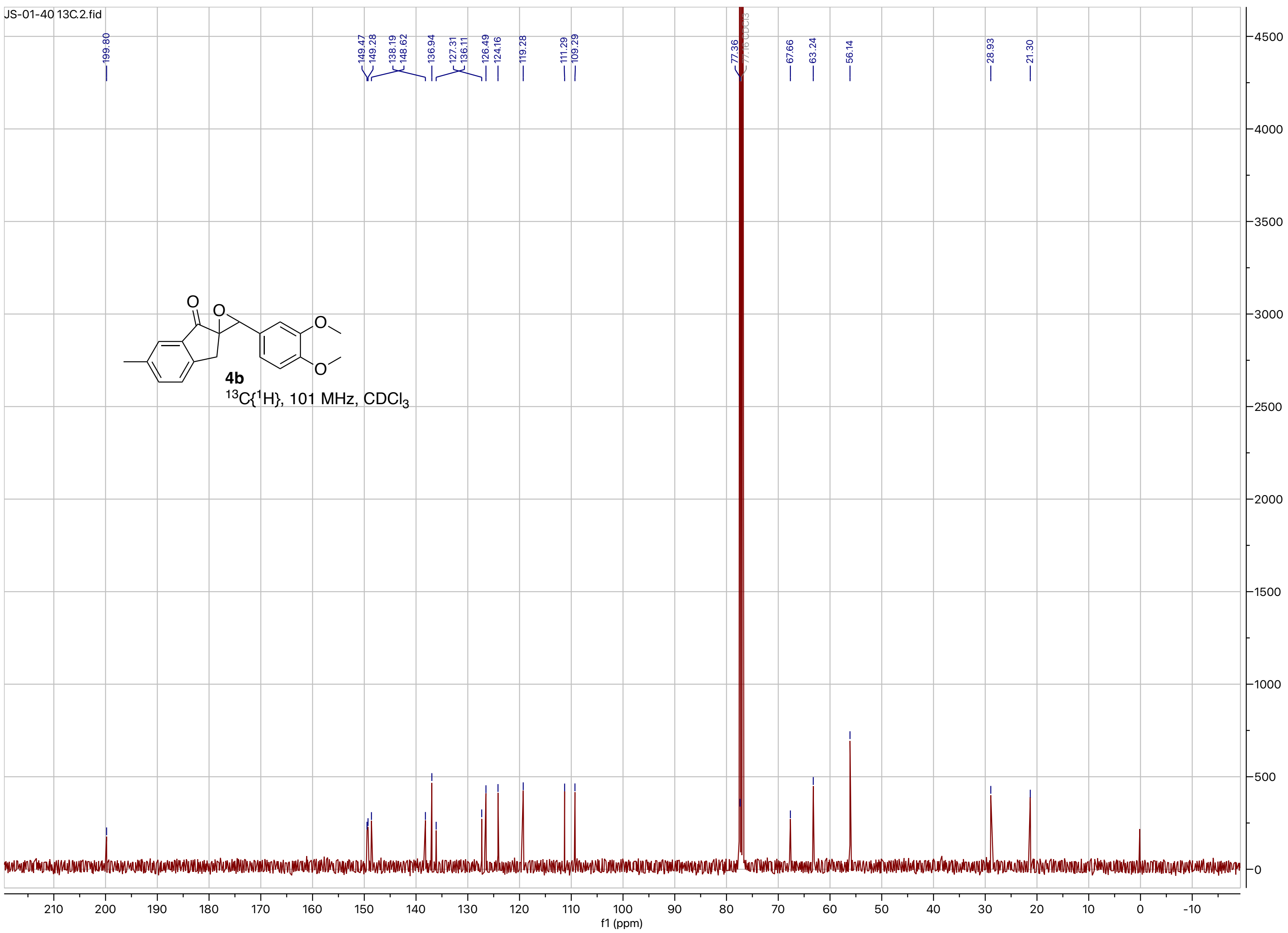
JS-01-40 13C.2.fid
199.80
149.47
149.28
138.19
148.62
136.94
127.31
136.11
126.49
124.16
119.28
111.29
109.29
77.36
77.16 CDCl3
67.66
63.24
56.14
28.93
21.30
4b
$^{13}C\{^1H\}$, 101 MHz, $CDCl_3$
f1 (ppm)

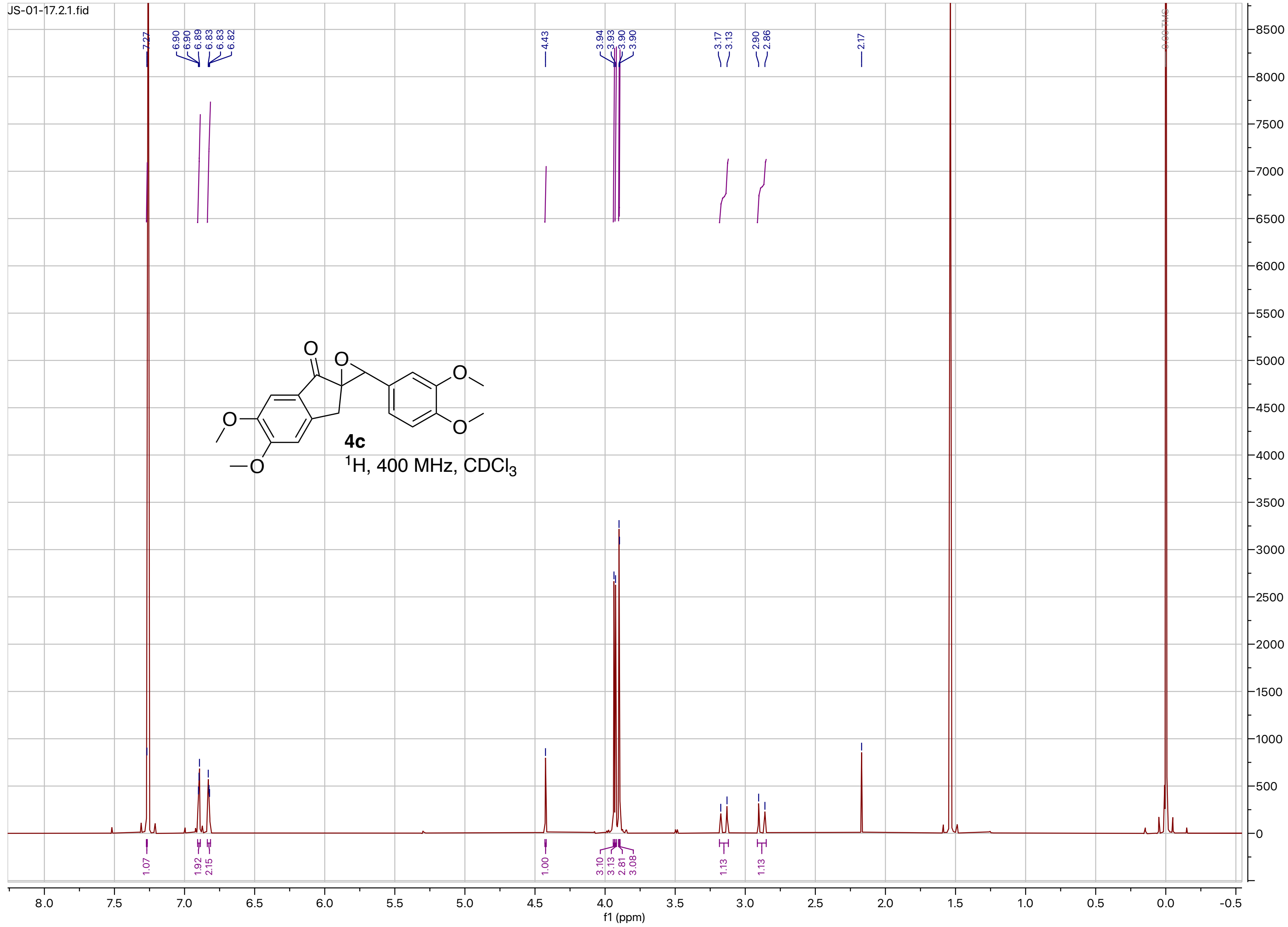

JS-01-17.2.1.fid
7.27
6.90
6.90
6.89
6.83
6.83
6.82
4.43
3.94
3.93
3.90
3.90
3.17
3.13
2.90
2.86
2.17
0.00 TMS
4c
^{1}H, 400 MHz, $CDCl_3$
1.07
1.92
2.15
1.00
3.10
3.13
2.81
3.08
1.13
1.13
f1 (ppm)

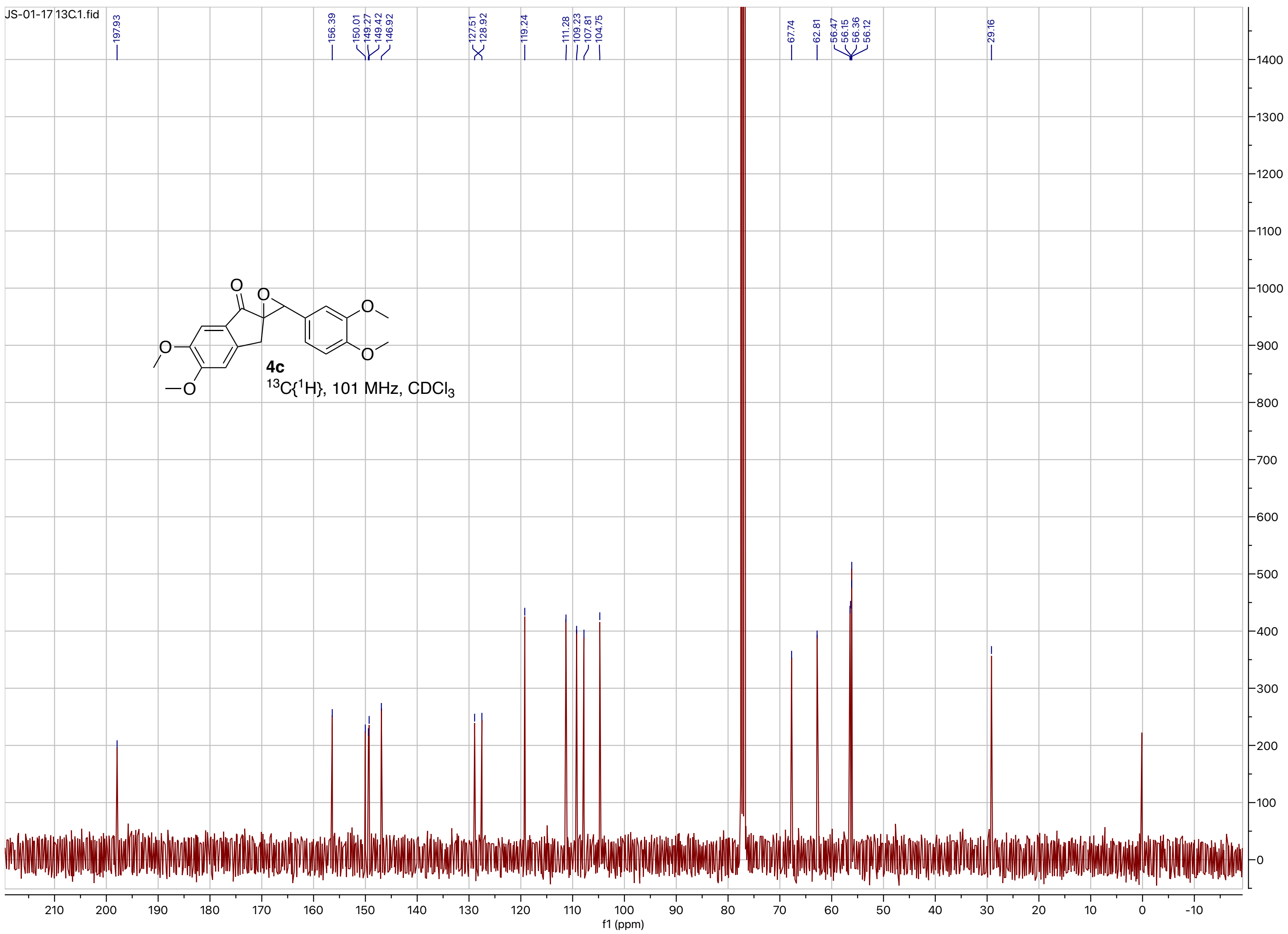
JS-01-17 13C1.fid
197.93
156.39
150.01
149.27
149.42
146.92
127.51
128.92
119.24
111.28
109.23
107.81
104.75
67.74
62.81
56.47
56.15
56.36
56.12
29.16
4c
13C{1H}, 101 MHz, CDCl3
f1 (ppm)

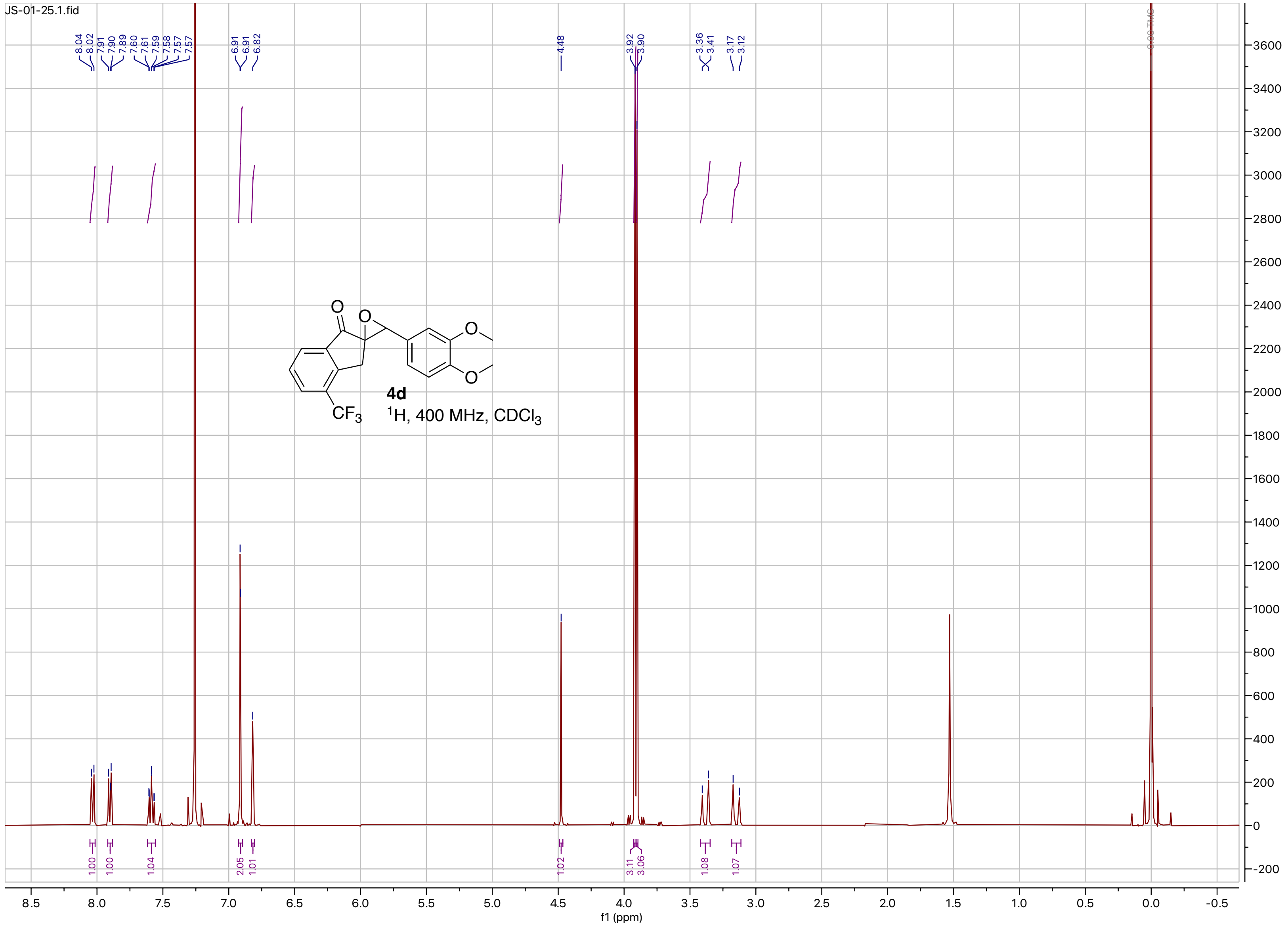
JS-01-25.1.fid
4d
1H, 400 MHz, CDCl3
CF3
f1 (ppm)

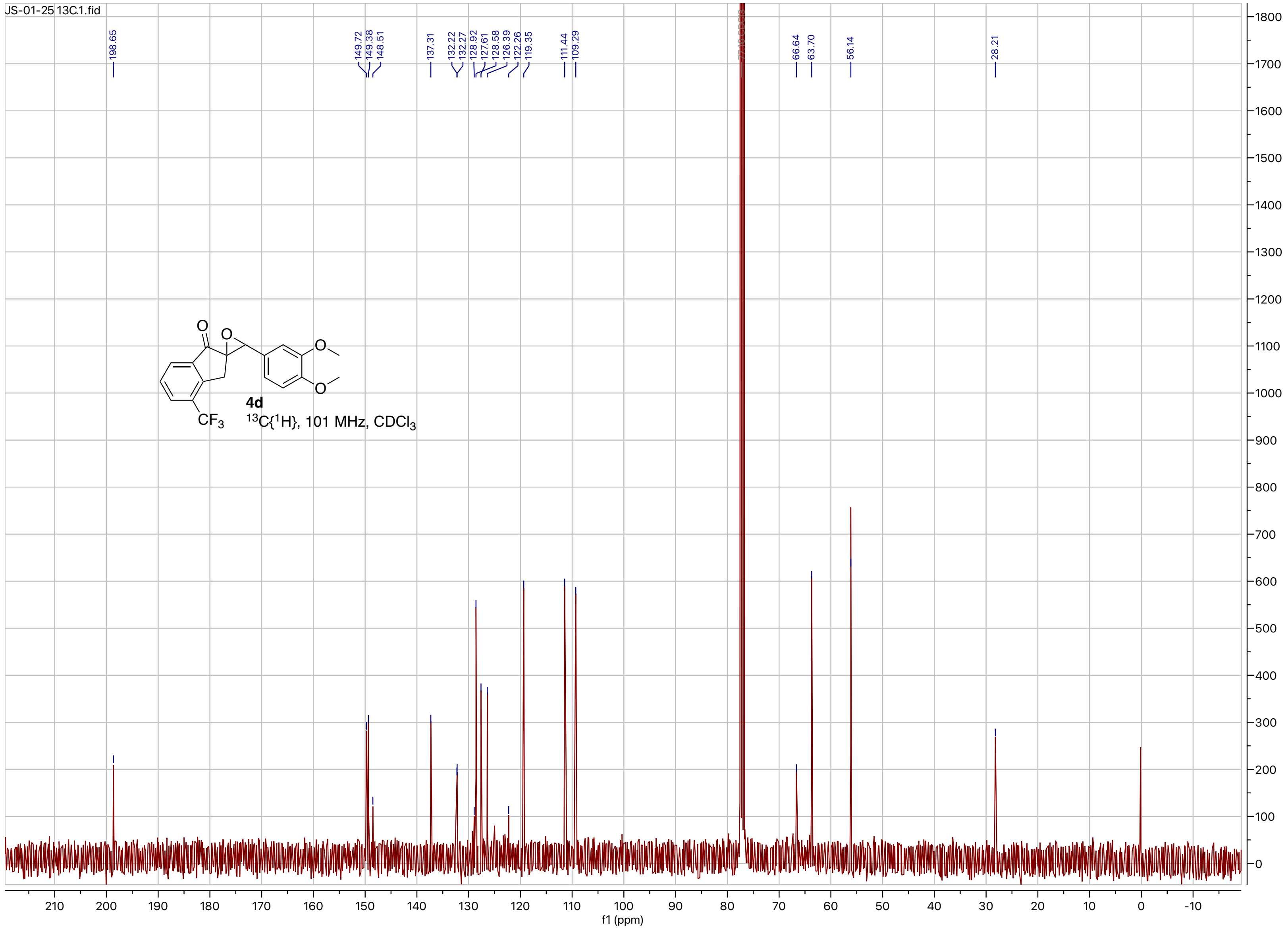

JS-01-25 13C.1.fid
198.65
149.72
149.38
148.51
137.31
132.22
132.27
128.92
127.61
128.58
126.39
122.26
119.35
111.44
109.29
66.64
63.70
56.14
28.21
4d
CF3
$^{13}C\{^{1}H\}$, 101 MHz, $CDCl_3$
f1 (ppm)

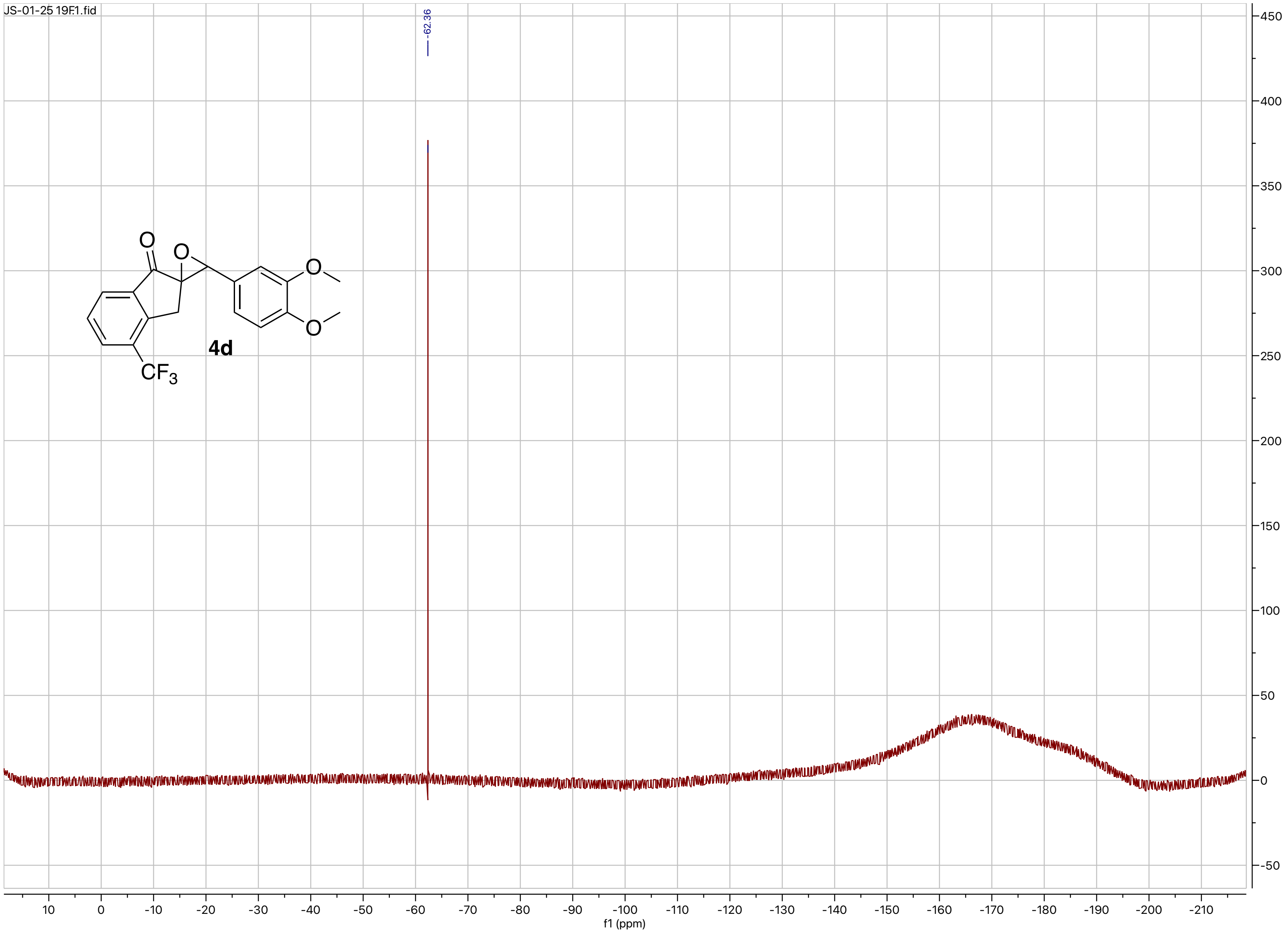
JS-01-25 19F1.fid
-62.36
4d
CF3
f1 (ppm)

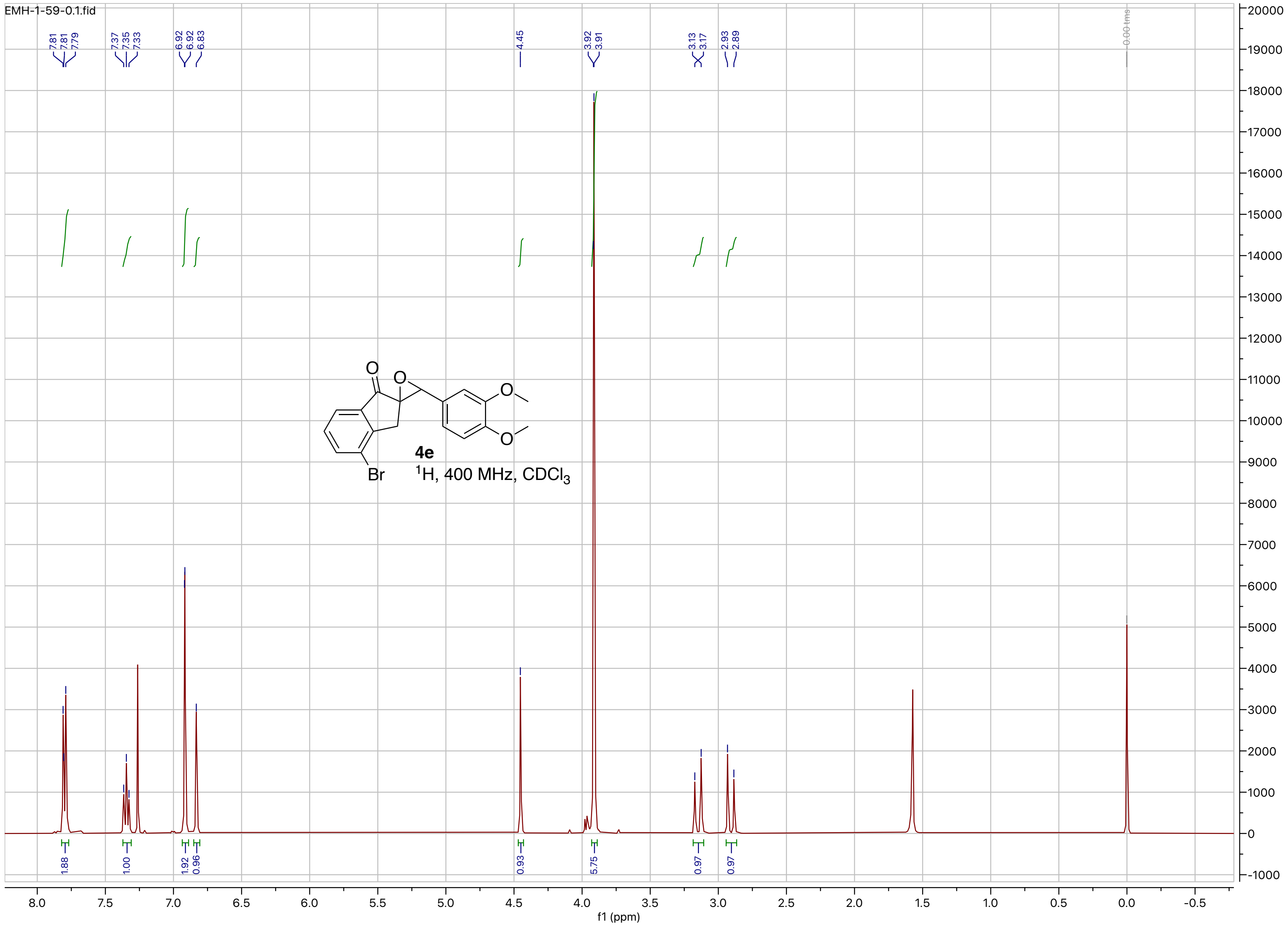

EMH-1-59-0.1.fid
7.81
7.81
7.79
7.37
7.35
7.33
6.92
6.92
6.83
4.45
3.92
3.91
3.13
3.17
2.93
2.89
0.00 tms
4e
^{1}H, 400 MHz, $CDCl_3$
O
O
O
O
Br
1.88
1.00
1.92
0.96
0.93
5.75
0.97
0.97
f1 (ppm)

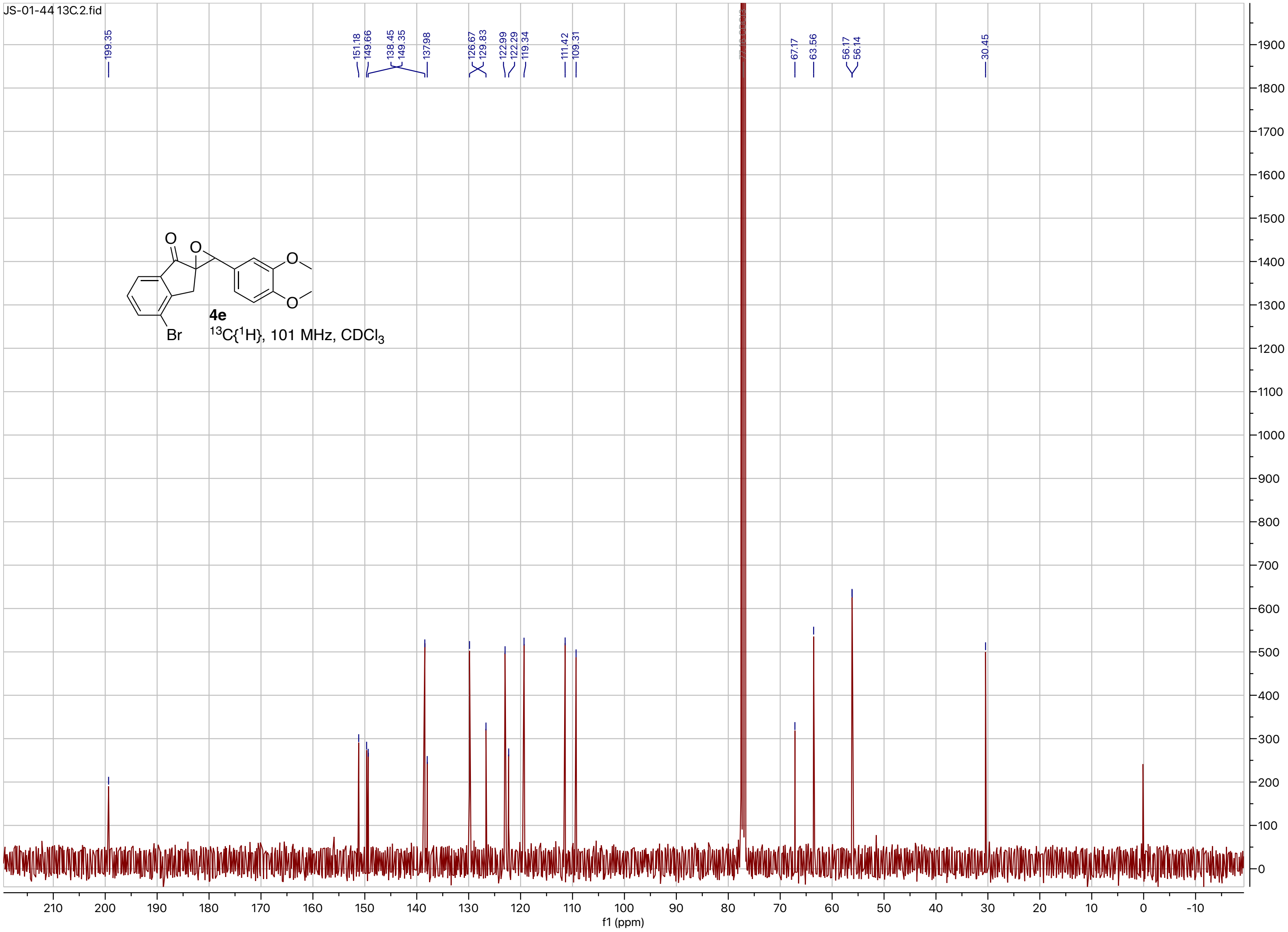
JS-01-44 13C.2.fid
199.35
151.18
149.66
138.45
149.35
137.98
126.67
129.83
122.99
122.29
119.34
111.42
109.31
67.17
63.56
56.17
56.14
30.45
O
O
O
O
Br
4e
13C{1H}, 101 MHz, CDCl3
f1 (ppm)

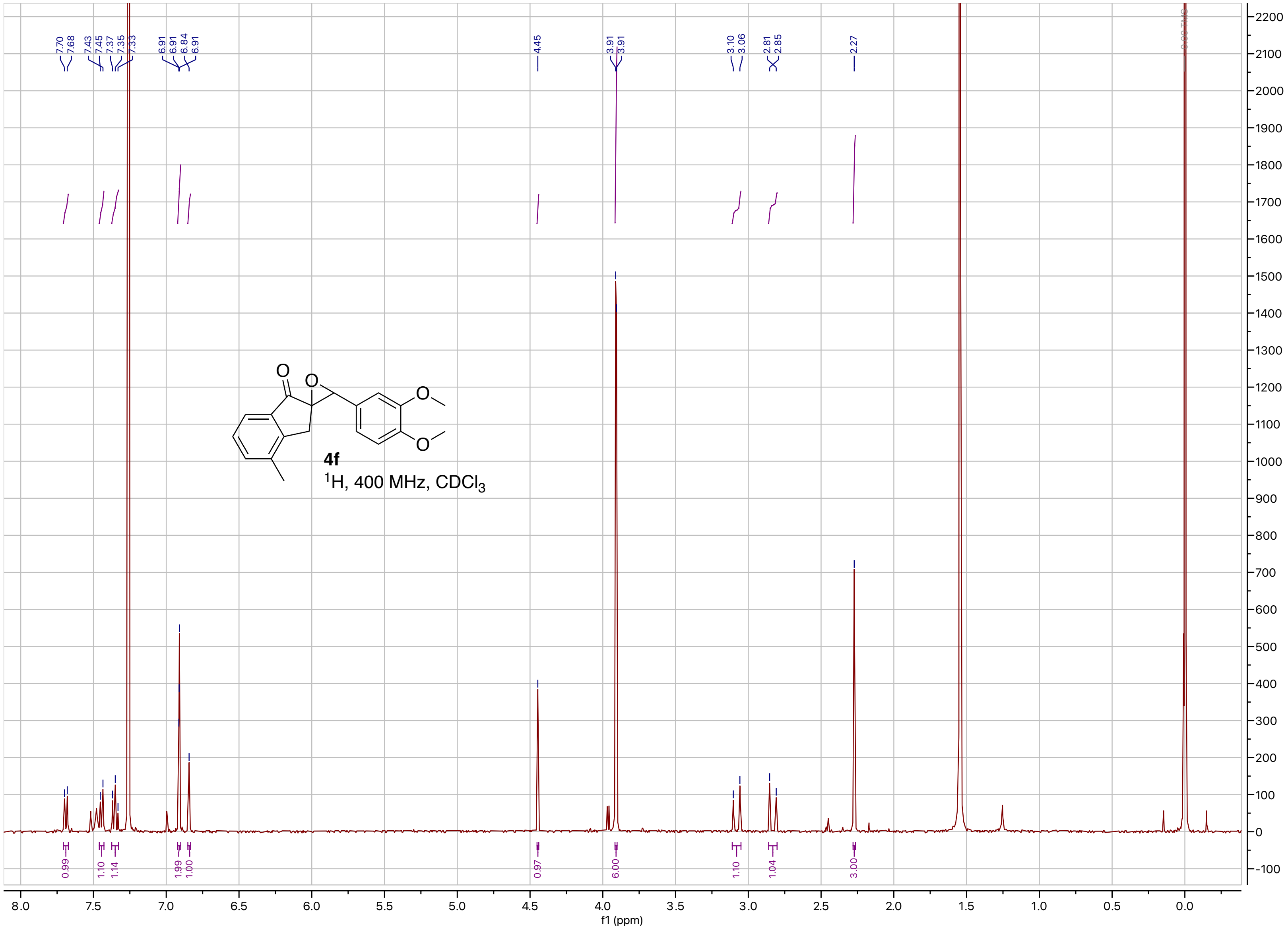
4f
^{1}H, 400 MHz, CDCl$_3$
f1 (ppm)

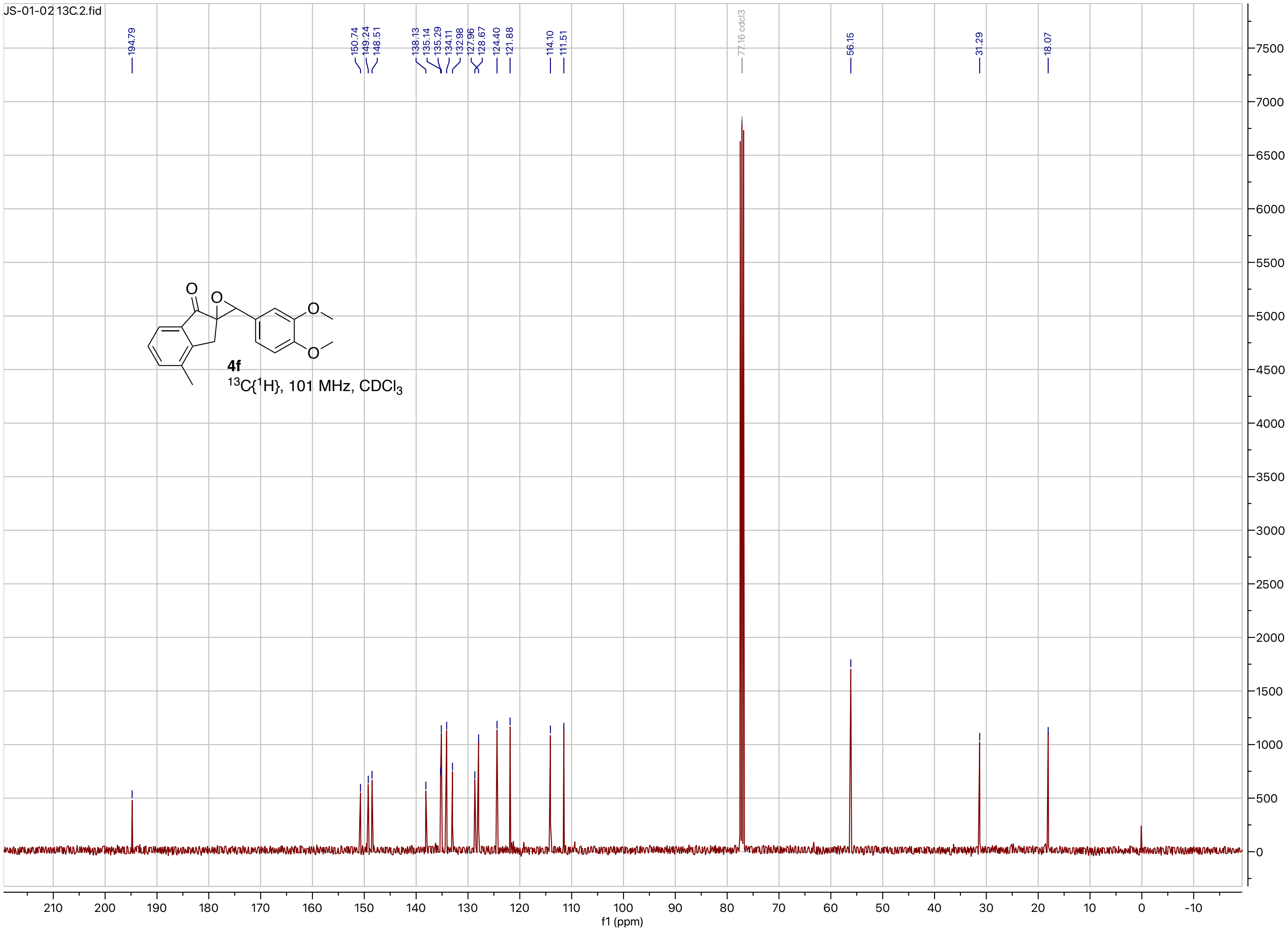

JS-01-02 13C.2.fid
194.79
150.74
149.24
148.51
138.13
135.14
135.29
134.11
132.98
127.96
128.67
124.40
121.88
114.10
111.51
77.16 cdcl3
56.15
31.29
18.07
4f
$^{13}C\{^{1}H\}$, 101 MHz, $CDCl_3$
f1 (ppm)

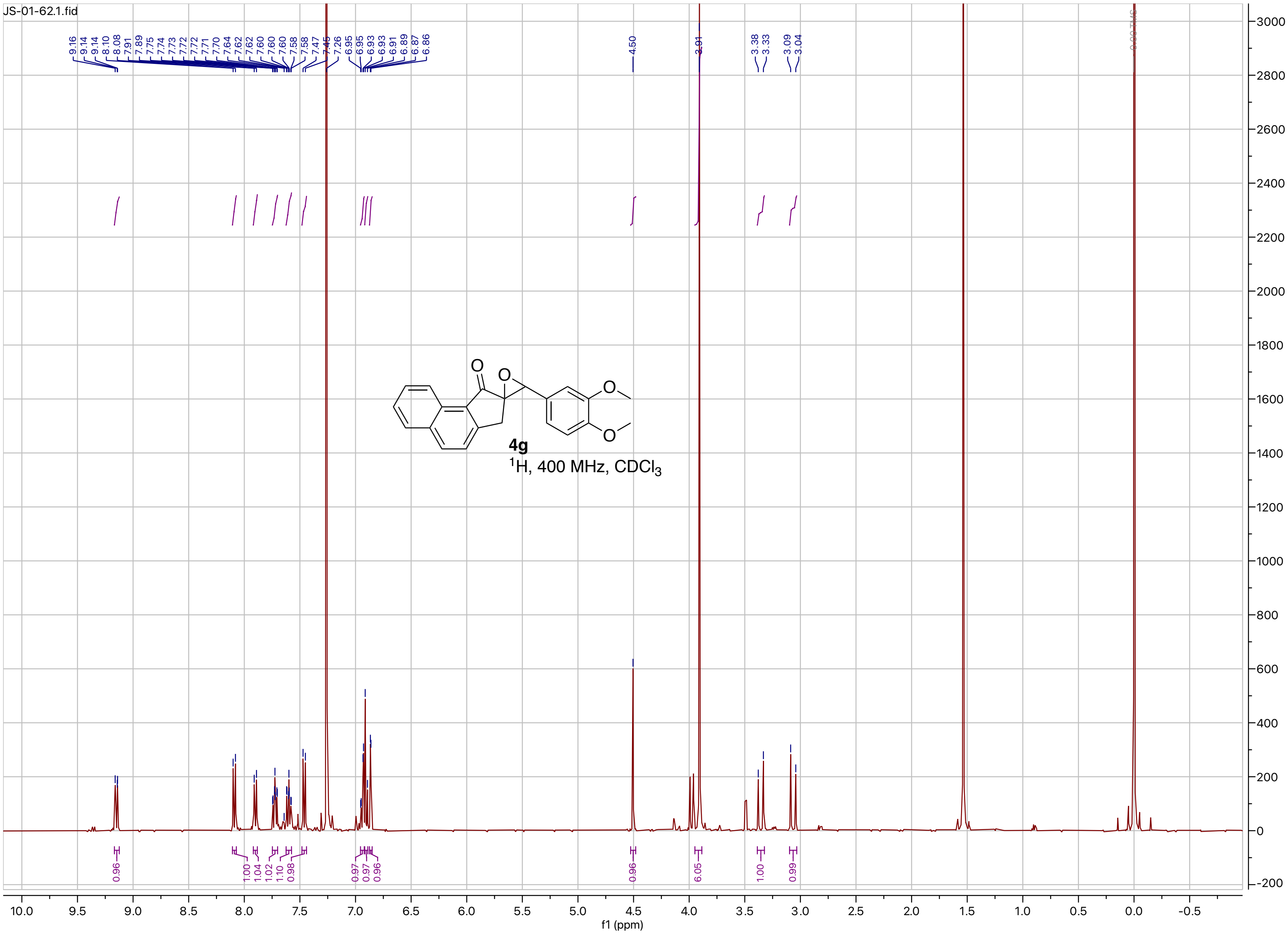

JS-01-62.1.fid
4g
^{1}H, 400 MHz, $CDCl_3$
f1 (ppm)

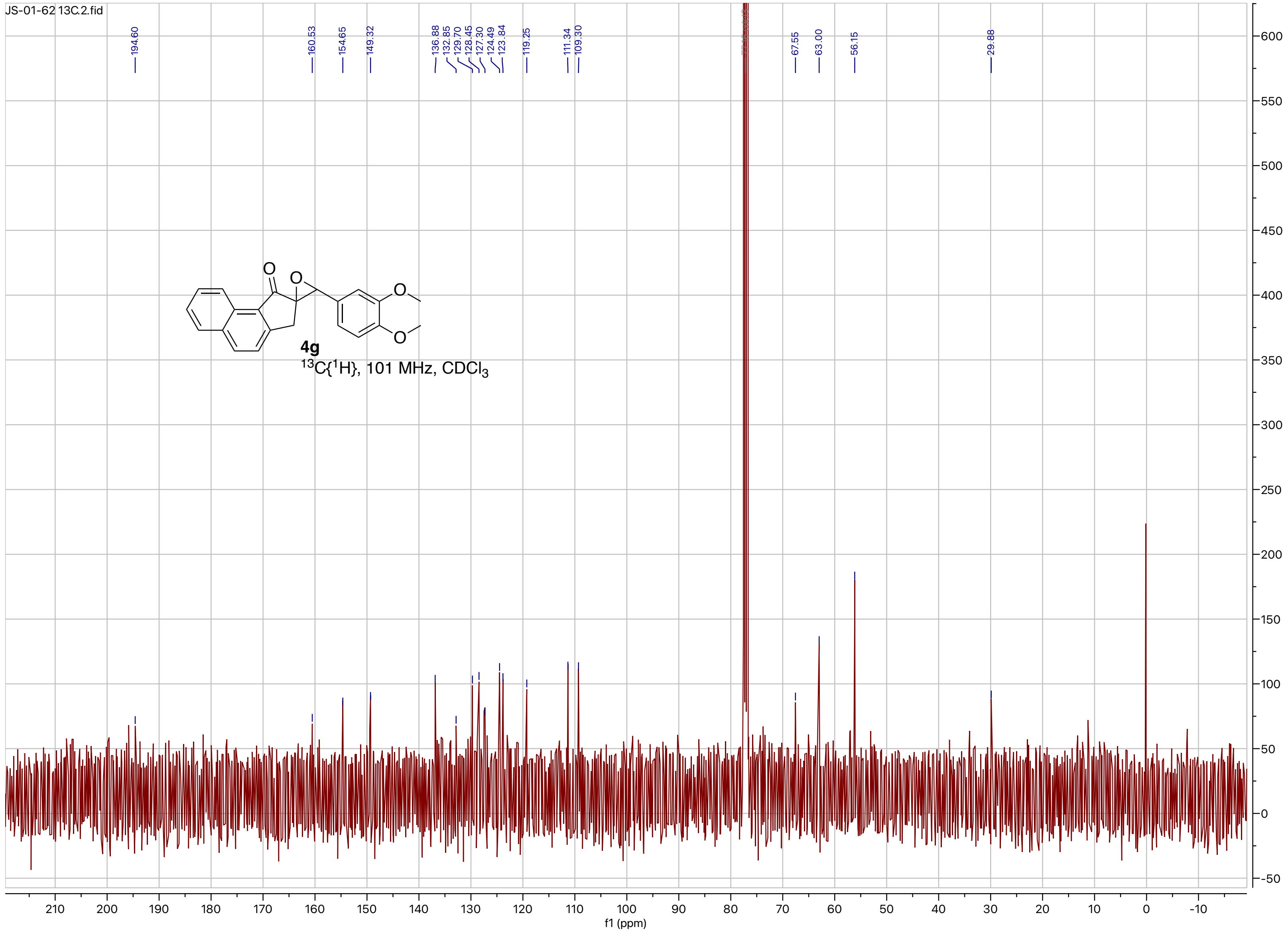
JS-01-62 13C.2.fid
194.60
160.53
154.65
149.32
136.88
132.85
129.70
128.45
127.30
124.49
123.84
119.25
111.34
109.30
67.55
63.00
56.15
29.88
4g
$^{13}C\{^{1}H\}$, 101 MHz, $CDCl_3$
f1 (ppm)

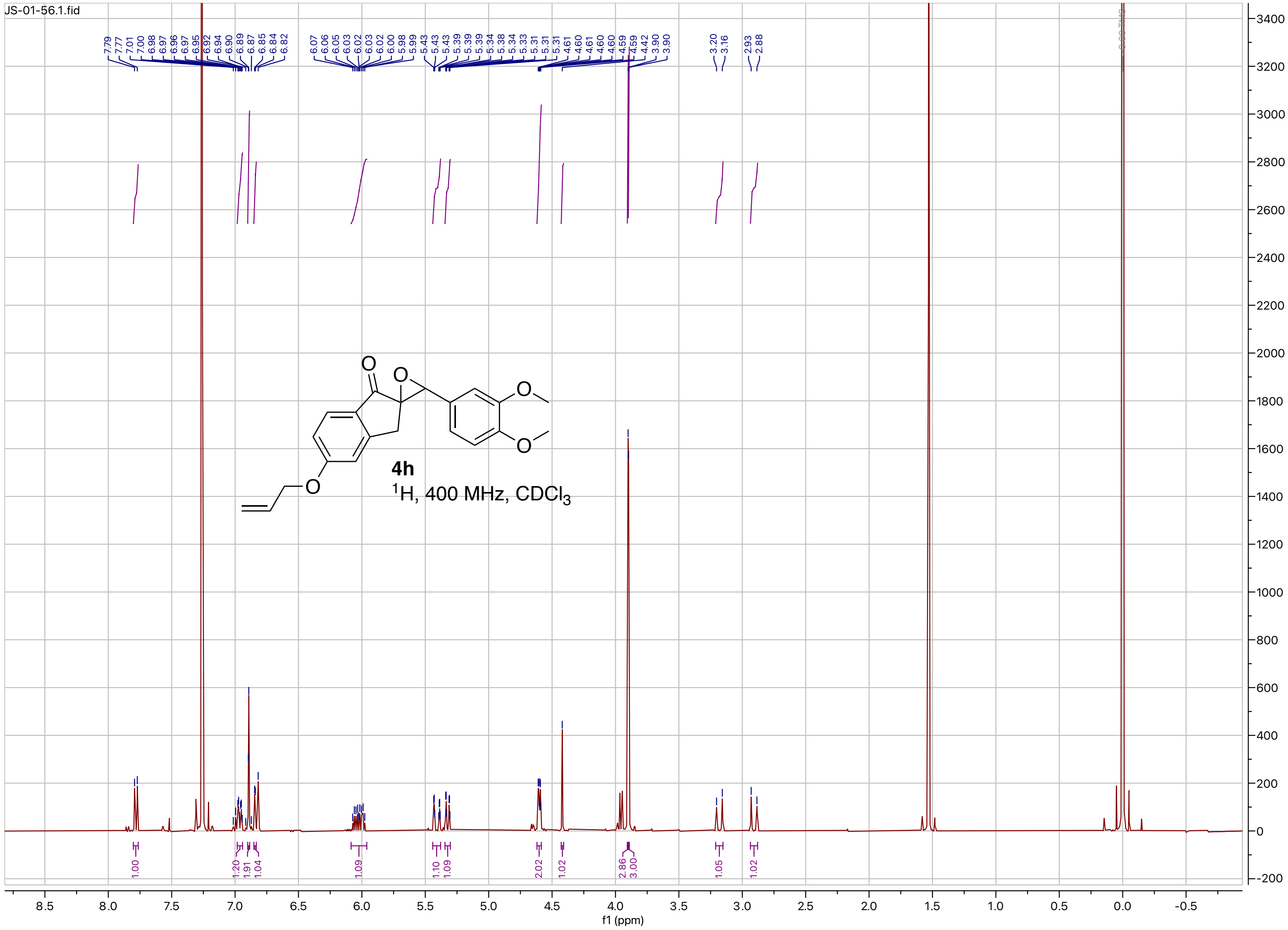
JS-01-56.1.fid
4h
1H, 400 MHz, CDCl3
f1 (ppm)

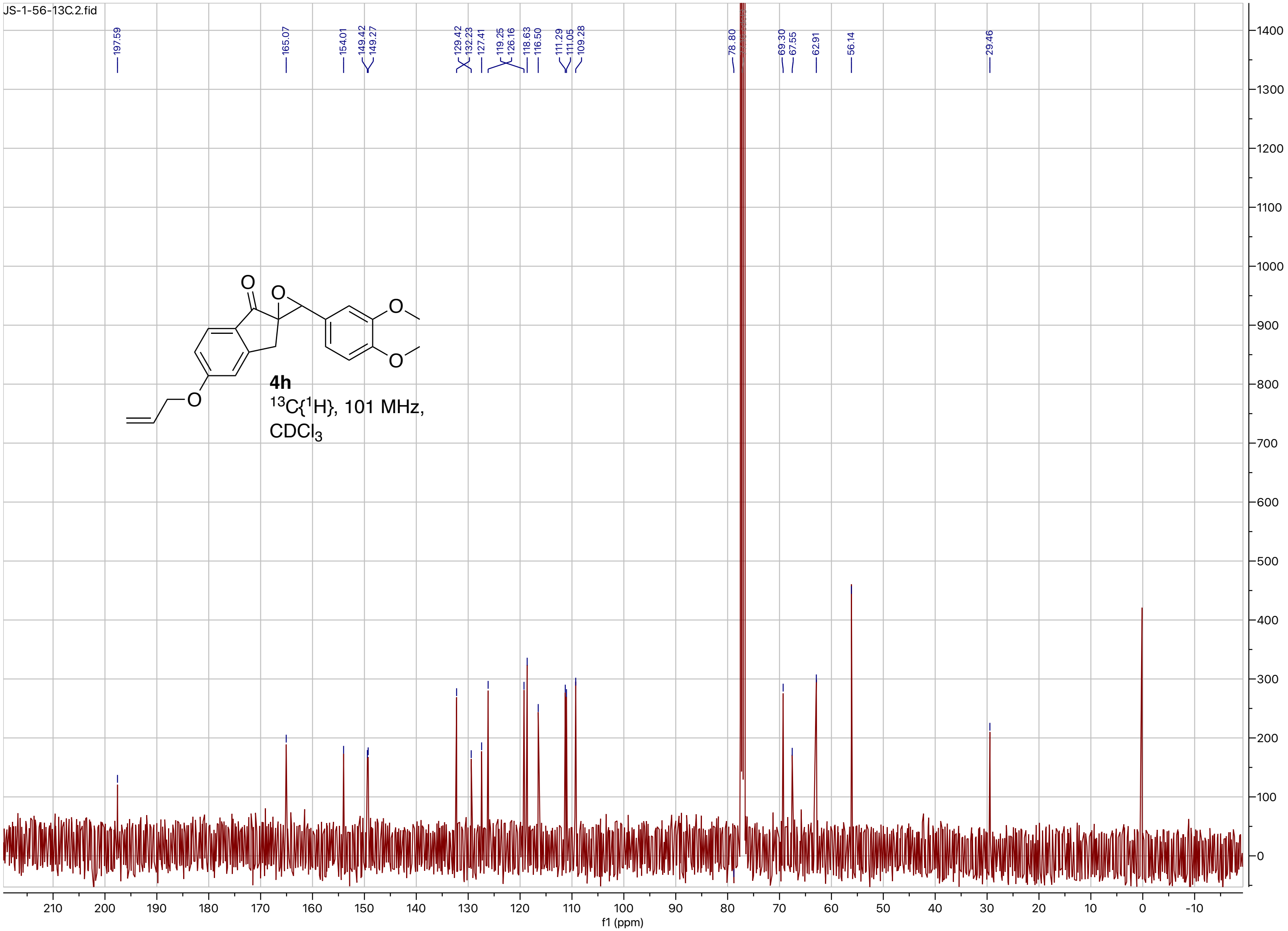
JS-1-56-13C.2.fid
197.59
165.07
154.01
149.42
149.27
129.42
132.23
127.41
119.25
126.16
118.63
116.50
111.29
111.05
109.28
78.80
69.30
67.55
62.91
56.14
29.46
4h
$^{13}C\{^1H\}$, 101 MHz, $CDCl_3$
f1 (ppm)

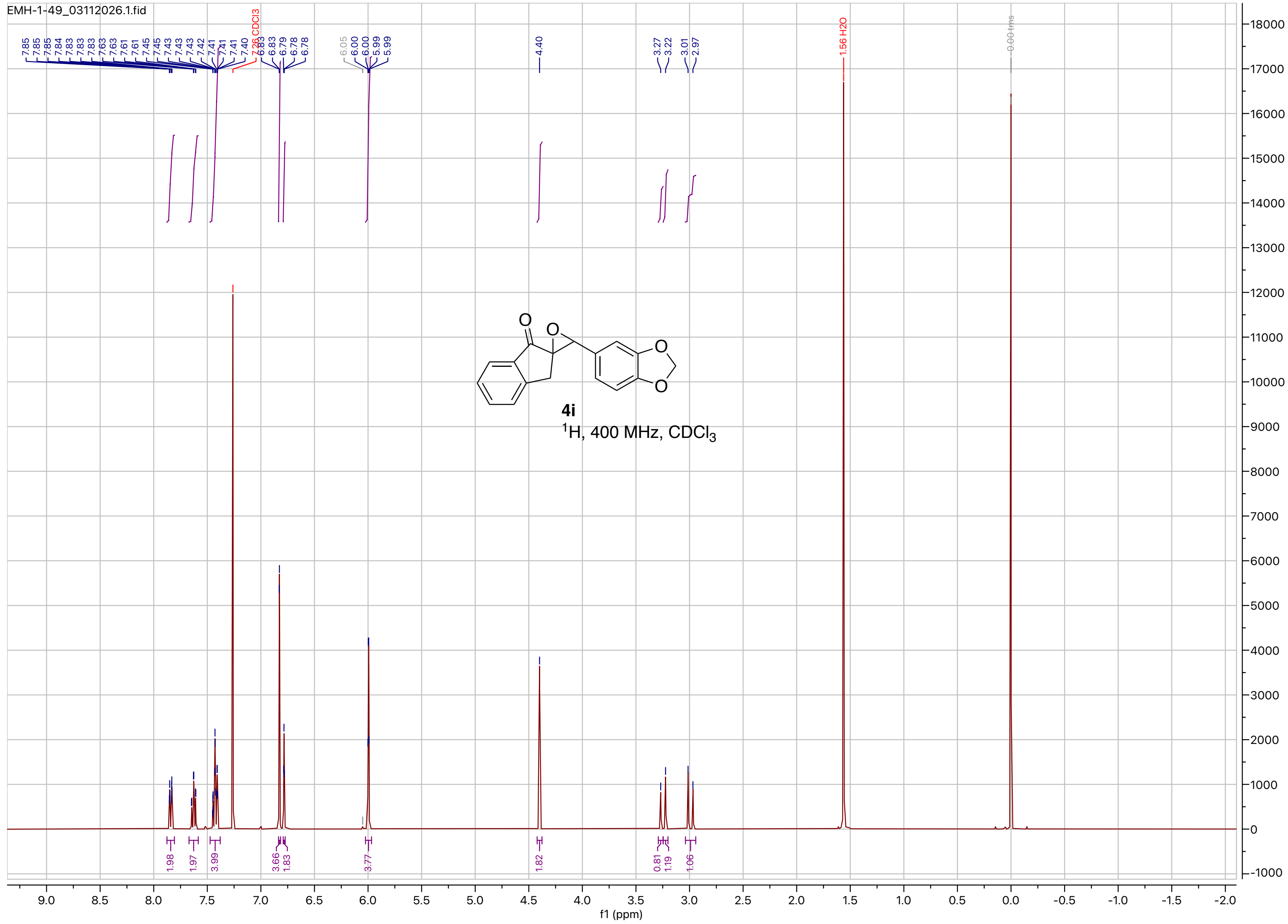
EMH-1-49_03112026.1.fid
7.85 7.85 7.85 7.84 7.83 7.83 7.83 7.63 7.63 7.61 7.61 7.45 7.45 7.43 7.43 7.43 7.42 7.41 7.41 7.41 7.40
7.26 CDCl3
6.83 6.83 6.79 6.78 6.78
6.05 6.00 6.00 5.99 5.99
4.40
3.27 3.22 3.01 2.97
1.56 H2O
0.00 tms
4i
1H, 400 MHz, CDCl3
1.98 1.97 3.99 3.66 1.83 3.77 1.82 0.81 1.19 1.06
f1 (ppm)

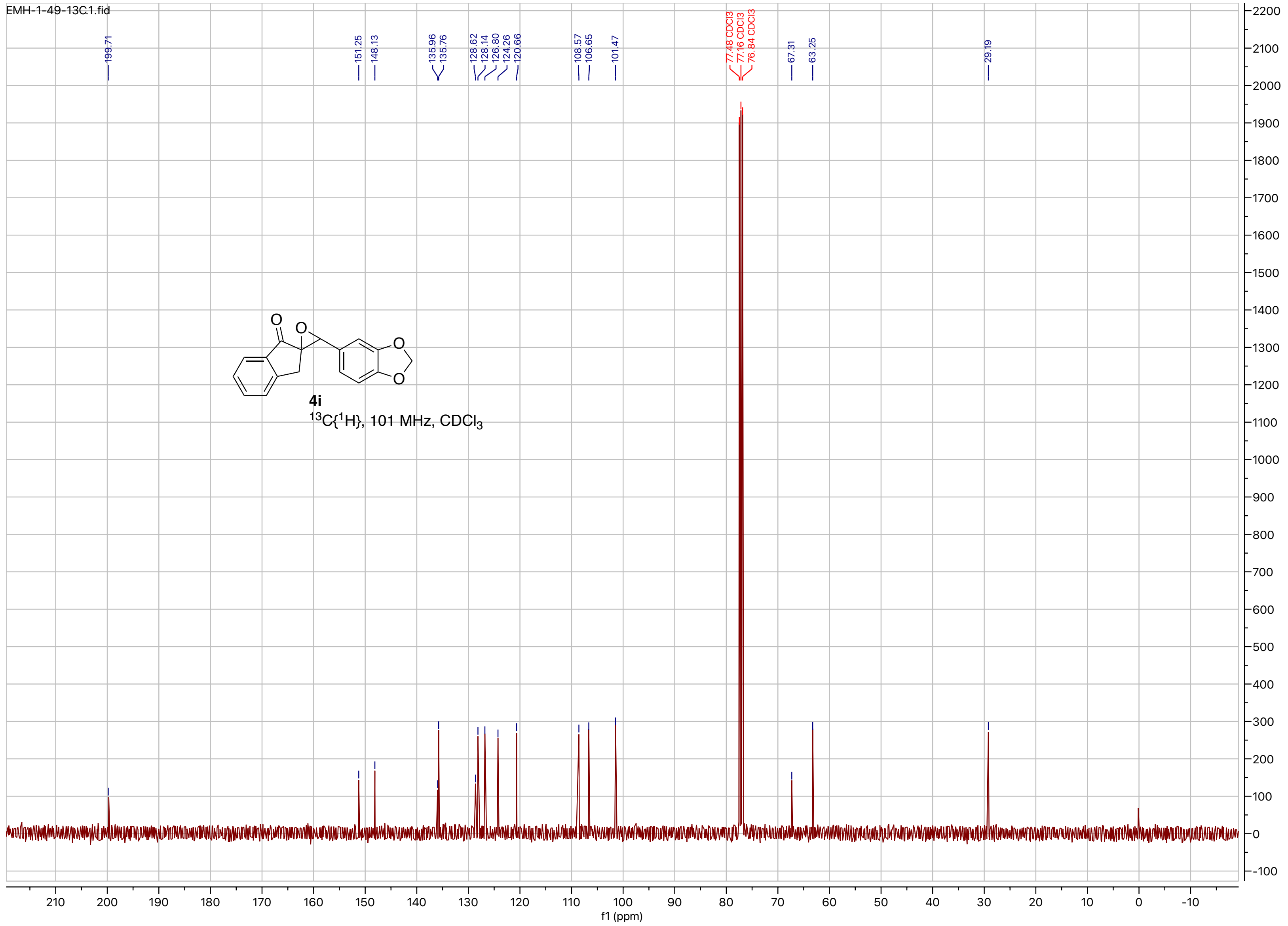
EMH-1-49-13C.1.fid
199.71
151.25
148.13
135.96
135.76
128.62
128.14
126.80
124.26
120.66
108.57
106.65
101.47
77.48 CDCl3
77.16 CDCl3
76.84 CDCl3
67.31
63.25
29.19
4i
13C{1H}, 101 MHz, CDCl3
f1 (ppm)

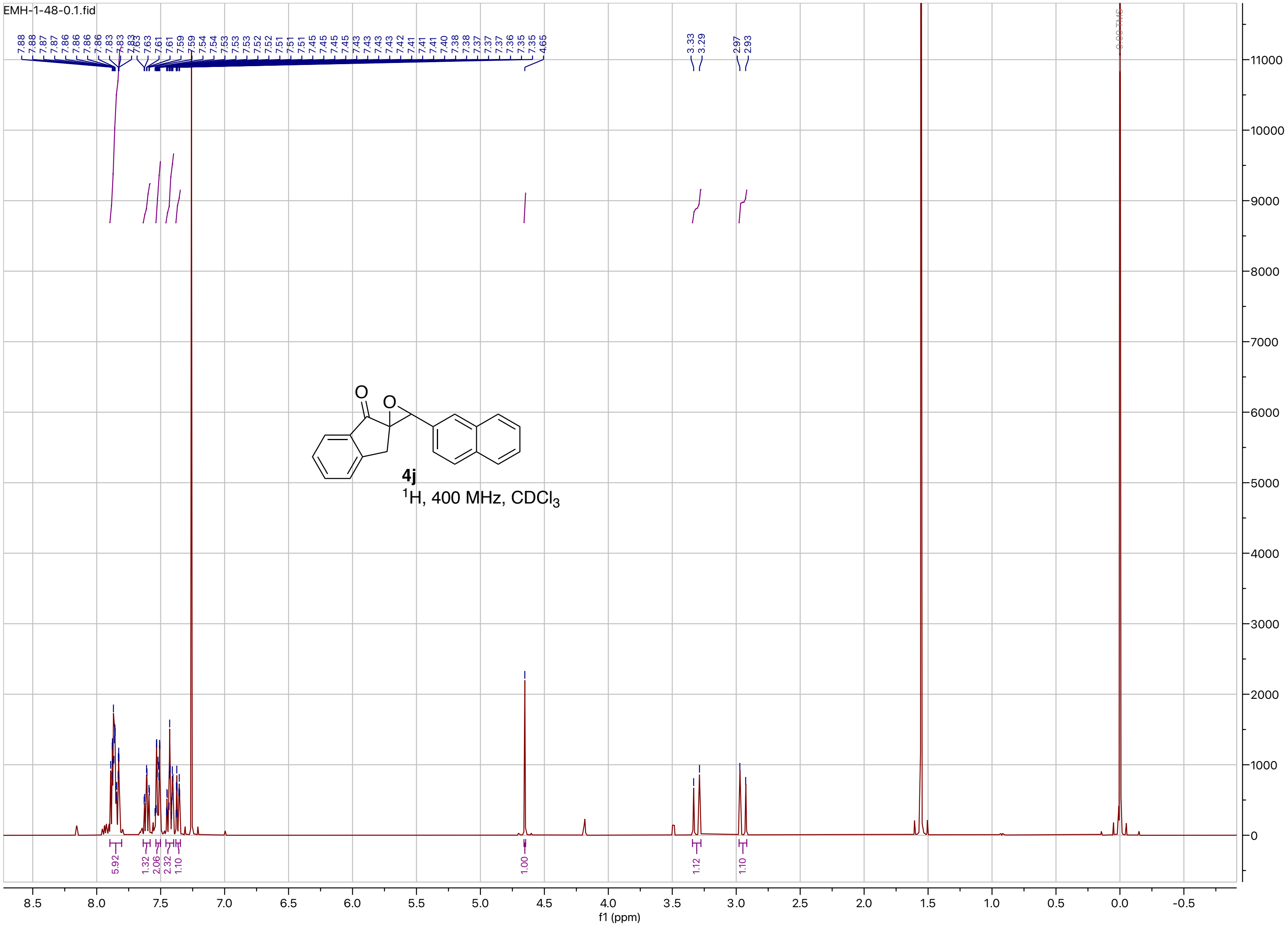
EMH-1-48-0.1.fid
4j
^{1}H, 400 MHz, $CDCl_3$
f1 (ppm)

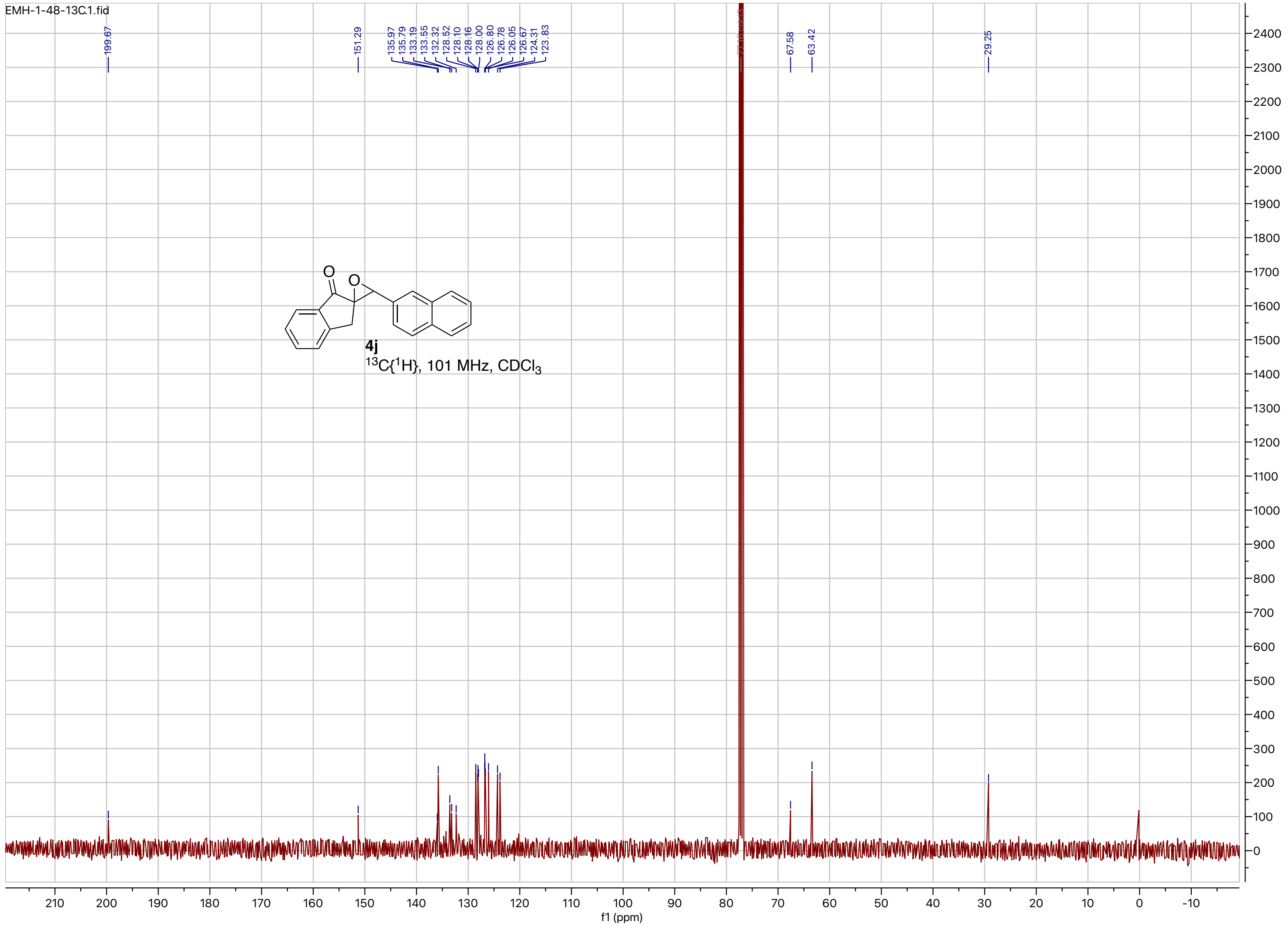
EMH-1-48-13C1.fid
199.67
151.29
135.97
135.79
133.19
133.55
132.32
128.52
128.10
128.16
128.00
126.80
126.78
126.05
126.67
124.31
123.83
67.58
63.42
29.25
4j
$^{13}C\{^{1}H\}$, 101 MHz, $CDCl_3$
f1 (ppm)

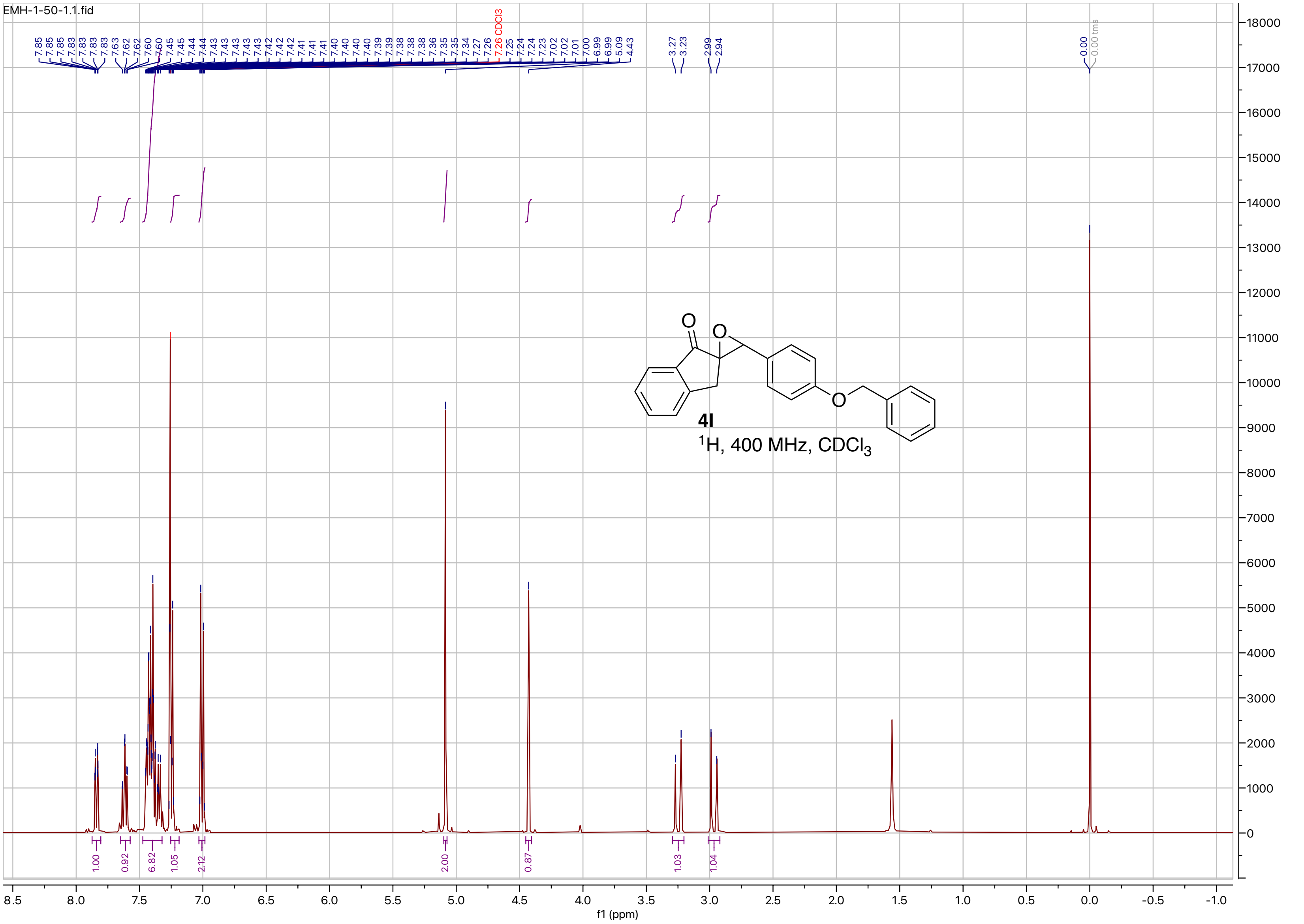
EMH-1-50-1.1.fid
4l
^{1}H, 400 MHz, $CDCl_3$
f1 (ppm)

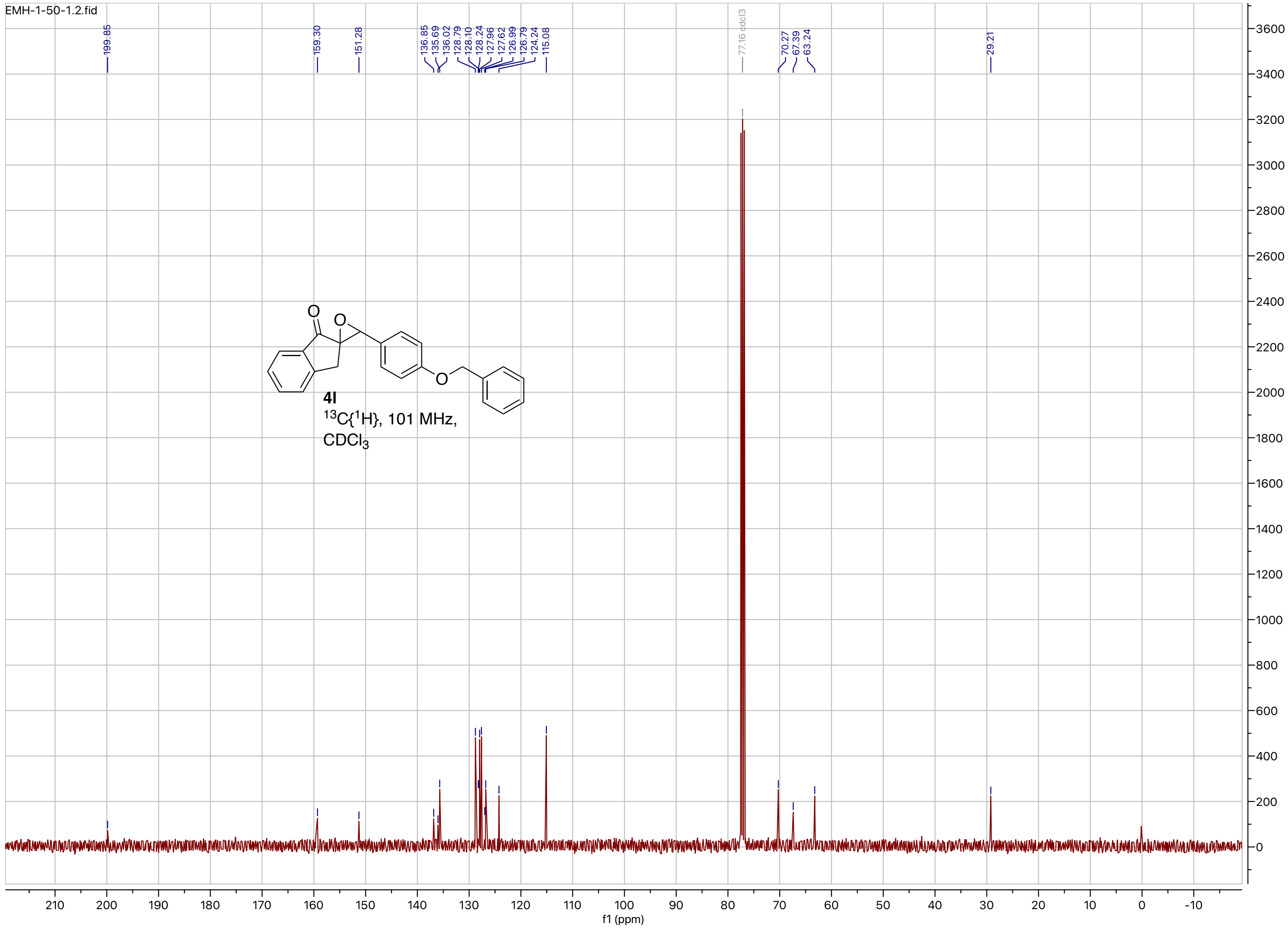
EMH-1-50-1.2.fid
199.85
159.30
151.28
136.85
135.69
136.02
128.79
128.10
128.24
127.96
127.62
126.99
126.79
124.24
115.08
77.16 cdcl3
70.27
67.39
63.24
29.21
4l
13C{1H}, 101 MHz,
CDCl3
f1 (ppm)

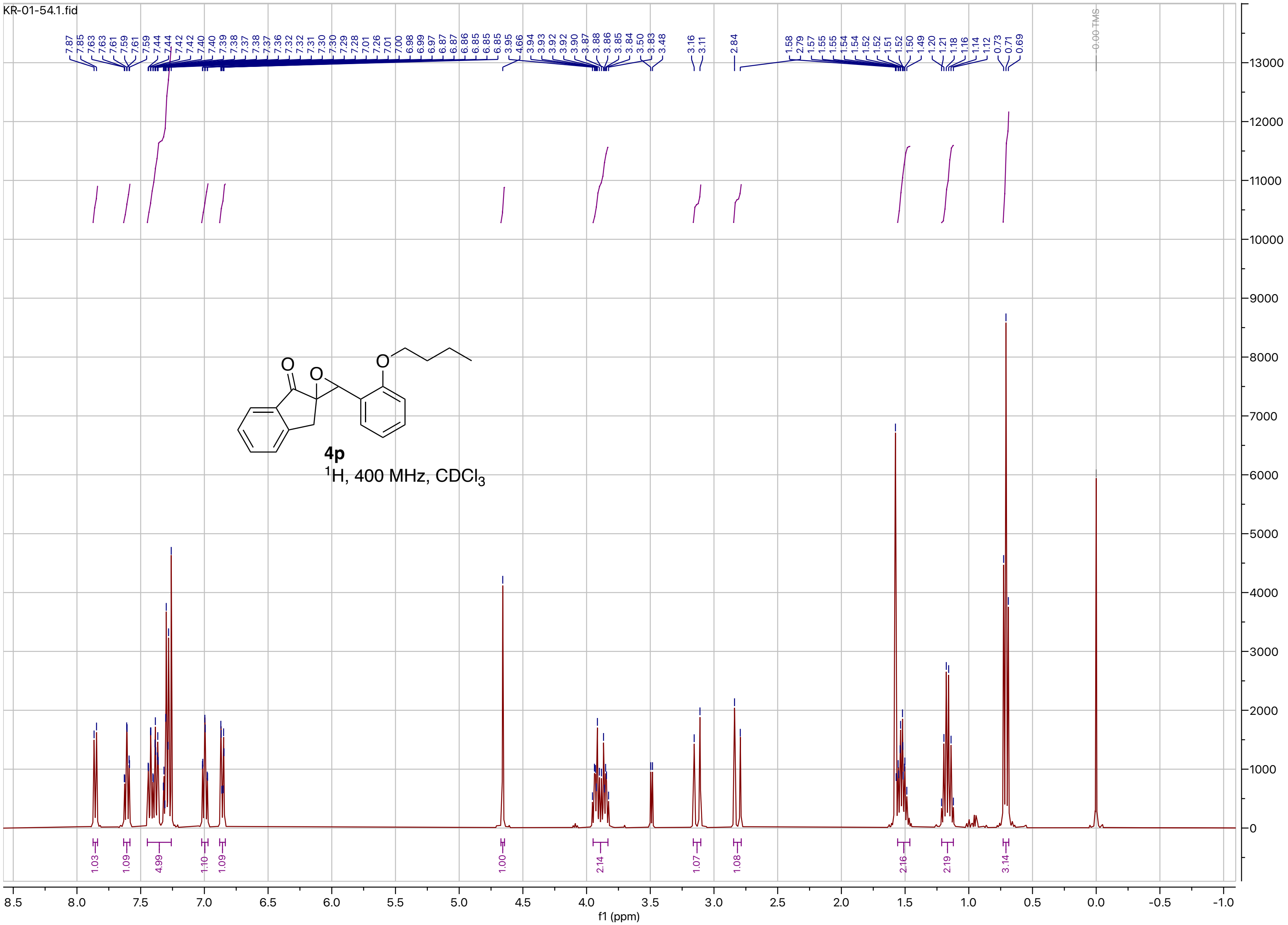

KR-01-54.1.fid
4p
¹H, 400 MHz, CDCl₃
f1 (ppm)

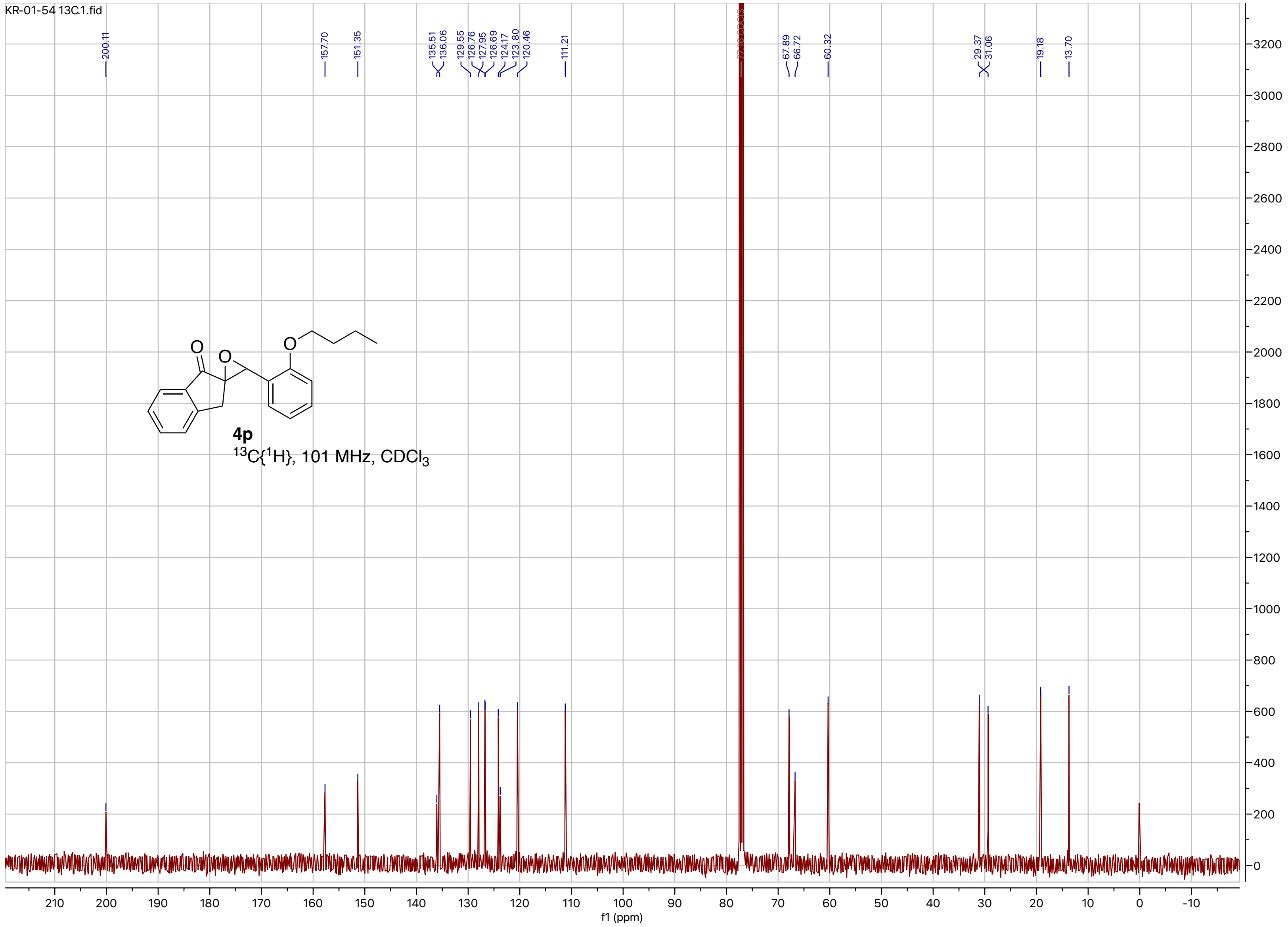
KR-01-54 13C1.fid
200.11
157.70
151.35
135.51
136.06
129.55
128.76
127.95
126.69
124.17
123.80
120.46
111.21
67.89
66.72
60.32
29.37
31.06
19.18
13.70
4p
¹³C{¹H}, 101 MHz, CDCl₃
f1 (ppm)

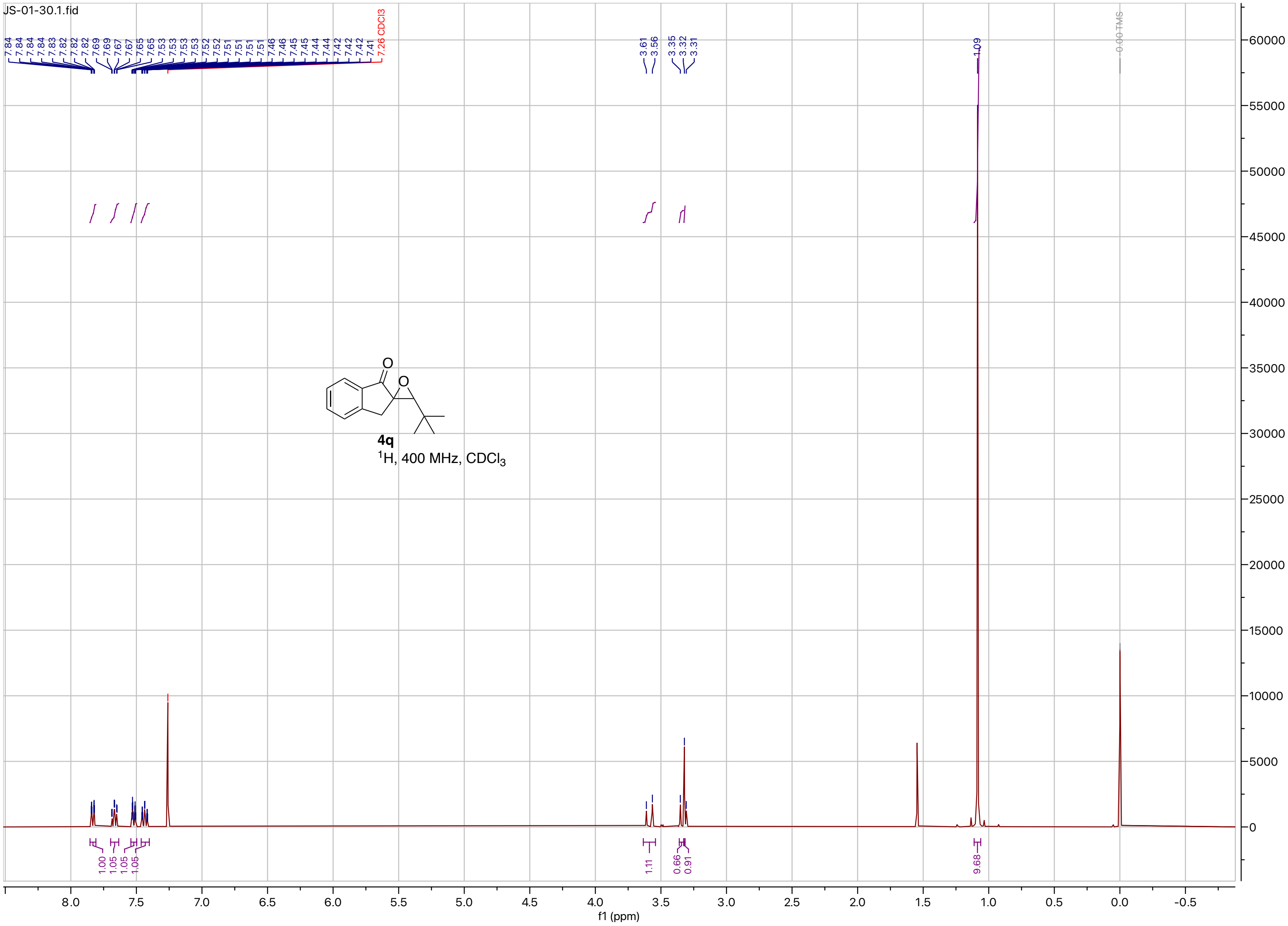
JS-01-30.1.fid
7.84
7.84
7.84
7.84
7.83
7.82
7.82
7.82
7.69
7.69
7.67
7.67
7.65
7.65
7.53
7.53
7.53
7.53
7.52
7.52
7.51
7.51
7.51
7.51
7.46
7.46
7.45
7.45
7.44
7.44
7.42
7.42
7.42
7.41
7.26 CDCl3
3.61
3.56
3.35
3.32
3.31
1.09
0.00 TMS
4q
¹H, 400 MHz, CDCl₃
1.00
1.05
1.05
1.05
1.11
0.66
0.91
9.68
f1 (ppm)

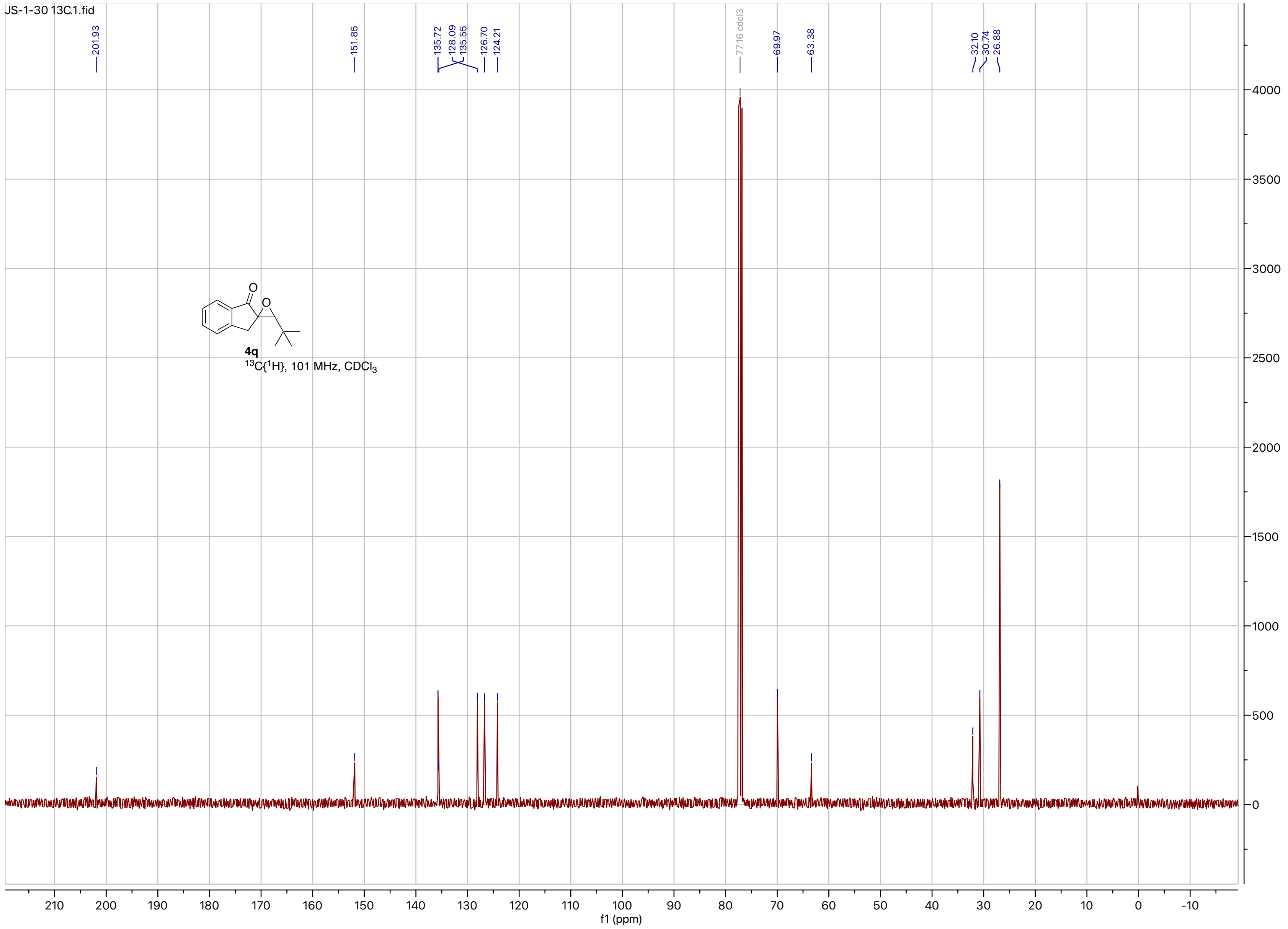
JS-1-30 13C.1.fid
201.93
151.85
135.72
128.09
135.55
126.70
124.21
77.16 cdcl3
69.97
63.38
32.10
30.74
26.88
O
O
4q
$^{13}C\{^1H\}$, 101 MHz, $CDCl_3$
f1 (ppm)

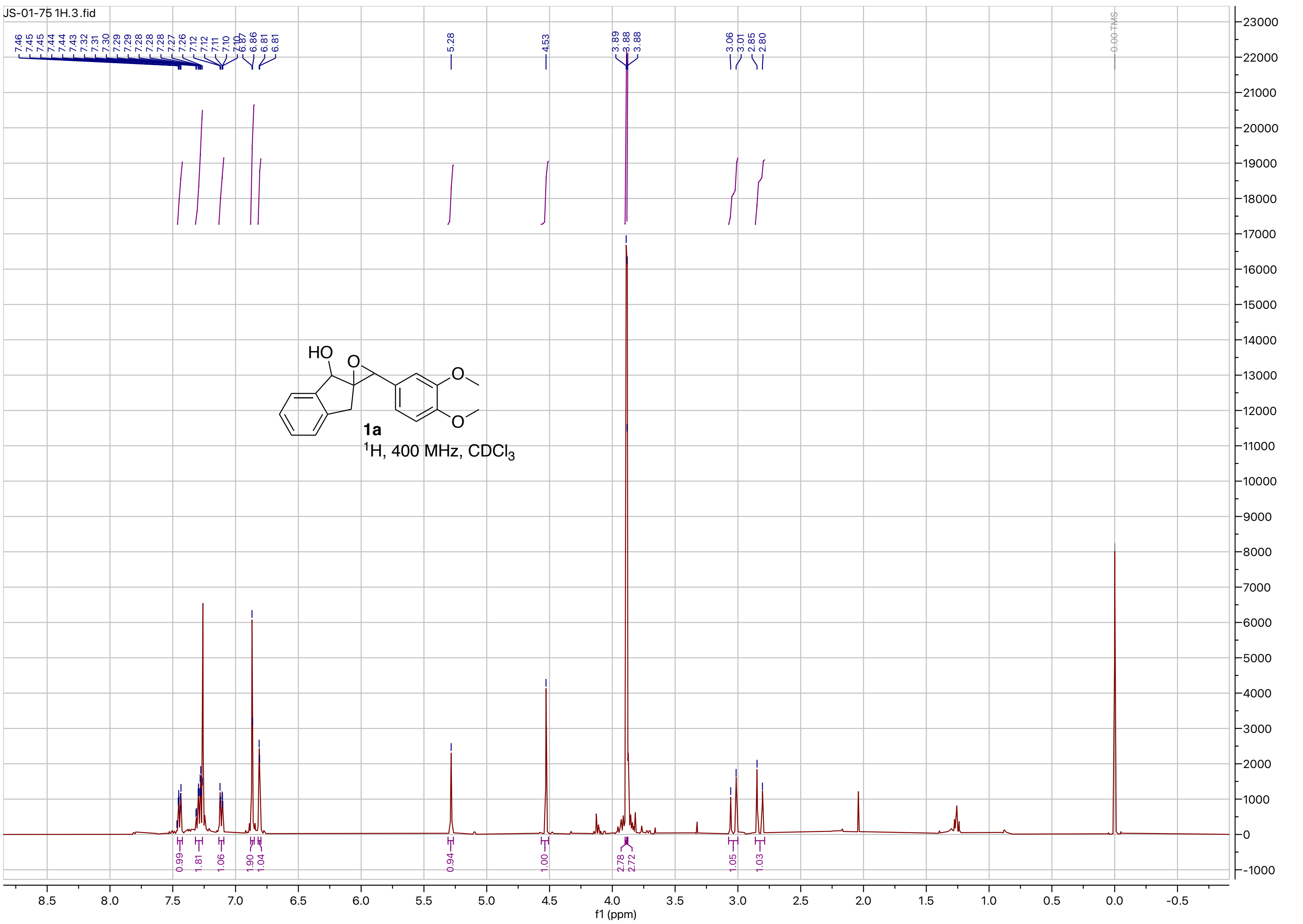
JS-01-75 1H.3.fid
7.46
7.45
7.45
7.44
7.44
7.43
7.32
7.31
7.30
7.29
7.29
7.28
7.28
7.28
7.27
7.26
7.12
7.12
7.11
7.10
7.10
6.87
6.86
6.81
6.81
5.28
4.53
3.89
3.88
3.88
3.06
3.01
2.85
2.80
0.00 TMS
HO
O
O
O
1a
1H, 400 MHz, CDCl3
0.99
1.81
1.06
1.90
1.04
0.94
1.00
2.78
2.72
1.05
1.03
f1 (ppm)

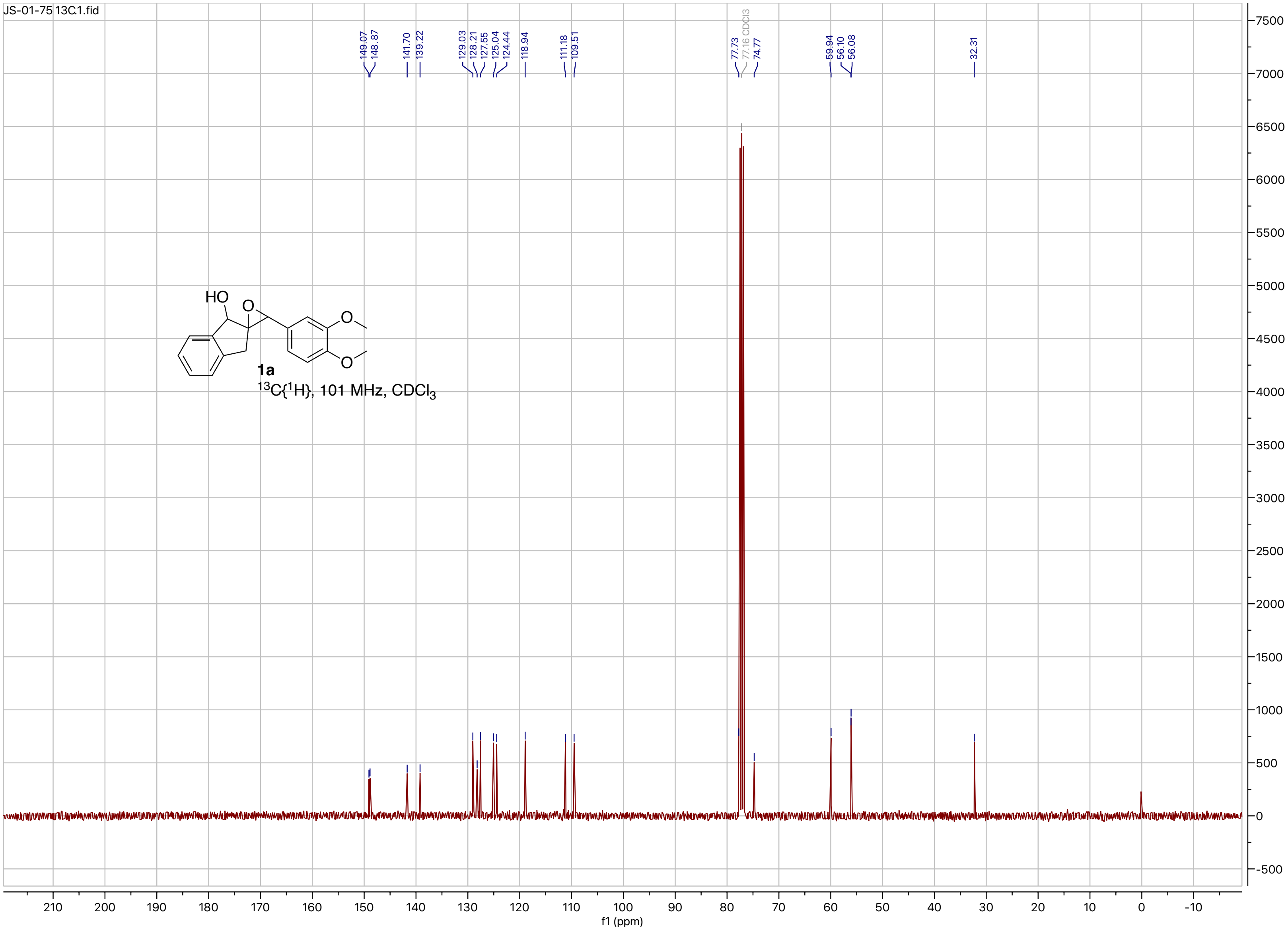
JS-01-75 13C.1.fid
149.07
148.87
141.70
139.22
129.03
128.21
127.55
125.04
124.44
118.94
111.18
109.51
77.73
77.16 CDCl3
74.77
59.94
56.10
56.08
32.31
HO
O
O
O
1a
$^{13}C\{^{1}H\}$, 101 MHz, $CDCl_3$
f1 (ppm)

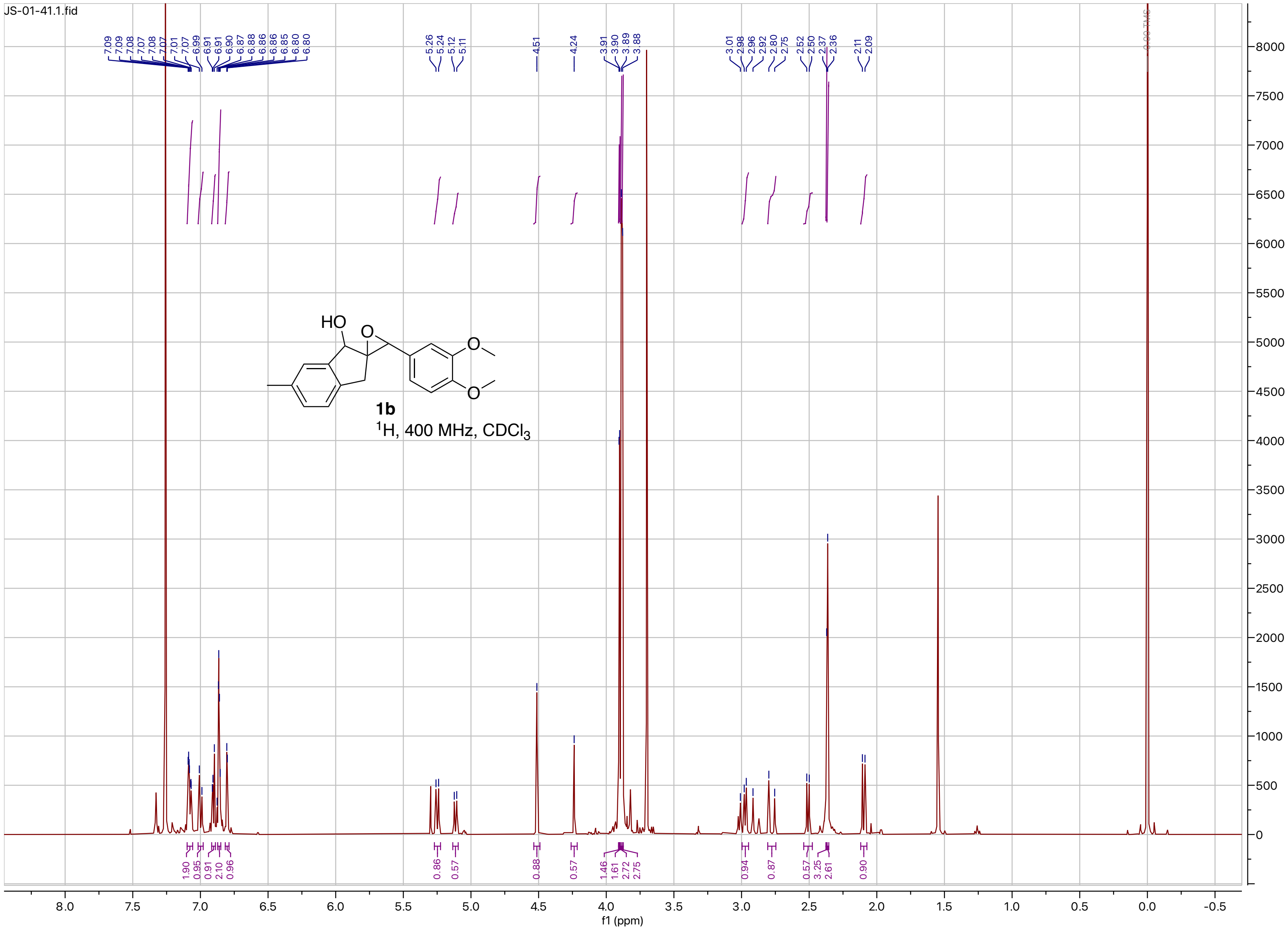

JS-01-41.1.fid
HO
O
1b
^{1}H, 400 MHz, $CDCl_3$
f1 (ppm)

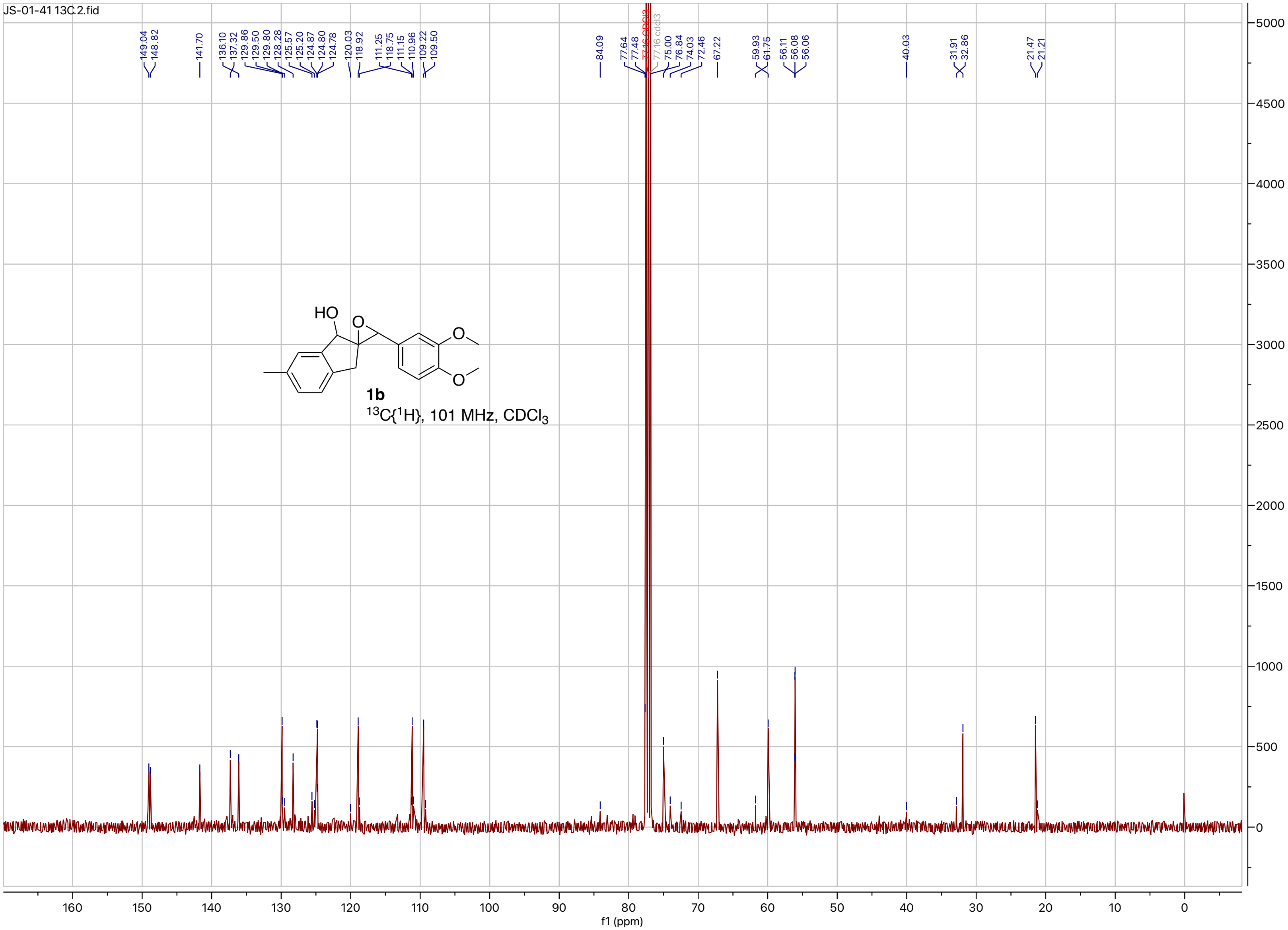
JS-01-41 13C.2.fid
149.04
148.82
141.70
136.10
137.32
129.86
129.50
129.80
128.28
125.57
125.20
124.87
124.80
124.78
120.03
118.92
111.25
118.75
111.15
110.96
109.22
109.50
84.09
77.64
77.48
77.16 cdcl3
75.00
76.84
74.03
72.46
67.22
59.93
61.75
56.11
56.08
56.06
40.03
31.91
32.86
21.47
21.21
HO
O
1b
$^{13}C\{^1H\}$, 101 MHz, $CDCl_3$
f1 (ppm)

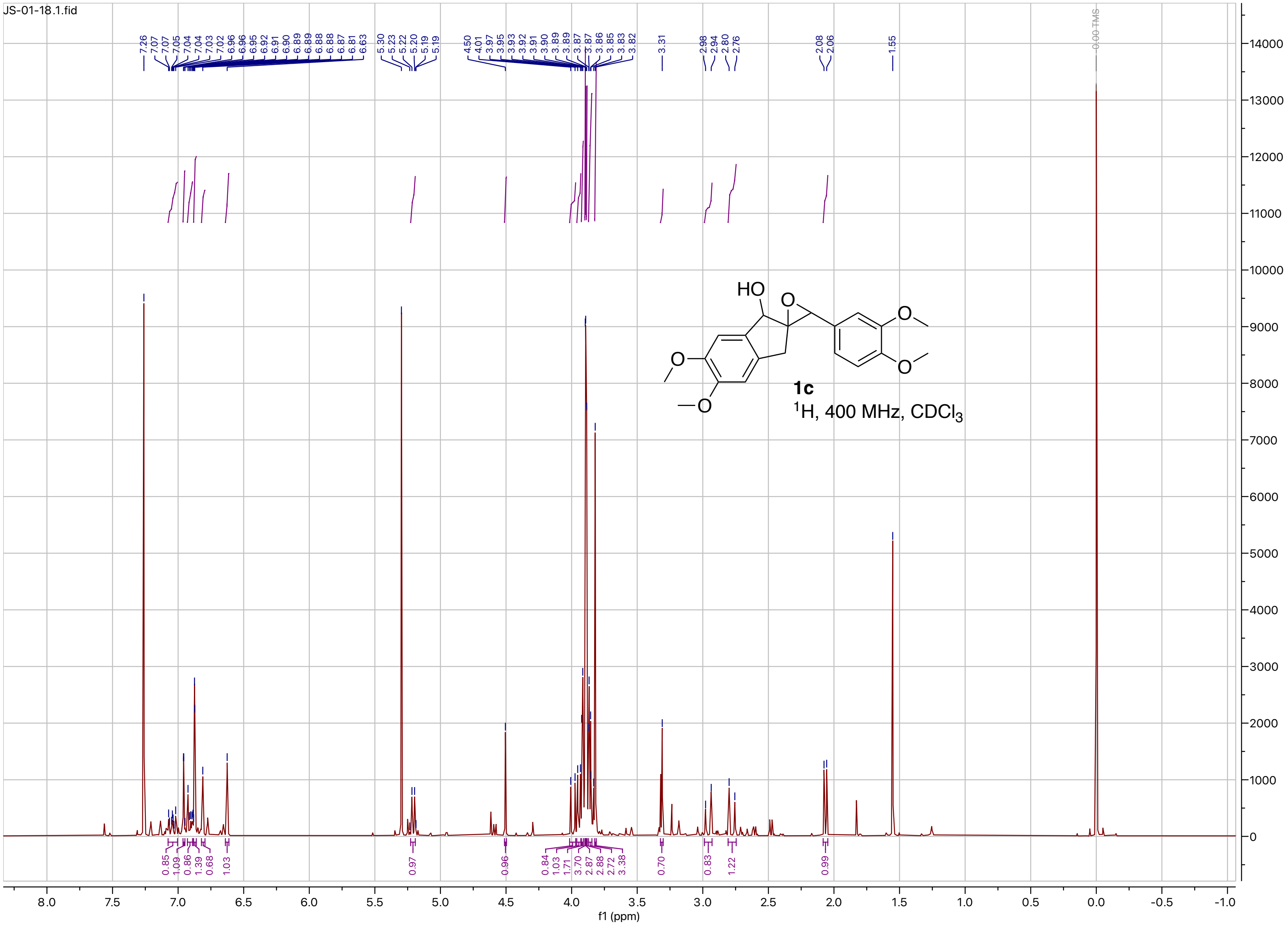
JS-01-18.1.fid
1c
¹H, 400 MHz, CDCl3
f1 (ppm)

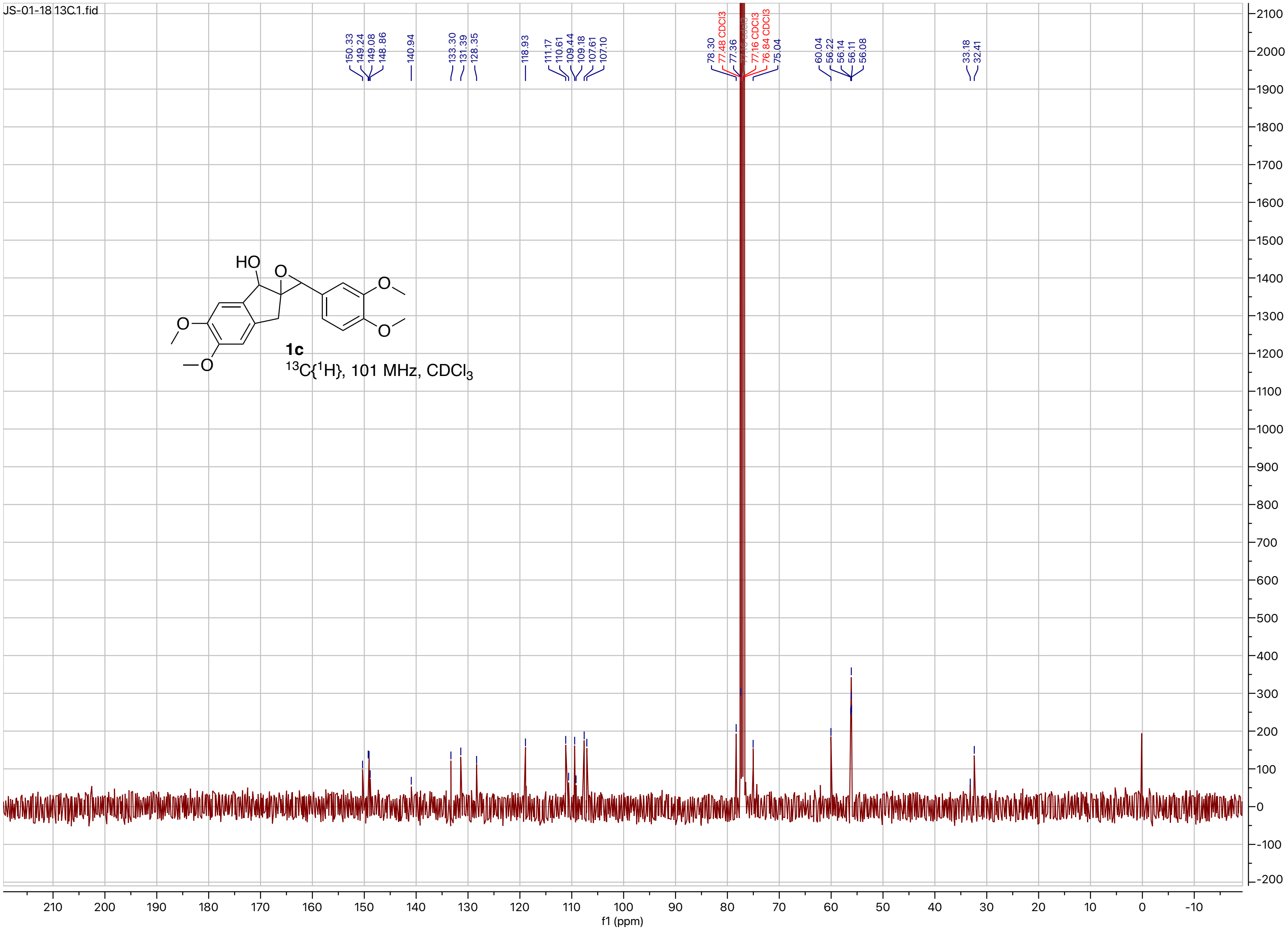

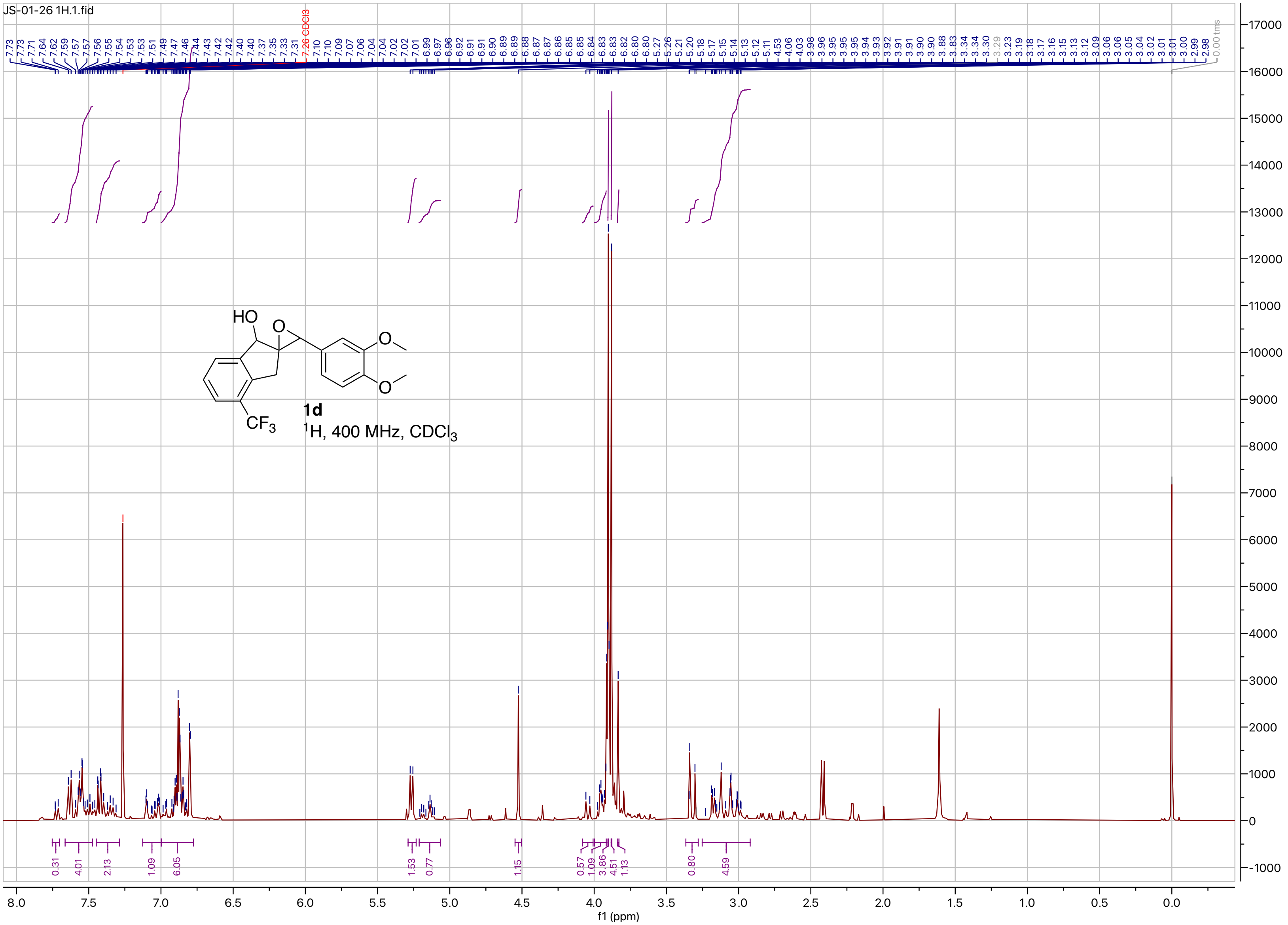
JS-01-26 1H.1.fid
1d
¹H, 400 MHz, CDCl3
HO
O
CF3
f1 (ppm)

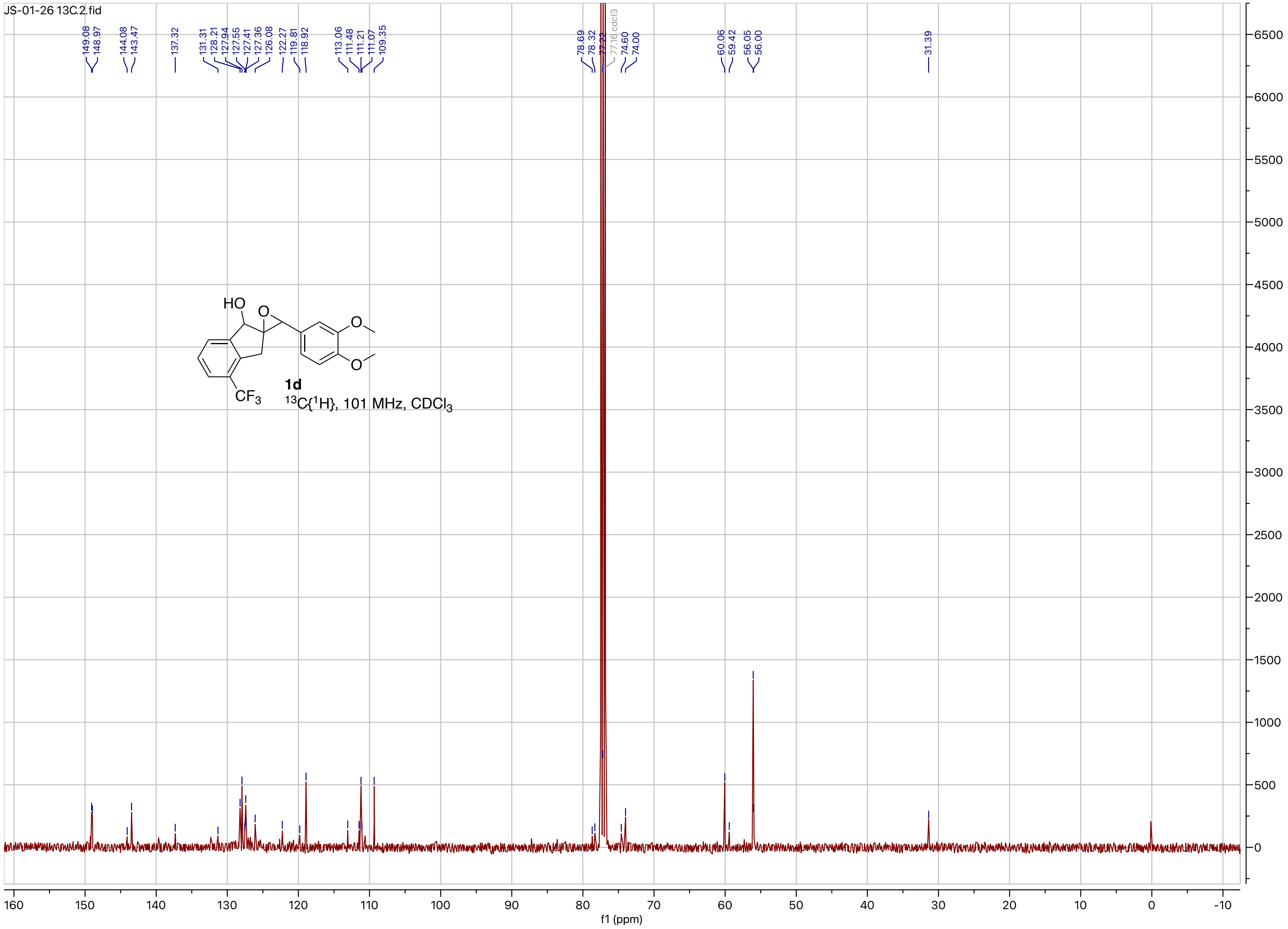
JS-01-26 13C.2.fid
149.08
148.97
144.08
143.47
137.32
131.31
128.21
127.94
127.55
127.41
127.36
126.08
122.27
119.81
118.92
113.06
111.48
111.21
111.07
109.35
78.69
78.32
77.16 cdcl3
74.60
74.00
60.06
59.42
56.05
56.00
31.39
HO
O
O
O
CF3
1d
13C{1H}, 101 MHz, CDCl3
f1 (ppm)

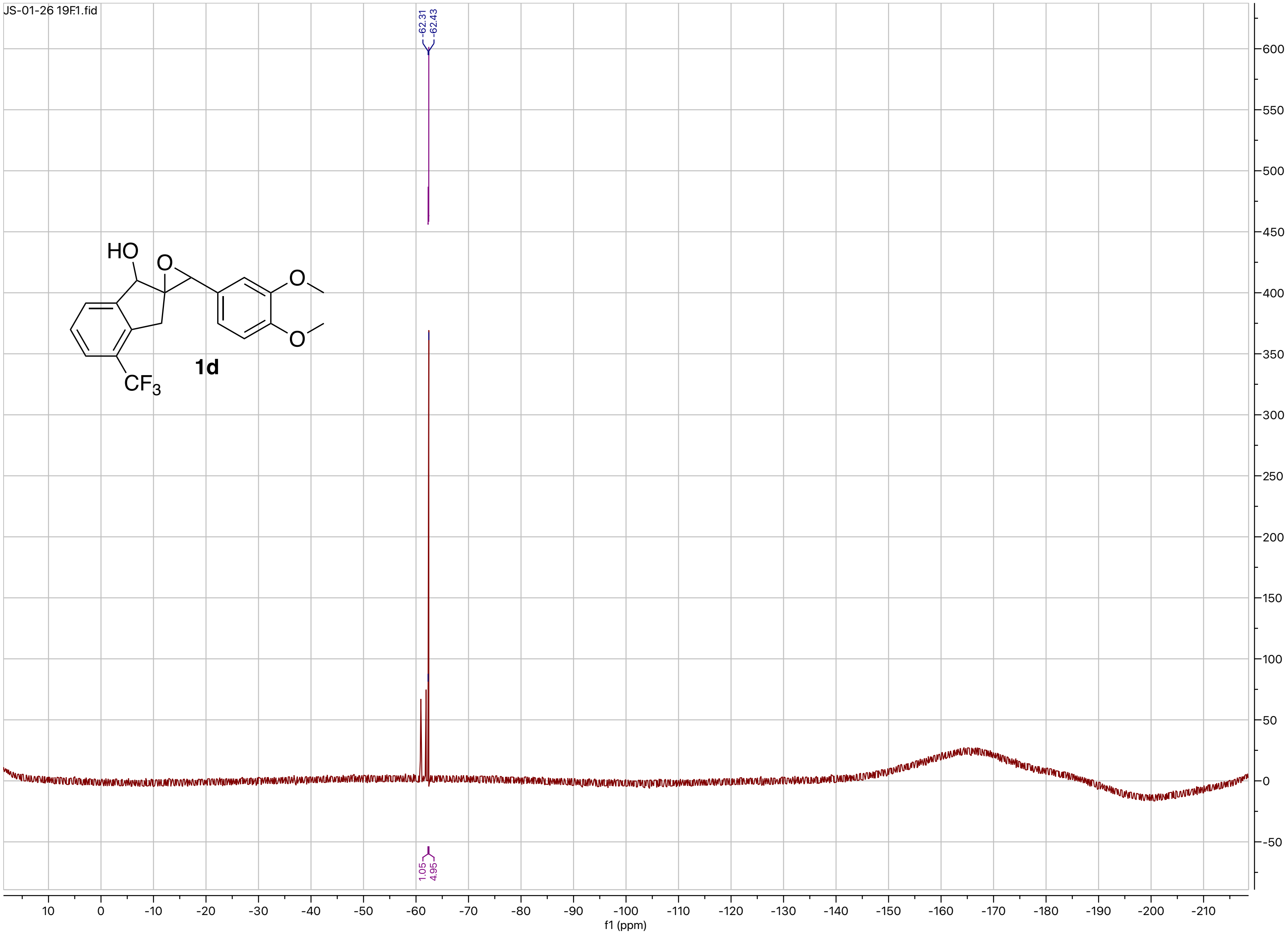

JS-01-26 19F1.fid
-62.31
-62.43
HO
O
O
O
CF3
1d
1.05
4.95
f1 (ppm)

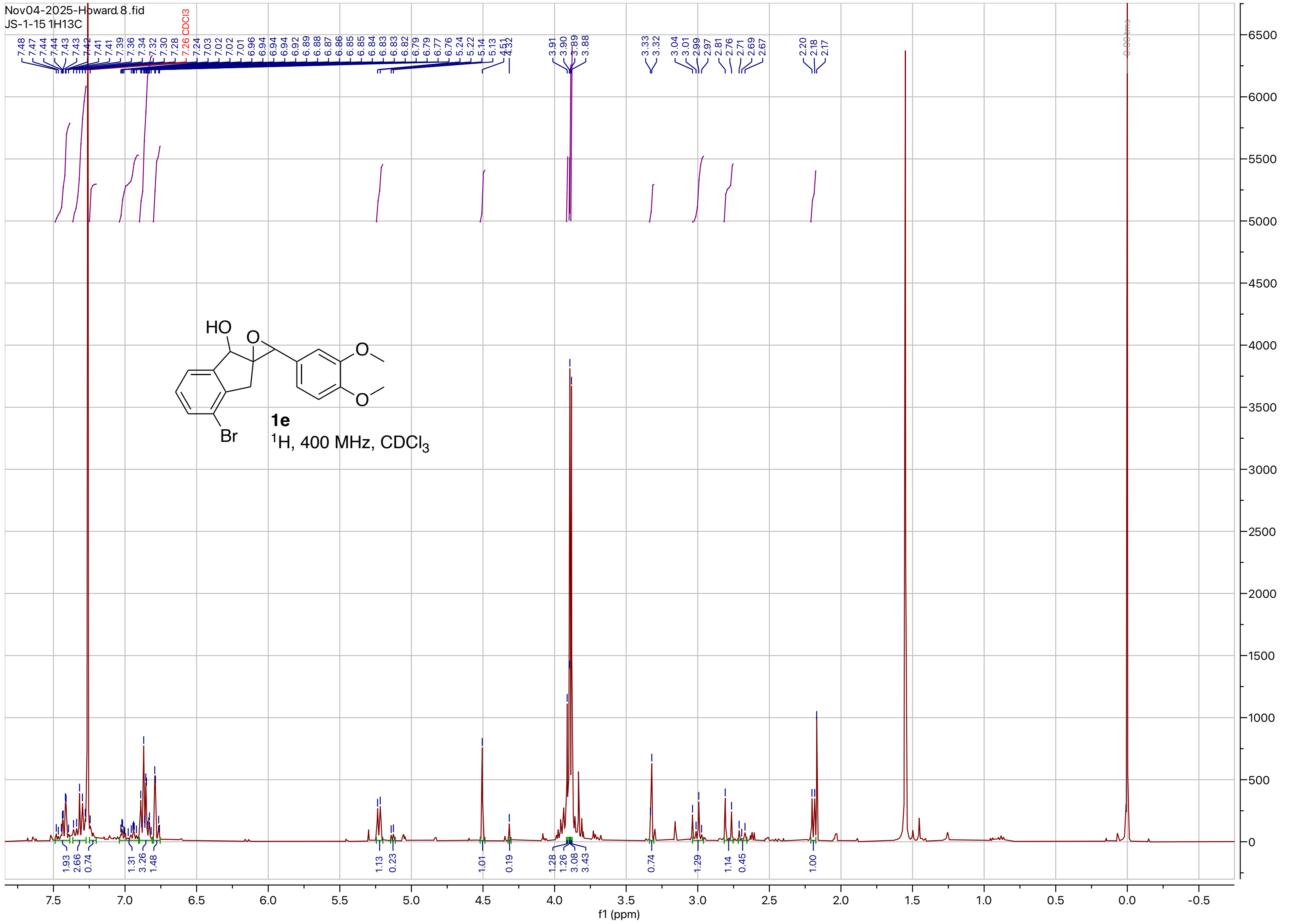

1e
^{1}H, 400 MHz, $CDCl_3$

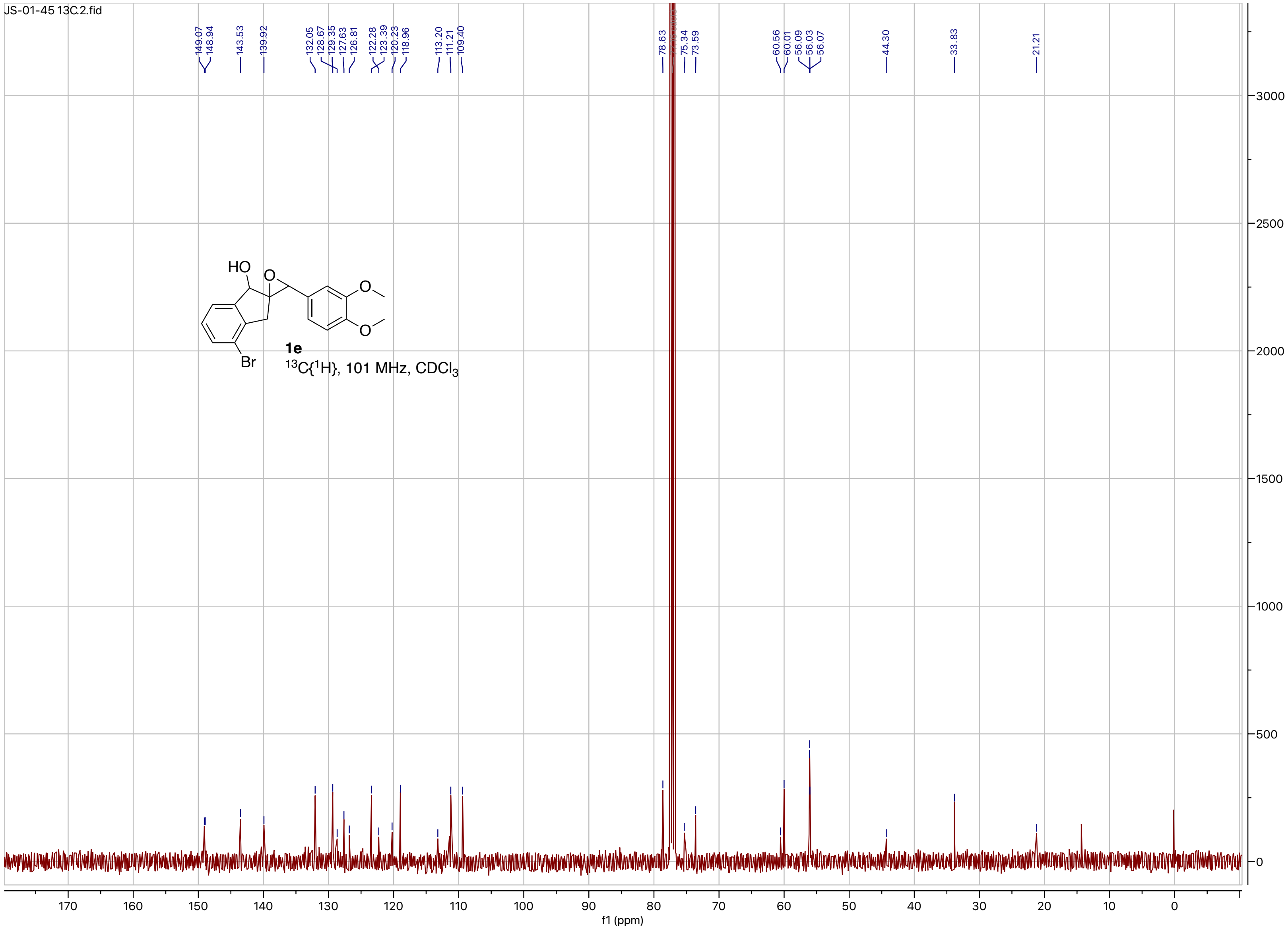
JS-01-45 13C.2.fid
149.07
148.94
143.53
139.92
132.05
128.67
129.35
127.63
126.81
122.28
123.39
120.23
118.96
113.20
111.21
109.40
78.63
75.34
73.59
60.56
60.01
56.09
56.03
56.07
44.30
33.83
21.21
HO
O
O
O
Br
1e
13C{1H}, 101 MHz, CDCl3
f1 (ppm)

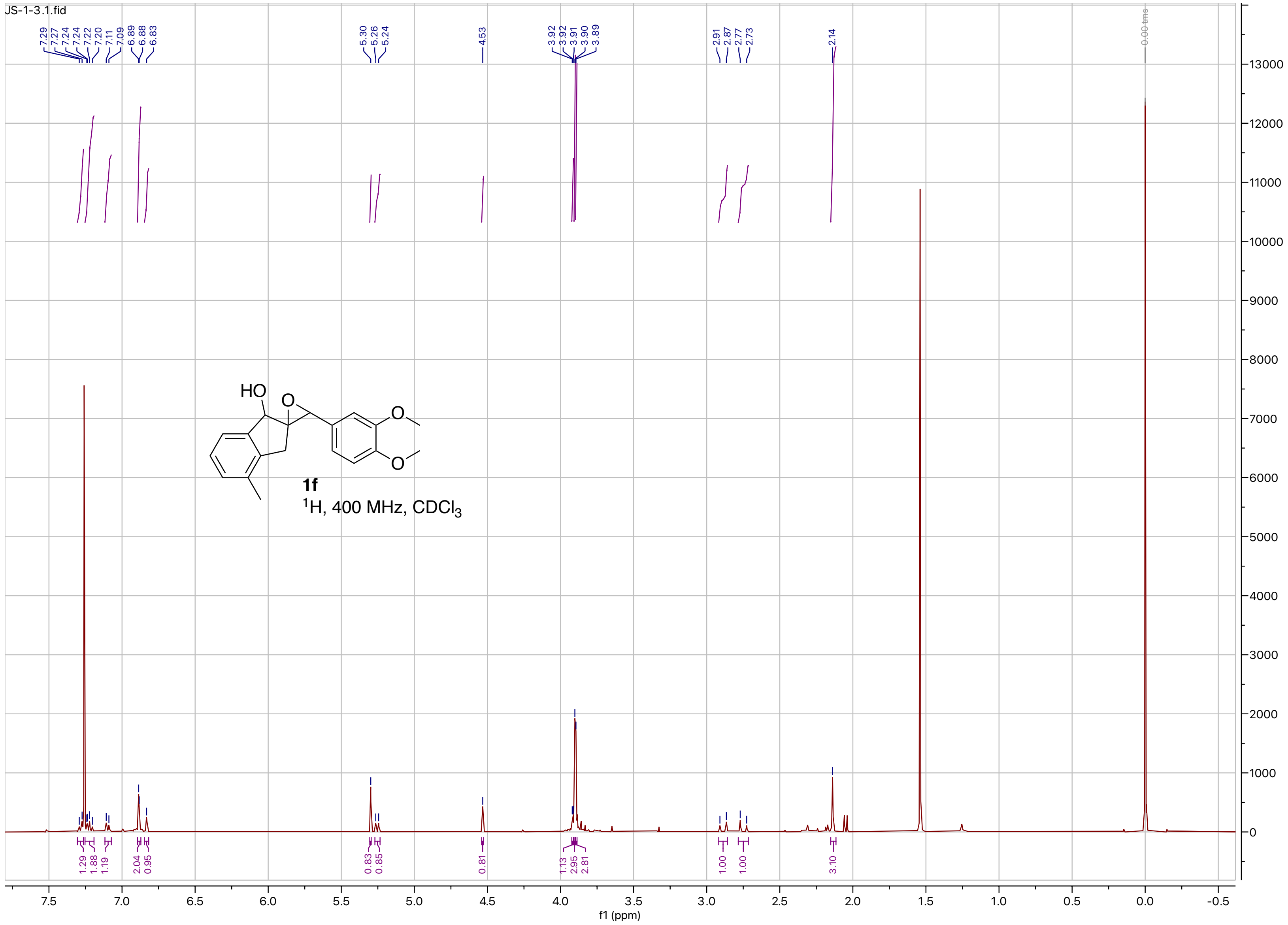
JS-1-3.1.fid
7.29
7.27
7.24
7.24
7.22
7.20
7.11
7.09
6.89
6.88
6.83
5.30
5.26
5.24
4.53
3.92
3.92
3.91
3.90
3.89
2.91
2.87
2.77
2.73
2.14
0.00 tms
HO
O
O
O
1f
1H, 400 MHz, CDCl3
1.29
1.88
1.19
2.04
0.95
0.83
0.85
0.81
1.13
2.95
2.81
1.00
1.00
3.10
f1 (ppm)

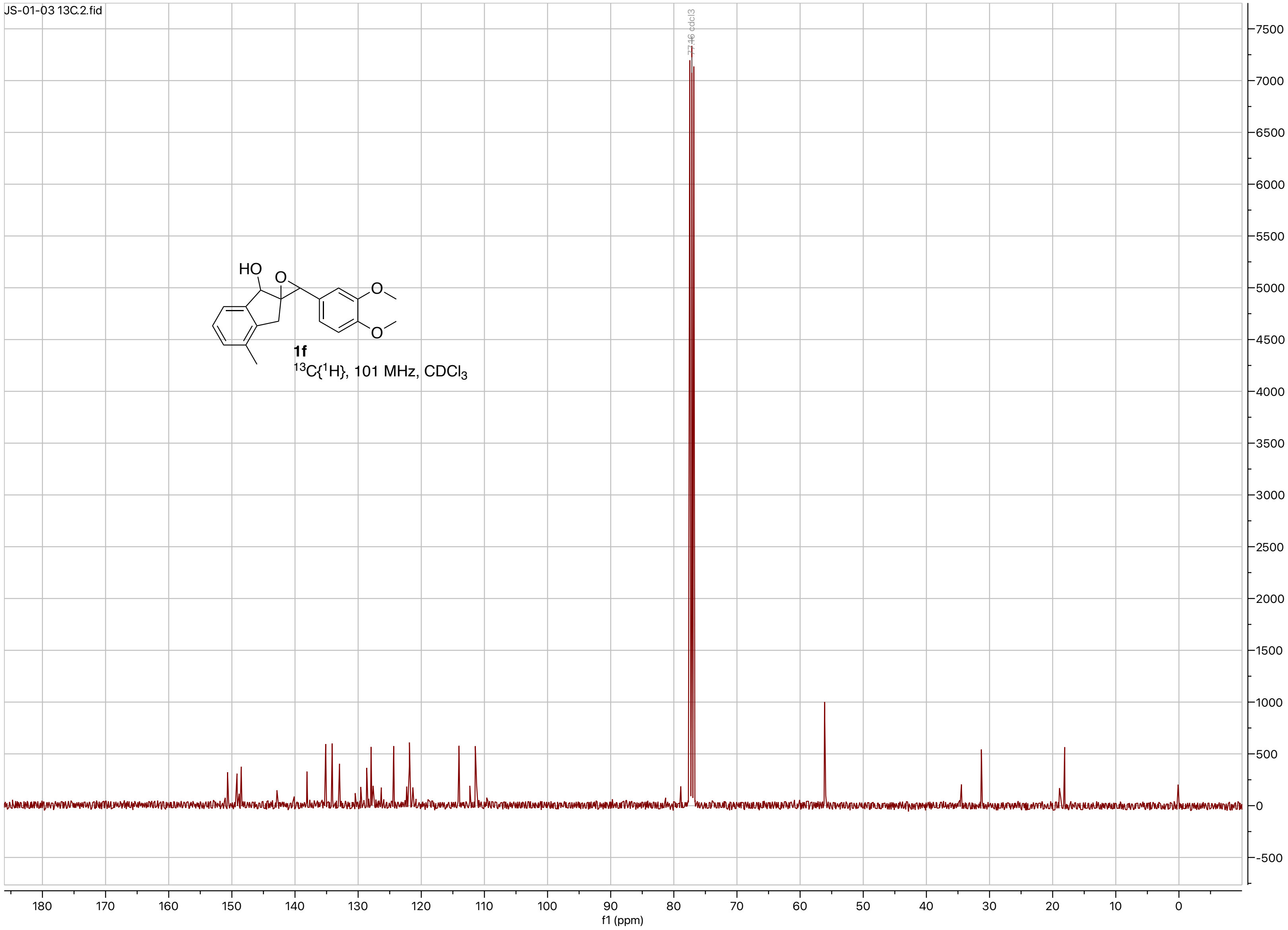
JS-01-03 13C.2.fid
77.16 cdcl3
HO
O
O
O
1f
13C{1H}, 101 MHz, CDCl3
f1 (ppm)

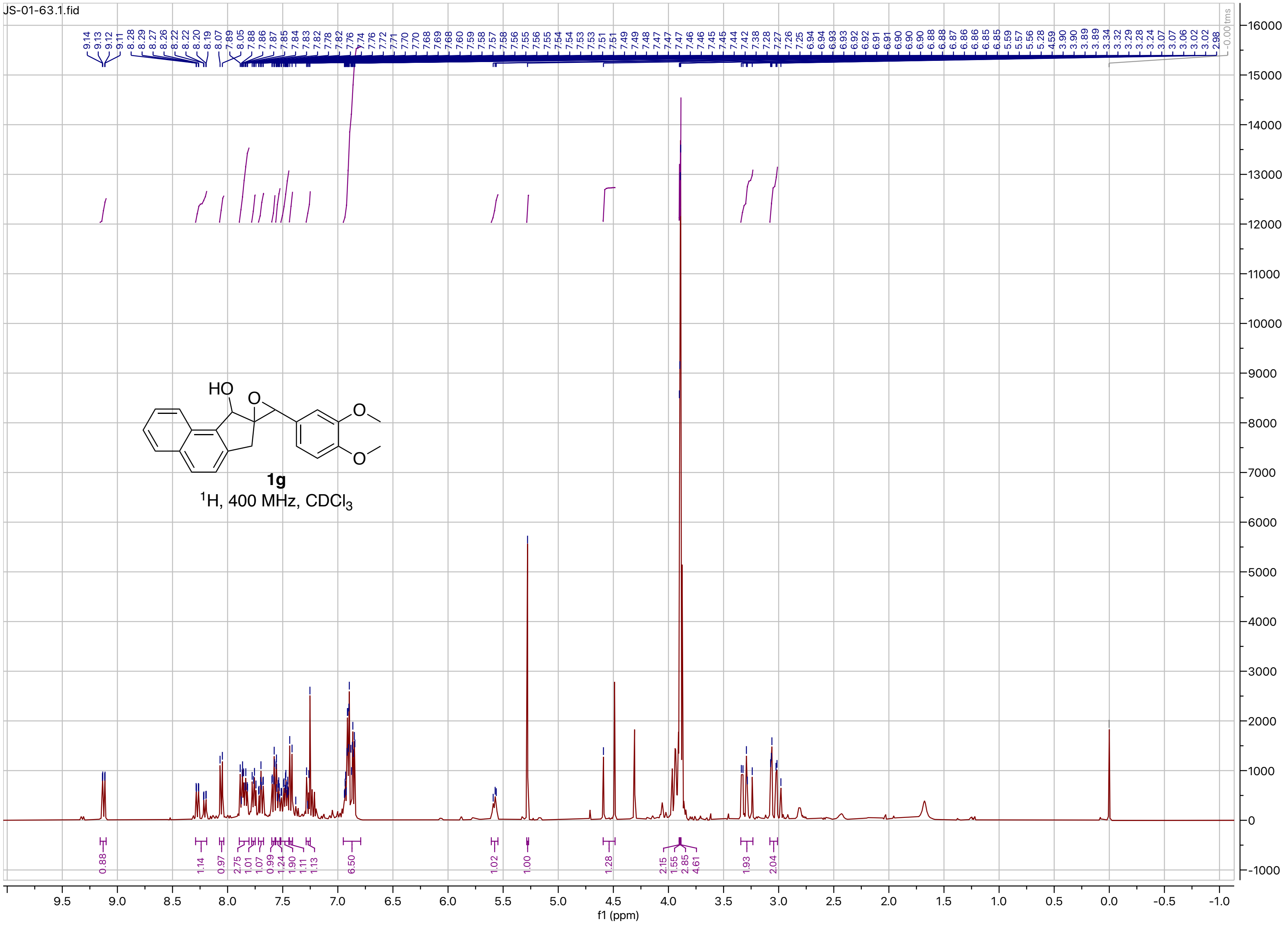
JS-01-63.1.fid
HO
O
O
O
1g
^{1}H, 400 MHz, CDCl$_3$
f1 (ppm)

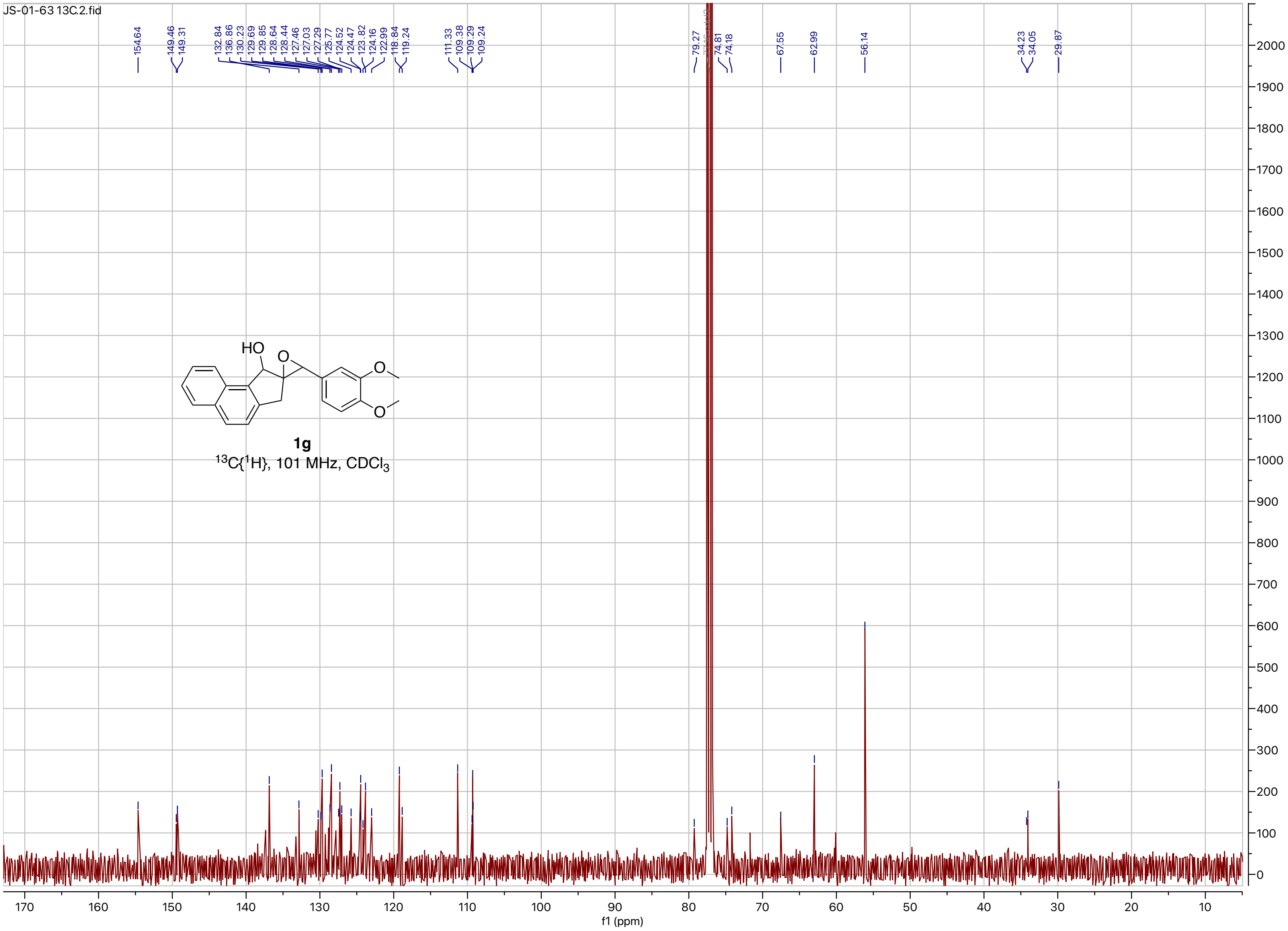
JS-01-63 13C.2.fid
154.64
149.46
149.31
132.84
136.86
130.23
129.69
129.85
128.64
128.44
127.46
127.03
127.29
125.77
124.52
124.47
123.82
124.16
122.99
118.84
119.24
111.33
109.38
109.29
109.24
79.27
74.81
74.18
67.55
62.99
56.14
34.23
34.05
29.87
HO
O
O
O
1g
$^{13}C\{^{1}H\}$, 101 MHz, $CDCl_3$
f1 (ppm)

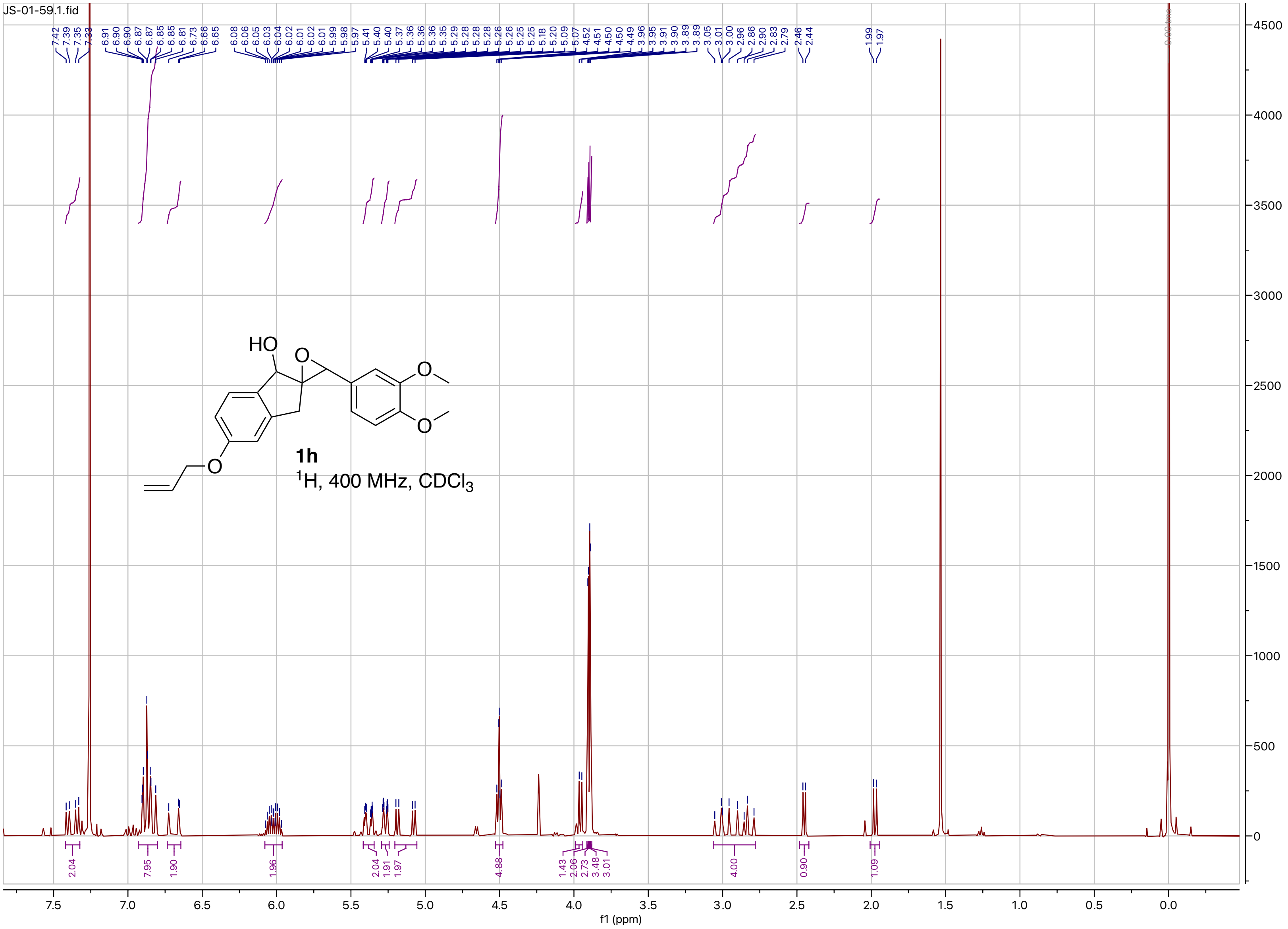
JS-01-59.1.fid
1h
1H, 400 MHz, CDCl3
HO
O
f1 (ppm)

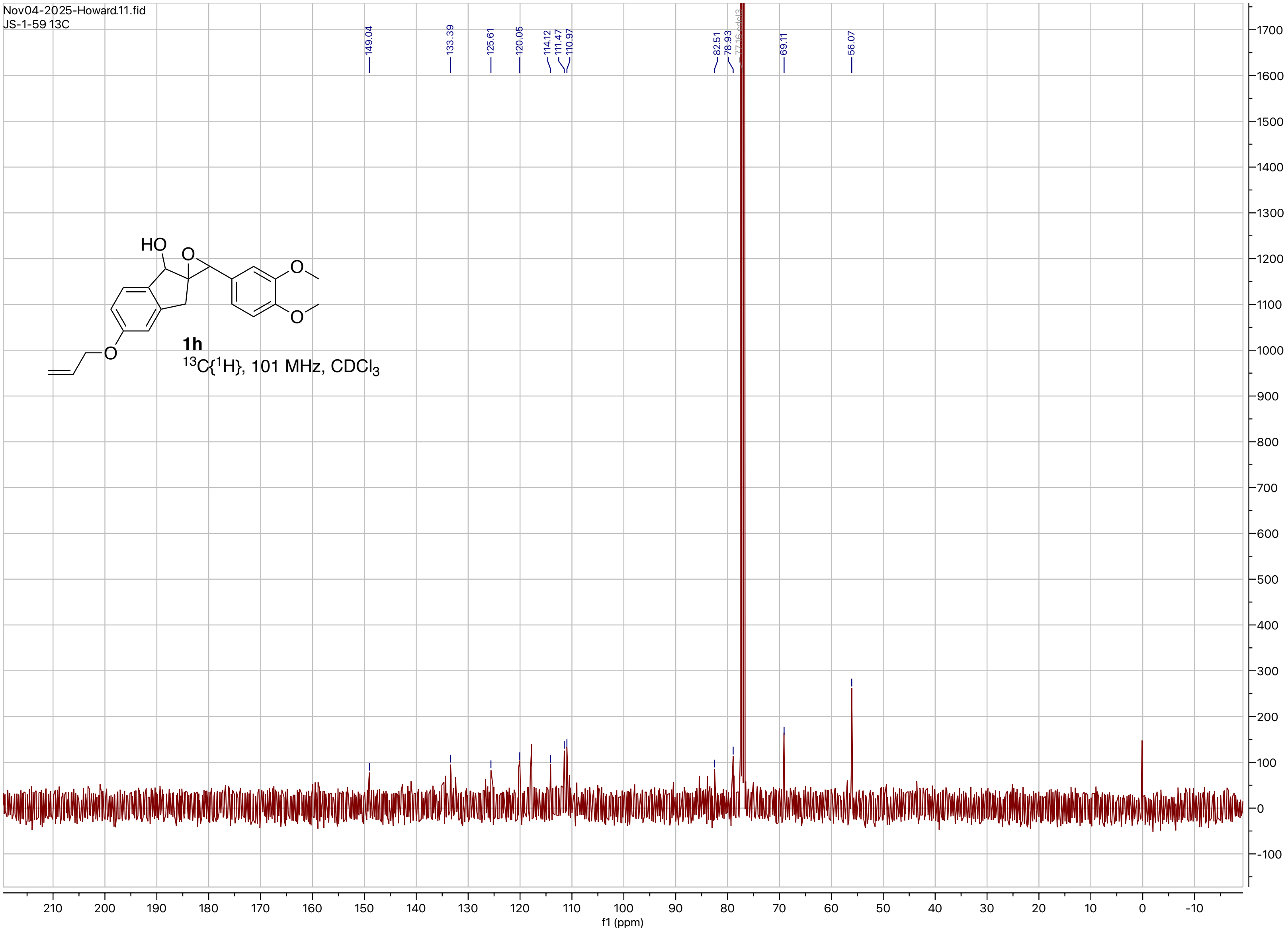
Nov04-2025-Howard.11.fid
JS-1-59 13C
149.04
133.39
125.61
120.05
114.12
111.47
110.97
82.51
78.93
69.11
56.07
HO
O
1h
$^{13}C\{^{1}H\}$, 101 MHz, $CDCl_3$
f1 (ppm)

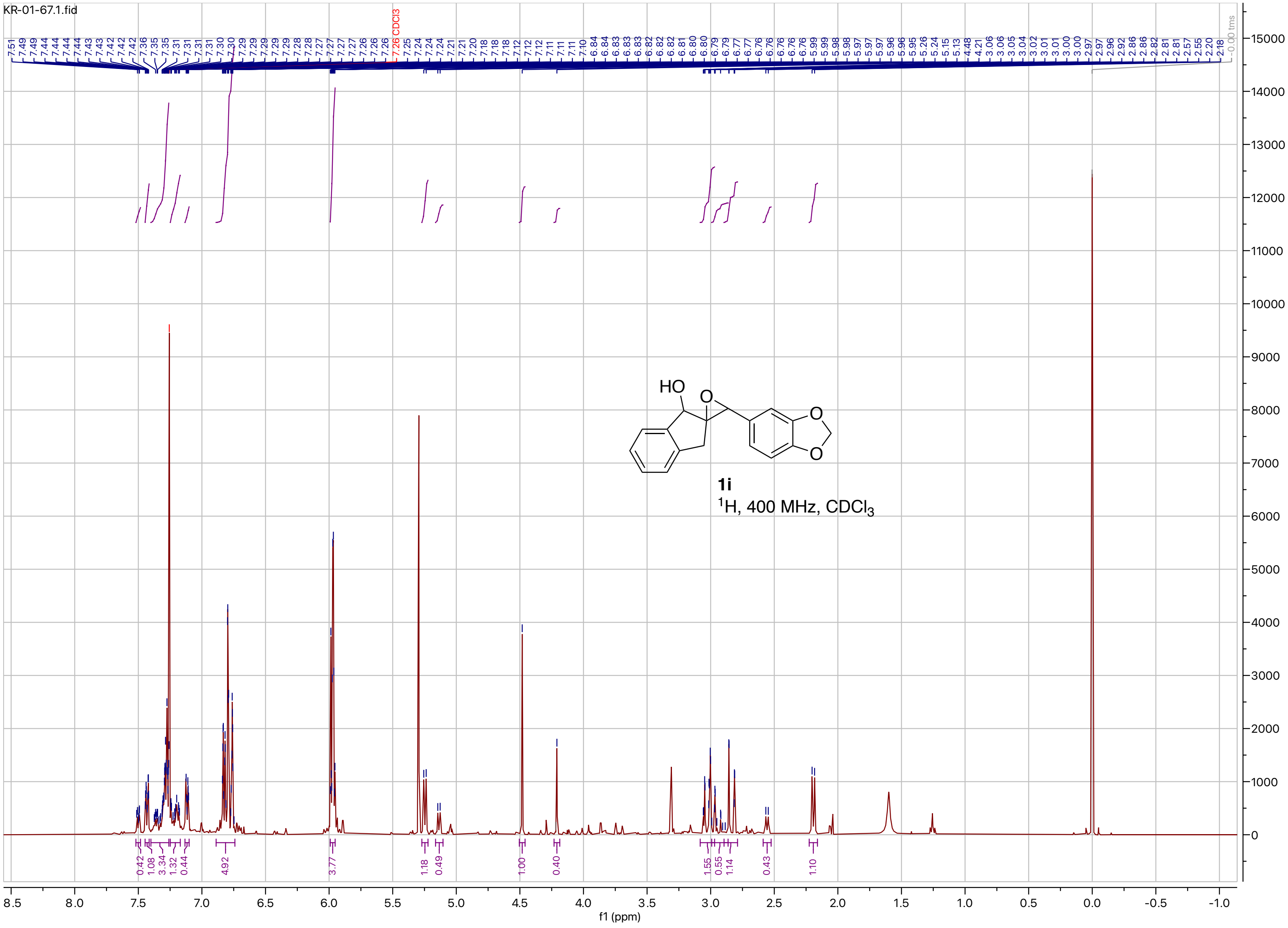
KR-01-67.1.fid
1i
1H, 400 MHz, CDCl3
f1 (ppm)
HO
O
O
O

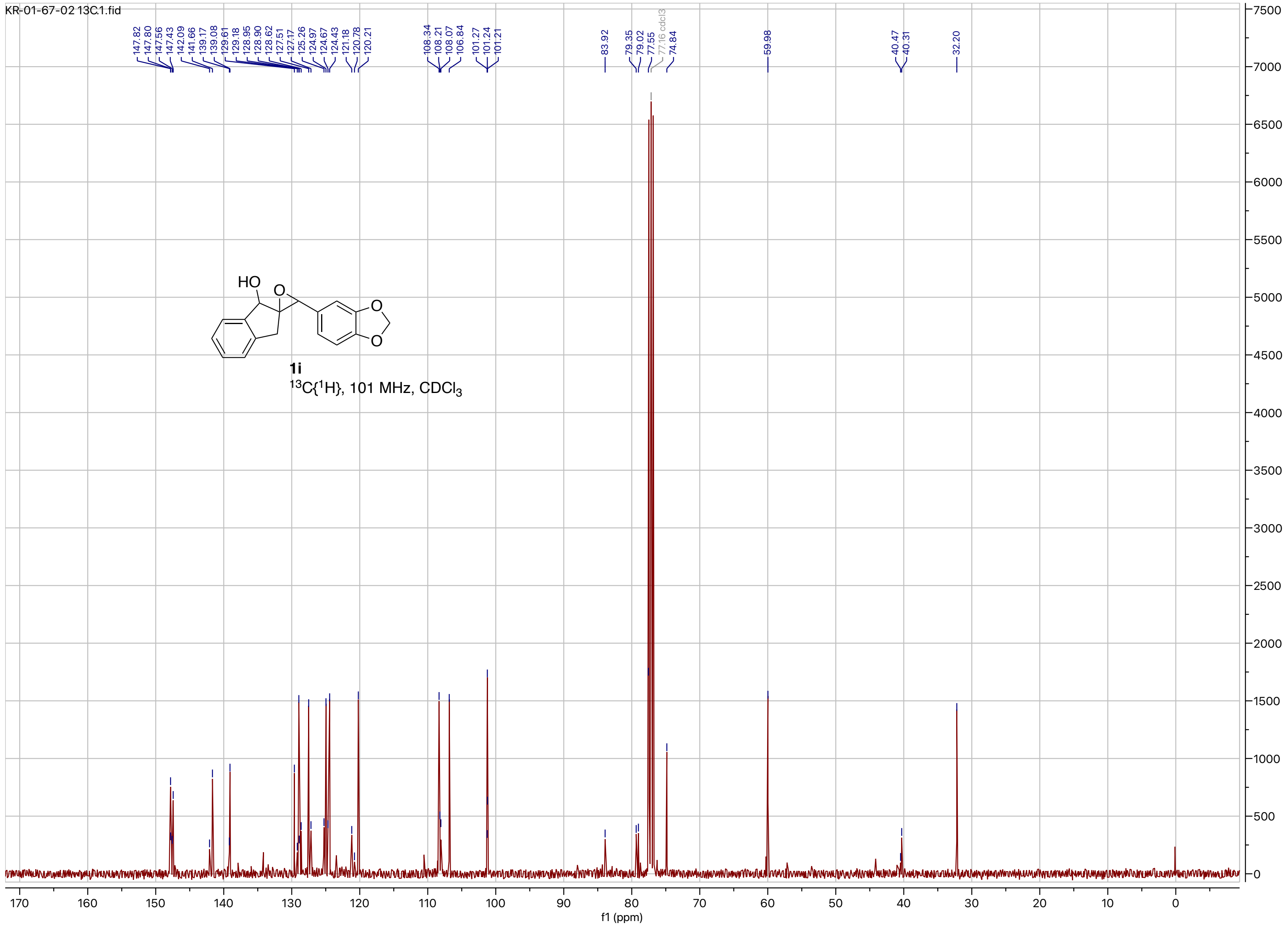

KR-01-67-02 13C.1.fid
147.82
147.80
147.56
147.43
142.09
141.66
139.17
139.08
129.61
129.18
128.95
128.90
128.62
127.51
127.17
125.26
124.97
124.67
124.43
121.18
120.78
120.21
108.34
108.21
108.07
106.84
101.27
101.24
101.21
83.92
79.35
79.02
77.55
77.16 cdcl3
74.84
59.98
40.47
40.31
32.20
HO
O
O
O
1i
13C{1H}, 101 MHz, CDCl3
f1 (ppm)

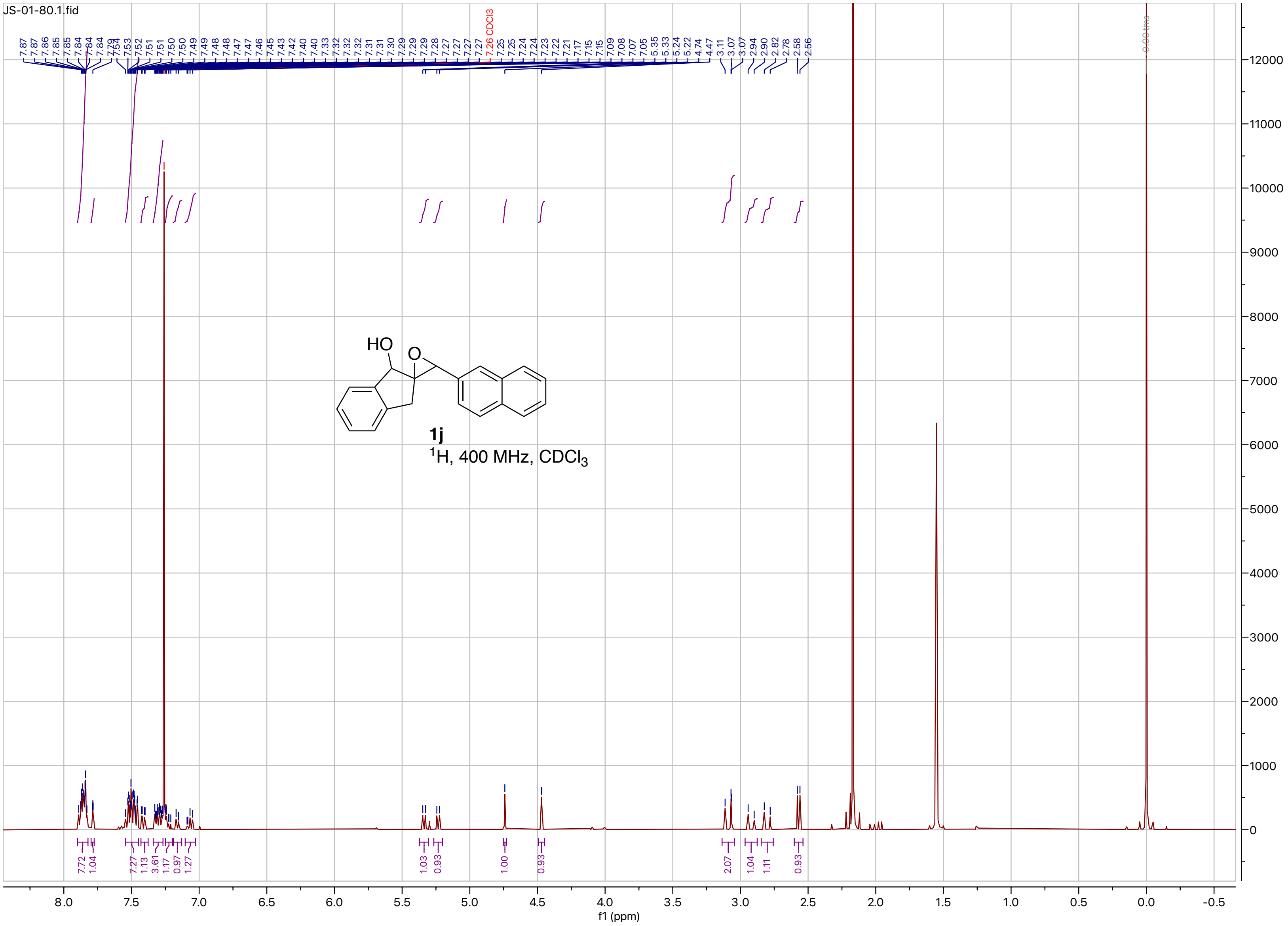
JS-01-80.1.fid
1j
1H, 400 MHz, CDCl3
f1 (ppm)

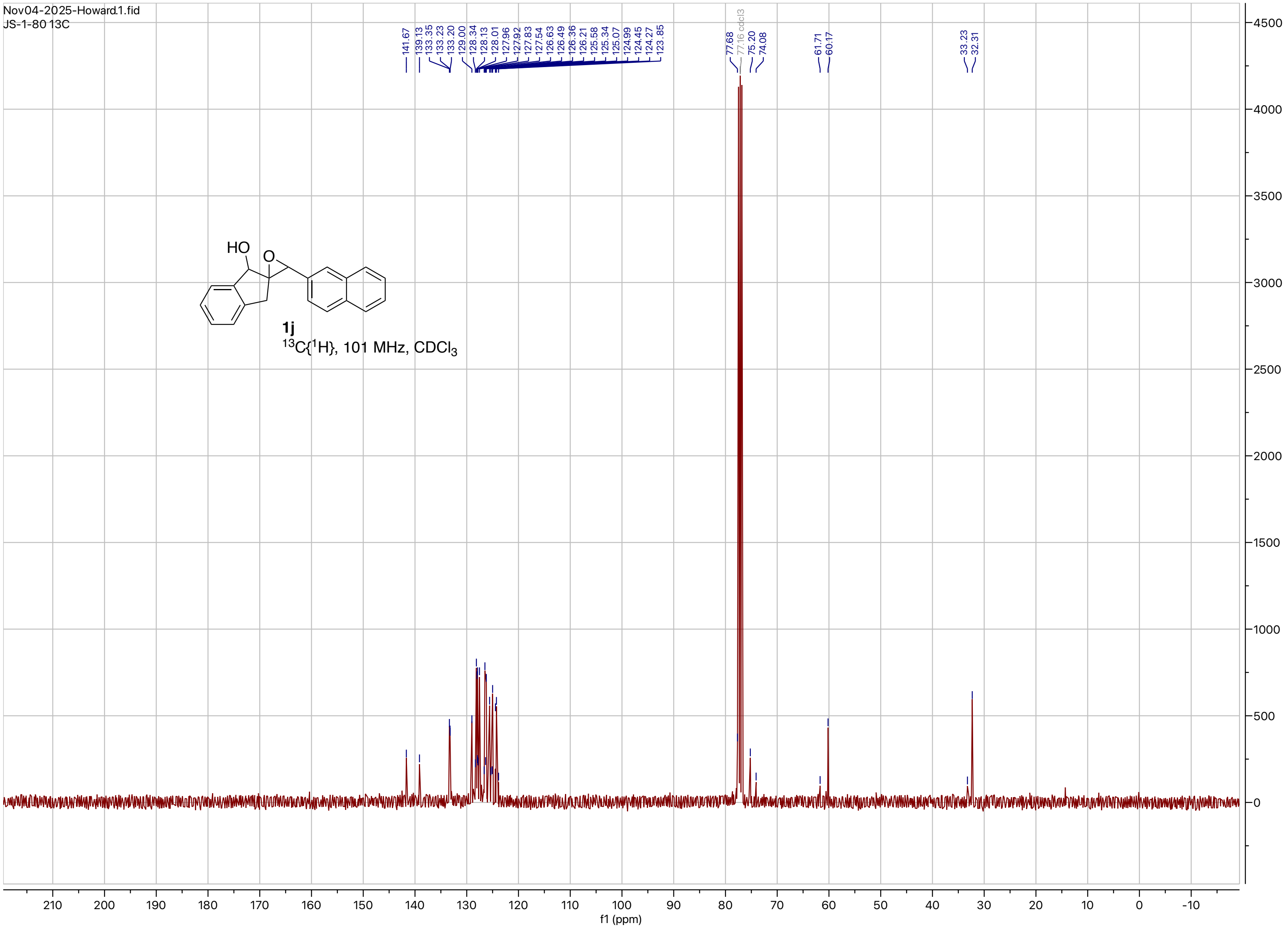
Nov04-2025-Howard.1.fid
JS-1-80 13C
141.67
139.13
133.35
133.23
133.20
129.00
128.34
128.13
128.01
127.96
127.92
127.83
127.54
126.63
126.49
126.36
126.21
125.58
125.34
125.07
124.99
124.45
124.27
123.85
77.68
77.16 cdcl3
75.20
74.08
61.71
60.17
33.23
32.31
HO
O
1j
13C{1H}, 101 MHz, CDCl3
f1 (ppm)

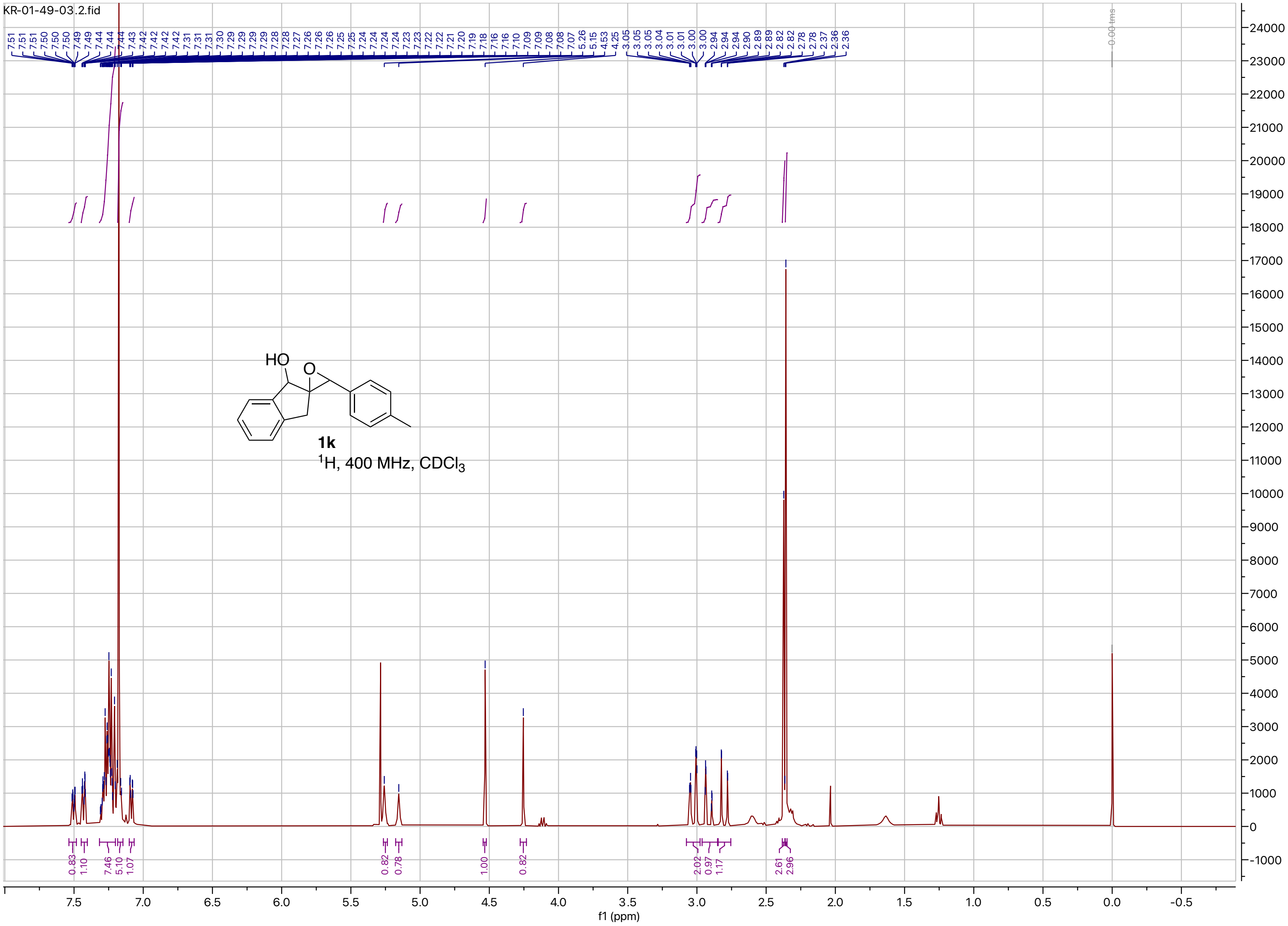
KR-01-49-03.2.fid
HO
O
1k
^{1}H, 400 MHz, $CDCl_3$
f1 (ppm)

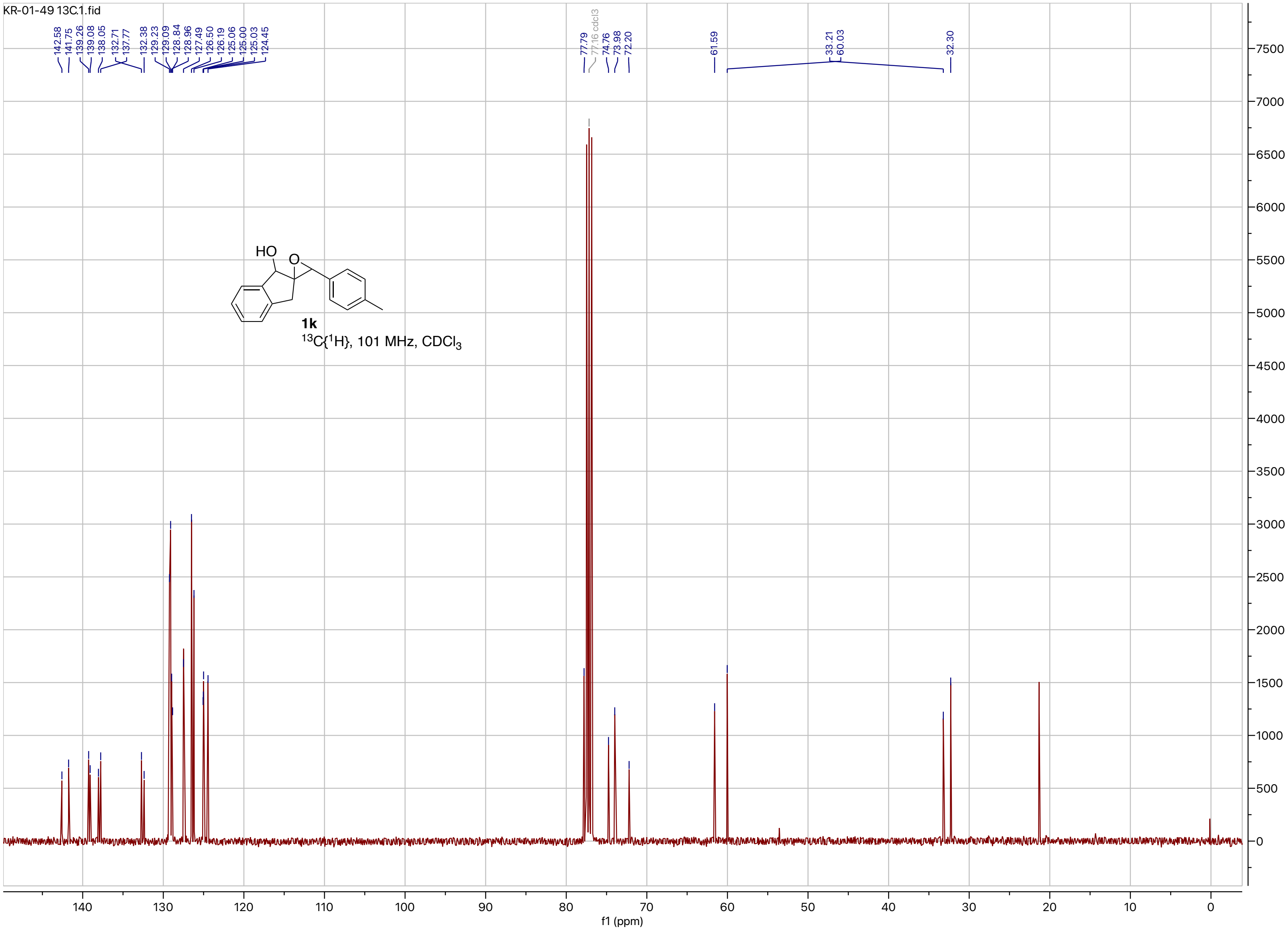
KR-01-49 13C.1.fid
142.58
141.75
139.26
139.08
138.05
132.71
137.77
132.38
129.23
129.09
128.84
128.96
127.49
126.50
126.19
125.06
125.00
125.03
124.45
77.79
77.16 cdcl3
74.76
73.98
72.20
61.59
33.21
60.03
32.30
HO
O
1k
$^{13}C\{^1H\}$, 101 MHz, $CDCl_3$
f1 (ppm)

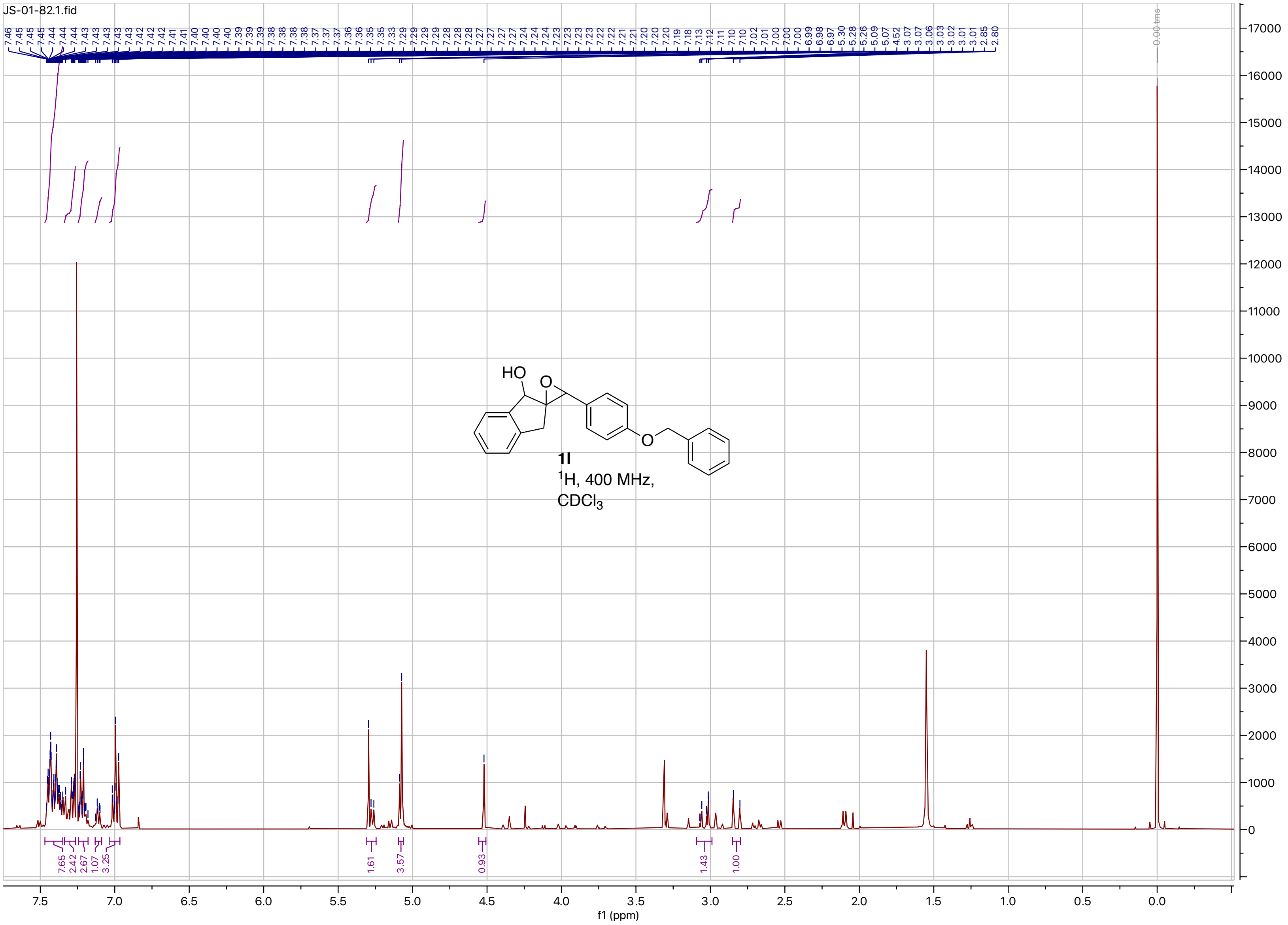
JS-01-82.1.fid
HO
O
O
1l
1H, 400 MHz,
CDCl3
f1 (ppm)
7.65
2.42
2.67
1.07
3.25
1.61
3.57
0.93
1.43
1.00

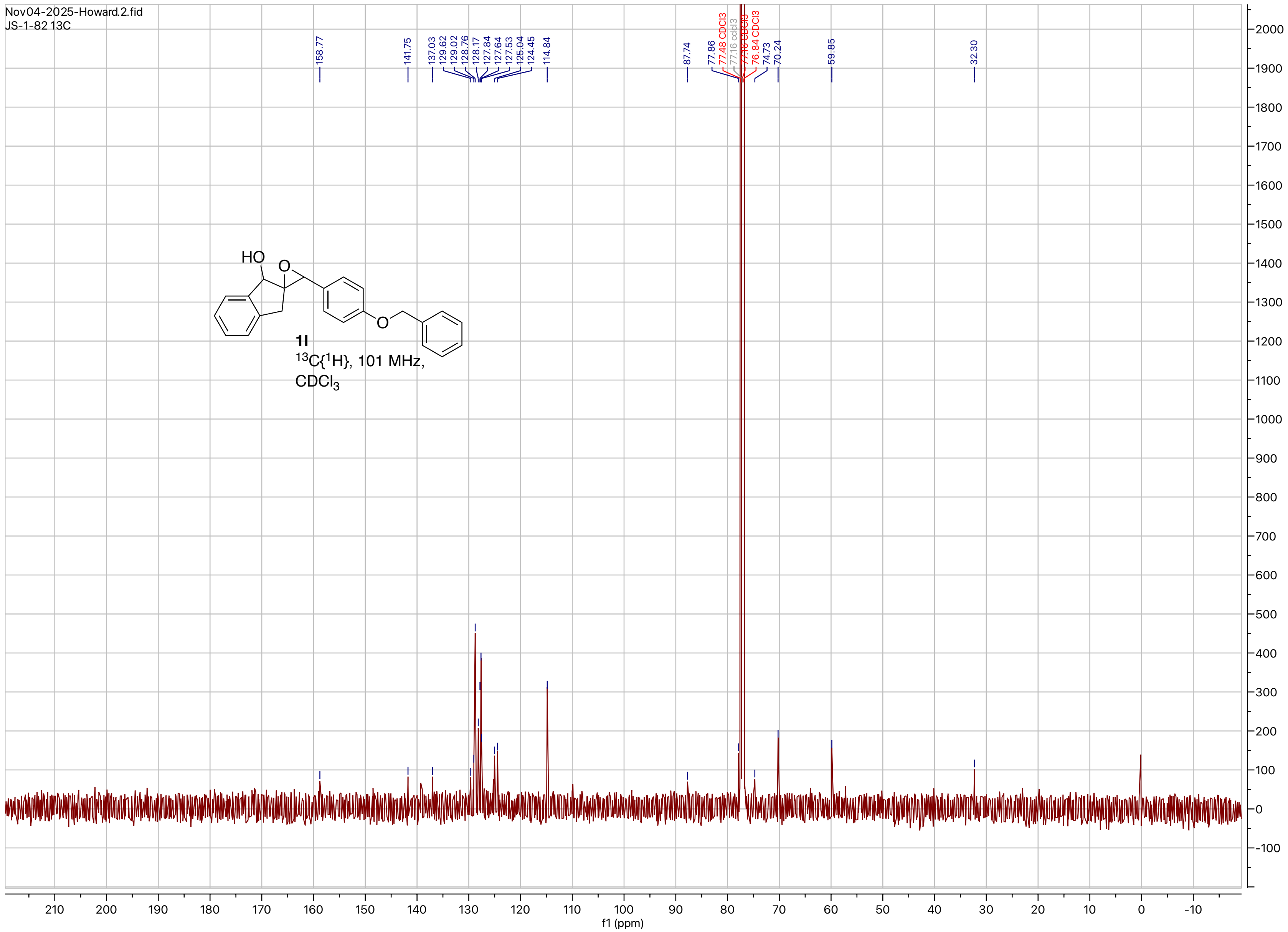
Nov04-2025-Howard.2.fid
JS-1-82 13C
158.77
141.75
137.03
129.62
129.02
128.76
128.17
127.84
127.64
127.53
125.04
124.45
114.84
87.74
77.86
77.48 CDCl3
77.16 cdcl3
77.16 CDCl3
76.84 CDCl3
74.73
70.24
59.85
32.30
HO
O
O
1l
13C{1H}, 101 MHz,
CDCl3
f1 (ppm)

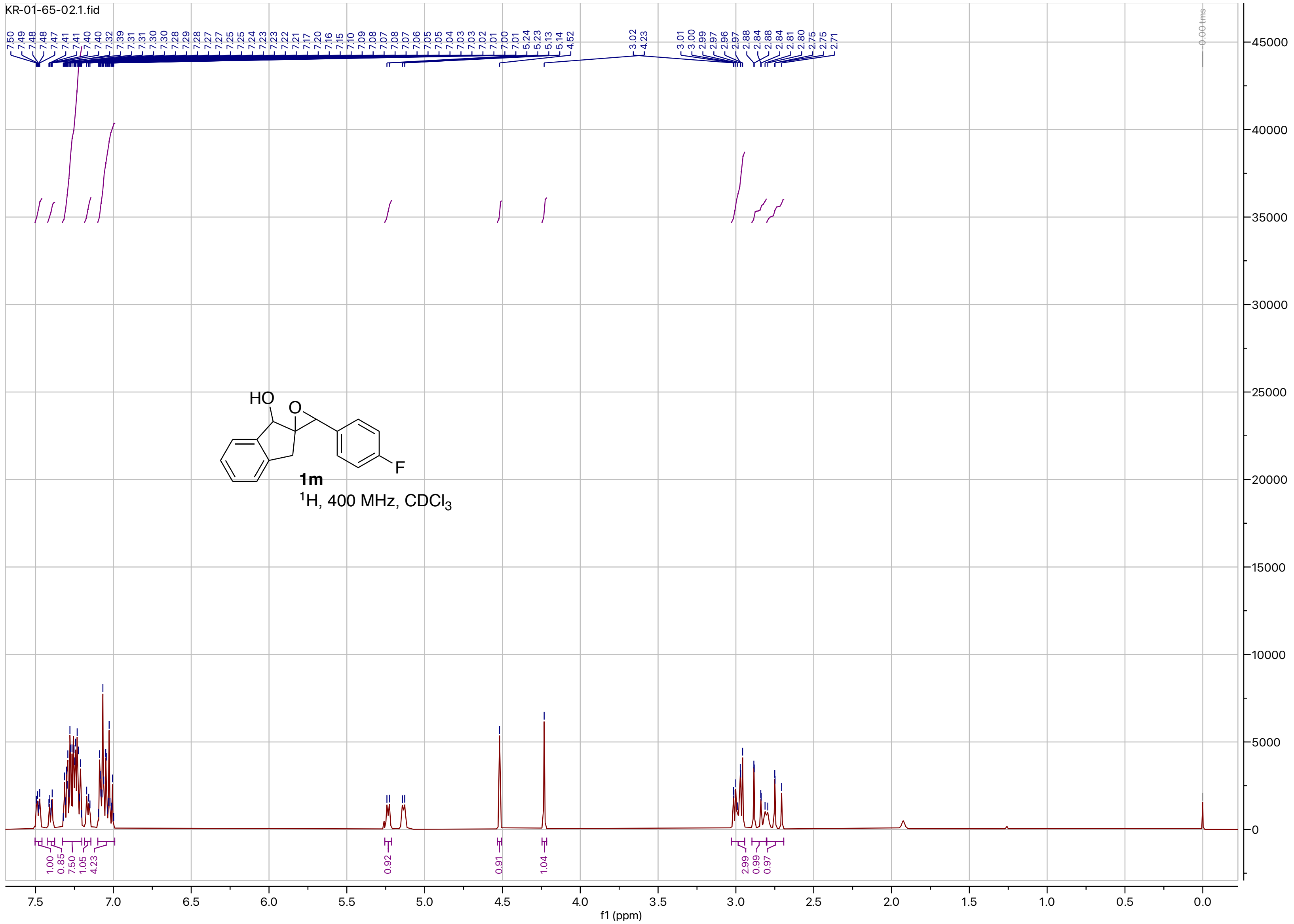
KR-01-65-02.1.fid
HO
O
F
1m
^{1}H, 400 MHz, CDCl$_3$
f1 (ppm)

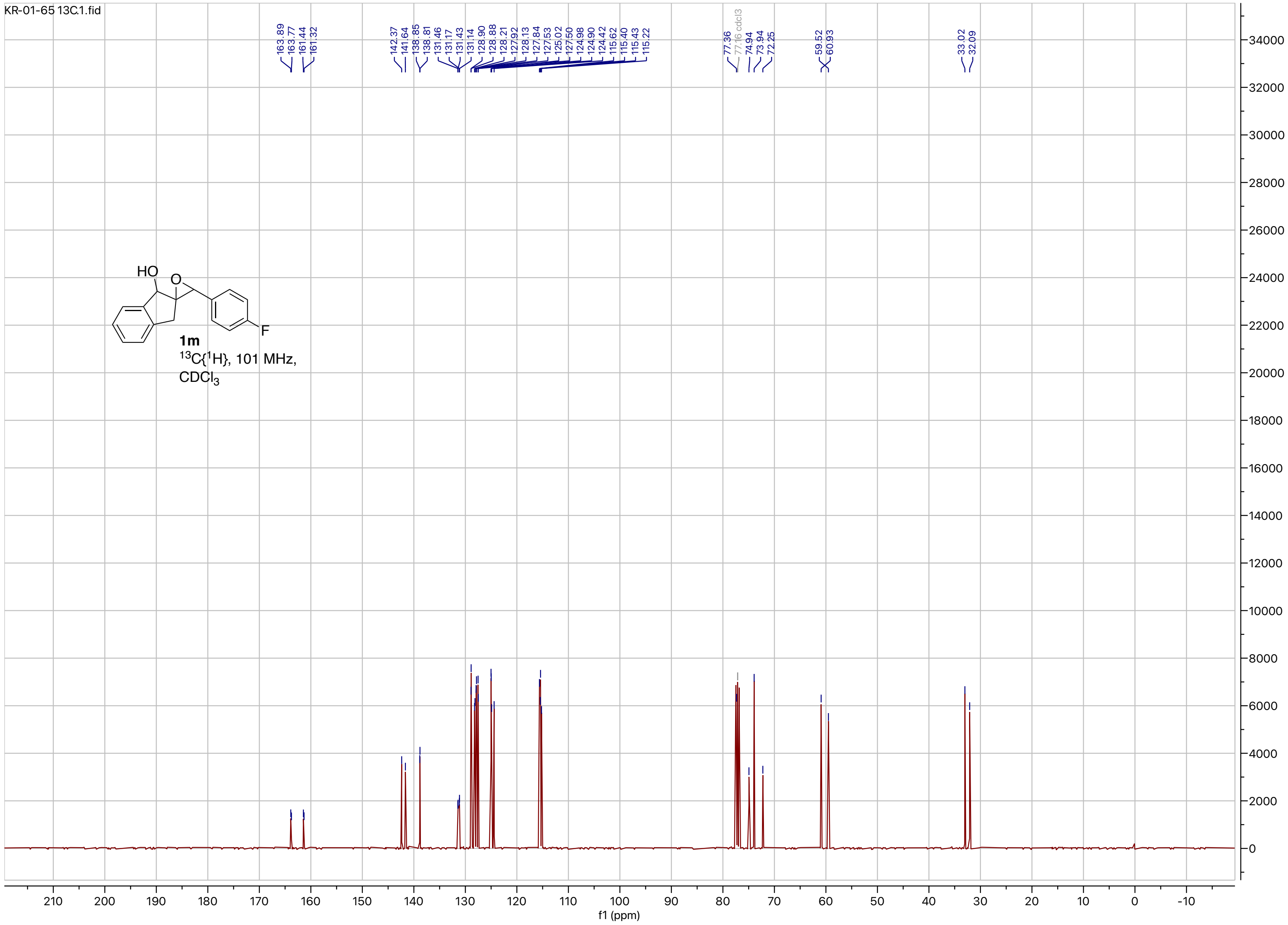
KR-01-65 13C.1.fid
163.89
163.77
161.44
161.32
142.37
141.64
138.85
138.81
131.46
131.17
131.43
131.14
128.90
128.88
128.21
127.92
128.13
127.84
127.53
125.02
127.50
124.98
124.90
124.42
115.62
115.40
115.43
115.22
77.36
77.16 cdcl3
74.94
73.94
72.25
59.52
60.93
33.02
32.09
HO
O
F
1m
13C{1H}, 101 MHz,
CDCl3
f1 (ppm)

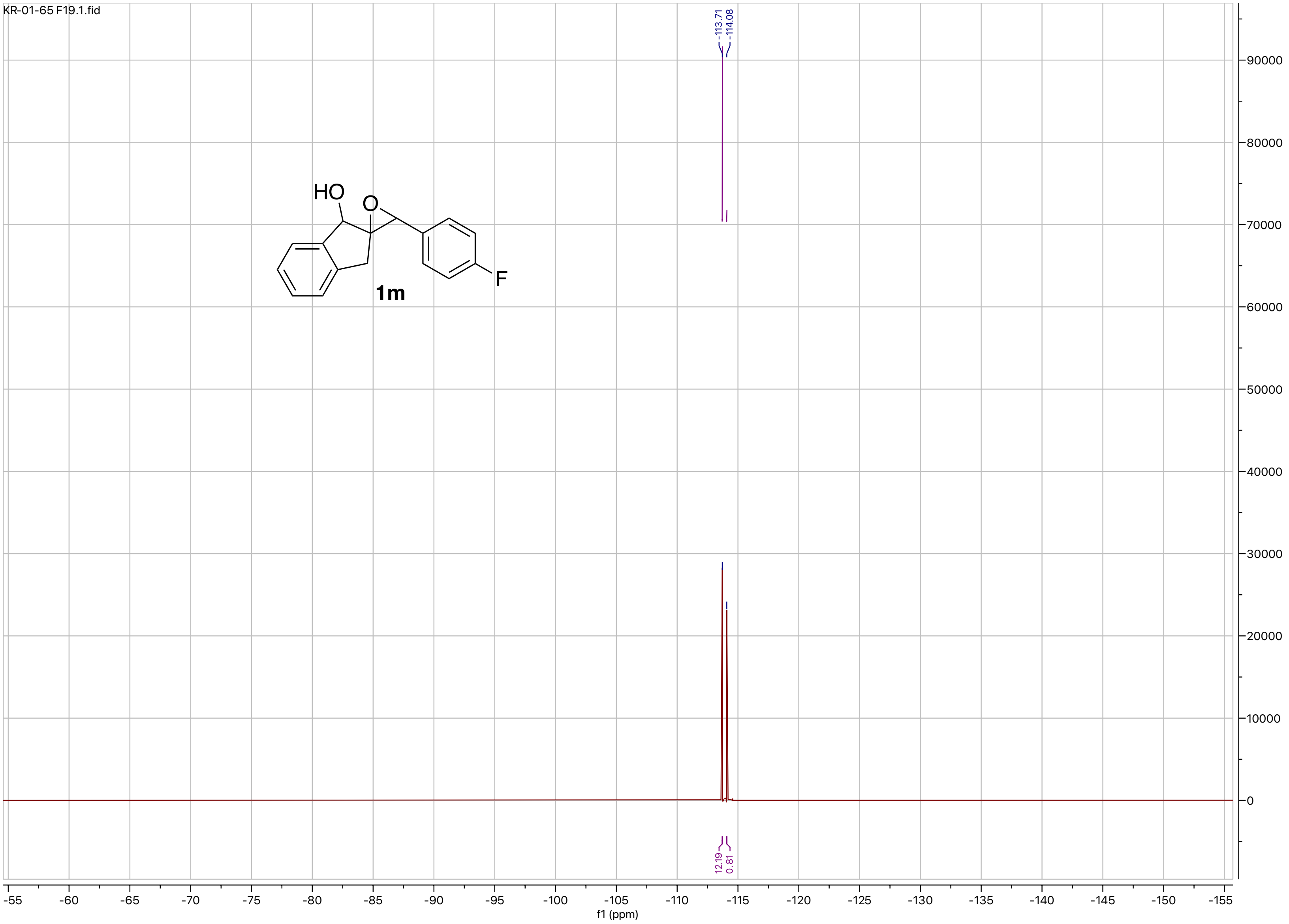
KR-01-65 F19.1.fid
-113.71
-114.08
HO
O
F
1m
12.19
0.81
f1 (ppm)

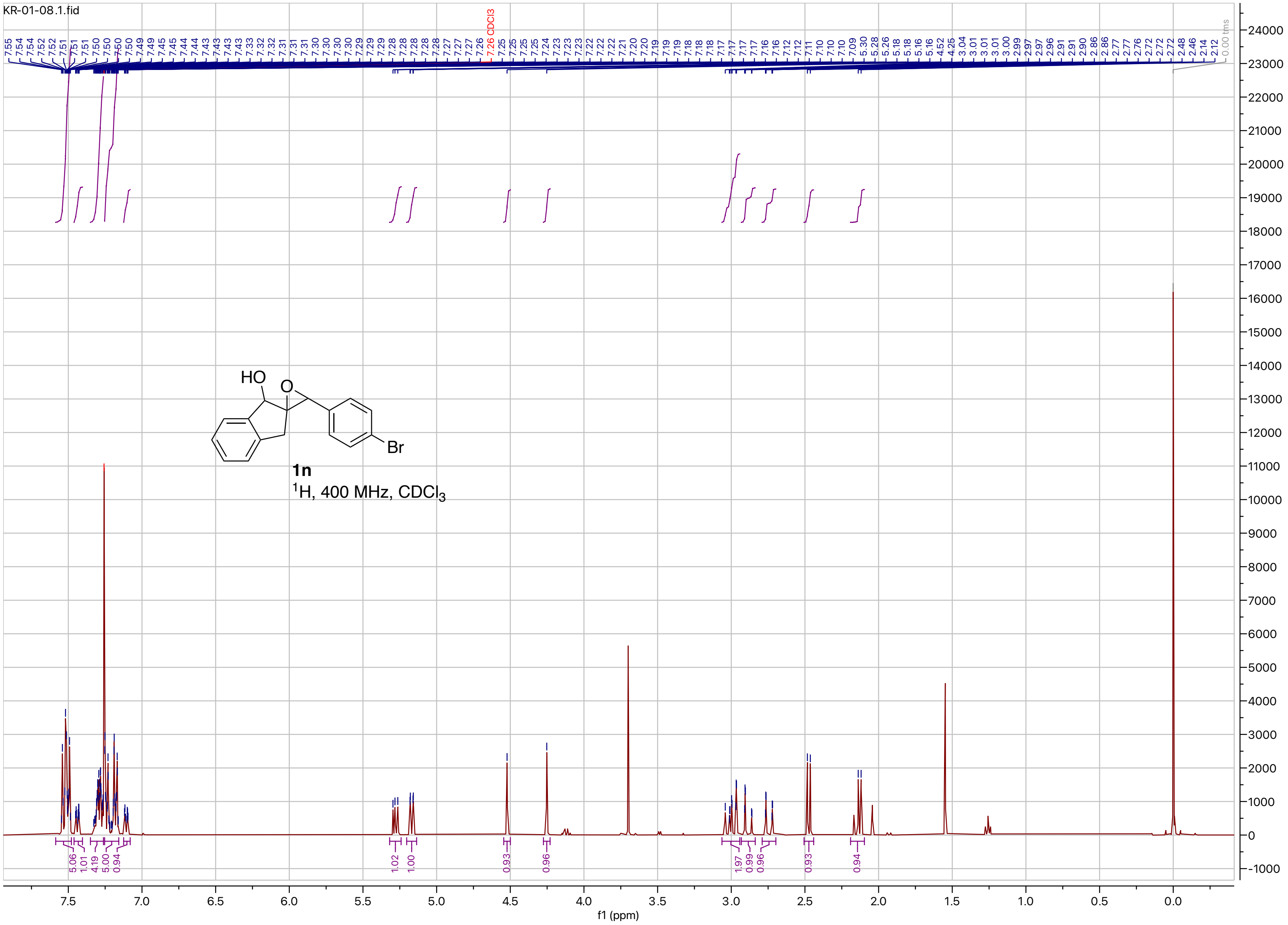

1n
^1H, 400 MHz, $CDCl_3$

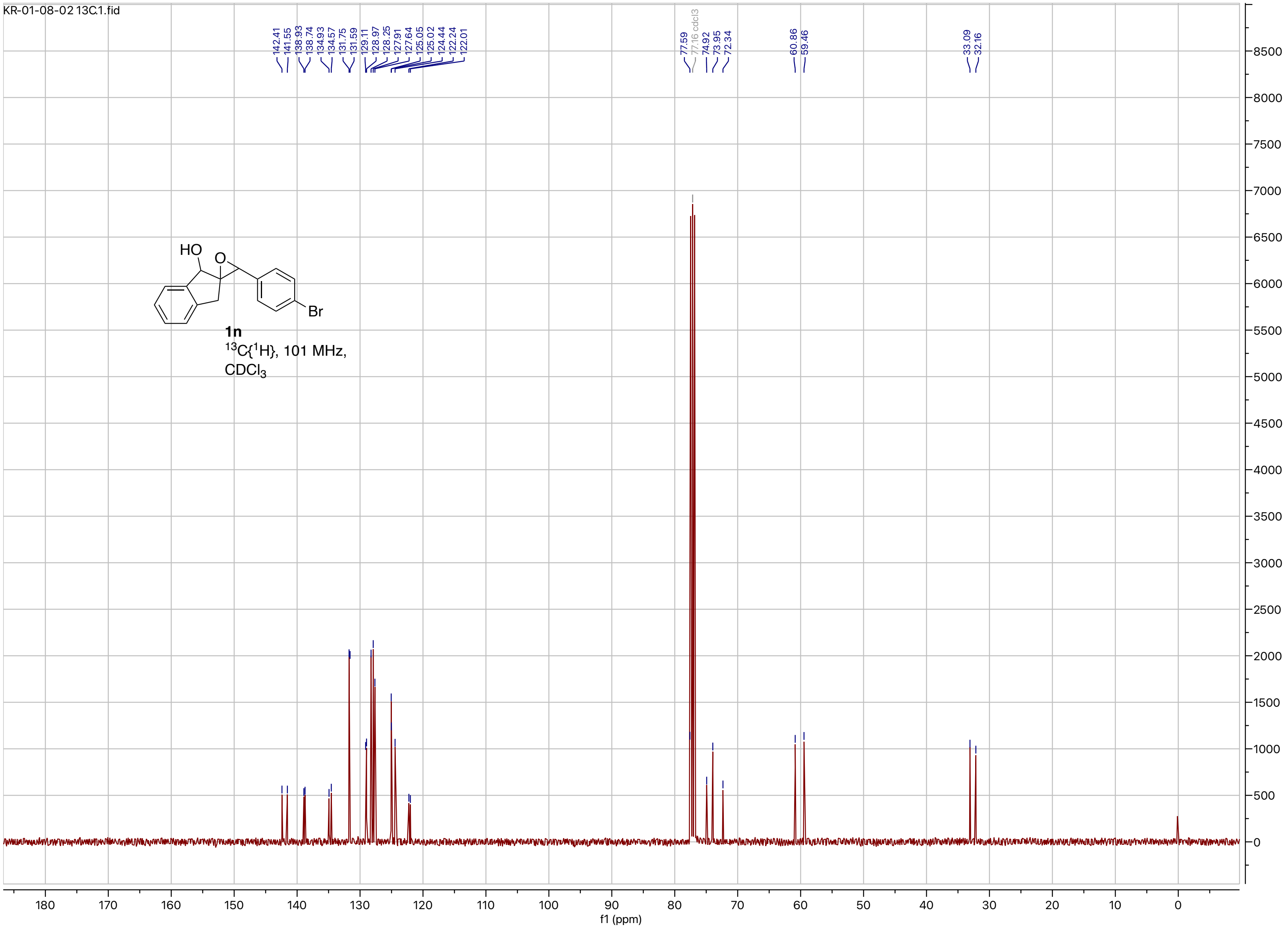
KR-01-08-02 13C1.fid
142.41
141.55
138.93
138.74
134.93
134.57
131.75
131.59
129.11
128.97
128.25
127.91
127.64
125.05
125.02
124.44
122.24
122.01
77.59
77.16 cdcl3
74.92
73.95
72.34
60.86
59.46
33.09
32.16
HO
O
Br
1n
$^{13}C\{^1H\}$, 101 MHz,
$CDCl_3$
f1 (ppm)

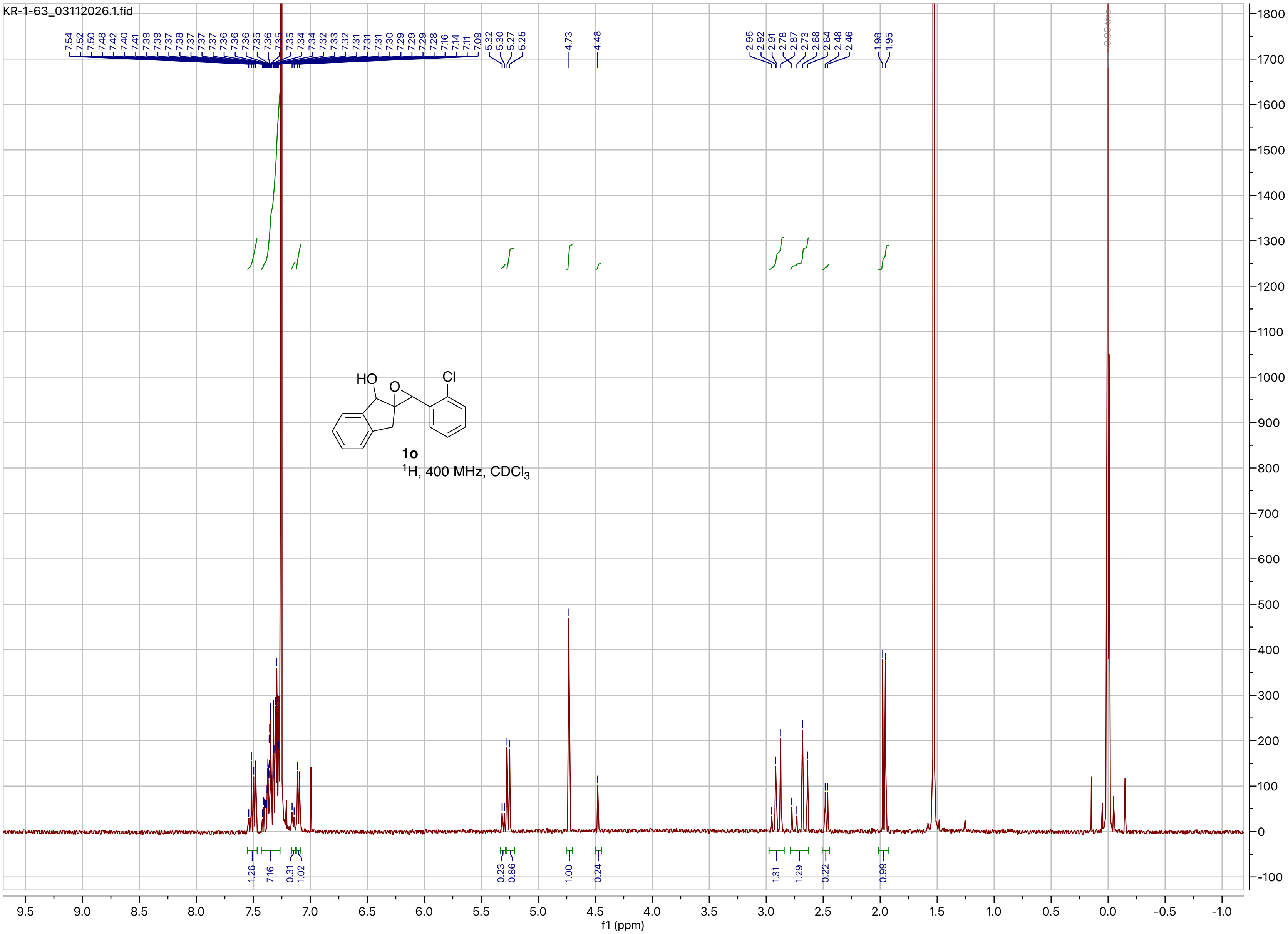
KR-1-63_03112026.1.fid
HO
O
Cl
1o
1H, 400 MHz, CDCl3
f1 (ppm)

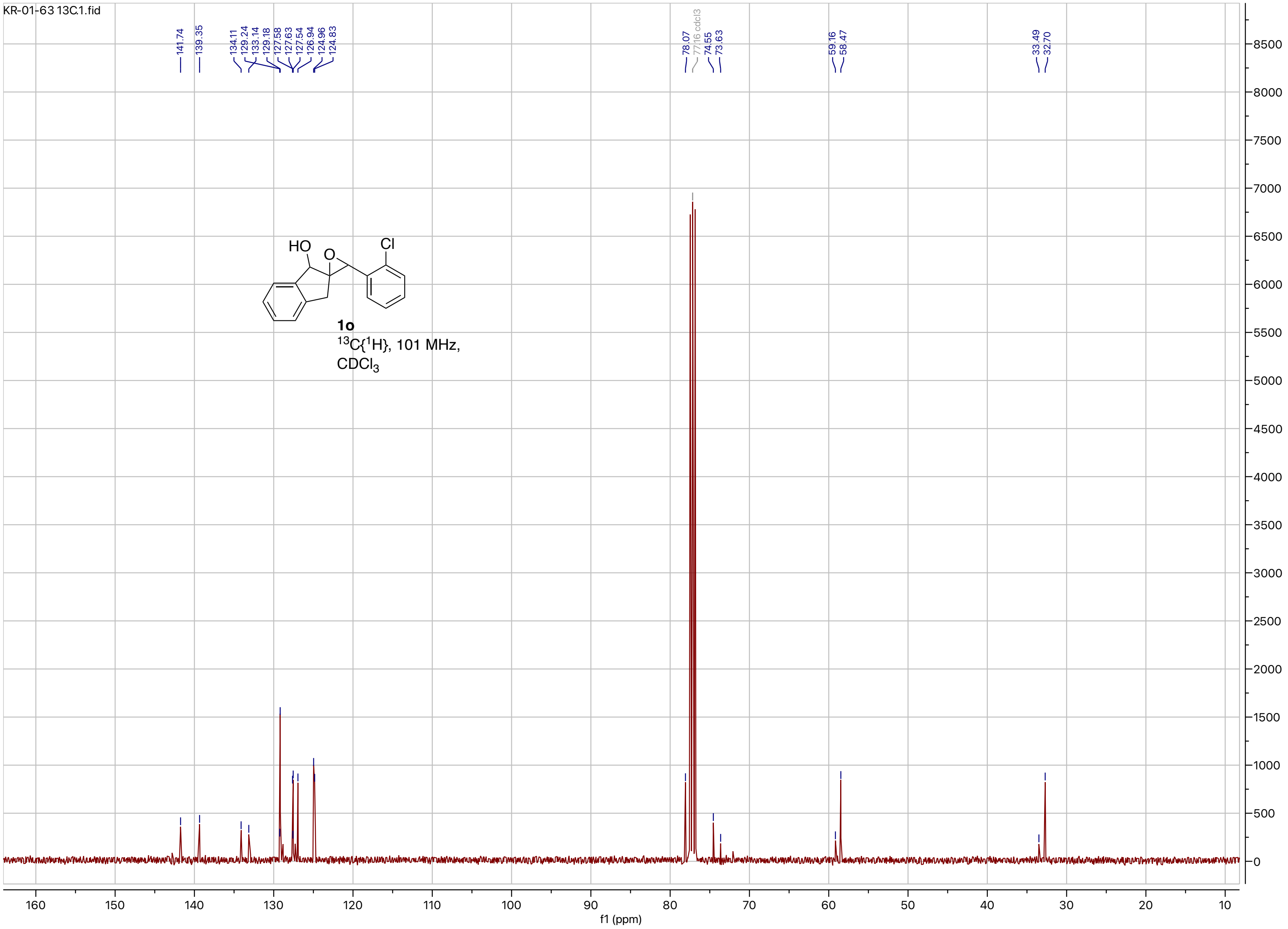
KR-01-63 13C1.fid
141.74
139.35
134.11
129.24
133.14
129.18
127.58
127.63
127.54
126.94
124.96
124.83
78.07
77.16 cdcl3
74.55
73.63
59.16
58.47
33.49
32.70
HO
O
Cl
1o
$^{13}C\{^{1}H\}$, 101 MHz,
$CDCl_3$
f1 (ppm)

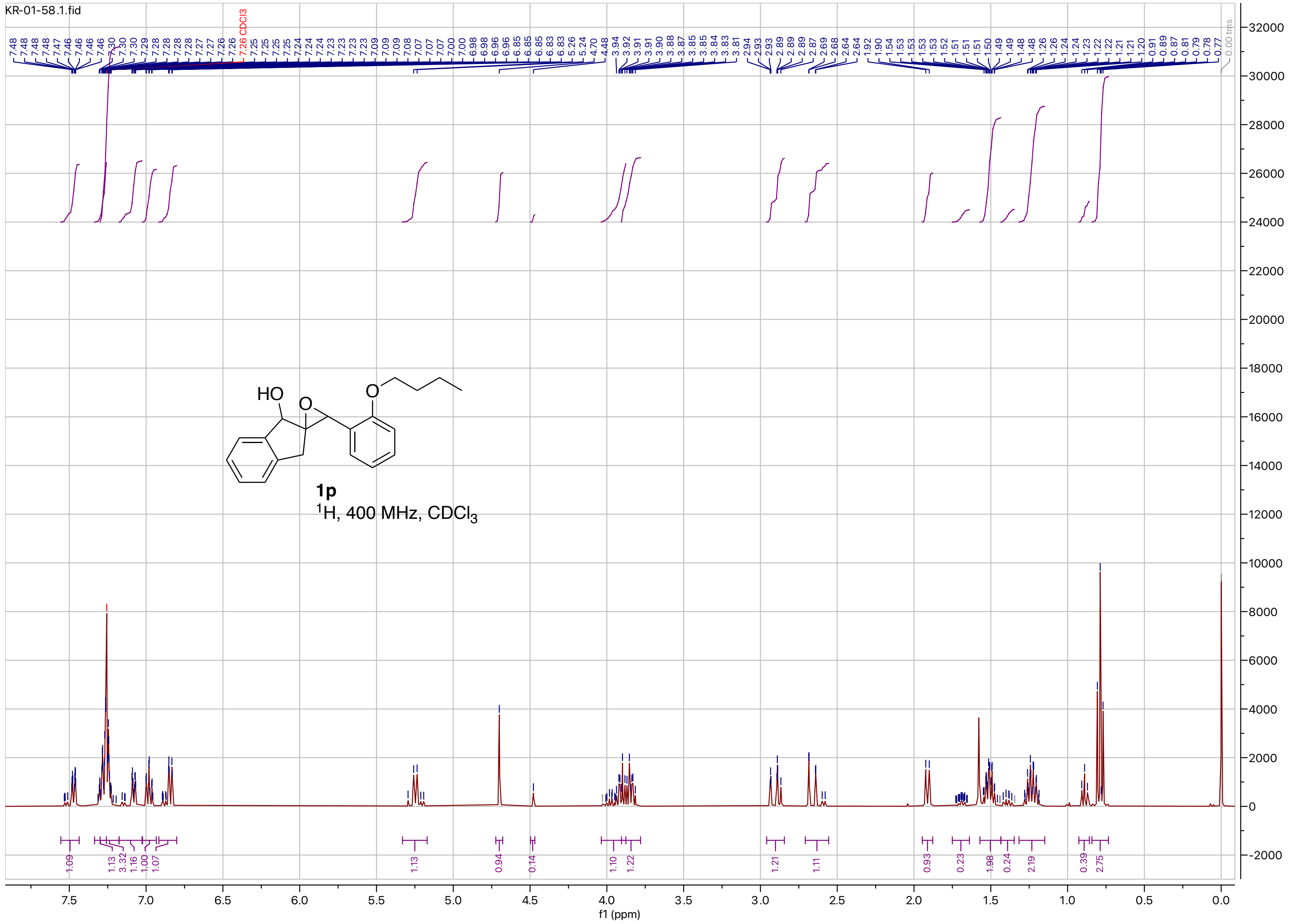

1p

^{1}H, 400 MHz, $CDCl_3$

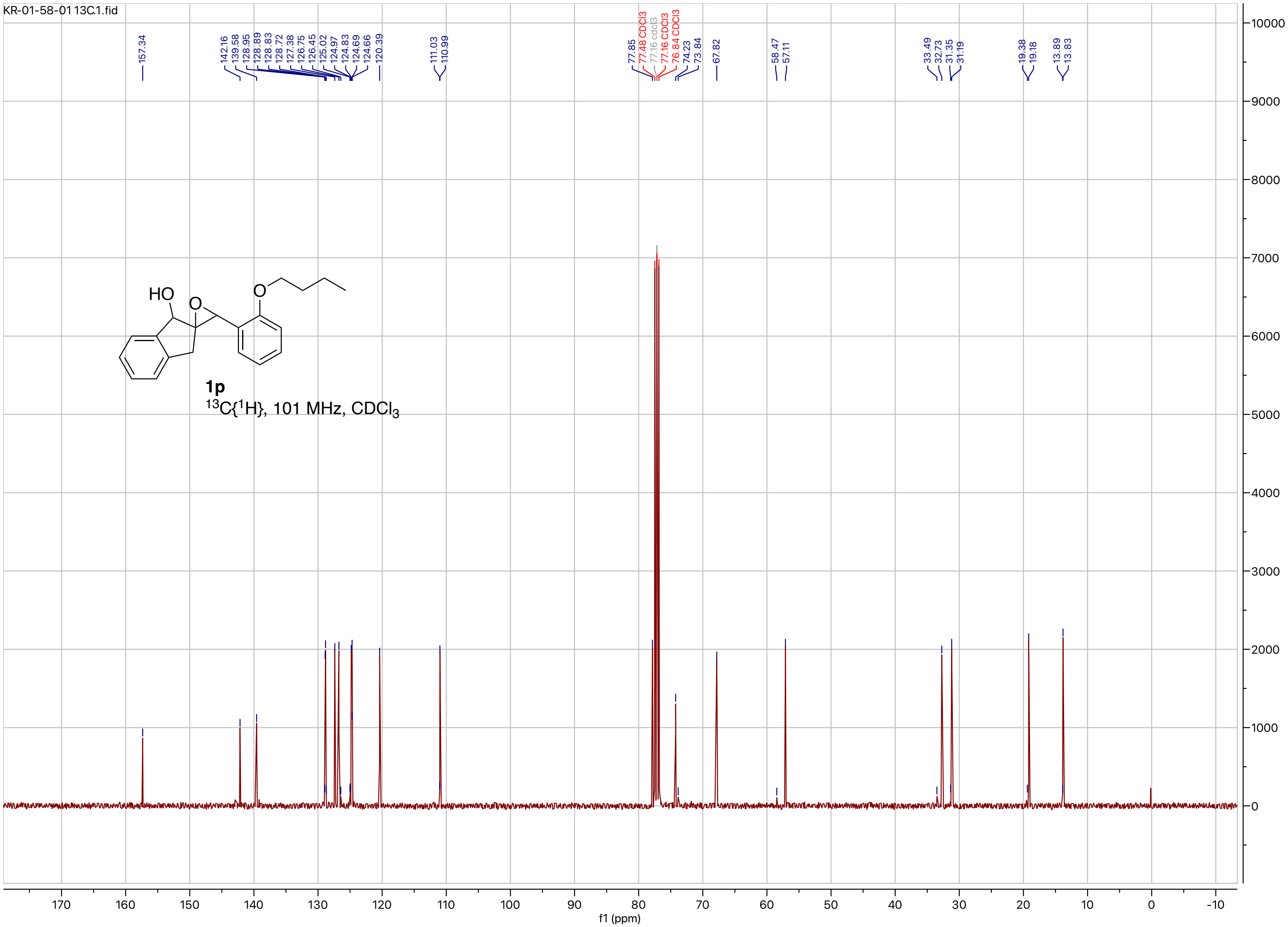
KR-01-58-01 13C.1.fid
157.34
142.16
139.58
128.95
128.89
128.83
128.72
127.38
126.75
126.45
125.02
124.97
124.83
124.69
124.66
120.39
111.03
110.99
77.85
77.48 CDCl3
77.16 cdcl3
77.16 CDCl3
76.84 CDCl3
74.23
73.84
67.82
58.47
57.11
33.49
32.73
31.35
31.19
19.38
19.18
13.89
13.83
HO
O
O
1p
13C{1H}, 101 MHz, CDCl3
f1 (ppm)

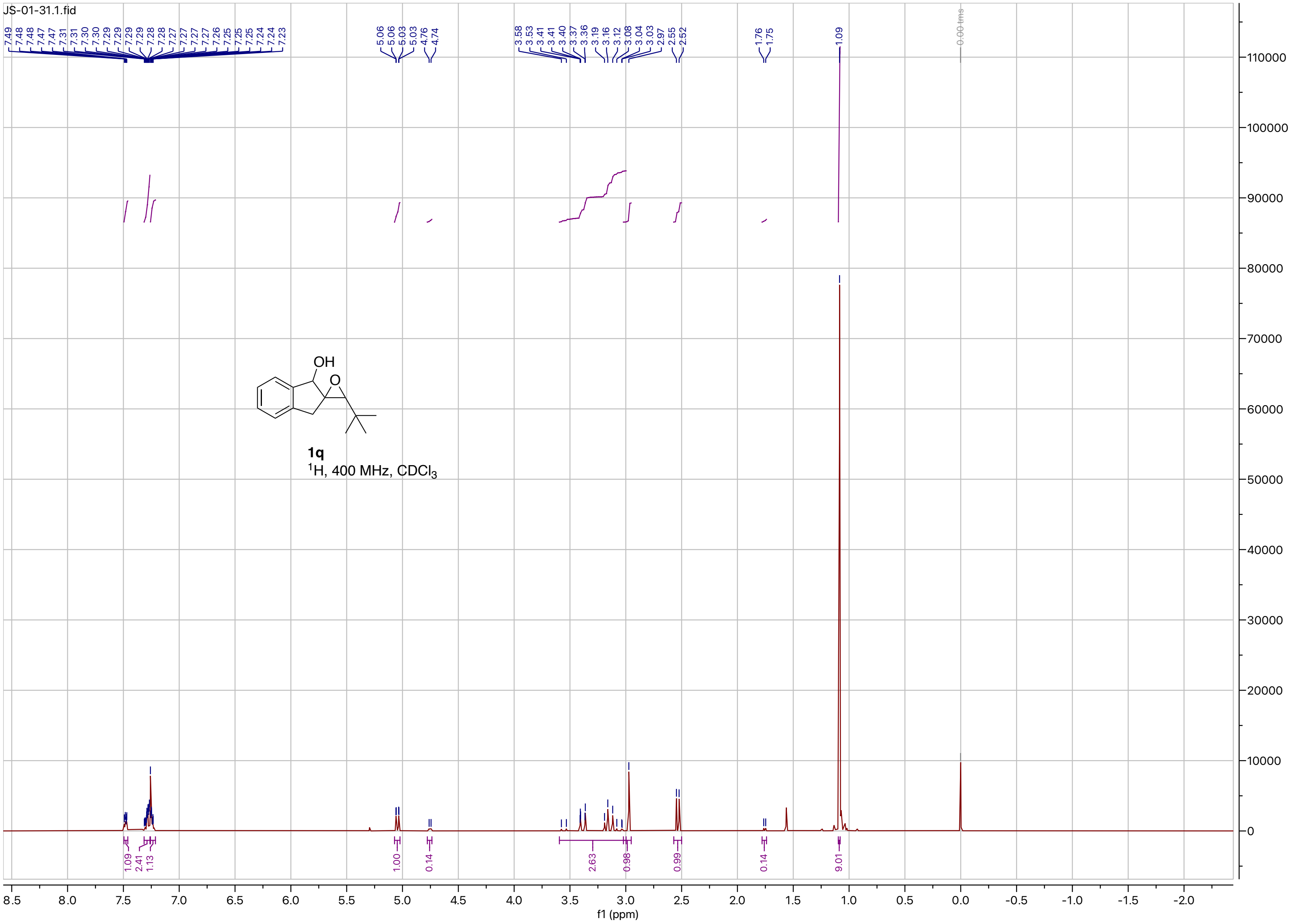
JS-01-31.1.fid
1q
^{1}H, 400 MHz, CDCl$_3$
f1 (ppm)

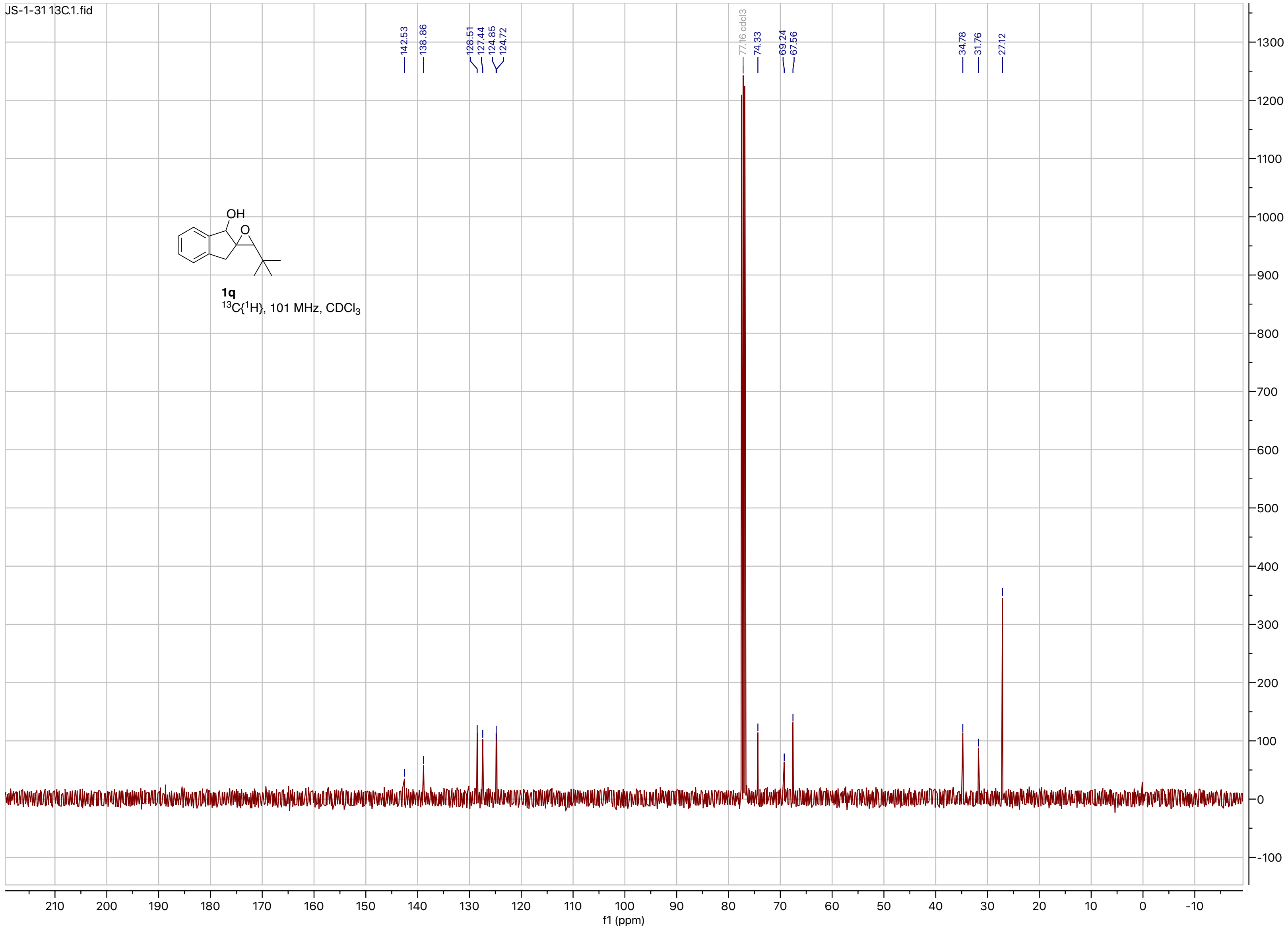

JS-1-31 13C.1.fid
142.53
138.86
128.51
127.44
124.85
124.72
77.16 cdcl3
74.33
69.24
67.56
34.78
31.76
27.12
OH
O
1q
13C{1H}, 101 MHz, CDCl3
f1 (ppm)

B-NAPHTHOLS

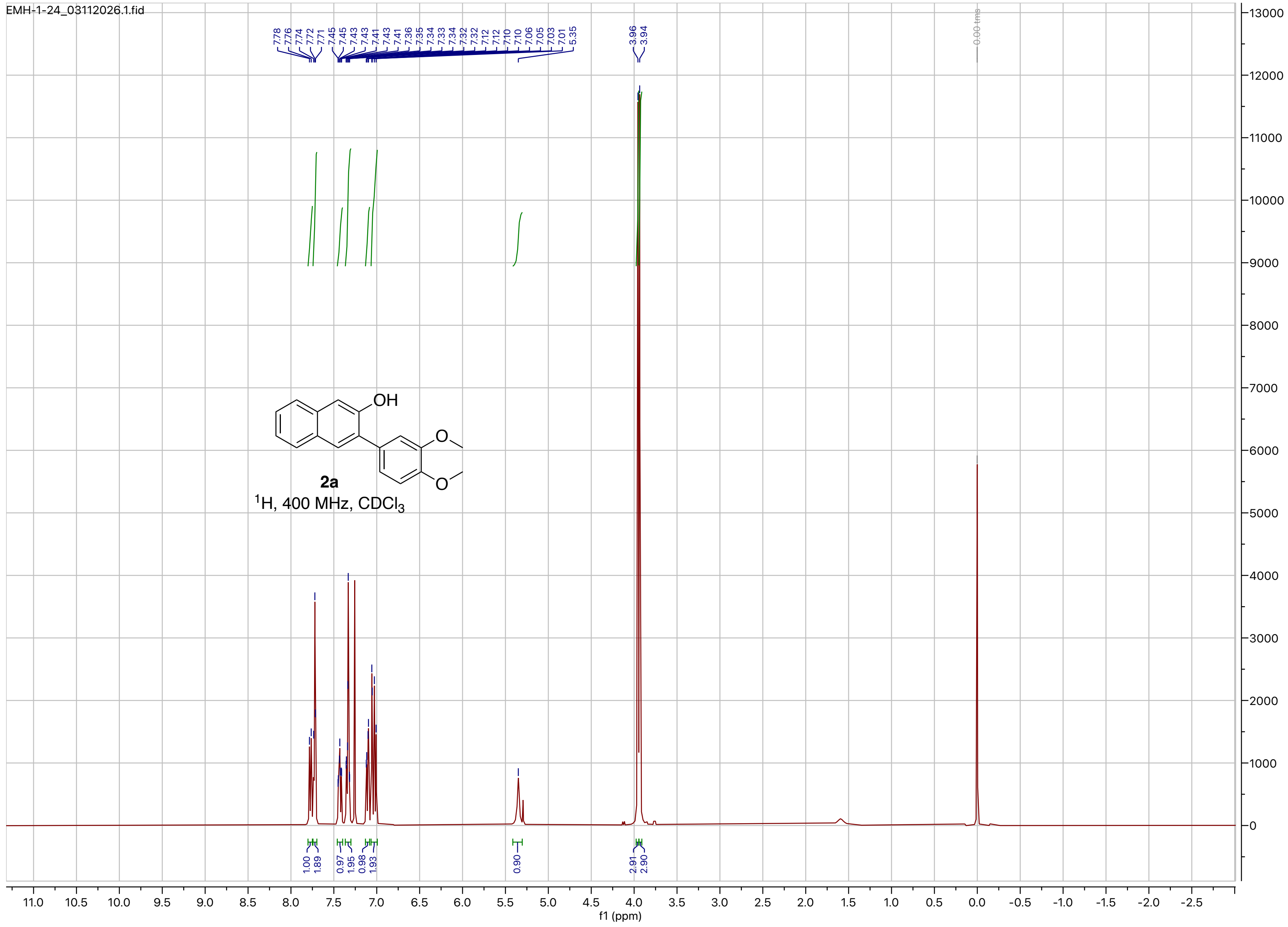

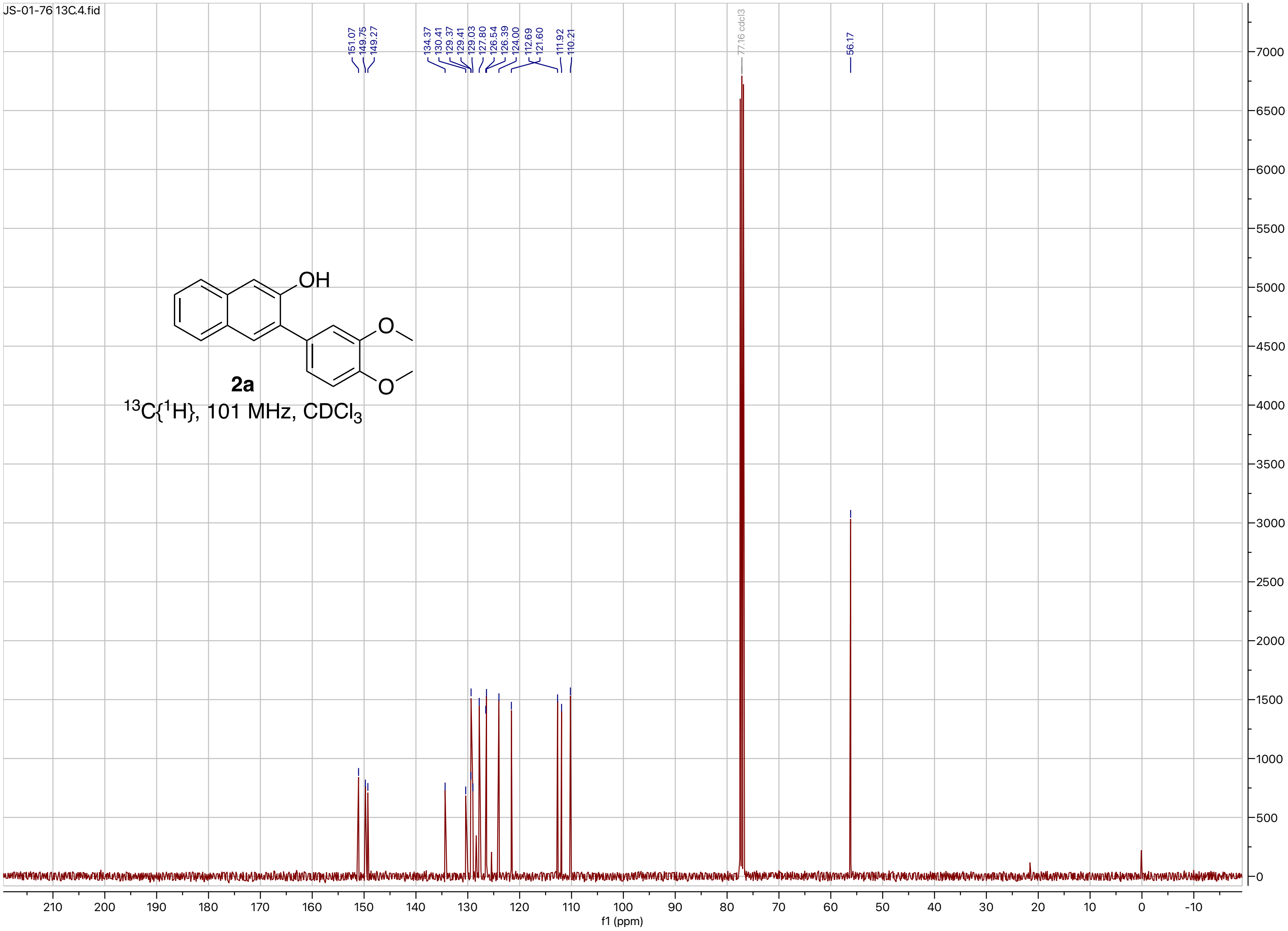
JS-01-76 13C.4.fid
151.07
149.75
149.27
134.37
130.41
129.37
129.41
129.03
127.80
126.54
126.39
124.00
112.69
121.60
111.92
110.21
77.16 cdcl3
56.17
OH
O
O
2a
13C{1H}, 101 MHz, CDCl3
f1 (ppm)

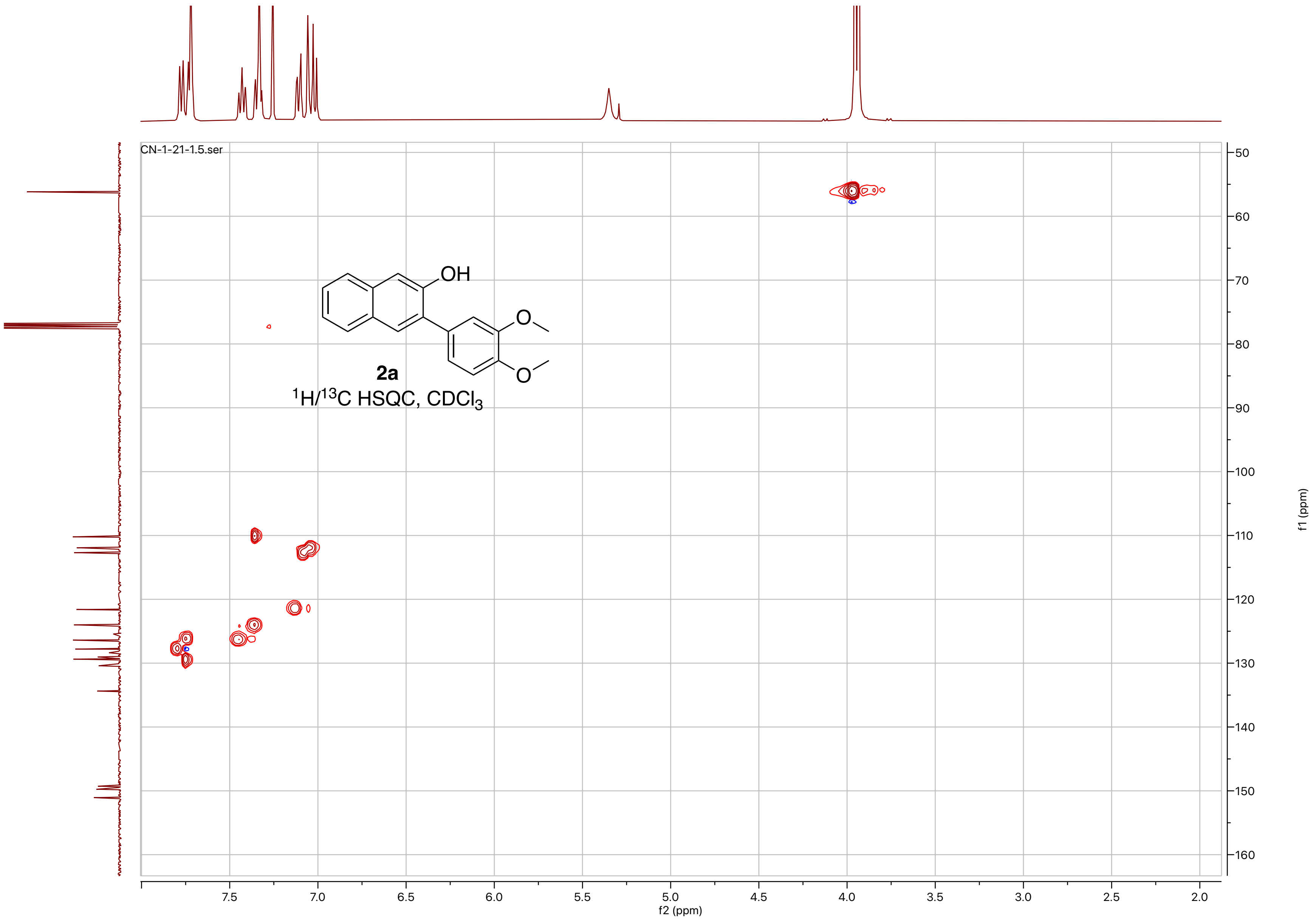

CN-1-21-1.5.ser
OH
O
O
2a
1H/13C HSQC, CDCl3
f1 (ppm)
f2 (ppm)

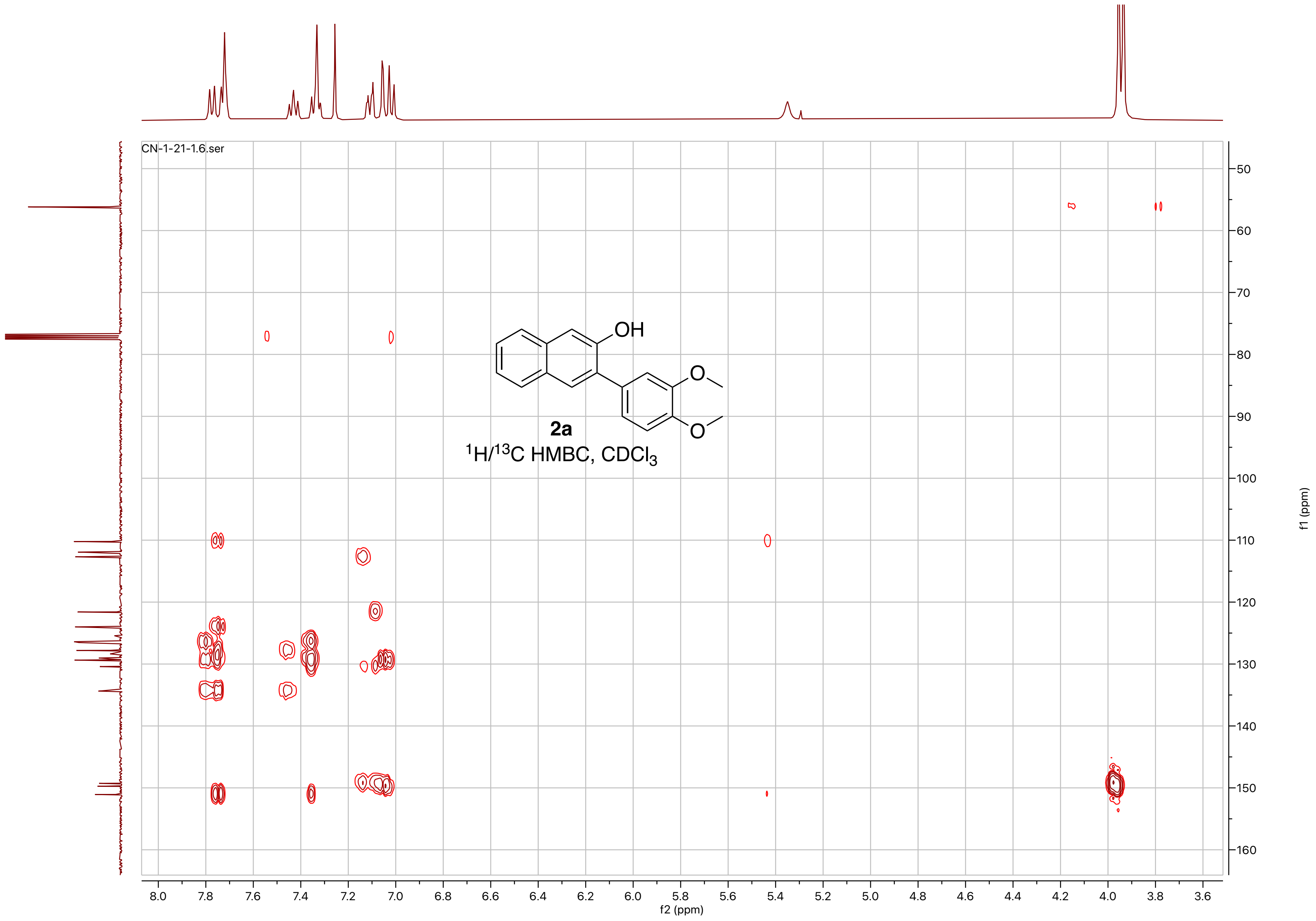
CN-1-21-1.6.ser
OH
O
O
2a
1H/13C HMBC, CDCl3
f2 (ppm)
f1 (ppm)

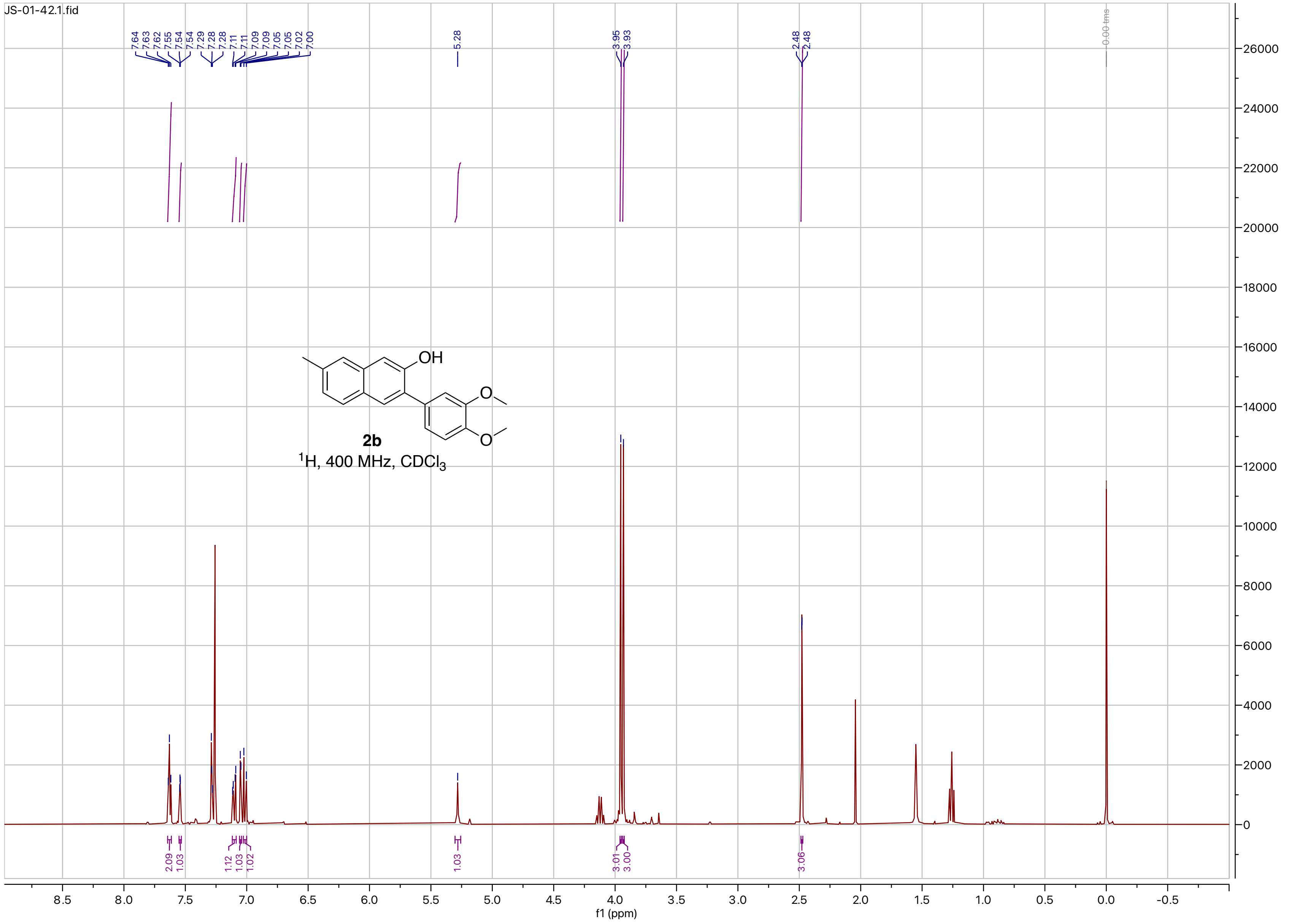
JS-01-42.1.fid
7.64
7.63
7.62
7.55
7.54
7.54
7.29
7.28
7.28
7.11
7.11
7.09
7.09
7.05
7.05
7.02
7.00
5.28
3.95
3.93
2.48
2.48
0.00 tms
OH
O
O
2b
1H, 400 MHz, CDCl3
2.09
1.03
1.12
1.03
1.02
1.03
3.01
3.00
3.06
f1 (ppm)

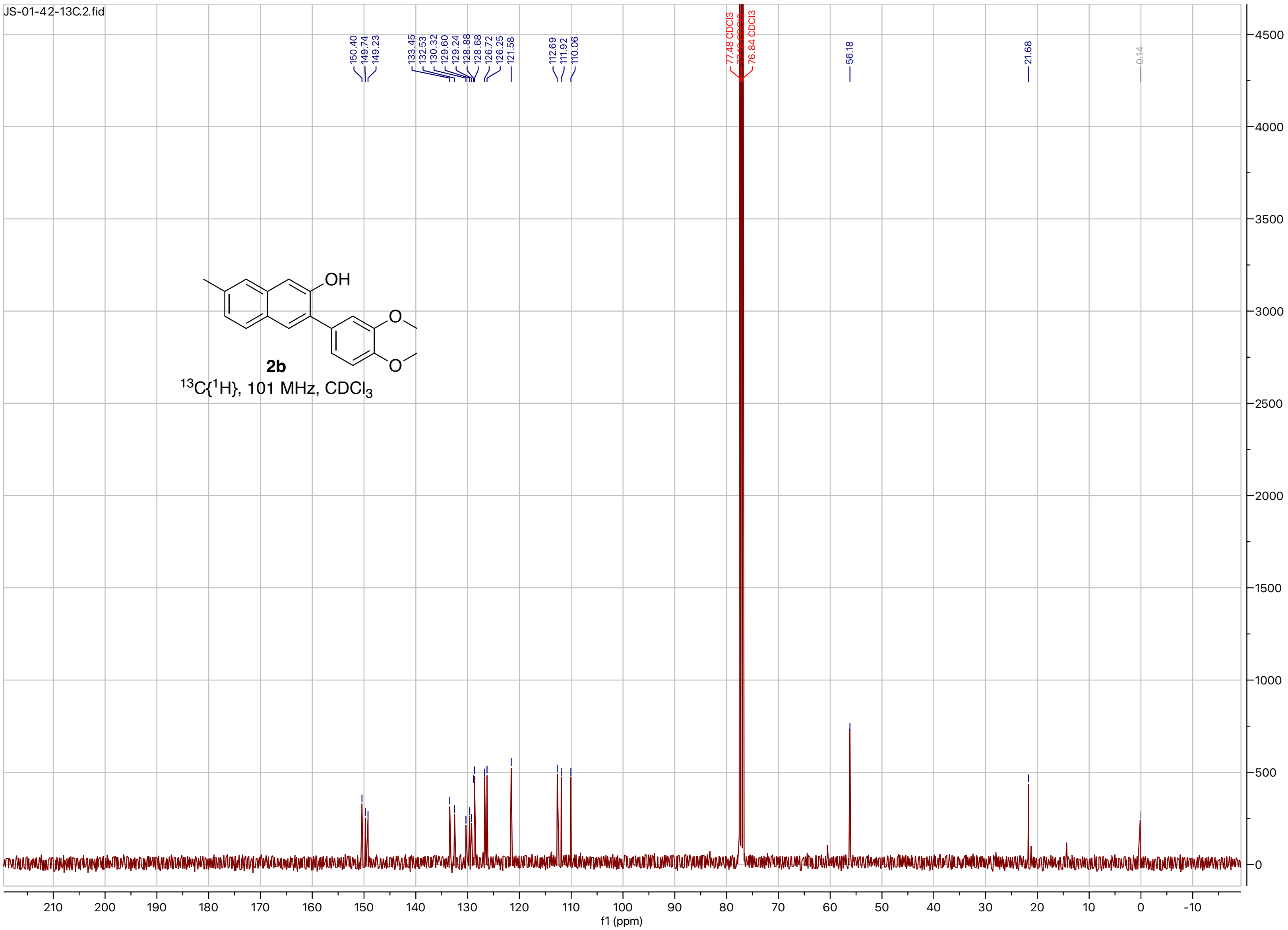
JS-01-42-13C.2.fid
150.40
149.74
149.23
133.45
132.53
130.32
129.60
129.24
128.88
128.68
126.72
126.25
121.58
112.69
111.92
110.06
77.48 CDCl3
76.84 CDCl3
56.18
21.68
OH
O
O
2b
$^{13}C\{^{1}H\}$, 101 MHz, $CDCl_3$
f1 (ppm)

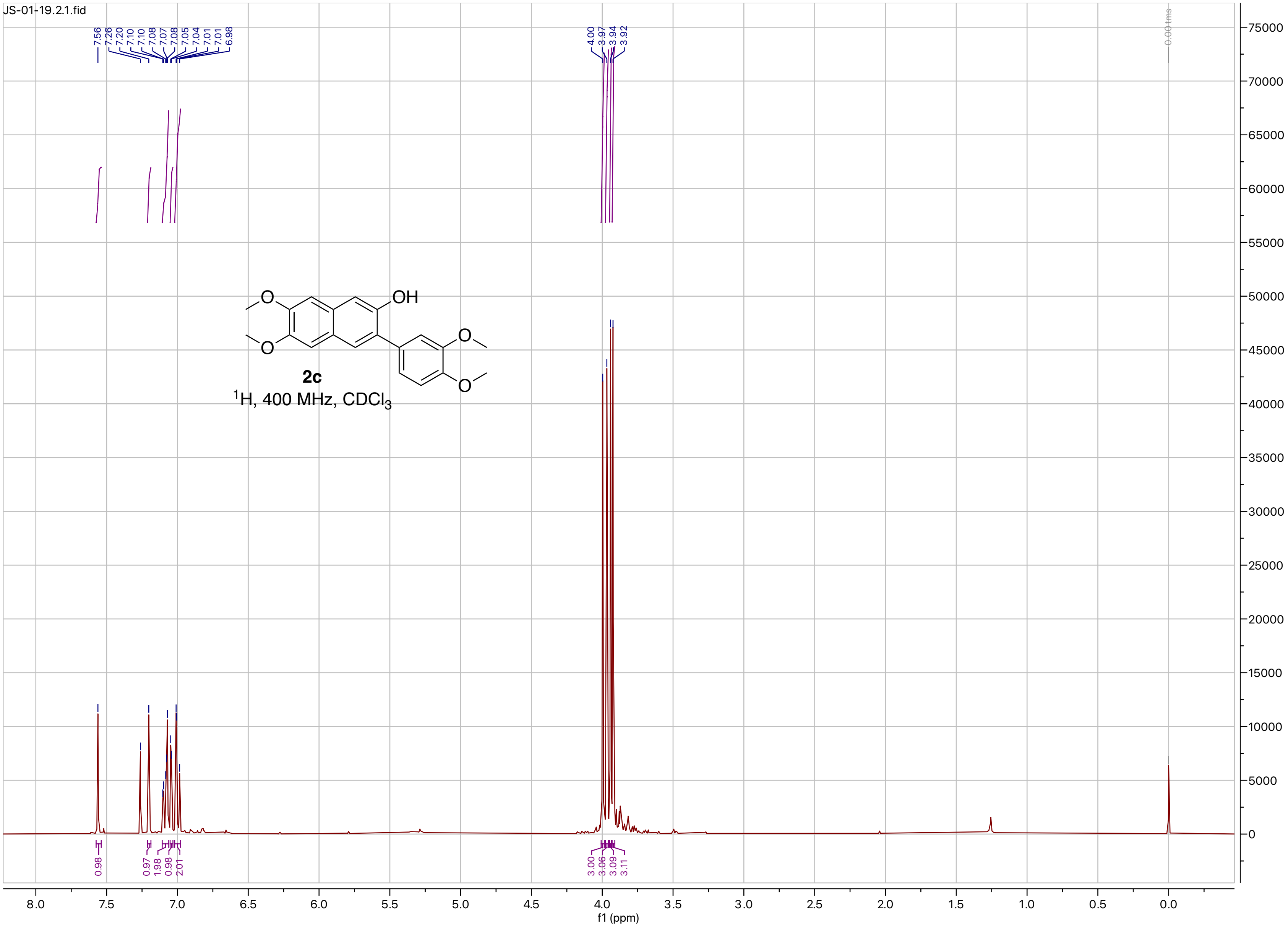
JS-01-19.2.1.fid
7.56
7.26
7.20
7.10
7.10
7.08
7.07
7.08
7.05
7.04
7.01
7.01
6.98
4.00
3.97
3.94
3.92
0.00 tms
OH
O
O
O
O
2c
1H, 400 MHz, CDCl3
0.98
0.97
1.98
0.98
2.01
3.00
3.06
3.09
3.11
f1 (ppm)

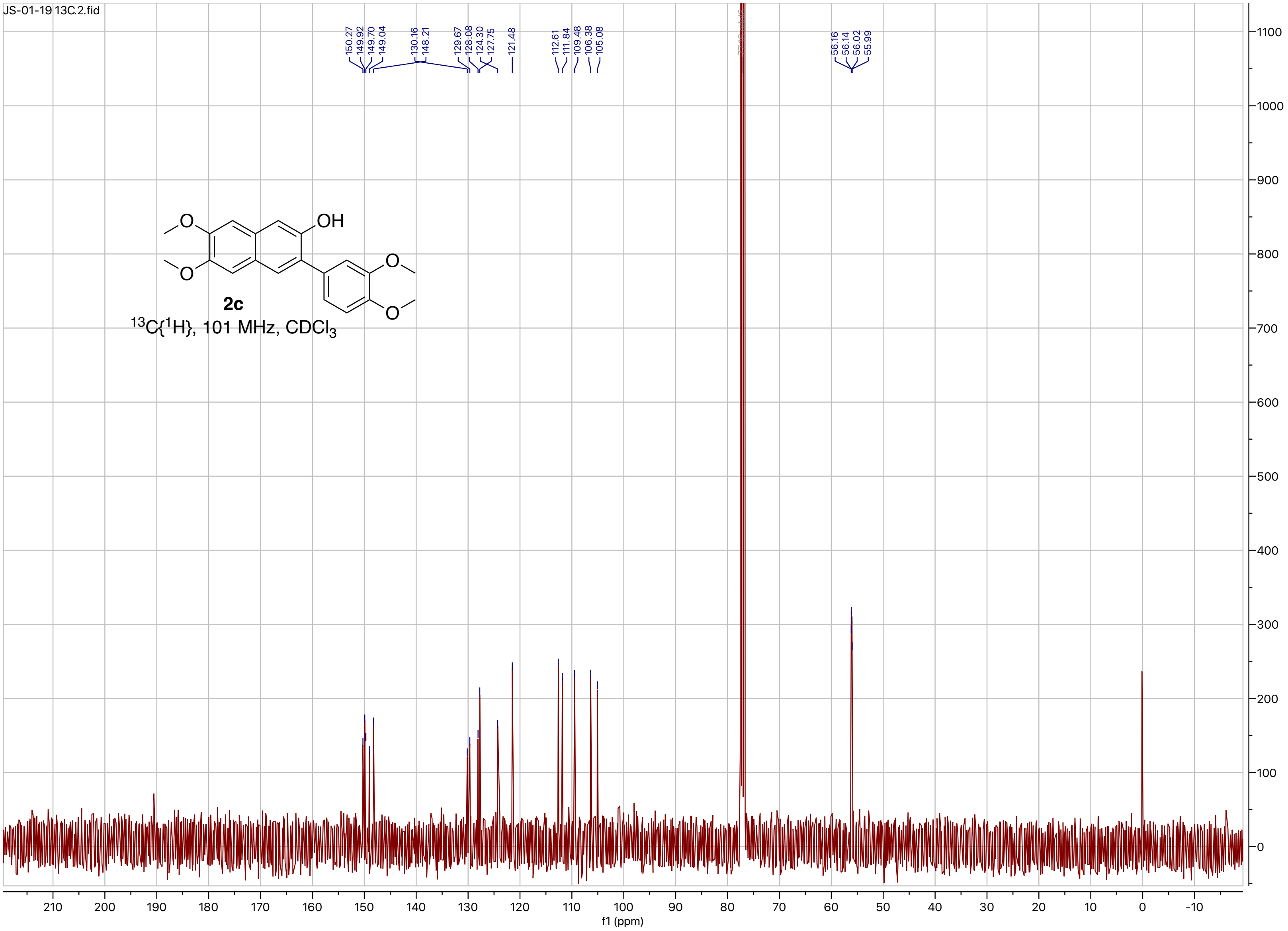
JS-01-19 13C.2.fid
150.27
149.92
149.70
149.04
130.16
148.21
129.67
128.08
124.30
127.75
121.48
112.61
111.84
109.48
106.38
105.08
56.16
56.14
56.02
55.99
OH
O
O
O
O
2c
13C{1H}, 101 MHz, CDCl3
f1 (ppm)

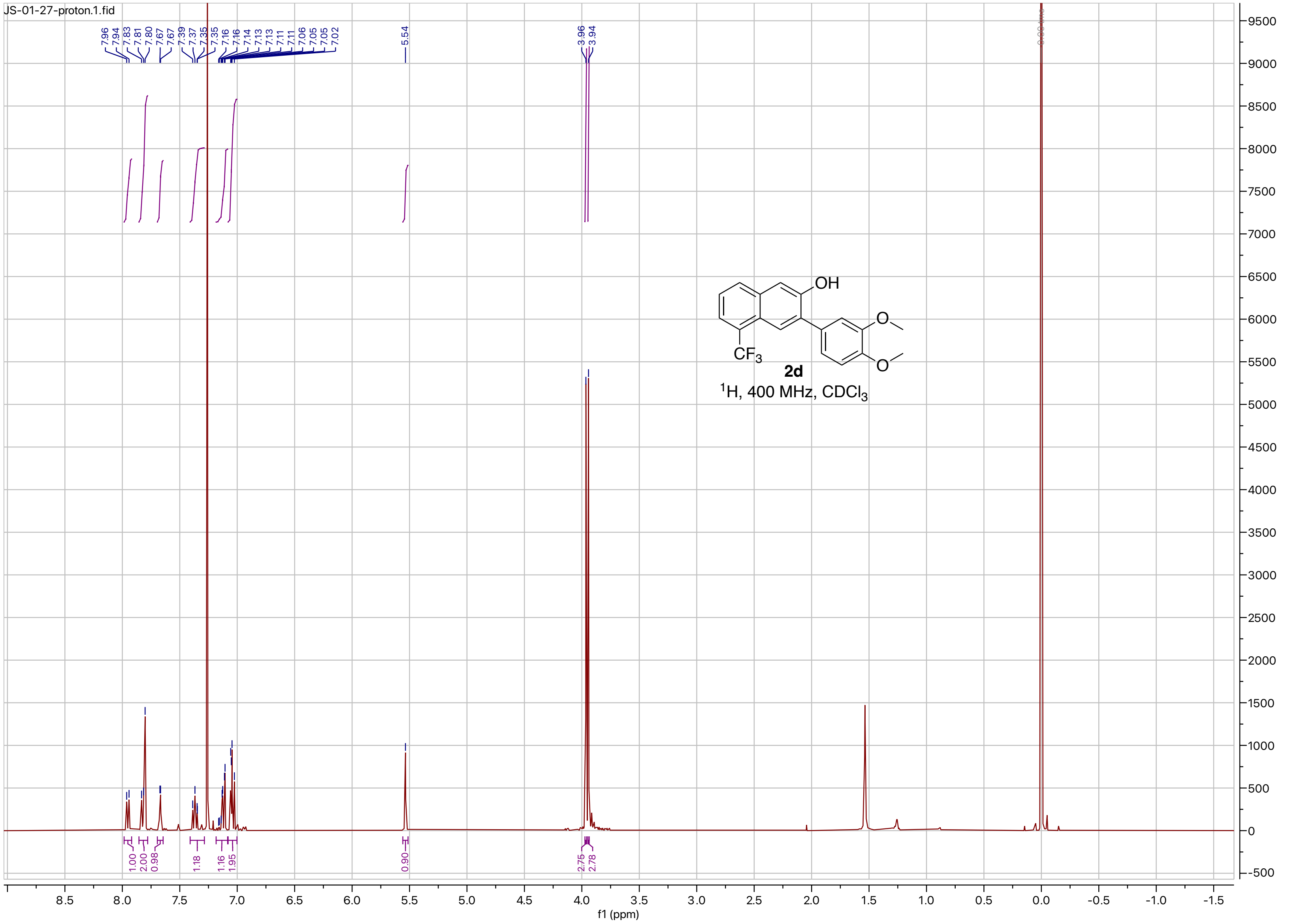
JS-01-27-proton.1.fid
7.96 7.94 7.83 7.81 7.80 7.67 7.67 7.39 7.37 7.35 7.16 7.14 7.13 7.13 7.11 7.11 7.06 7.05 7.05 7.02
5.54
3.96 3.94
OH
O
O
CF3
2d
1H, 400 MHz, CDCl3
1.00 2.00 0.98 1.18 1.16 1.95 0.90 2.75 2.78
f1 (ppm)

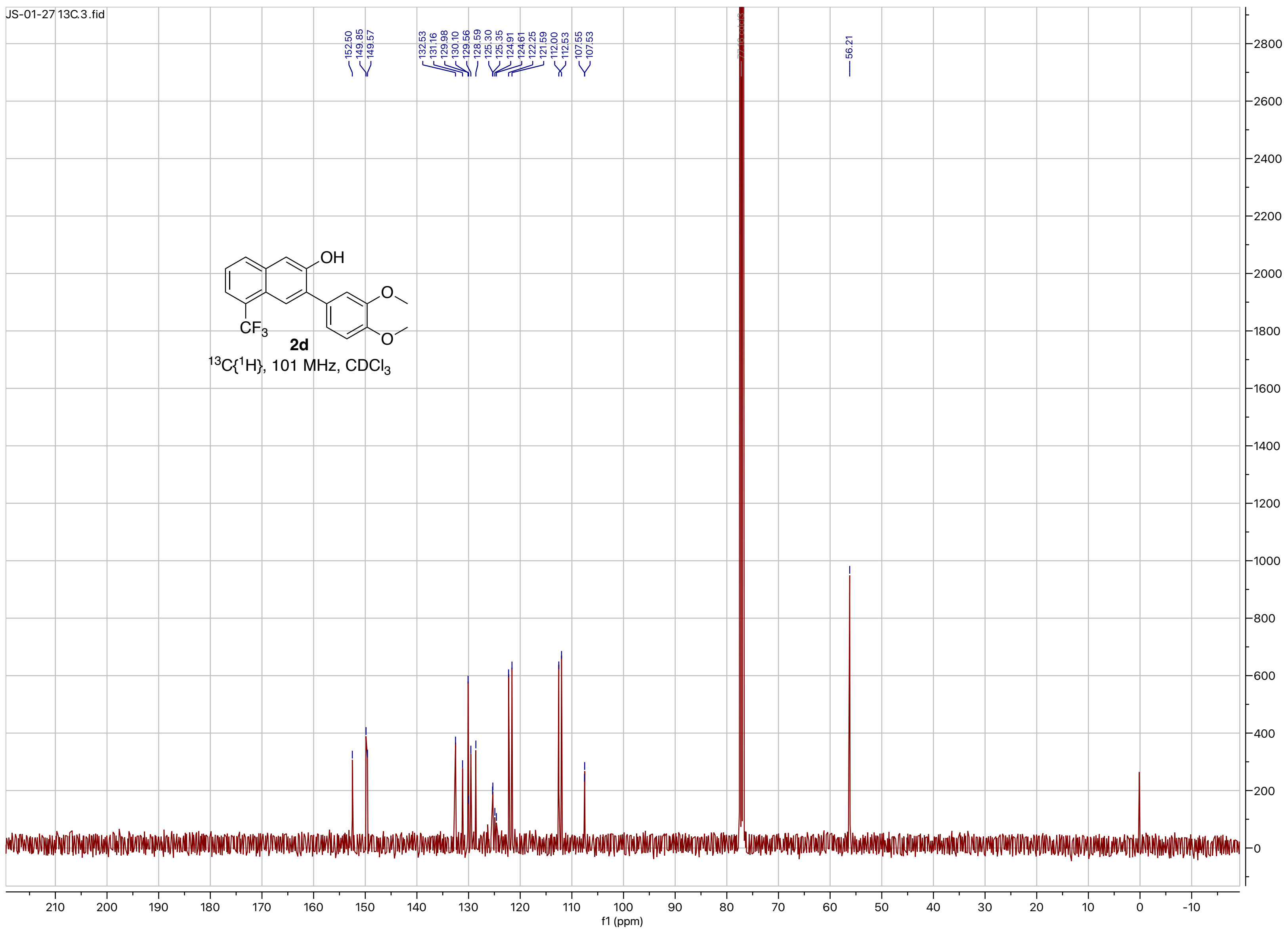
JS-01-27 13C.3.fid
152.50
149.85
149.57
132.53
131.16
129.98
130.10
129.56
128.59
125.30
125.35
124.91
124.61
122.25
121.59
112.00
112.53
107.55
107.53
56.21
OH
O
O
CF3
2d
13C{1H}, 101 MHz, CDCl3
f1 (ppm)

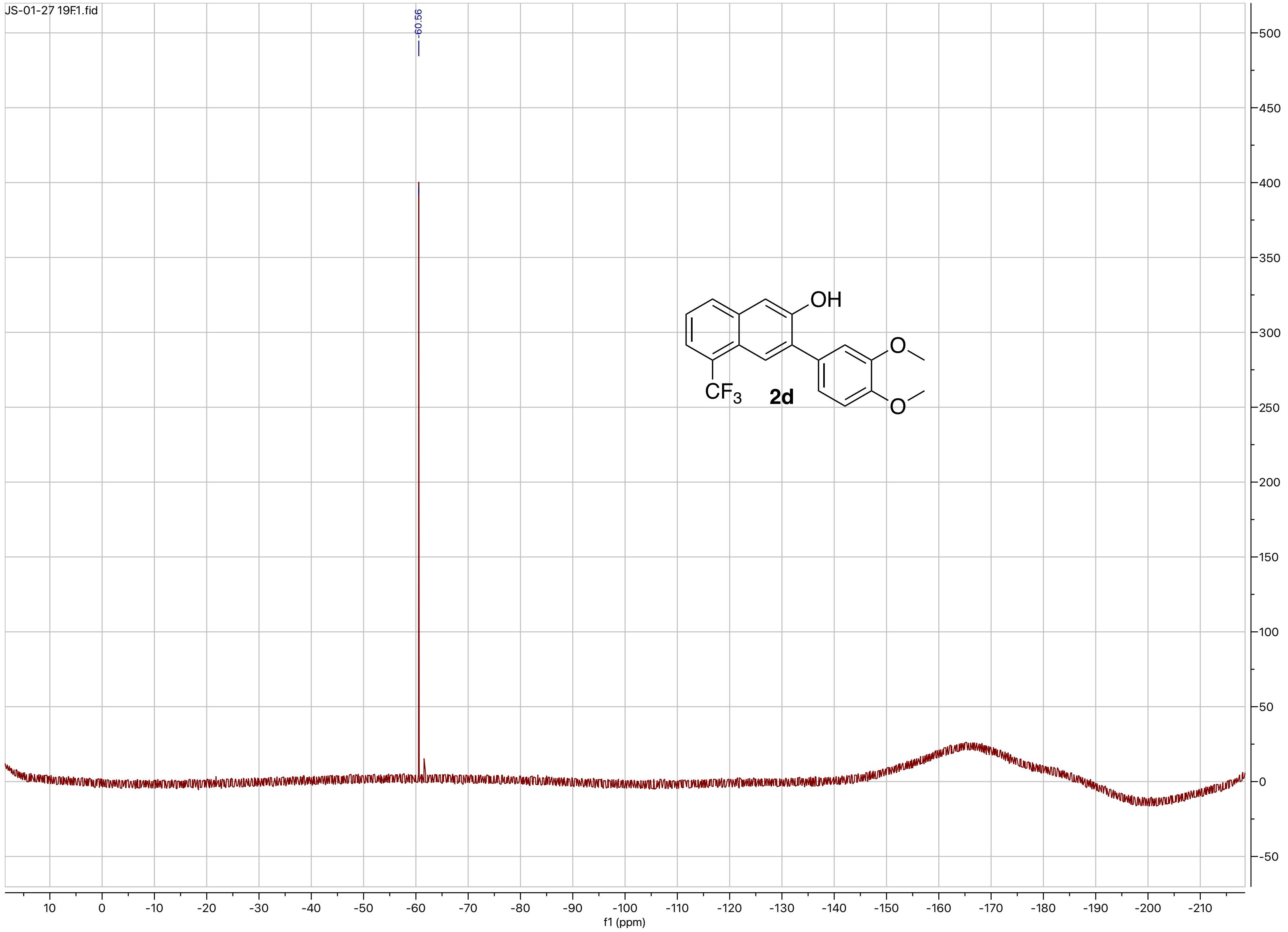
JS-01-27 19F1.fid
-60.56
OH
O
O
CF3
2d
f1 (ppm)

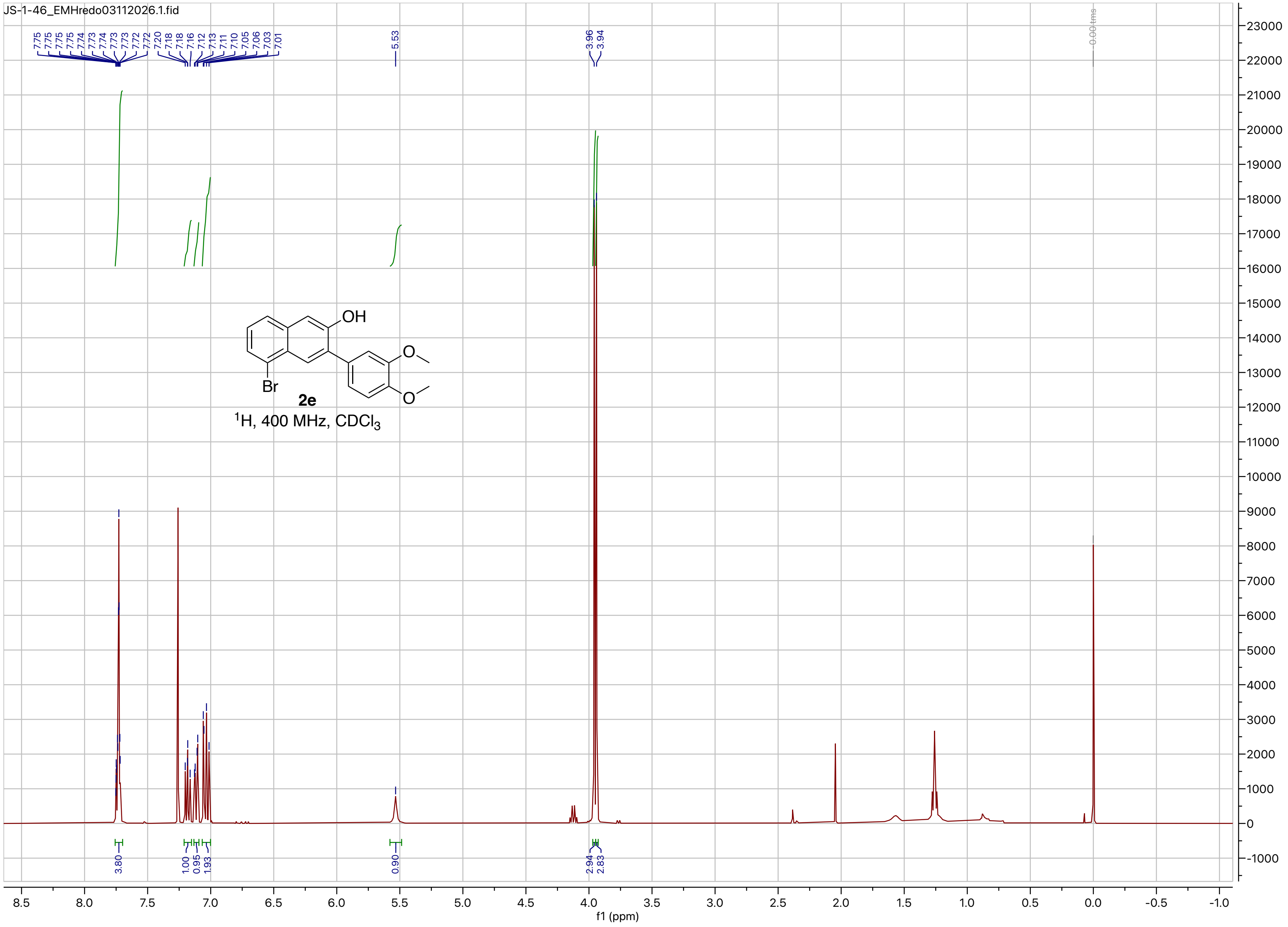
JS-1-46_EMHredo03112026.1.fid
OH
O
O
Br
2e
^{1}H, 400 MHz, $CDCl_3$
f1 (ppm)

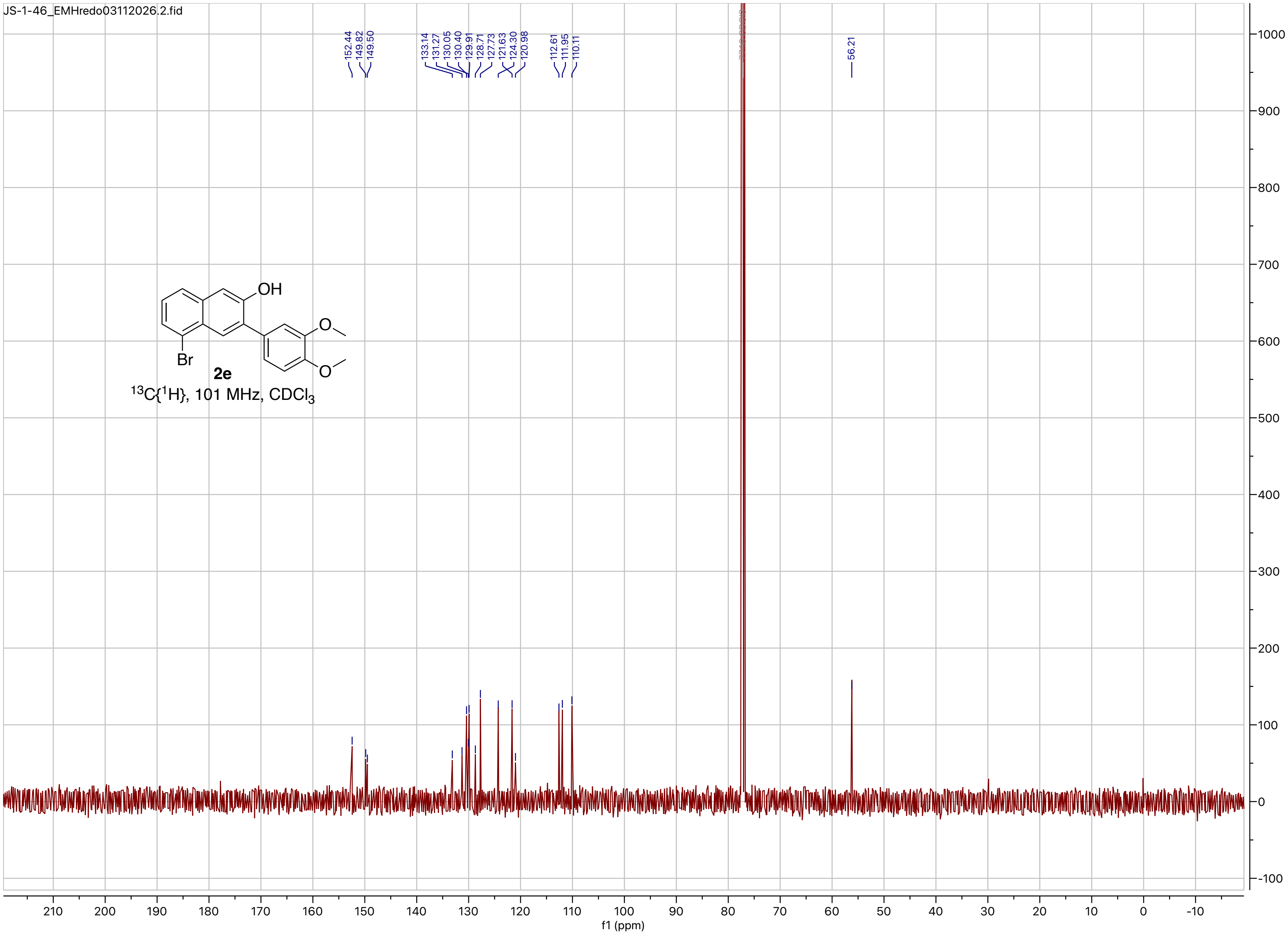

JS-1-46_EMHredo03112026.2.fid
152.44
149.82
149.50
133.14
131.27
130.05
130.40
129.91
128.71
127.73
121.63
124.30
120.98
112.61
111.95
110.11
56.21
OH
O
O
Br
2e
13C{1H}, 101 MHz, CDCl3
f1 (ppm)

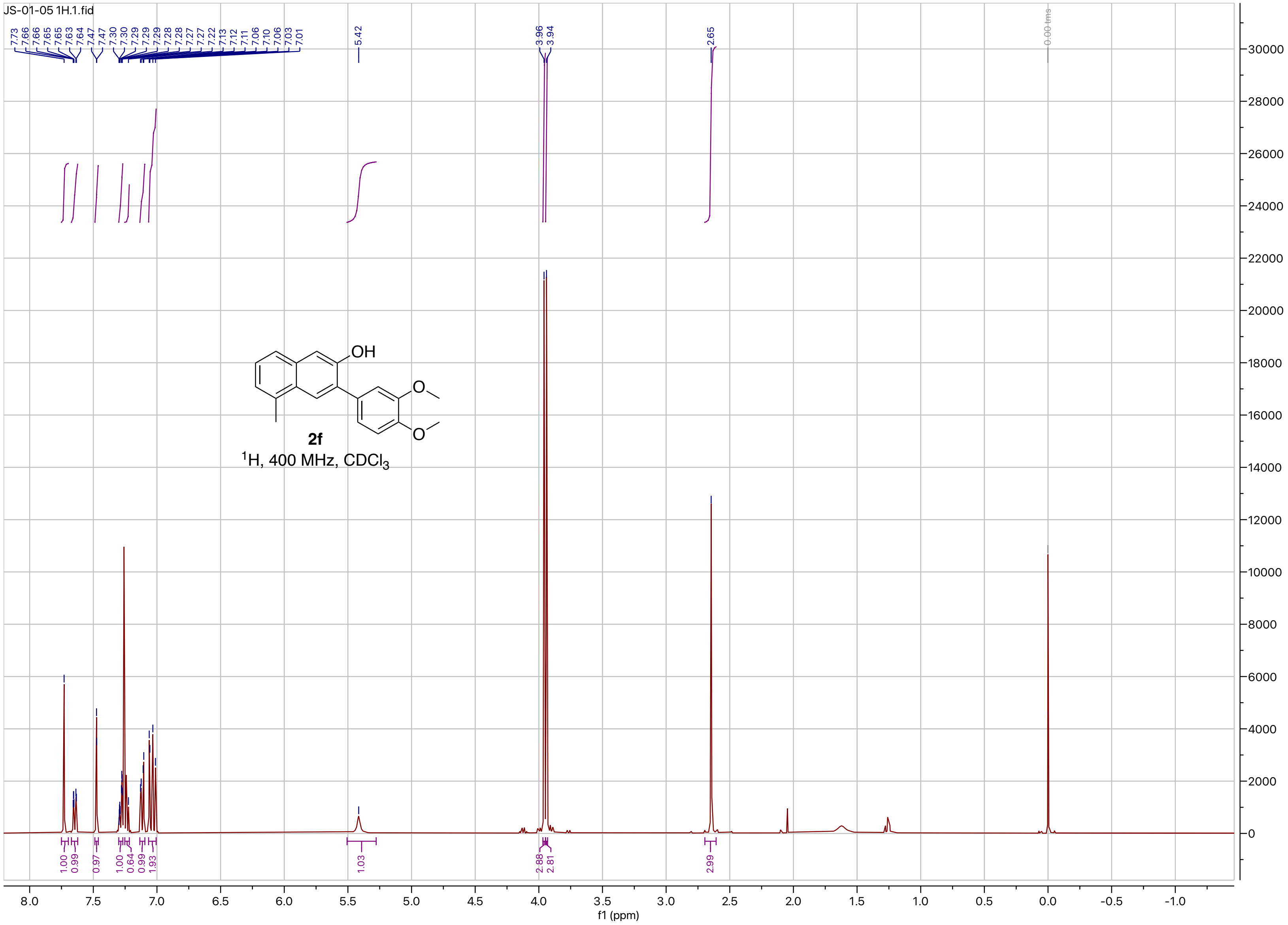
JS-01-05 1H.1.fid
7.73 7.66 7.66 7.65 7.65 7.63 7.64 7.47 7.47 7.30 7.30 7.29 7.29 7.29 7.28 7.28 7.27 7.27 7.22 7.13 7.12 7.11 7.06 7.10 7.06 7.03 7.01
5.42
3.96 3.94
2.65
0.00 tms
OH
O
O
2f
^{1}H, 400 MHz, $CDCl_3$
1.00 0.99 0.97 1.00 0.64 0.99 1.93
1.03
2.88 2.81
2.99
f1 (ppm)

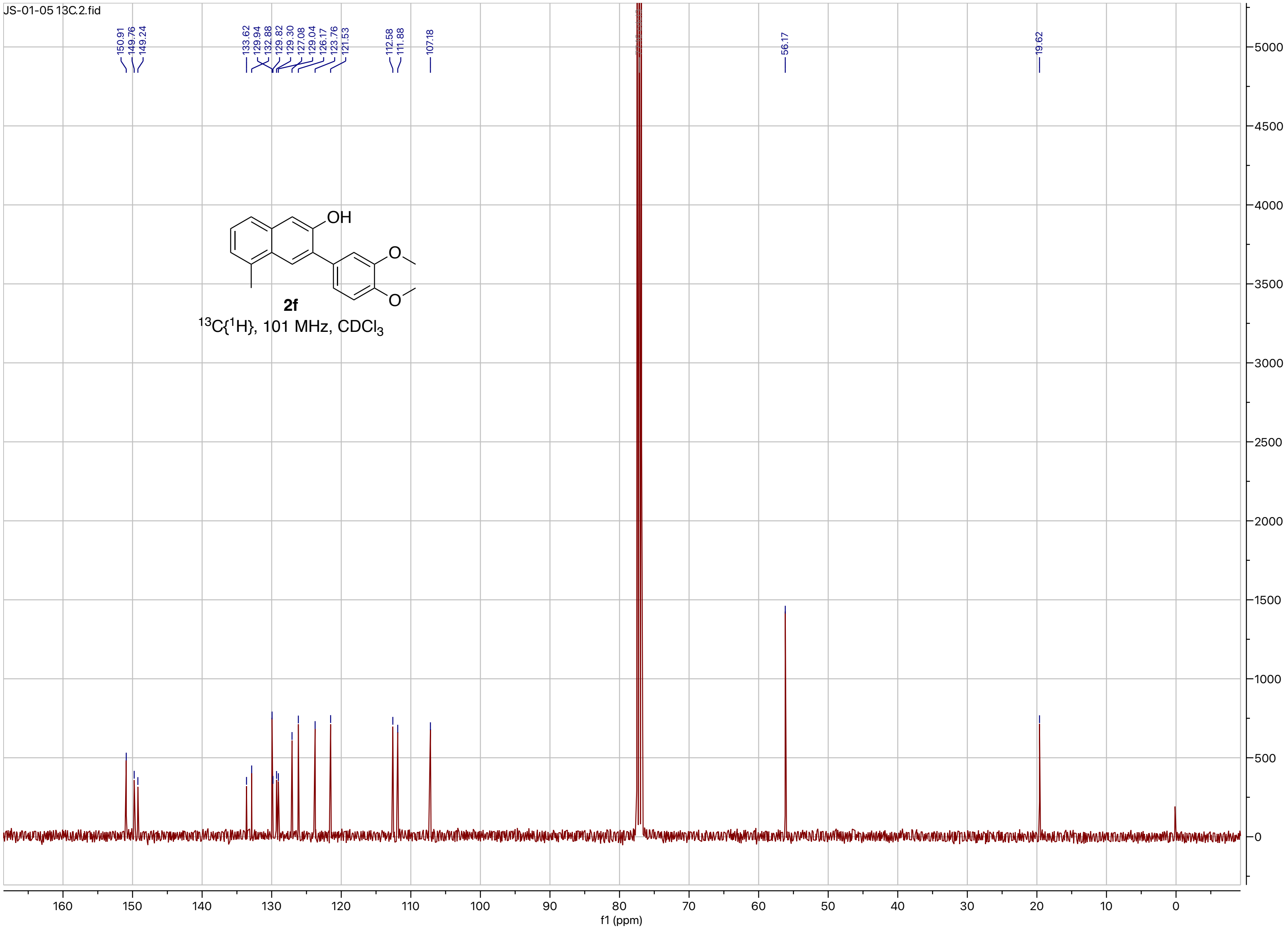
JS-01-05 13C.2.fid
150.91
149.76
149.24
133.62
129.94
132.88
129.82
129.30
127.08
129.04
126.17
123.76
121.53
112.58
111.88
107.18
56.17
19.62
OH
O
O
2f
13C{1H}, 101 MHz, CDCl3
f1 (ppm)

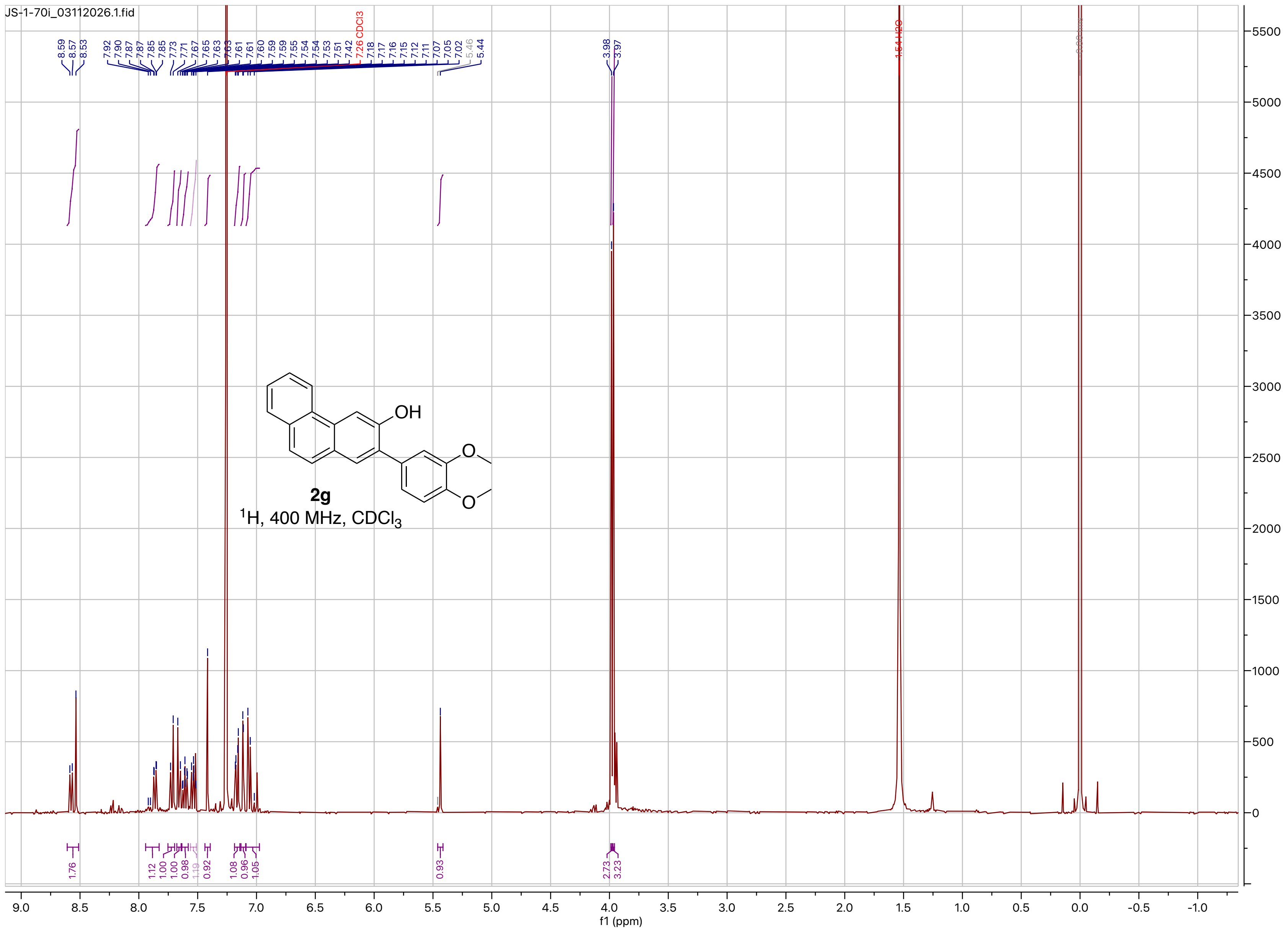
JS-1-70i_03112026.1.fid
8.59 8.57 8.53 7.92 7.90 7.87 7.85 7.85 7.73 7.71 7.67 7.65 7.63 7.63 7.61 7.61 7.60 7.59 7.59 7.55 7.54 7.54 7.53 7.51 7.42 7.26 CDCl3 7.18 7.17 7.16 7.15 7.12 7.11 7.07 7.05 7.02 5.46 5.44
3.98 3.97
1.54 H2O
OH
O
O
2g
1H, 400 MHz, CDCl3
1.76 1.12 1.00 1.00 0.98 1.19 0.92 1.08 0.96 1.05 0.93 2.73 3.23
f1 (ppm)

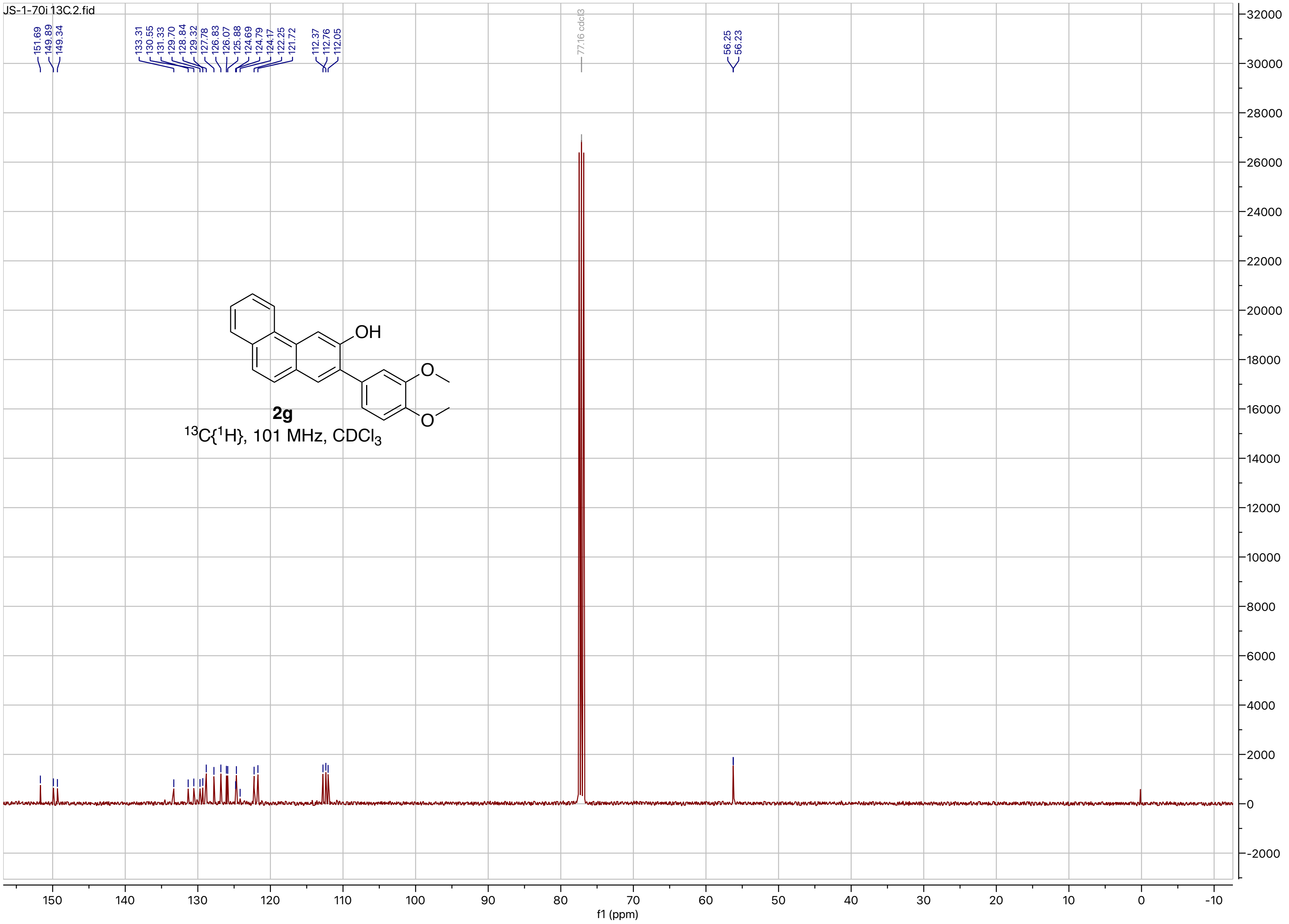

JS-1-70i 13C.2.fid
151.69
149.89
149.34
133.31
130.55
131.33
129.70
128.84
129.32
127.78
126.83
126.07
125.88
124.69
124.79
124.17
122.25
121.72
112.37
112.76
112.05
77.16 cdcl3
56.25
56.23
OH
O
O
2g
13C{1H}, 101 MHz, CDCl3
f1 (ppm)

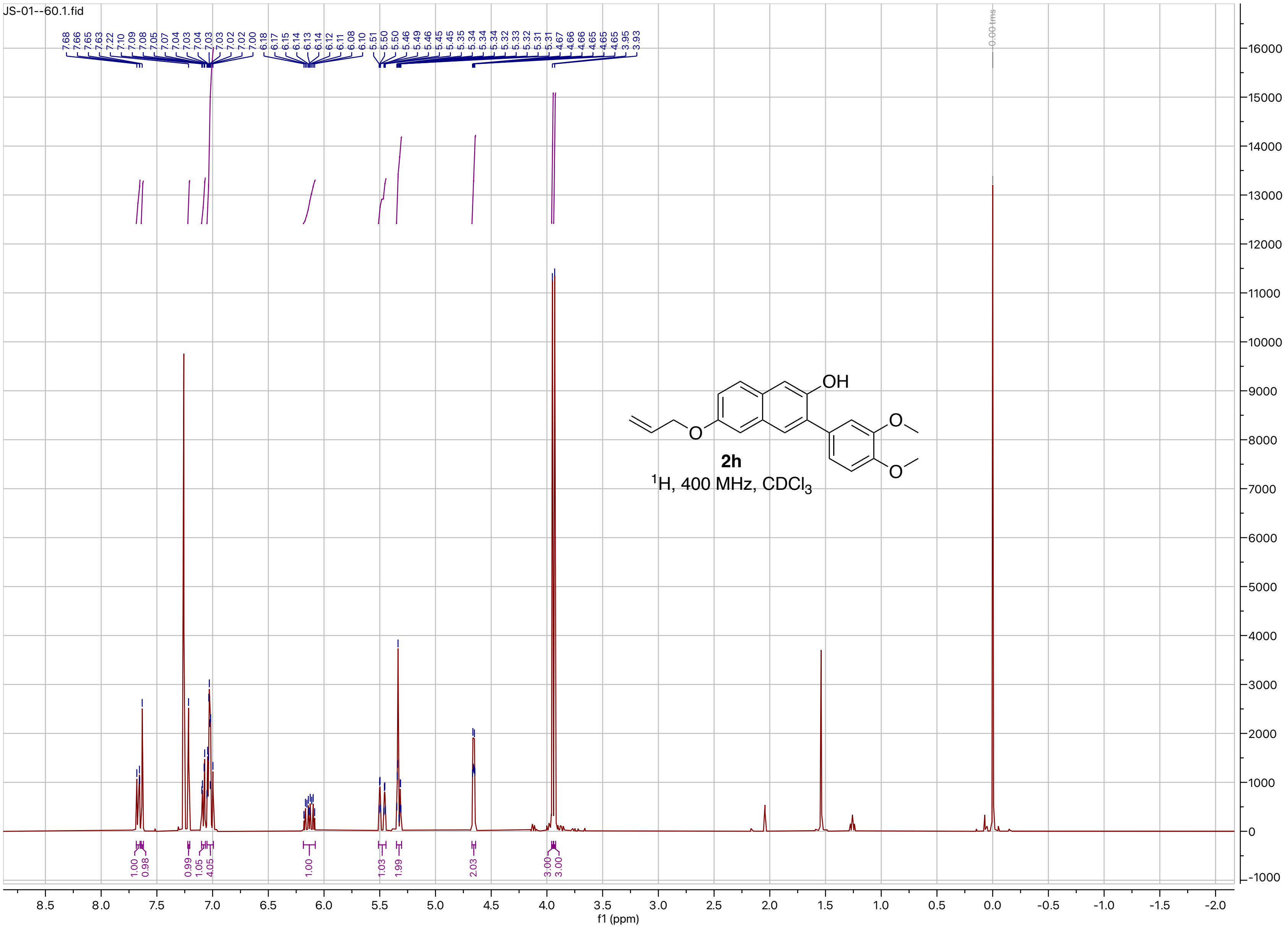
JS-01--60.1.fid
2h
1H, 400 MHz, CDCl3
OH
O
f1 (ppm)

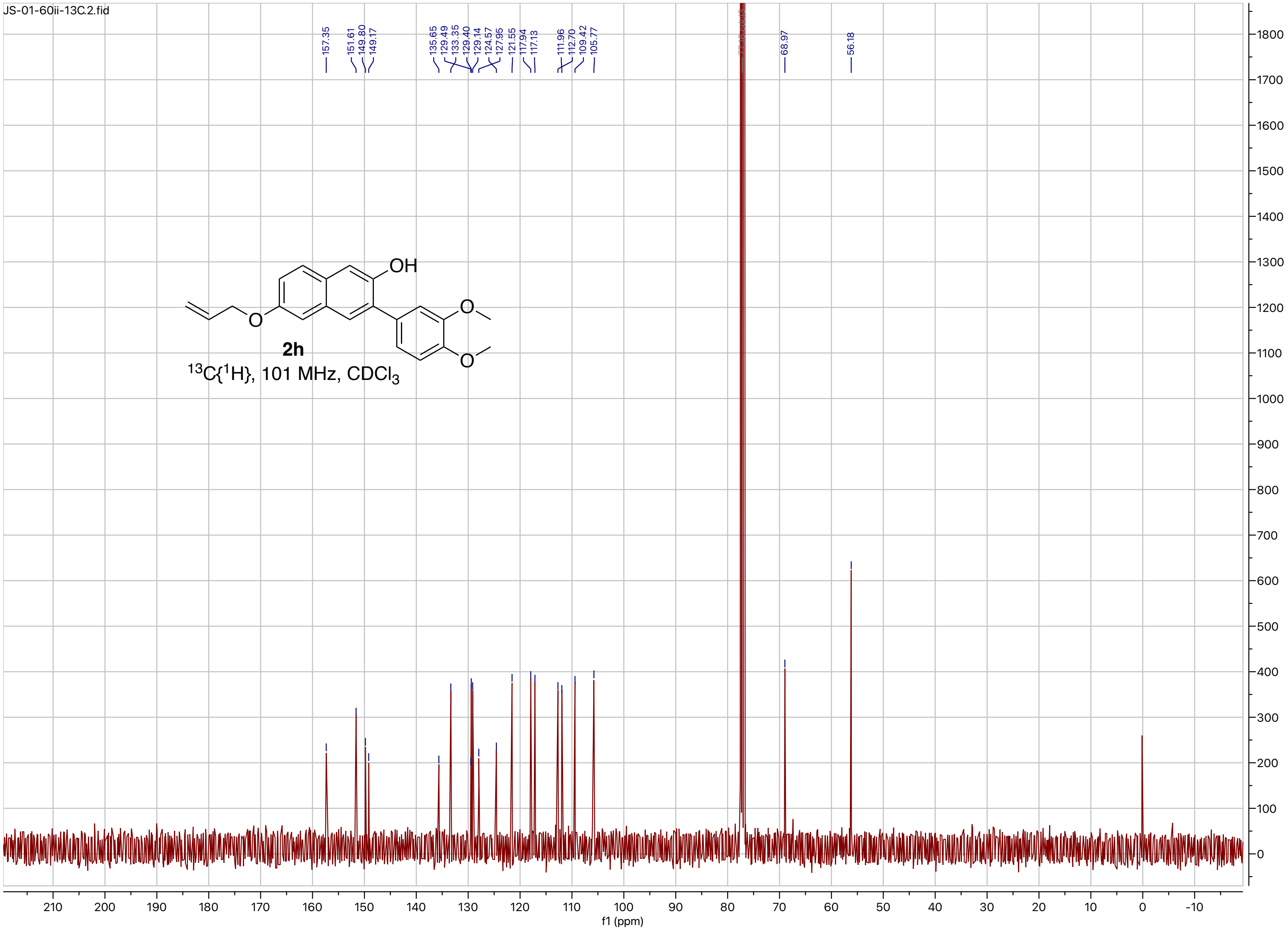
JS-01-60ii-13C.2.fid
157.35
151.61
149.80
149.17
135.65
129.49
133.35
129.40
129.14
124.57
127.95
121.55
117.94
117.13
111.96
112.70
109.42
105.77
68.97
56.18
OH
O
O
O
2h
13C{1H}, 101 MHz, CDCl3
f1 (ppm)

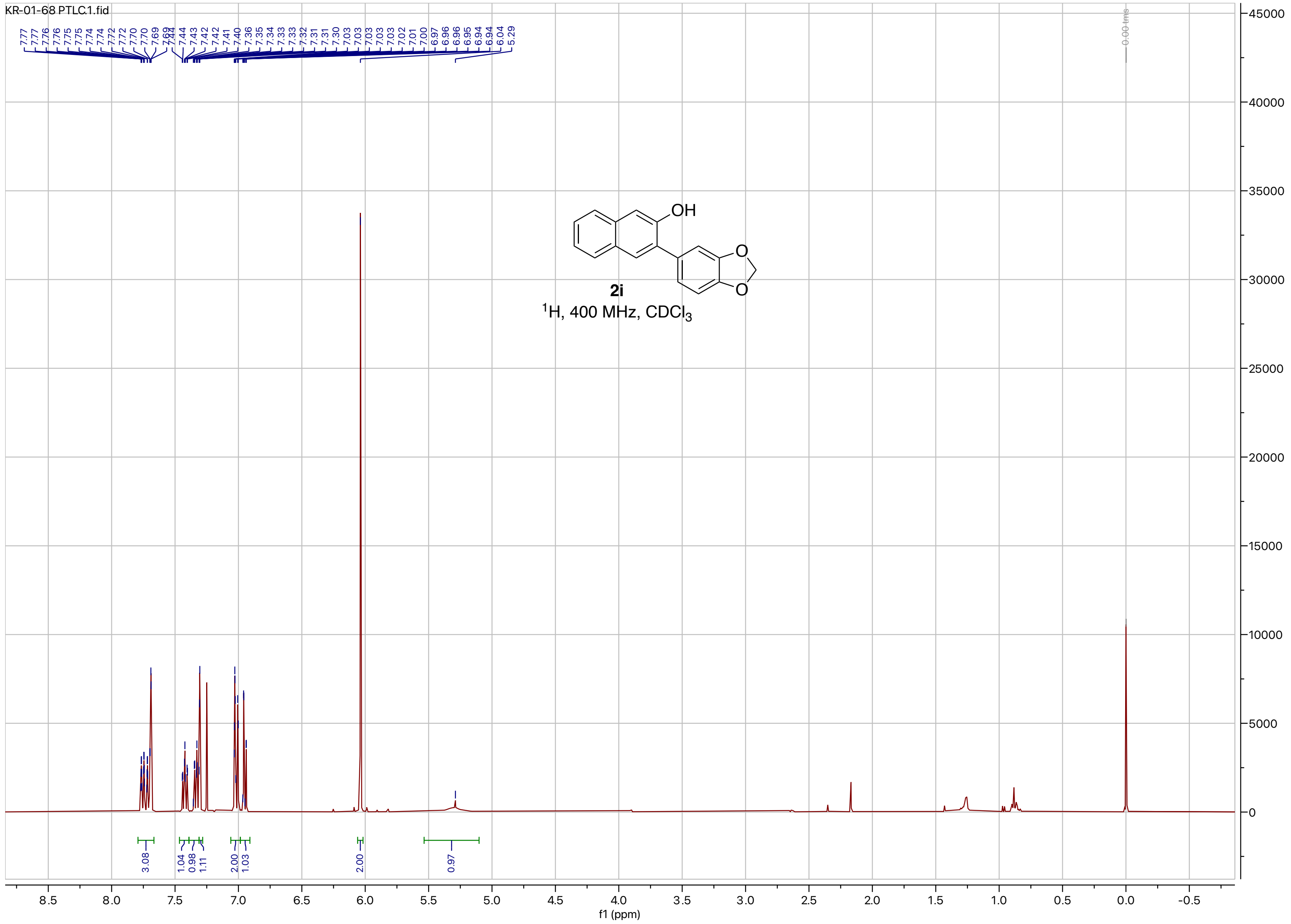
KR-01-68 PTLC.1.fid
2i
¹H, 400 MHz, CDCl3
OH
O
O
f1 (ppm)

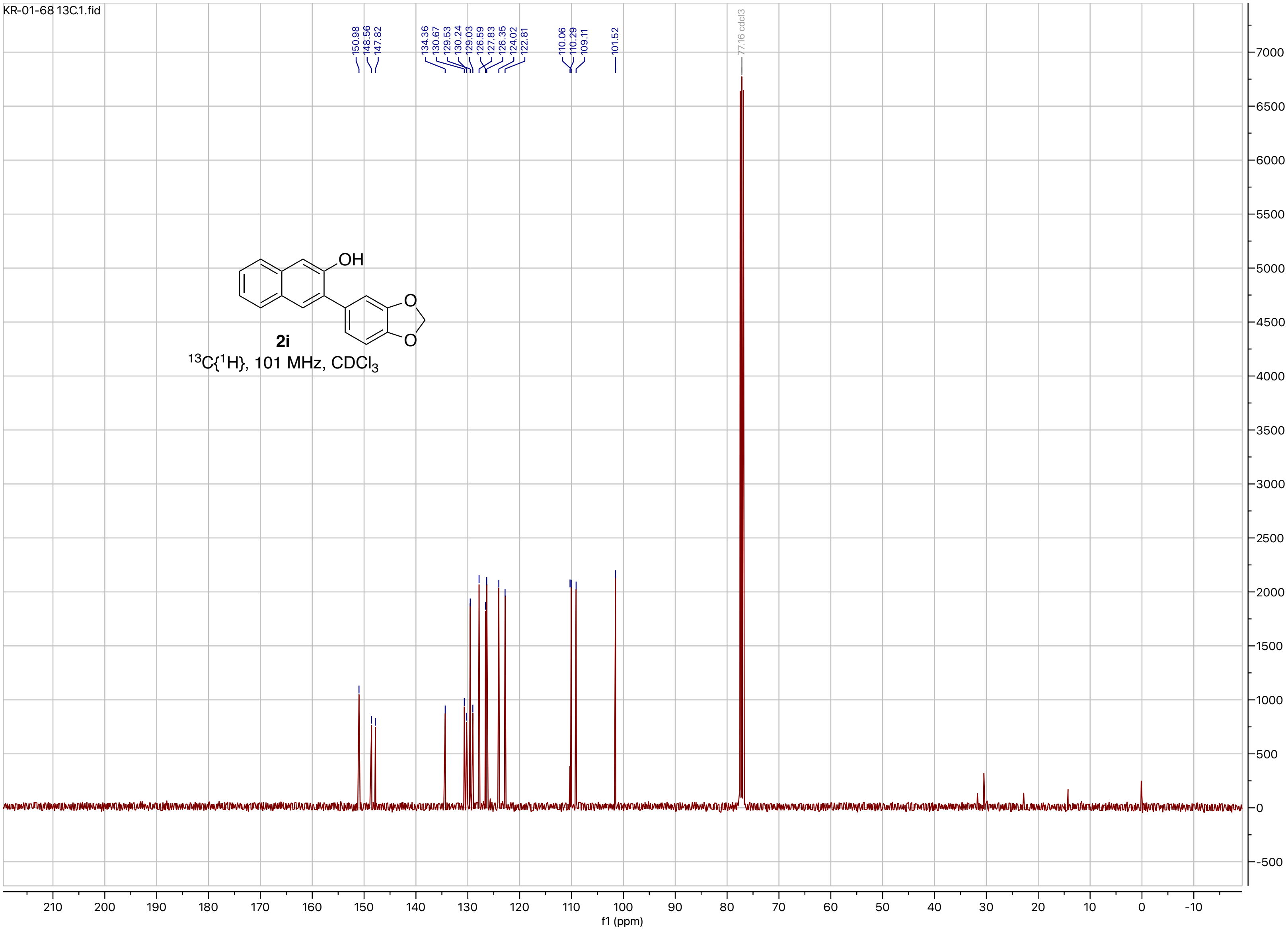
KR-01-68 13C1.fid
150.98
148.56
147.82
134.36
130.67
129.53
130.24
129.03
126.59
127.83
126.35
124.02
122.81
110.06
110.29
109.11
101.52
77.16 cdcl3
OH
O
O
2i
$^{13}C\{^1H\}$, 101 MHz, $CDCl_3$
f1 (ppm)

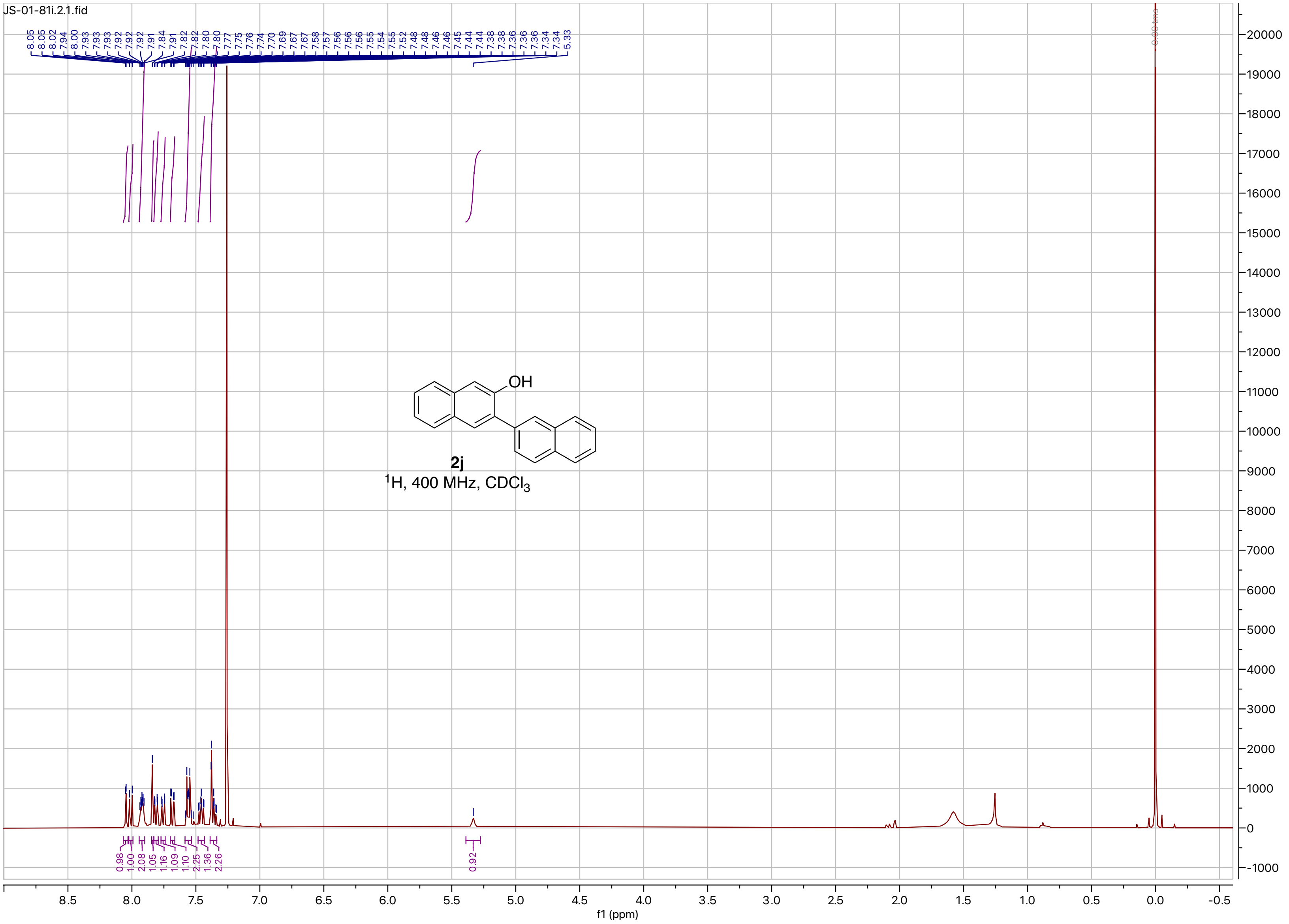
JS-01-81i.2.1.fid
OH
2j
1H, 400 MHz, CDCl3
f1 (ppm)

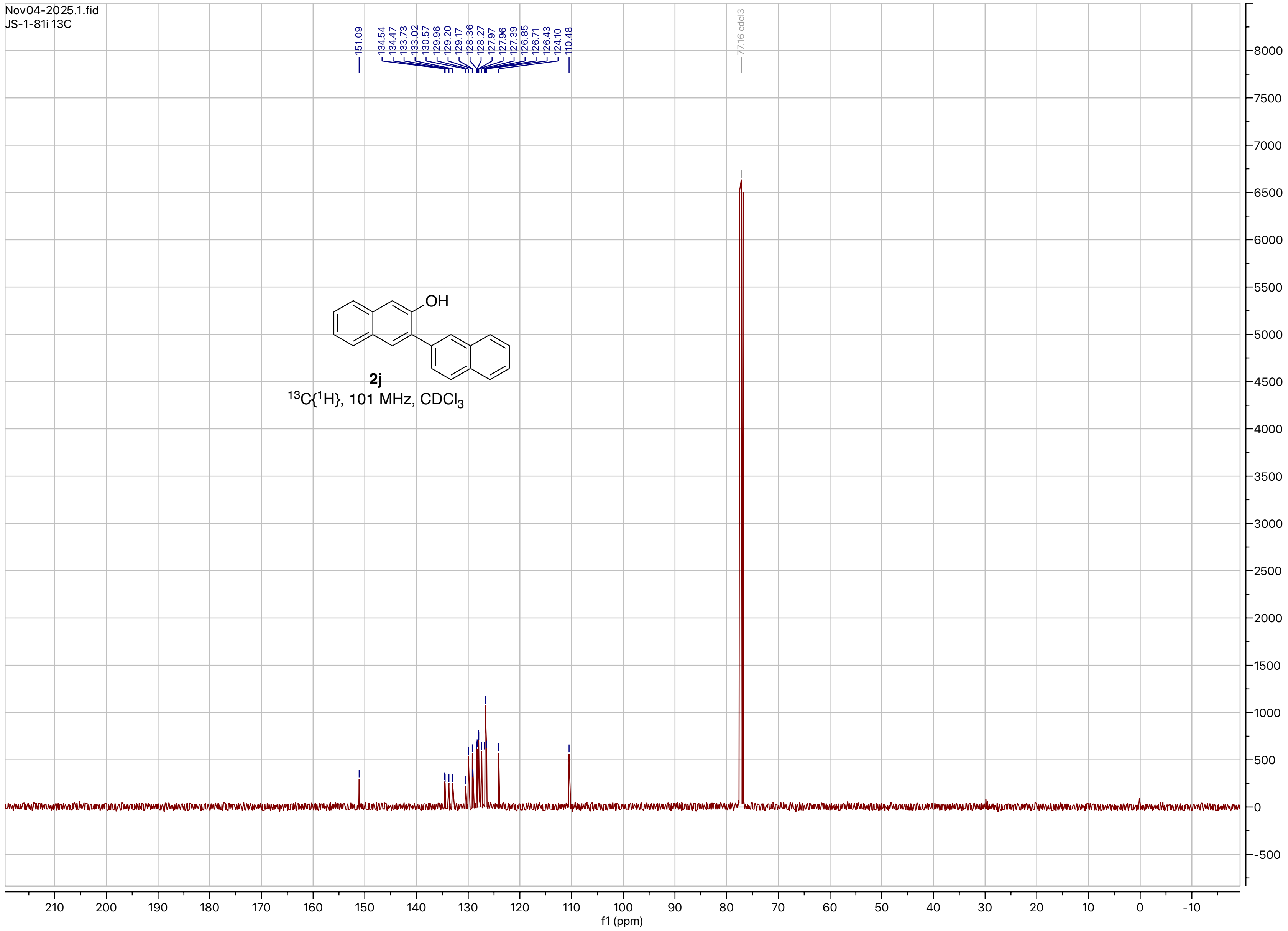
Nov04-2025.1.fid
JS-1-81i 13C
151.09
134.54
134.47
133.73
133.02
130.57
129.96
129.20
129.17
128.36
128.27
127.97
127.96
127.39
126.85
126.71
126.43
124.10
110.48
77.16 cdcl3
OH
2j
13C{1H}, 101 MHz, CDCl3
f1 (ppm)

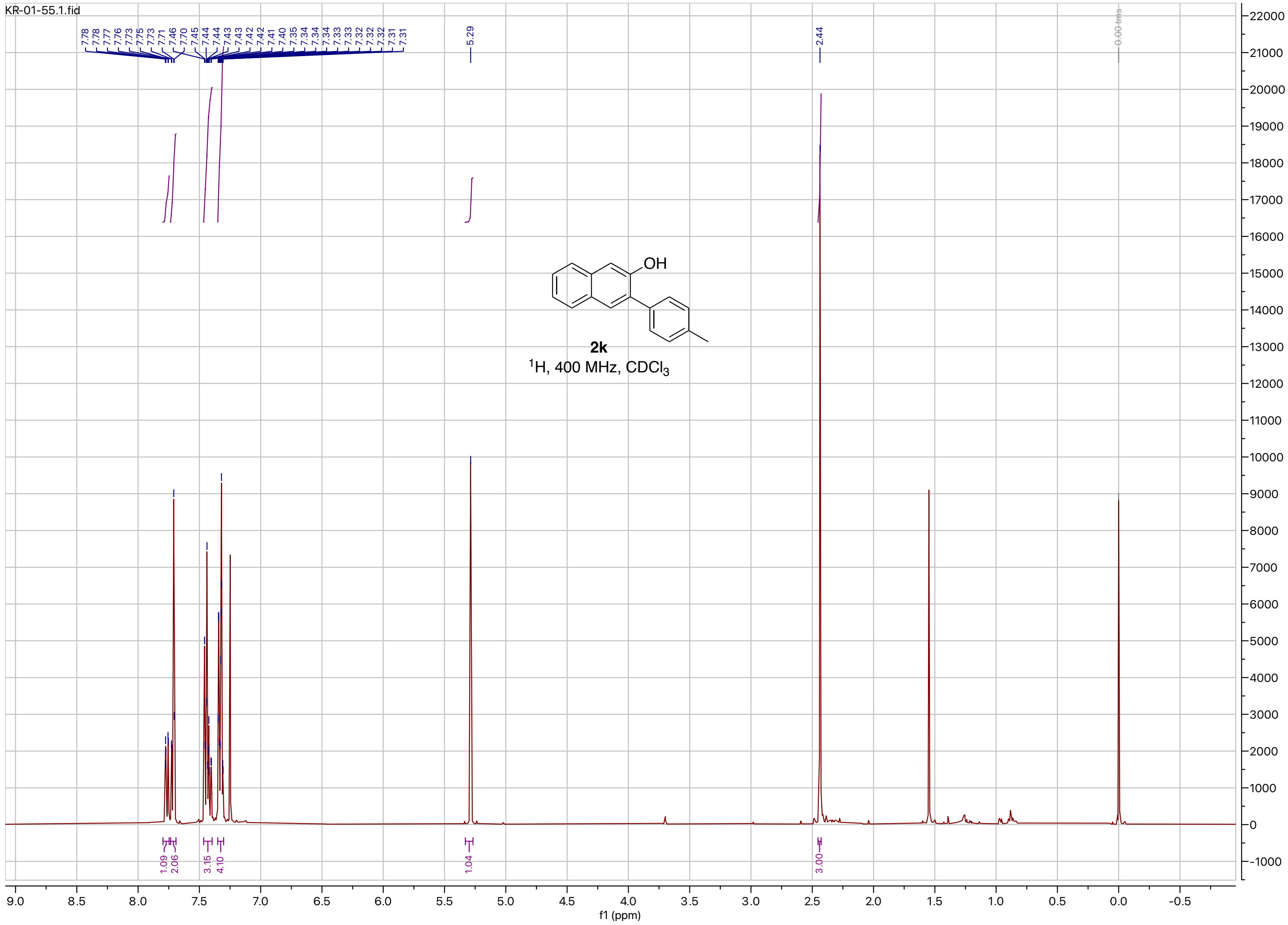

KR-01-55.1.fid
7.78
7.78
7.77
7.76
7.73
7.75
7.73
7.71
7.46
7.70
7.45
7.44
7.44
7.43
7.43
7.42
7.42
7.41
7.40
7.35
7.34
7.34
7.34
7.33
7.33
7.32
7.32
7.32
7.31
7.31
5.29
2.44
0.00 tms
OH
2k
¹H, 400 MHz, CDCl3
1.09
2.06
3.15
4.10
1.04
3.00
f1 (ppm)

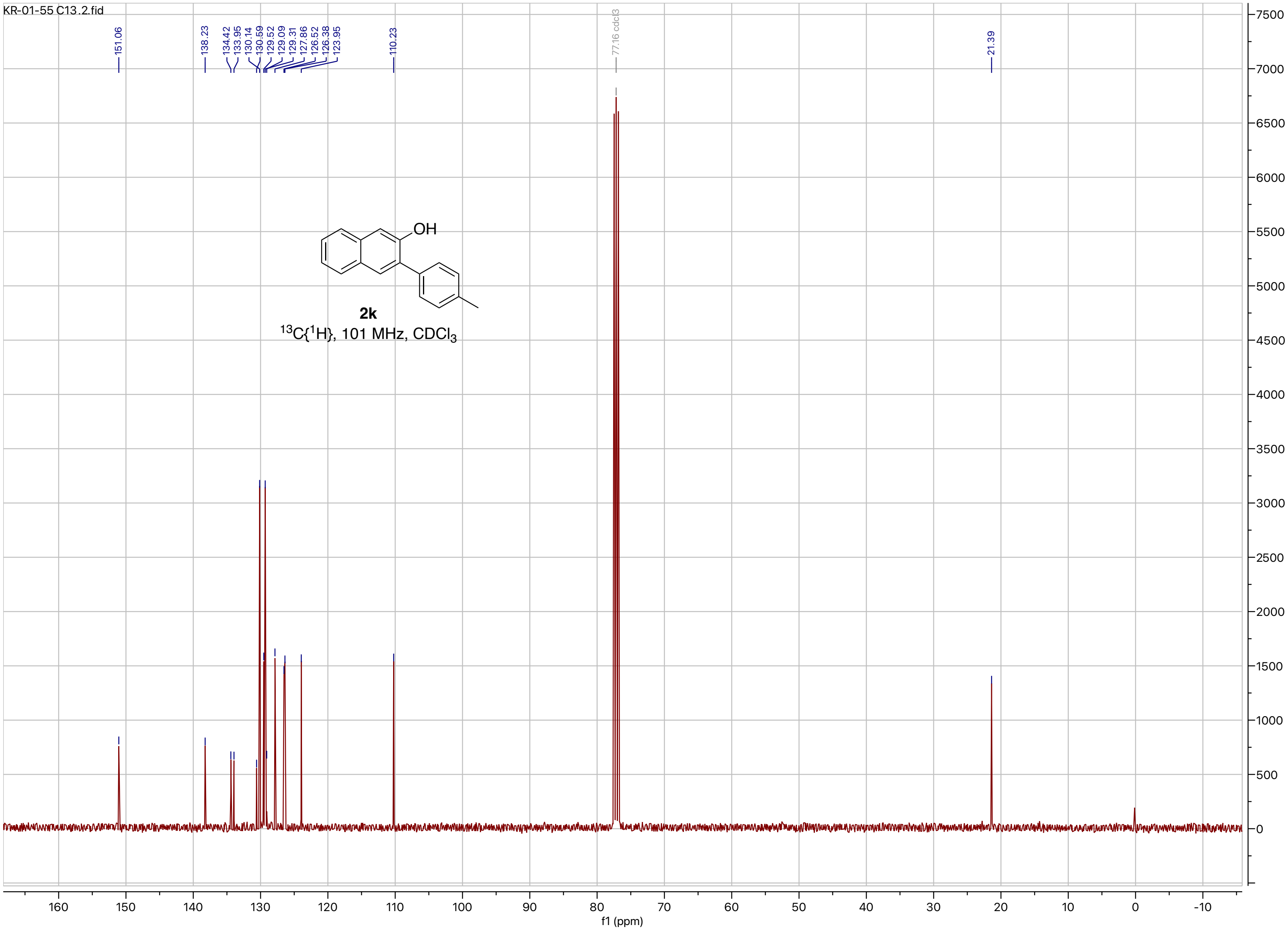

KR-01-55 C13.2.fid
151.06
138.23
134.42
133.95
130.14
130.59
129.52
129.09
129.31
127.86
126.52
126.38
123.95
110.23
77.16 cdcl3
21.39
OH
2k
$^{13}C\{^{1}H\}$, 101 MHz, $CDCl_3$
f1 (ppm)

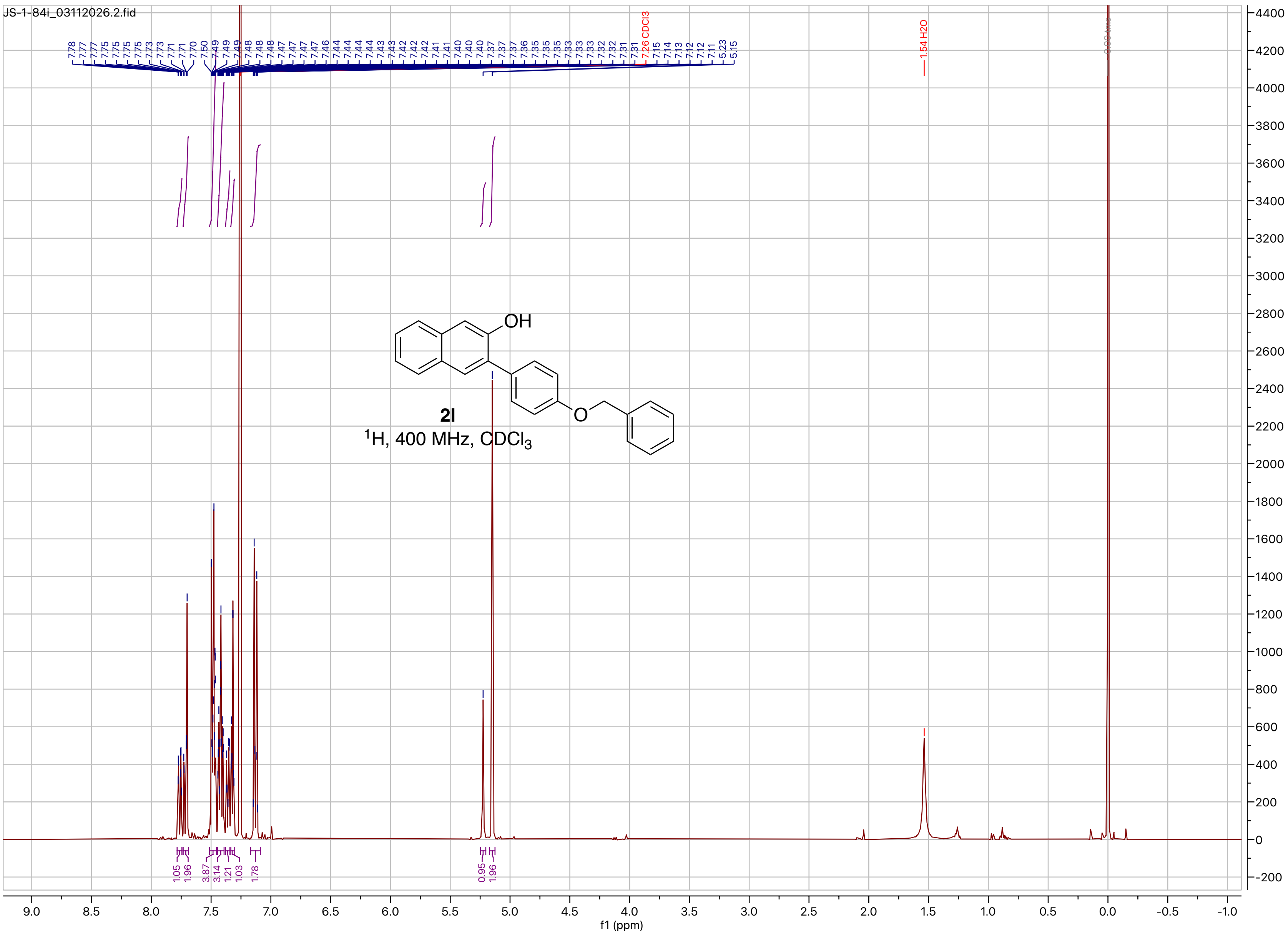

JS-1-84i_03112026.2.fid
OH
O
2l
^{1}H, 400 MHz, $CDCl_3$
f1 (ppm)

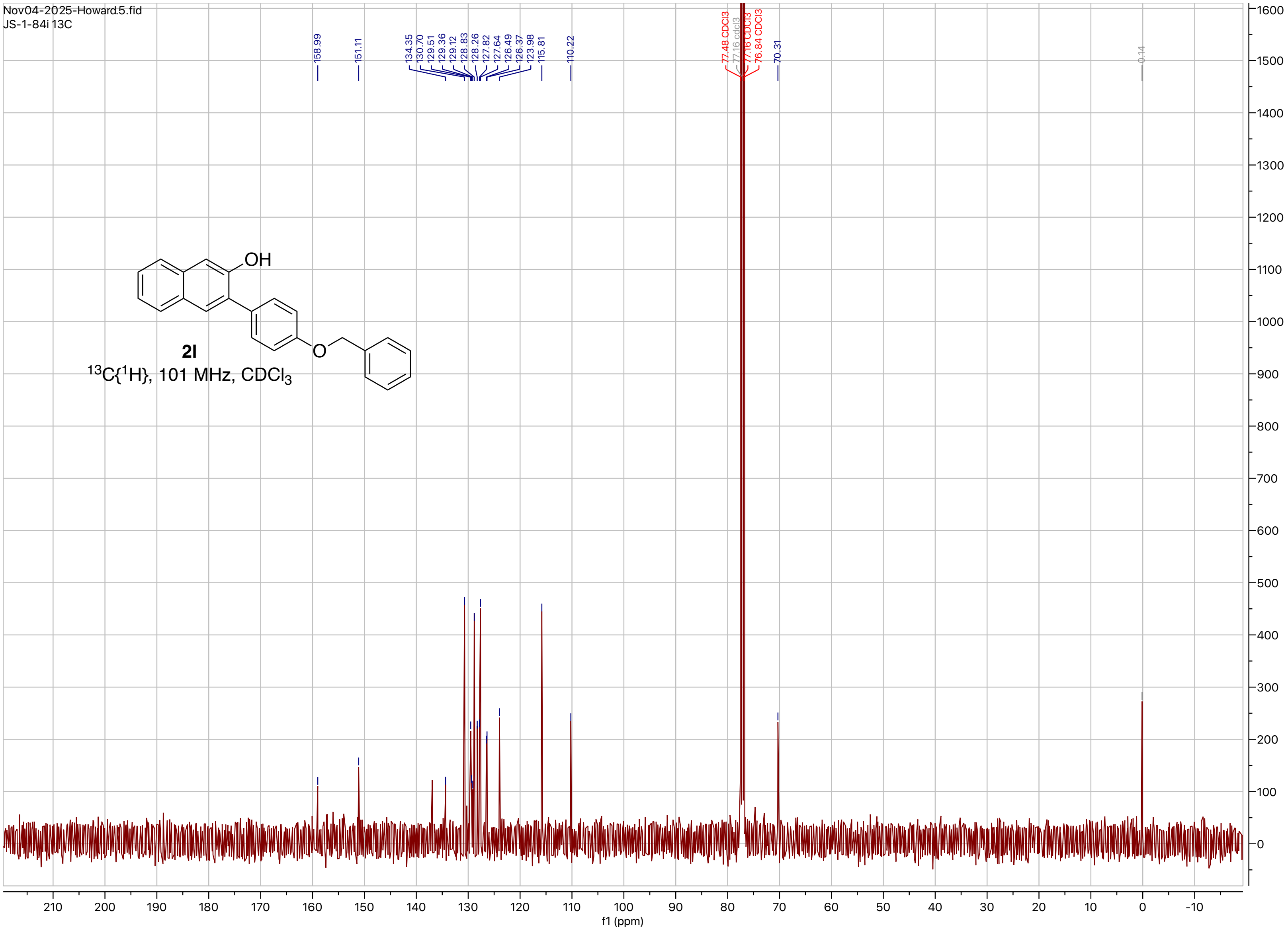
Nov04-2025-Howard.5.fid
JS-1-84i 13C
158.99
151.11
134.35
130.70
129.51
129.36
129.12
128.83
128.26
127.82
127.64
126.49
126.37
123.98
115.81
110.22
77.48 CDCl3
77.16 cdcl3
77.16 CDCl3
76.84 CDCl3
70.31
0.14
OH
O
2l
13C{1H}, 101 MHz, CDCl3
f1 (ppm)

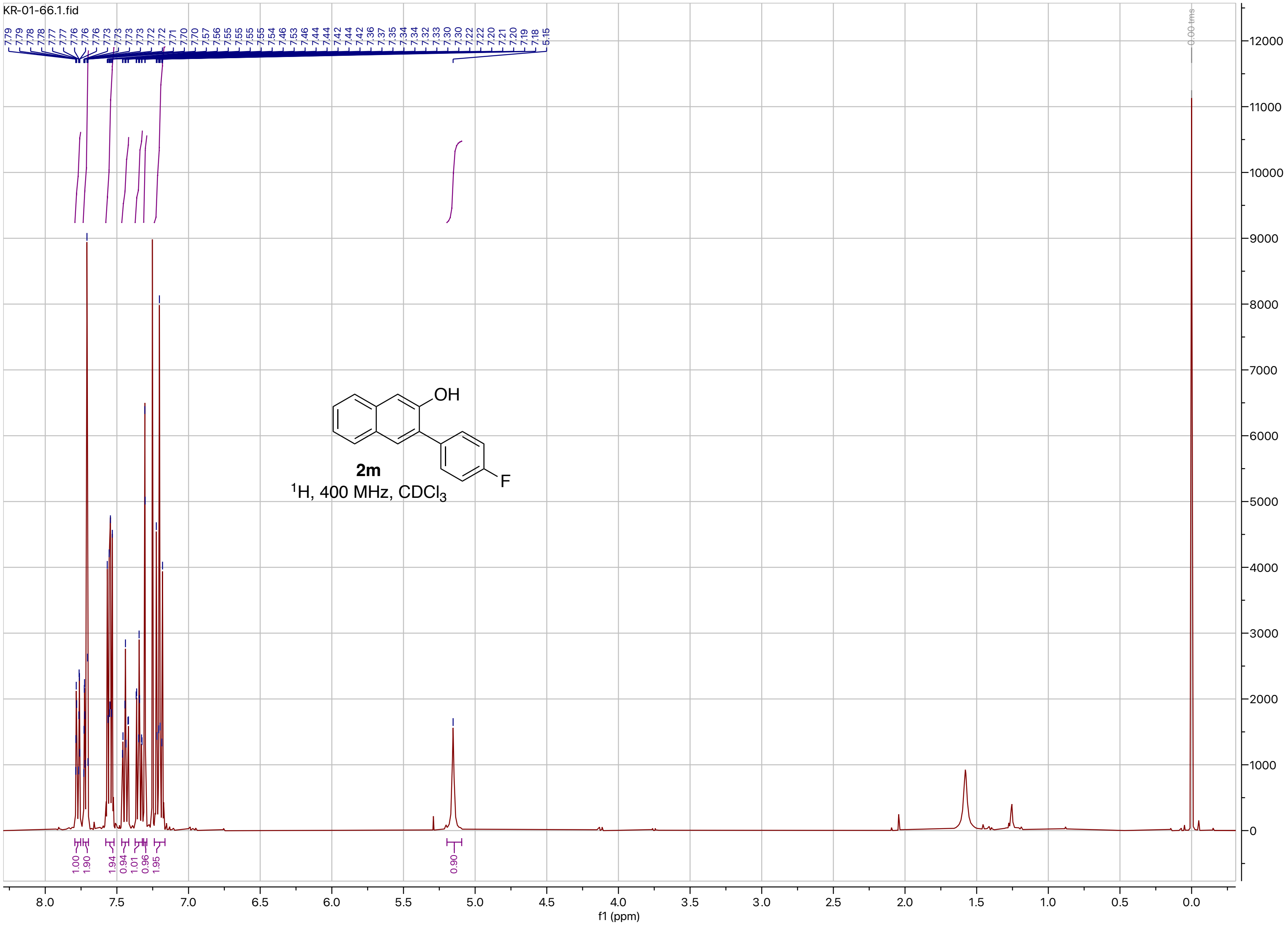
KR-01-66.1.fid
2m
1H, 400 MHz, CDCl3
OH
F
f1 (ppm)

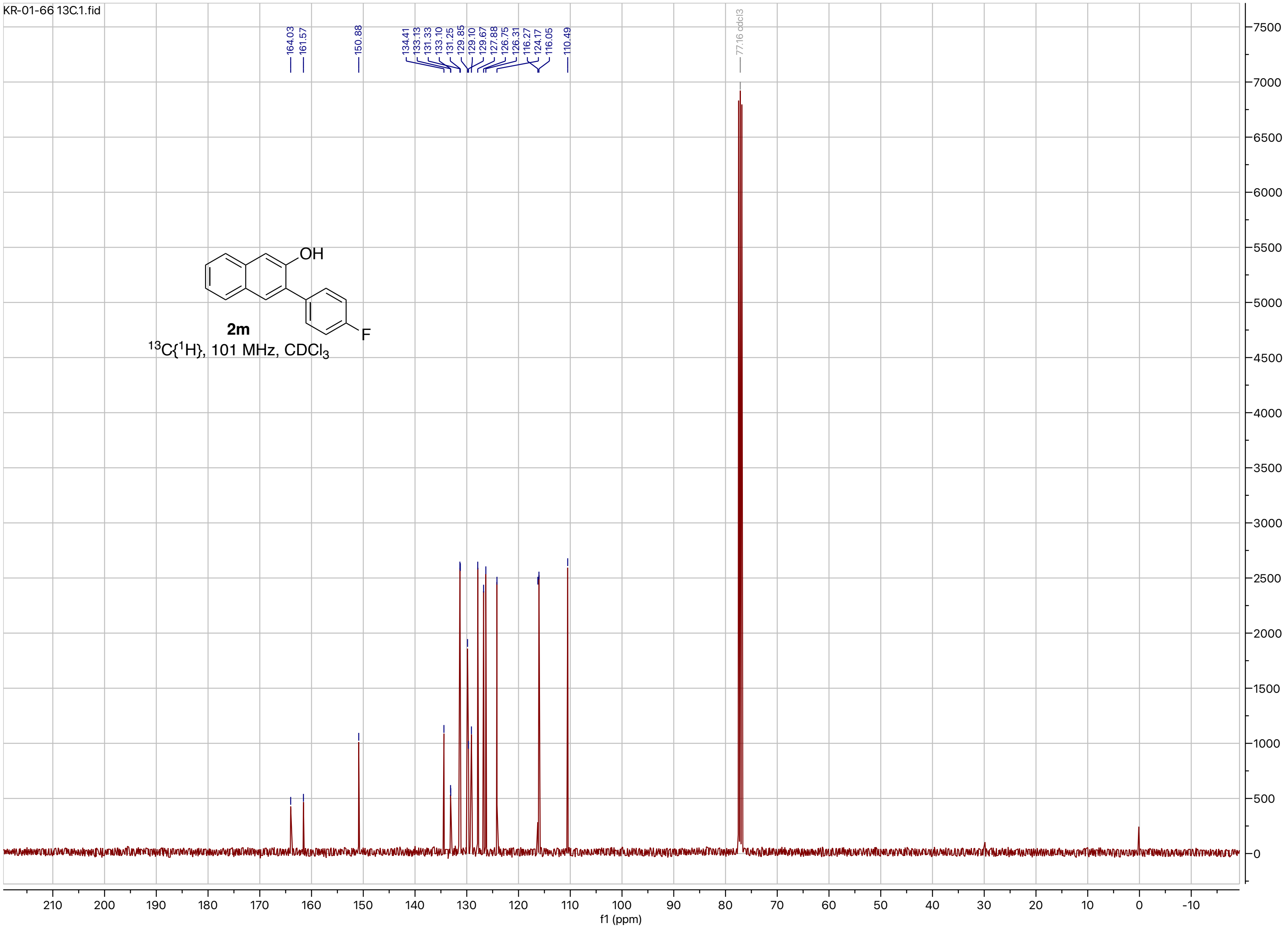

KR-01-66 13C.1.fid
164.03
161.57
150.88
134.41
133.13
131.33
133.10
131.25
129.85
129.10
129.67
127.88
126.75
126.31
116.27
124.17
116.05
110.49
77.16 cdcl3
OH
F
2m
13C{1H}, 101 MHz, CDCl3
f1 (ppm)

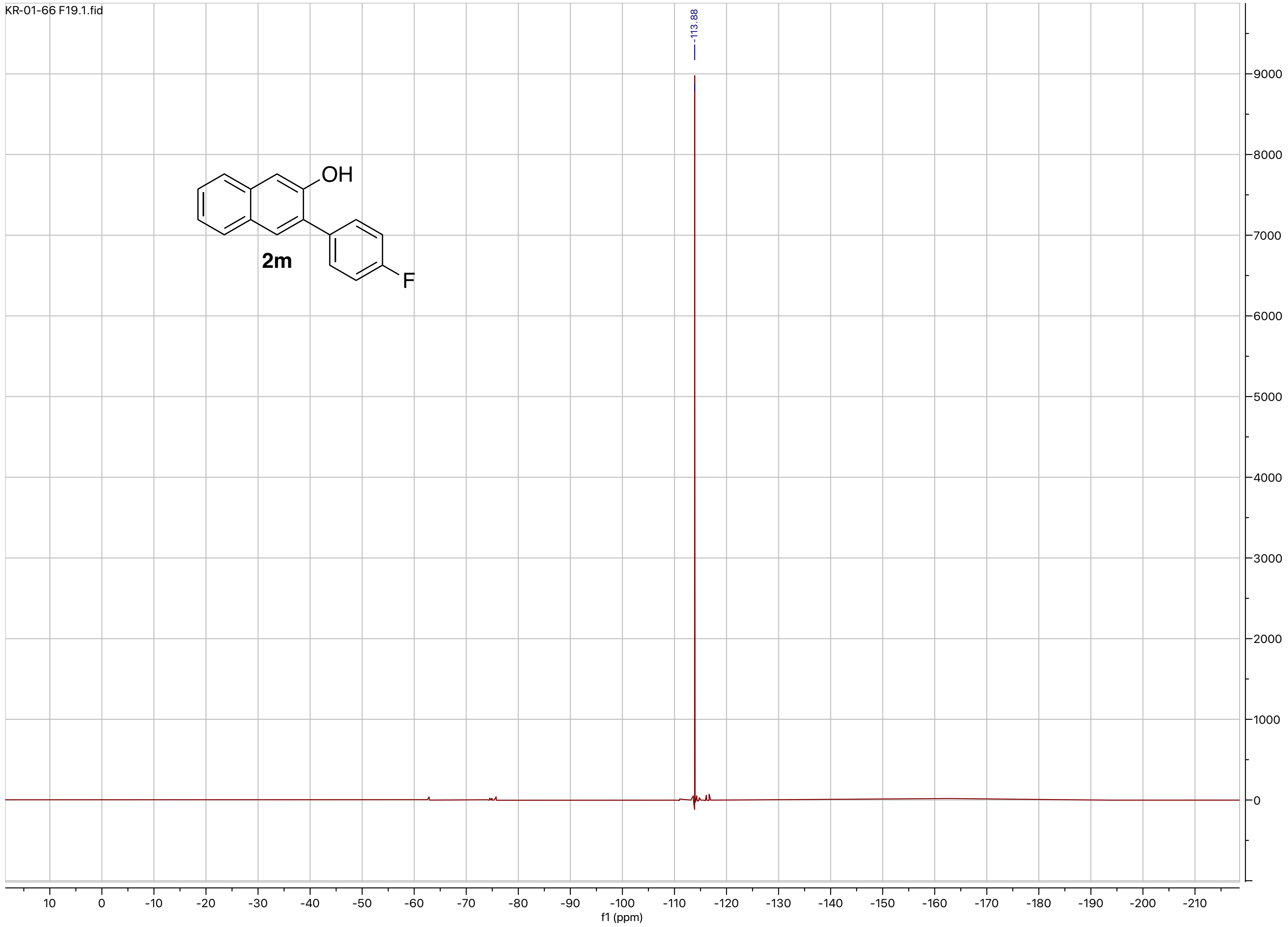
KR-01-66 F19.1.fid
-113.88
OH
2m
F
f1 (ppm)

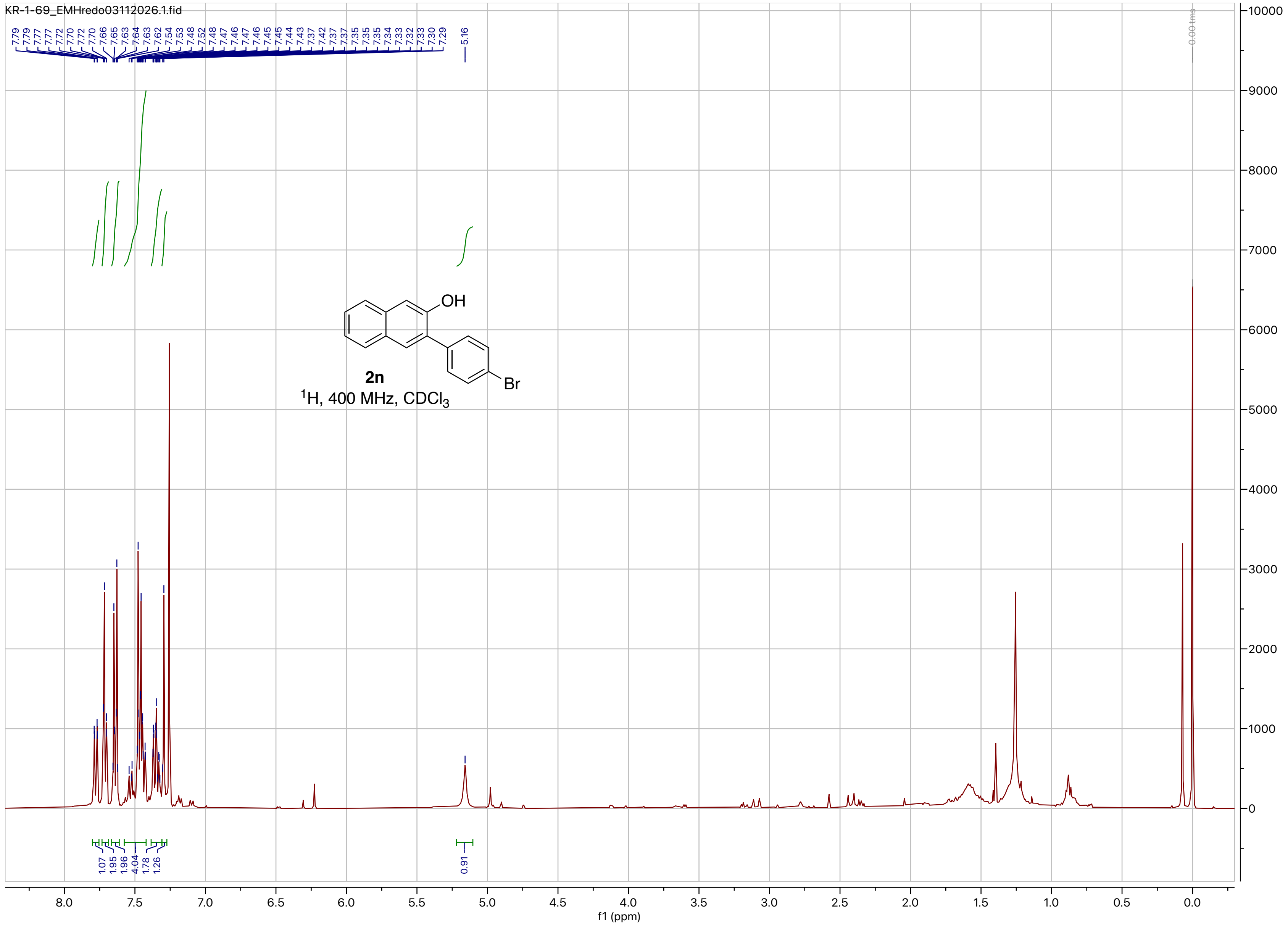

KR-1-69_EMHredo03112026.1.fid
OH
Br
2n
¹H, 400 MHz, CDCl3
f1 (ppm)

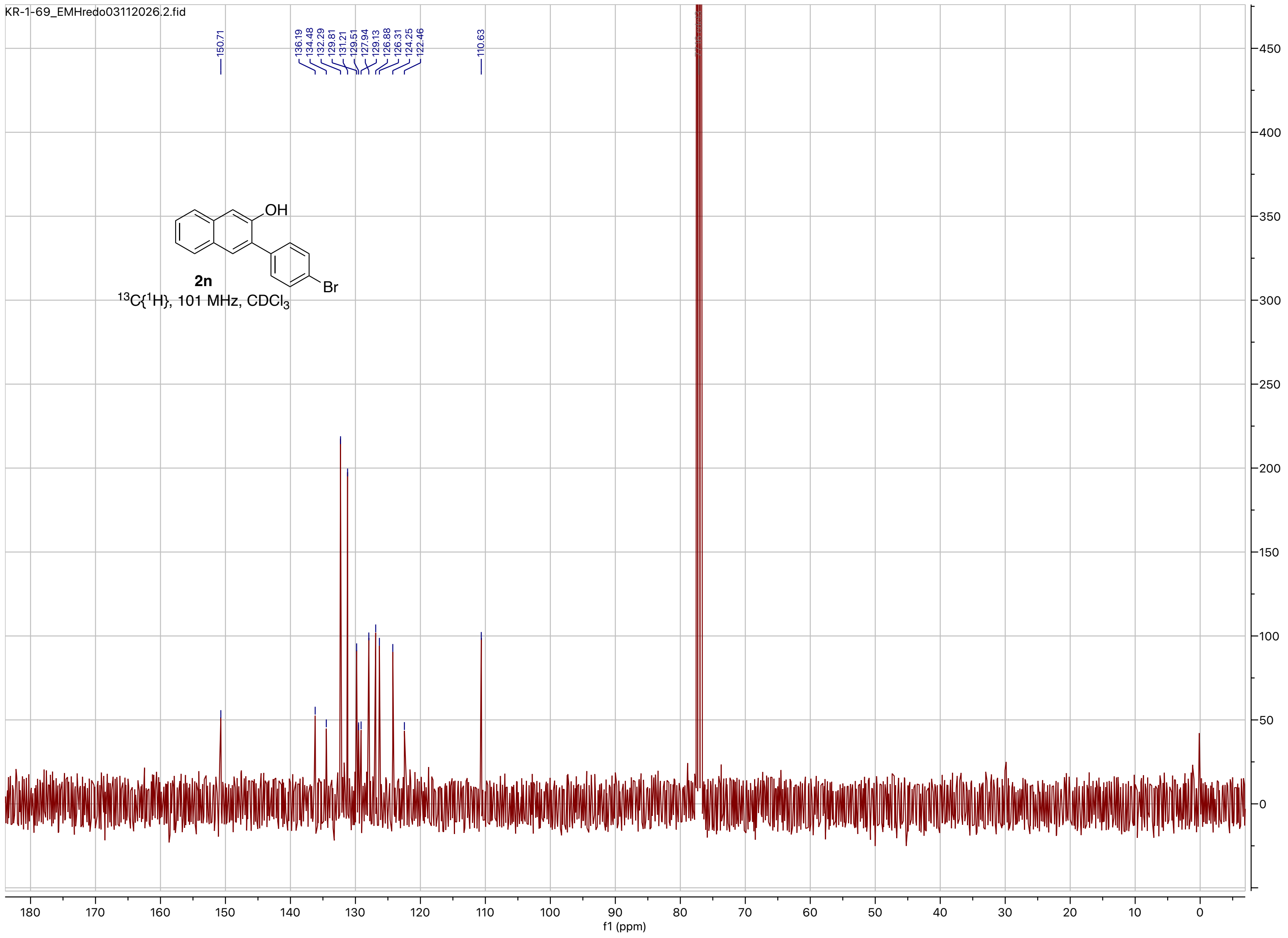
KR-1-69_EMHredo03112026.2.fid
150.71
136.19
134.48
132.29
129.81
131.21
129.51
127.94
129.13
126.88
126.31
124.25
122.46
110.63
OH
Br
2n
$^{13}C\{^1H\}$, 101 MHz, $CDCl_3$
f1 (ppm)

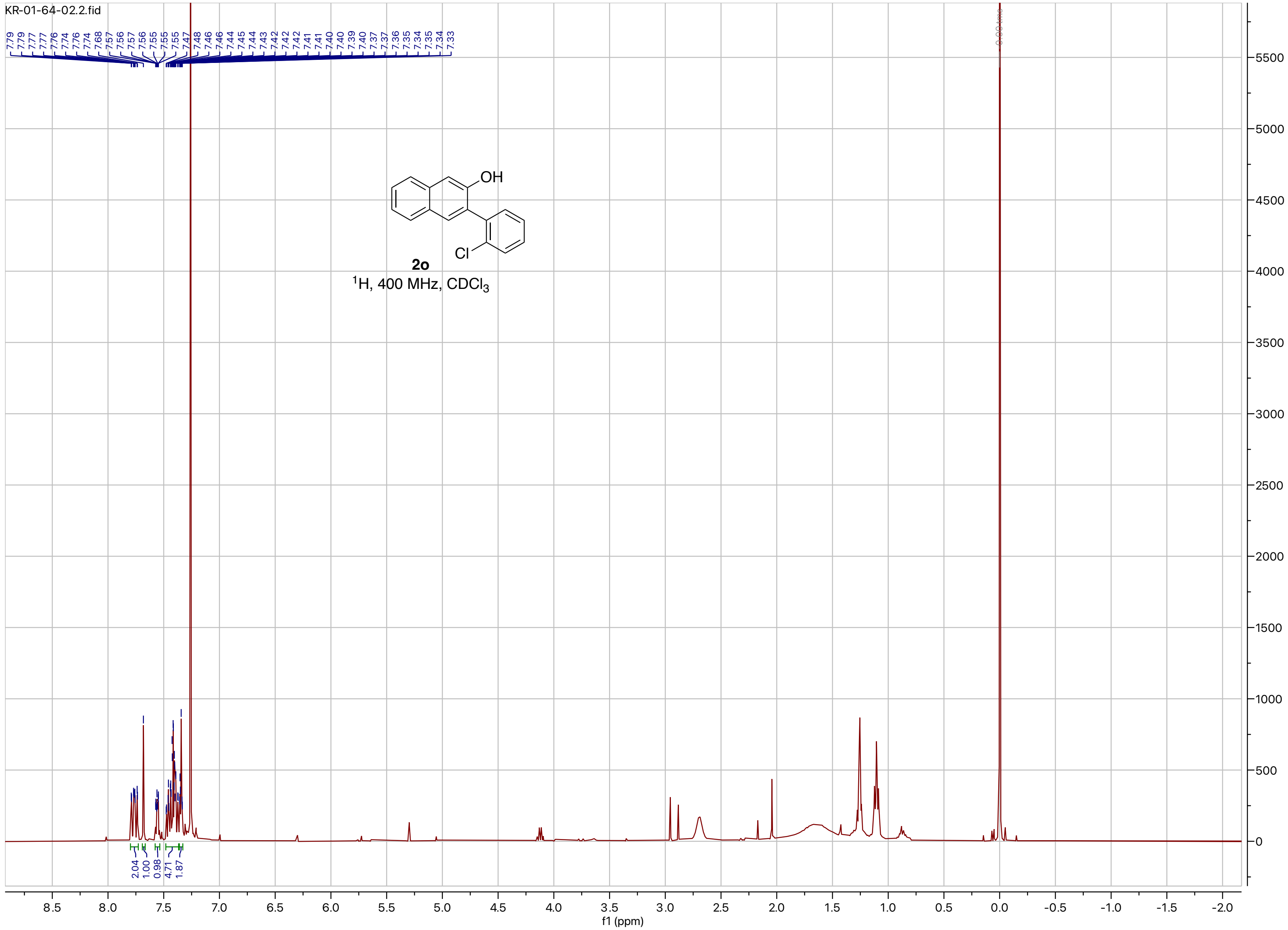
KR-01-64-02.2.fid
2o
1H, 400 MHz, CDCl3
OH
Cl
f1 (ppm)

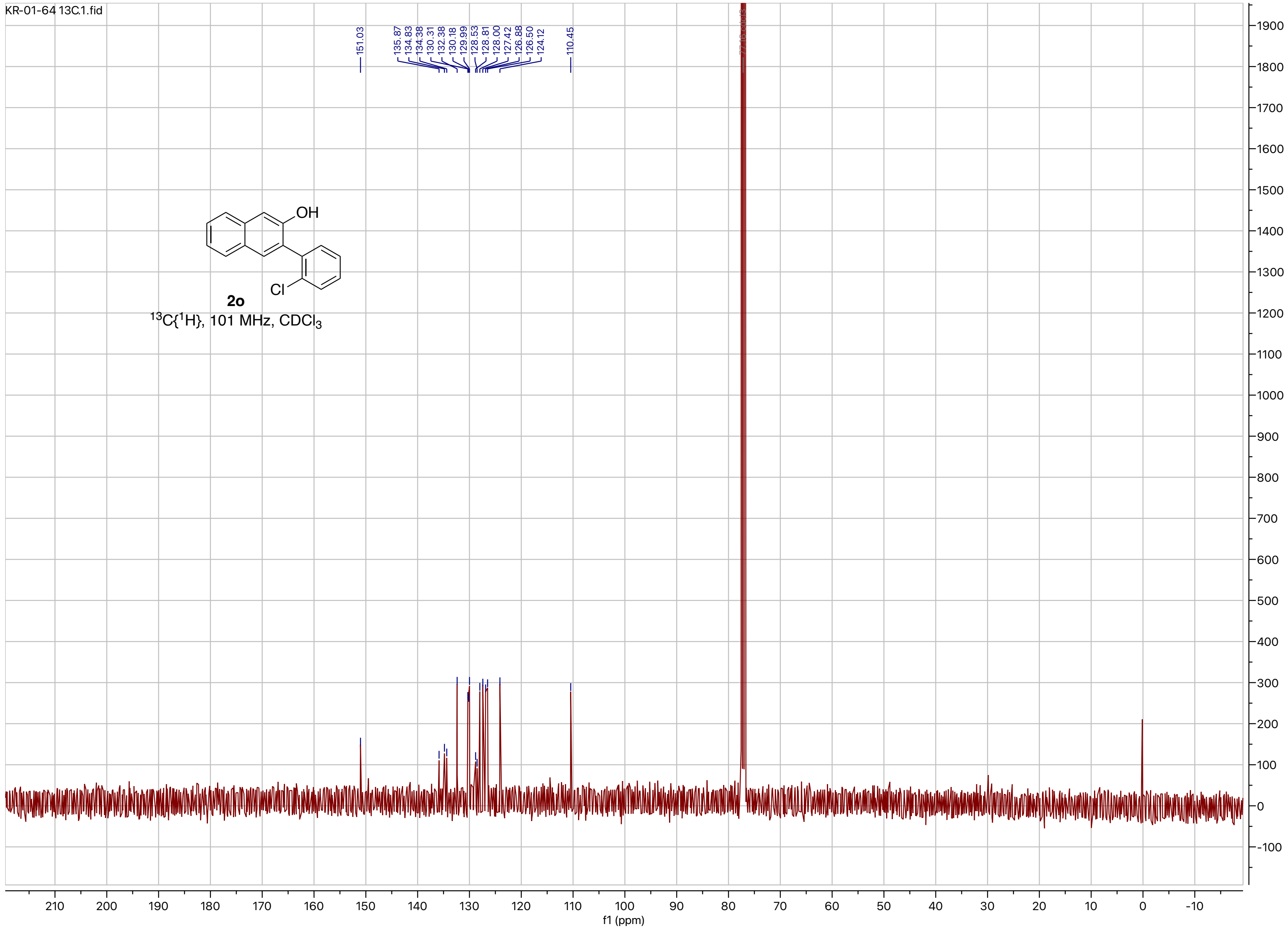

KR-01-64 13C1.fid
151.03
135.87
134.83
134.38
130.31
132.38
130.18
129.99
128.53
128.81
128.00
127.42
126.88
126.50
124.12
110.45
OH
Cl
2o
13C{1H}, 101 MHz, CDCl3
f1 (ppm)

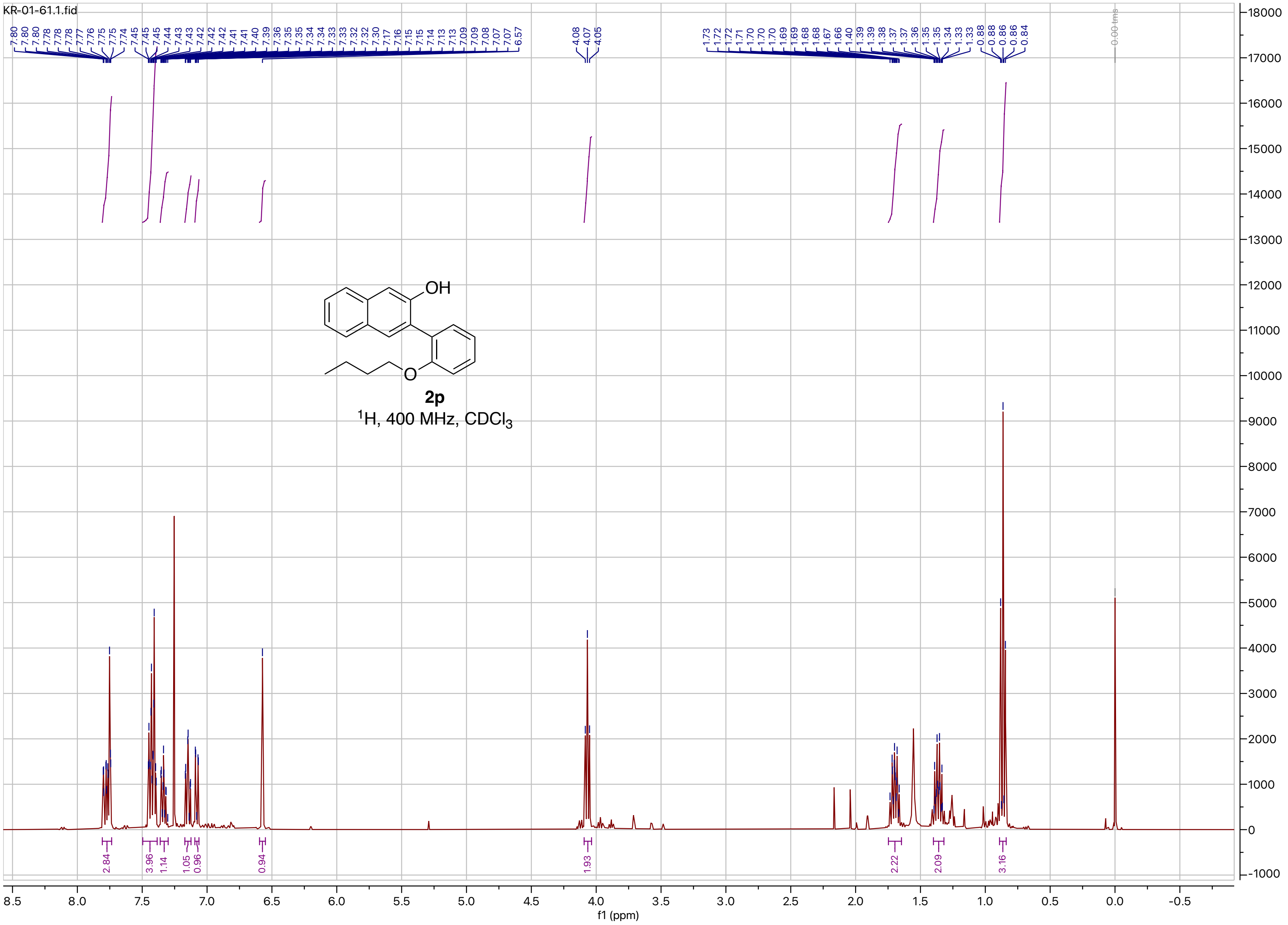
KR-01-61.1.fid
OH
O
2p
¹H, 400 MHz, CDCl₃
f1 (ppm)

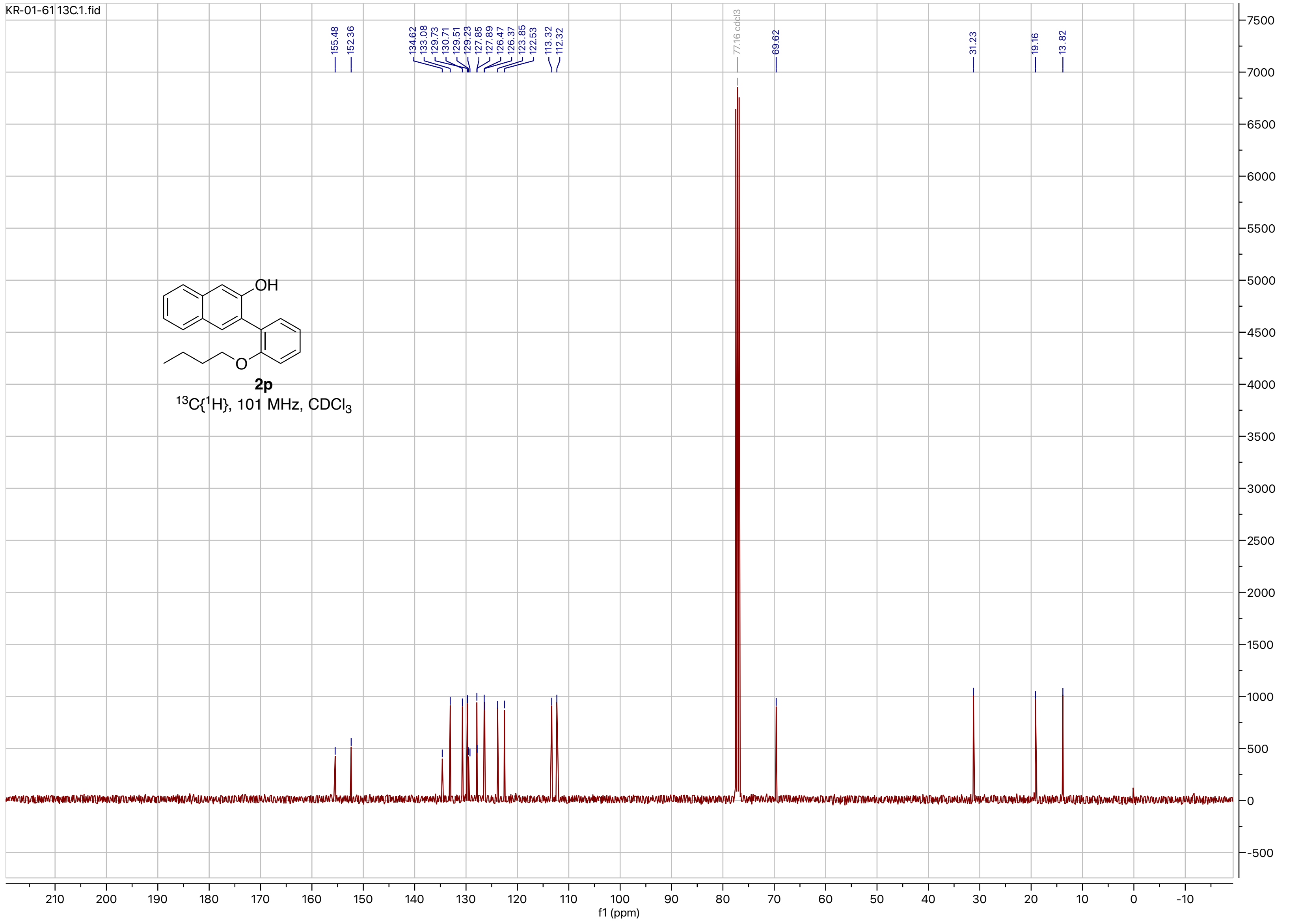
KR-01-61 13C.1.fid
155.48
152.36
134.62
133.08
129.73
130.71
129.51
129.23
127.85
127.89
126.47
126.37
123.85
122.53
113.32
112.32
77.16 cdcl3
69.62
31.23
19.16
13.82
OH
O
2p
$^{13}C\{^1H\}$, 101 MHz, $CDCl_3$
f1 (ppm)

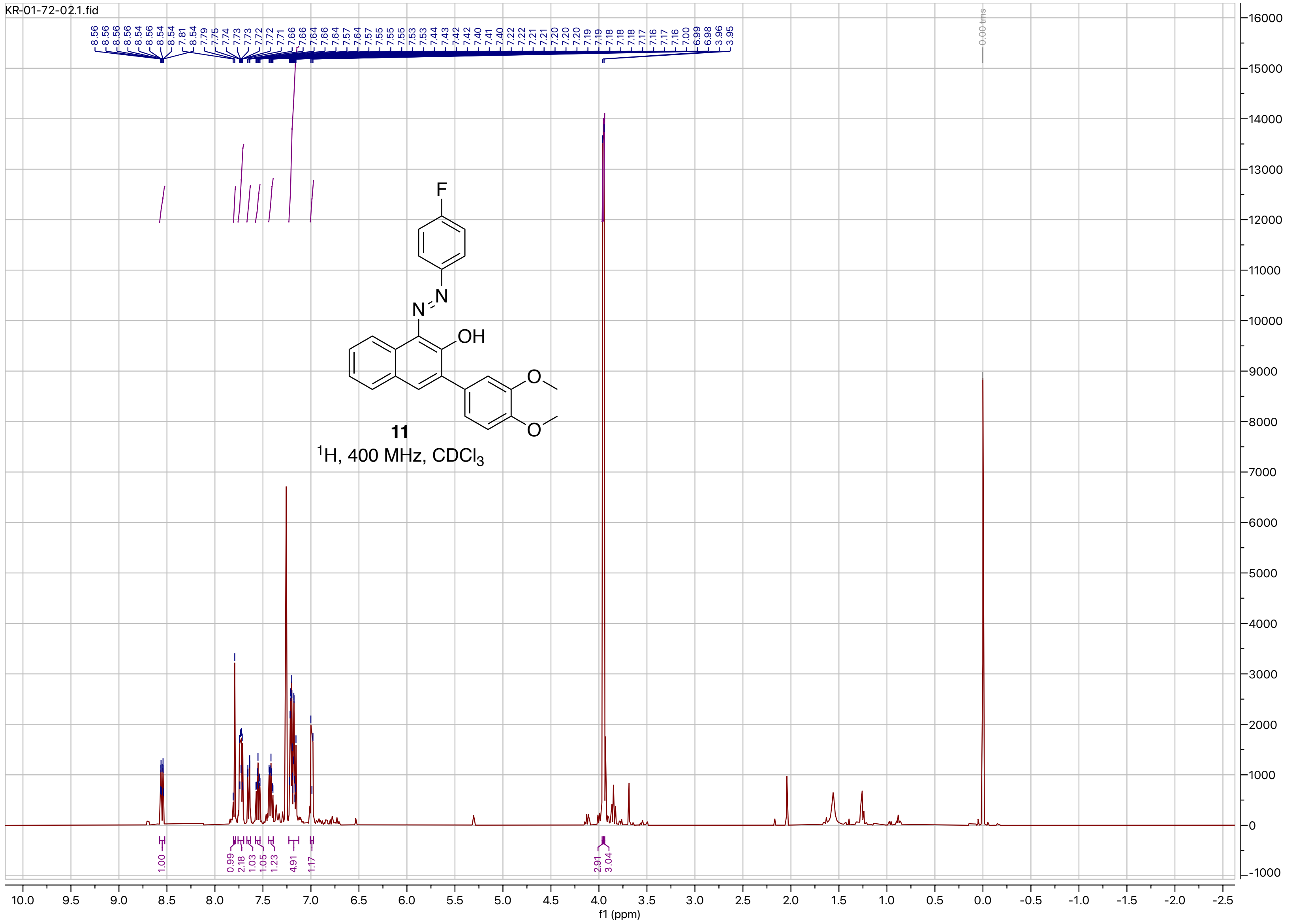
KR-01-72-02.1.fid
11
1H, 400 MHz, CDCl3
F
N
N
OH
O
O
f1 (ppm)
1.00
0.99
2.18
1.03
1.05
1.23
4.91
1.17
2.91
3.04

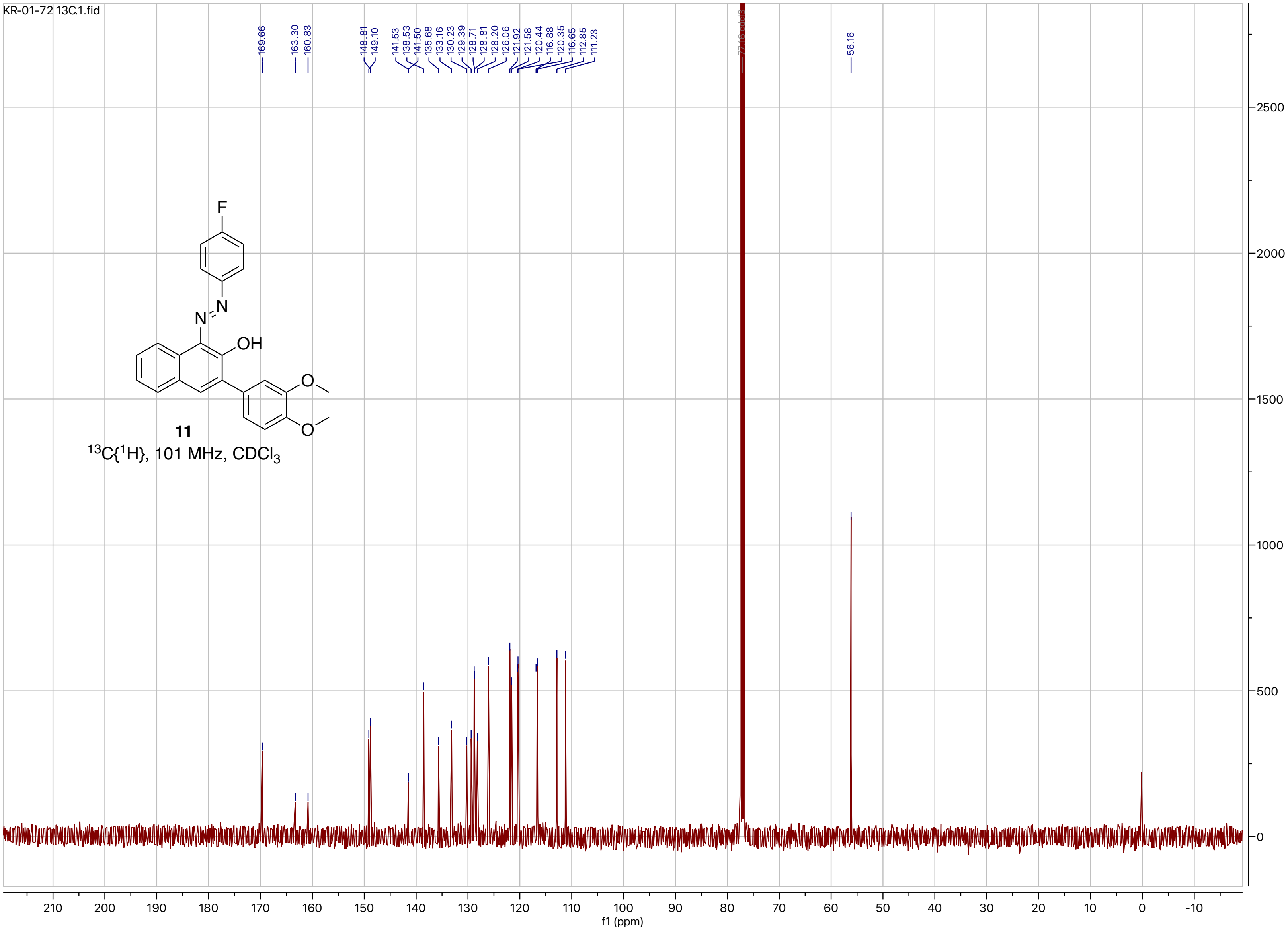

11

$^{13}C\{^{1}H\}$, 101 MHz, $CDCl_3$

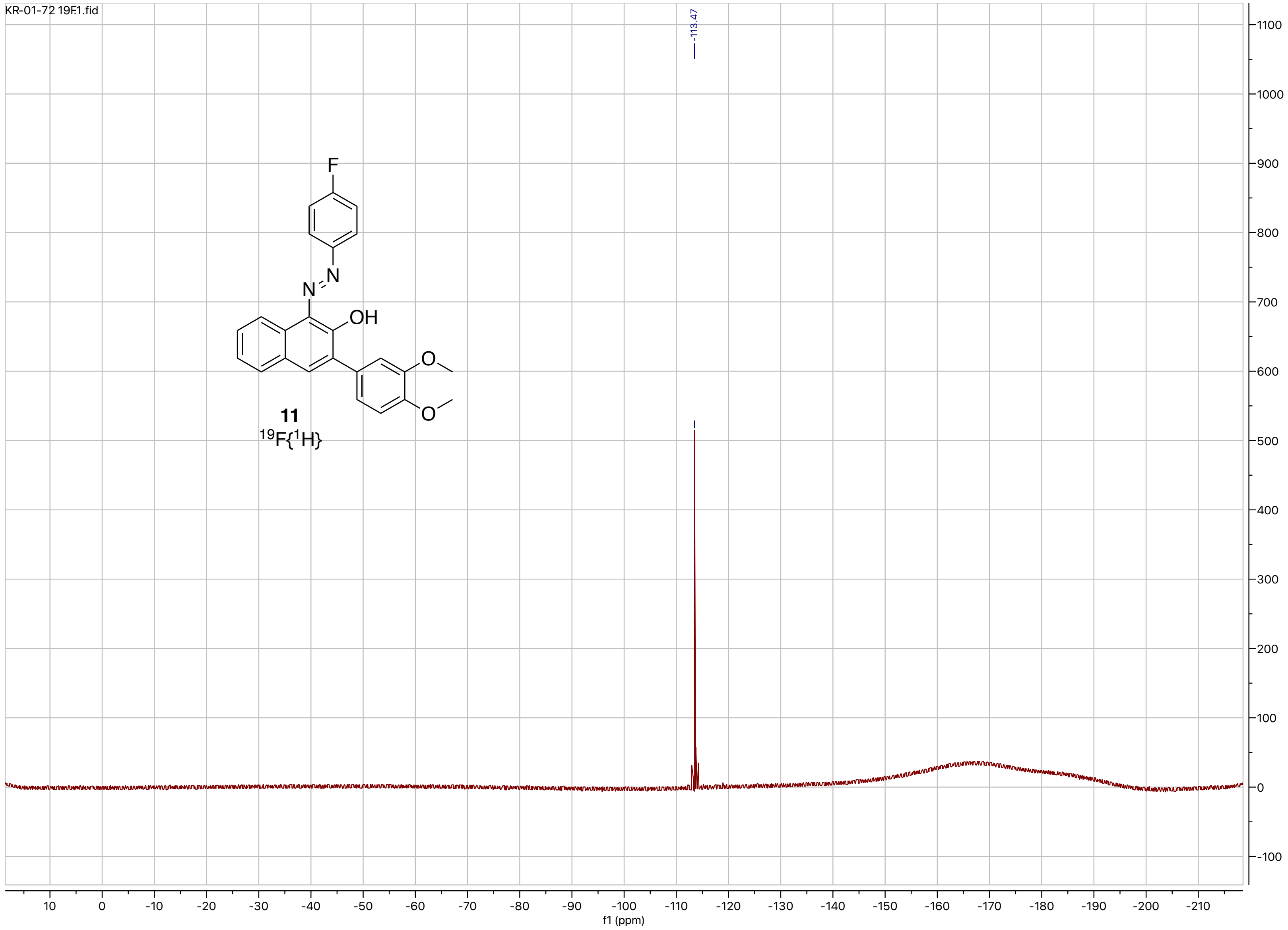
KR-01-72 19F1.fid
-113.47
F
N
N
OH
O
O
11
19F{1H}
f1 (ppm)

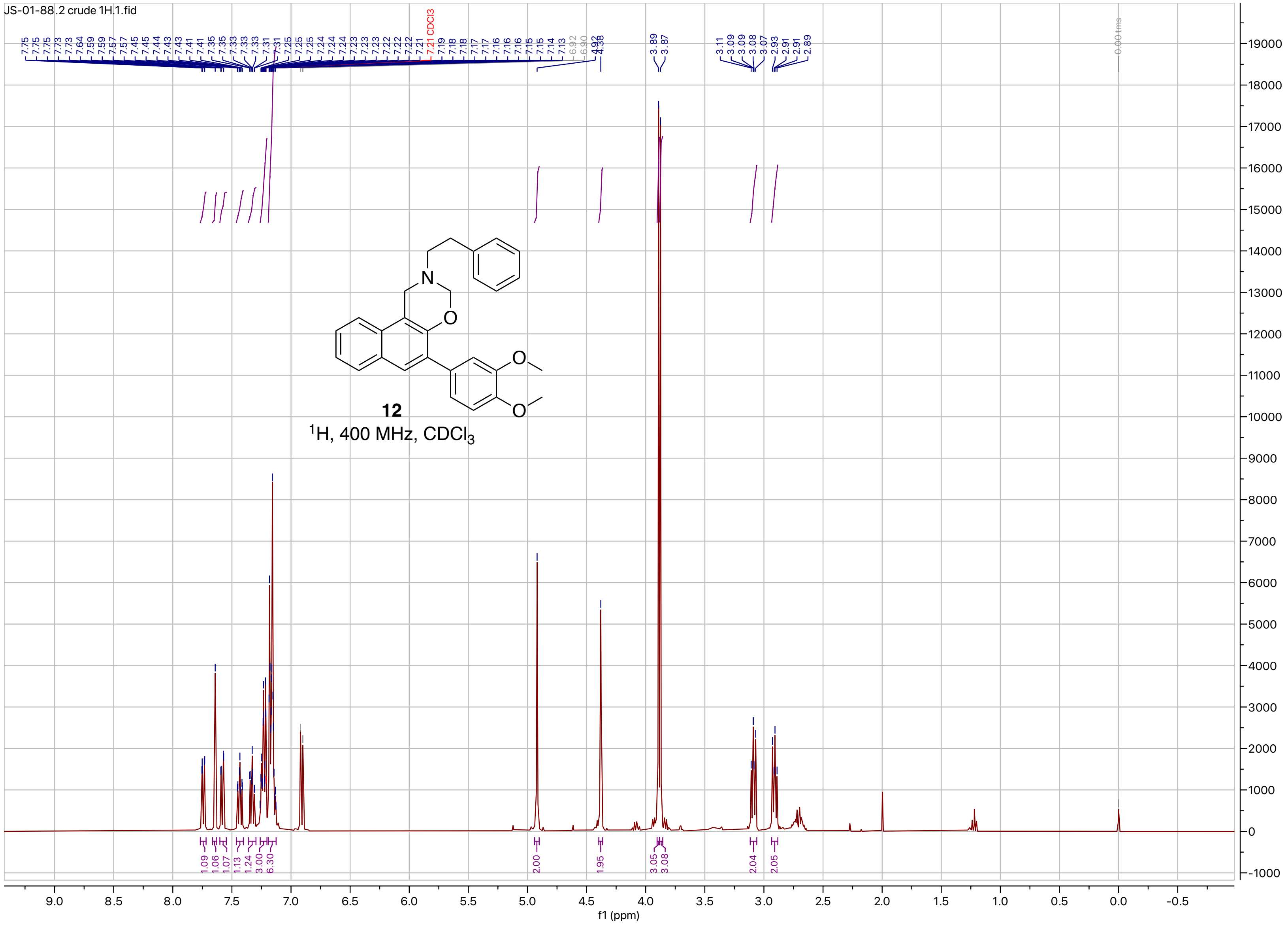

JS-01-88.2 crude 1H.1.fid
7.75
7.75
7.75
7.73
7.73
7.64
7.59
7.59
7.57
7.57
7.45
7.45
7.44
7.43
7.43
7.41
7.41
7.35
7.35
7.33
7.33
7.33
7.31
7.31
7.25
7.25
7.25
7.24
7.24
7.24
7.23
7.23
7.23
7.22
7.22
7.22
7.21
7.21 CDCl3
7.19
7.18
7.18
7.17
7.17
7.16
7.16
7.15
7.15
7.14
7.13
6.92
6.90
4.92
4.38
3.89
3.87
3.11
3.09
3.09
3.08
3.07
2.93
2.91
2.91
2.89
0.00 tms
N
O
O
O
12
^{1}H, 400 MHz, CDCl$_3$
1.09
1.06
1.07
1.13
1.24
3.00
6.30
2.00
1.95
3.05
3.08
2.04
2.05
f1 (ppm)

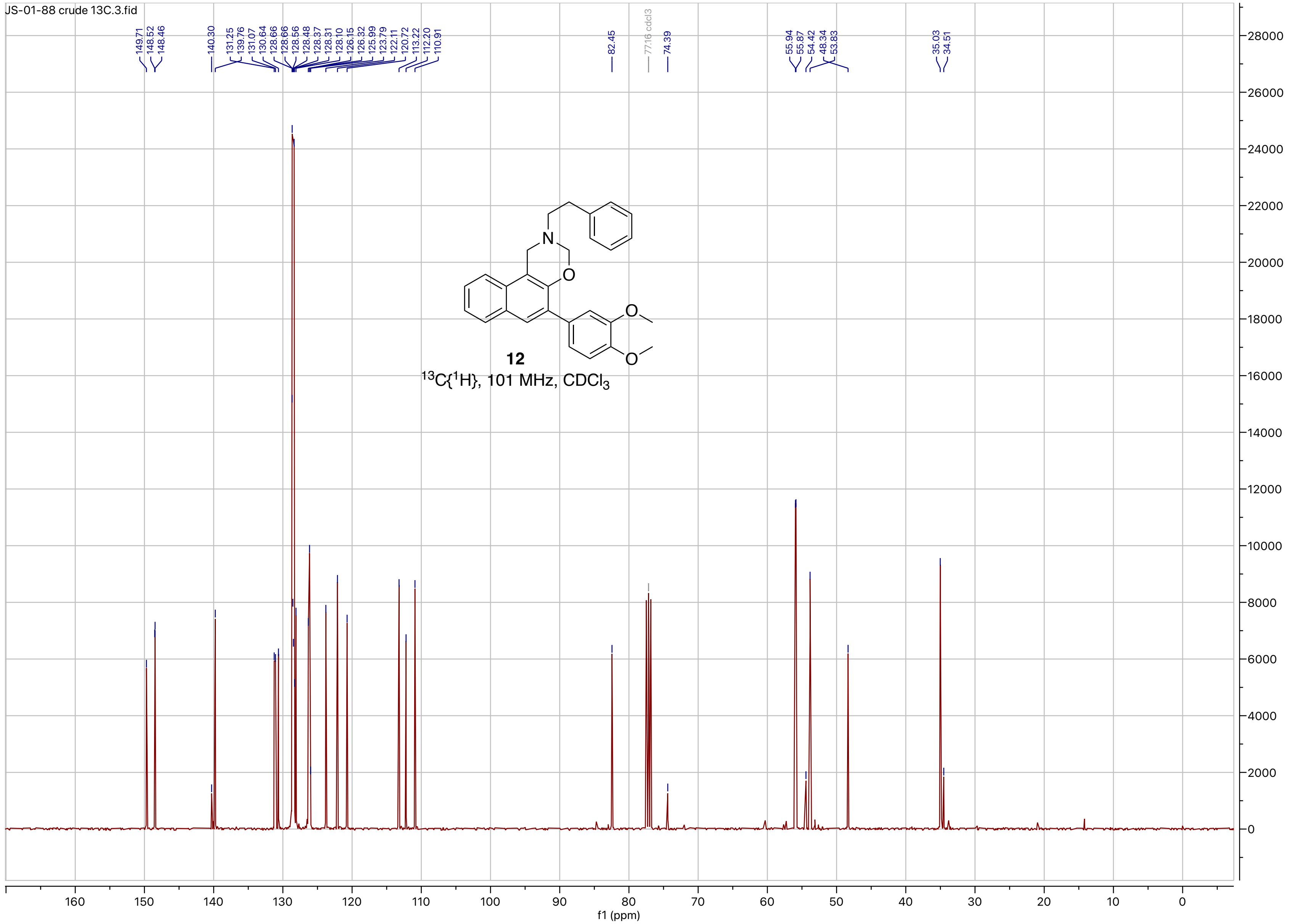
JS-01-88 crude 13C.3.fid
149.71 148.52 148.46 140.30 131.25 139.76 131.07 130.64 128.66 128.66 128.56 128.48 128.37 128.31 128.10 126.15 126.32 125.99 123.79 122.11 120.72 113.22 112.20 110.91
82.45 77.16 cdcl3 74.39
55.94 55.87 54.42 48.34 53.83
35.03 34.51
N
O
O
O
12
13C{1H}, 101 MHz, CDCl3
f1 (ppm)
